# Supplementary material for: Design, Synthesis, and Evaluation of Niclosamide Analogs as Therapeutic Agents for Enzalutamide-Resistant Prostate Cancer
Source: Pharmaceuticals (Basel). 2023 May 12;16(5):735. doi: 10.3390/ph16050735 (PMC10222209; doi:10.3390/ph16050735)
Supplement: Supplementary file 1 [file pharmaceuticals-16-00735-s001.zip › pharmaceuticals-2362863-supplementary.pdf]

Supporting Information

Design, Synthesis, and Evaluation of Niclosamide Analogs as Therapeutic Agents for Enzalutamide-Resistant Prostate Cancer

Borui Kang [1,2], Madhusoodanan Mottamal [1,2], Qiu Zhong[1,2], Melyssa Bratton[2], Changde Zhang [1,2], Shanchun Guo[1,2], Ahamed Hossain [2], Peng Ma [2], Qiang Zhang [1,2], Guangdi Wang [1,2], Florastina Payton-Stewart [1,2,*]*

1. Department of Chemistry, Xavier University of Louisiana, 1 Drexel Drive, New Orleans, Louisiana 70125, United States
2. RCMI Cancer Research Center, Xavier University of Louisiana, 1 Drexel Drive, New Orleans, Louisiana 70125, United States

* Correspondence: gwang@xula.edu and flpayton@xula.edu

1. General Information

Abbreviations and Symbol

aq	Aqueous solution
b	Broad
d	Doublet
DCM	Dichloromethane
DMAP	Dimethylaminopyridine
DMF	Dimethyl formamide
EA	Ethyl acetate
HRMS	High resolution mass spectra
m	Multiplet
NIH	National Institutes of Health
NMR	Nuclear magnetic resonance
q	Quartet
r.t.	Room temperature
s	Singlet
t	Triplet
Tf_2O	Trifluoromethanesulfonic anhydride
THF	Tetrahydrofuran
TMS	Tetramethylsilane

2. General Procedures for Preparation of Niclosamide Analogs A1-20, B1, B3-B16, C1-C5

To the solution of substituted benzoic acid (1.00 mmol) in THF (15 ml) was added $SOCl_2$ in THF (5 ml dissolved in 5 mL THF) dropwise at 0°C under N_2. The reaction mixture was stirred at r.t. for 1.5h. The solvent was removed by reduced pressure distillation to obtain white to off-white solid or semi-solid. The residue was dissolved in THF (10 ml), added to the mixture of substituted aniline and DMAP (cat.) in THF dropwise at 0°C under N_2. The reaction mixture was stirred at r.t. for 1.5 h or overnight. The solvent was removed under vacuum. And the residue was suspended in EA (10 ml) and washed with HCl (aq., 2M), saturated $NaHCO_3$ (aq.), and saturated NaCl (aq.). The organic fraction was dried over $MgSO_4$, concentrated, and purified by flash column chromatography on silica gel with a gradient eluent of hexane and EA.

2.1 Preparation of N-(4-amino-2-chlorophenyl)-5-chloro-2-hydroxybenzamide (B2)

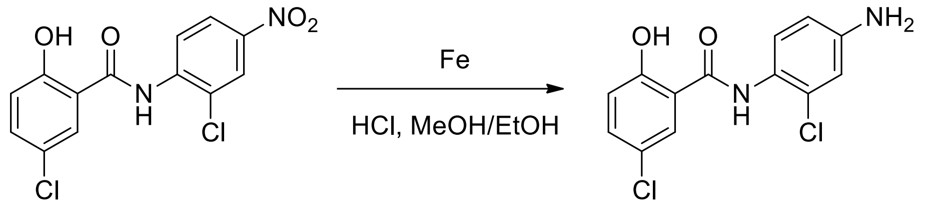

niclosamide **B2**

The reaction mixture of Niclosamide (1.0 g, 3.05 mmol), Fe powder (0.85g, 15.25 mmol) and HCl (aq., 1 ml, 12M) in methanol (8 ml) and ethanol (20 ml) was refluxed for 2 h. The suspension was filtered and washed with methanol. The combined filtrates were evaporated and purified by flash column chromatography on silica gel with a gradient eluent of hexane and EA to obtain the desired compound as pale yellow solid (0.86 g, 95.1%).

2.2 Preparation of niclosamide acetate (C7)

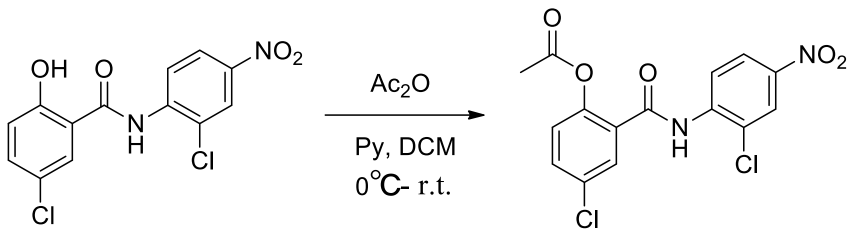

To the solution of Niclosamide (0.33 g, 1.00 mmol) and pyriding (1.00 mmol) in DCM was added acetic anhydride (1.10 mmol) dropwise at 0°C. The reaction mixture was refluxed for 1.0 h, and evaporated. The residue was dissolved in EA, washed with HCl (aq., 2M), saturated Na_2CO_3 (aq.), saturated NaCl (aq.), and dried over $MgSO_4$. The organic fraction was concentrated and purified by flash column chromatography on silica gel with a gradient eluent of hexane and EA to obtain the desired compound as colorless solid (0.29 g, 90.0%).

2.3 Preparation of 4-chloro-2-((2-chloro-4-nitrophenyl)carbamoyl)phenyltrifluoromethane sulfonate (C6) and niclosamide triflate (C8)

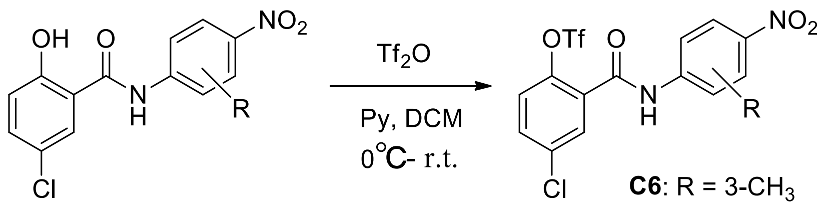

C6: R = 3-CH$_3$
C8: R = 2-Cl

To the solution of Niclosamide or 4-chloro-2-hydroxy-N-(3-methyl-4-nitrophenyl)benzamide (3.00 mmol) and pyriding (1.00 mmol) in DCM was added Tf_2O solution (1.00 mL in 5.0 mL DCM) dropwise at 0°C under N_2. The reaction mixture was stirred r.t. for 1.5 h, then washed with HCl (aq., 2M), saturated Na_2CO_3 (aq.), and saturated NaCl (aq.). The organic fraction was dried over $MgSO_4$, concentrated and purified by flash column chromatography on silica gel with a gradient eluent of hexane and EA to obtain the desired compound as off-white solid (yield: 90.1% (**C6**); 97.7%(**C8**)).

3. . Analytical Data

^1H and ^{13}C NMR spectra were recorded on n Agilent 400-MR NMR spectrometer (400 MHz and 100 MHz, respectively), Bruker 300-MR NMR spectrometer (300 MHz and 75 MHz, respectively), and Bruker 400-MR NMR spectrometer (400 MHz and 100 MHz, respectively). The data was processed using MestReNova NMR software (School of Chemistry, University of Bristol, Bristol, UK). Chemical shifts are reported as parts per million (ppm) relative to TMS (0.00 ppm) or residual undeuterated solvent signal. HRMS spectra data were collected on a Thermo LTQ Orbitrap-XL mass spectrometer in positive ion mode.

3.1. 4-Chloro-N-(2-fluoro-4-nitrophenyl)-2-hydroxybenzamide (A1)

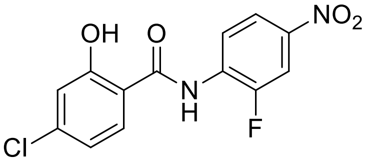

A1

The compound was purified by flash column chromatography on silica gel (Hexane/EA = 4/1, Rf = 0.36) as an off-white solid (yield: 51.7 %). **^{1}H NMR** (400 MHZ, DMSO-d_6) δ 6.96 (m, 2H), 7.95 (d, J = 8.0 Hz, 1H), 8.15 (d, J = 8.0 Hz, 1H), 8.21 (d, J = 8.0 Hz, 1H), 8.69 (b, 1H), 11.74 (b, 1H). **^{13}C NMR** (100 MHz, DMSO-d_6) δ 111.50, 117.60, 118.32, 120.75, 121.53, 132.69, 134.40, 138.49, 142.10, 150.04, 152.50, 161.13, 164.43. **HRMS** (ESI$^+$) m/z Calcd for C13H9ClFN2O4 $[M+H]^+$ 311.0235, found 311.0243.

3.2. 4-Chloro-2-hydroxy-N-(2-methyl-4-nitrophenyl)benzamide (A2)

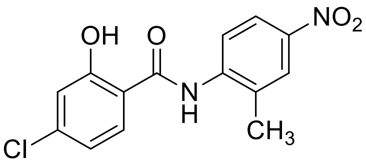

A2

The compound was purified by flash column chromatography on silica gel (Hexane/EA = 5/1, Rf = 0.38) as an off-white solid. (yield: 57.2 %). **^{1}H NMR** (400 MHz, $CDCl_3$) δ 2.59 (s, 3H), 7.01 (dd, J = 8.0 Hz, 4.0 Hz, 1H), 7.11 (d, J = 4.0 Hz, 1H), 7.45 (d, J = 8.0 Hz, 1H), 7.57 (d, J = 8.0 Hz, 1H), 7.60 (s, 1H), 8.08 (d, J = 8.0 Hz, 2H), 11.45 (s, 1H). **^{13}C NMR** (100 MHz, DMSO-d_6) δ 20.80, 117.06, 118.50, 118.62, 119.85, 124.36, 127.67, 131.77, 135.23, 137.87, 143.35, 144.11, 158.40, 165.87. **HRMS (**ESI$^+$) m/z Calcd for C14H12ClN2O4 $[M+H]^+$ 307.0486, found 307.0459.

3.3. 4-Chloro-2-hydroxy-N-(2-methoxy-4-nitrophenyl)benzamide (A3)

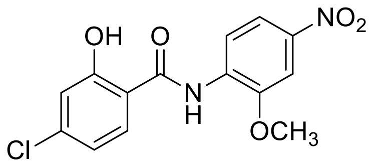

A3

The compound was purified by flash column chromatography on silica gel (Hexane/EA = 4/1, Rf = 0.20) as an off-white solid (yield: 78.5 %). **^{1}H NMR** (400 MHZ, DMSO-d_6) δ 4.02 (s, 3H), 7.04 (d, *J* = 8.0 Hz, 2H), 7.84 (d, *J* = 4.0 Hz, 1H), 7.54 (dd, *J* = 8.0 Hz, 4.0 Hz, 1H), 8.00 (d, *J* = 8.0 Hz, 1H), 8.70 (d, *J* = 8.0 Hz, 1H), 11.24 (s, 1H). **^{13}C NMR** (100 MHz, DMSO-d_6) δ 57.24, 106.07, 117.07, 117.80, 117.98, 118.66, 120.25, 133.23, 134.87, 138.25, 142.80, 148.30, 157.70, 163.17. **HRMS** (ESI$^+$) m/z Calcd for C14H12ClN2O5 [M+H]$^+$ 323.0435, found 323.0442.

3.4. 4-Chloro-N-(3-chloro-4-nitrophenyl)-2-hydroxybenzamide (A4)

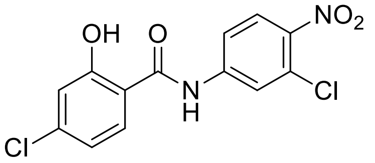

A4

The compound was purified by flash column chromatography on silica gel (Hexane/EA = 4/1, Rf = 0.33) as off-white solid (yield: 88.1 %). **^{1}H NMR** (400 MHz, DMSO-d_6) δ 7.02 (d, *J* = 8.0 Hz, 2H), 7.79 (d, *J* = 8.0 Hz, 1H), 7.84 (dd, *J* = 8.0 Hz, 4.0 Hz, 1H), 8.14 (s, 1H), 8.17 (d, *J* = 8.0 Hz, 1H), 10.76 (s, 1H), 11.63(b, 1H). **^{13}C NMR** (100 MHz, DMSO-d_6) δ 117.03, 118.80, 119.27, 119.88, 121.95, 127.12, 127.87, 131.82, 137.96, 142.28, 143.82, 158.19, 165.89. **HRMS** (ESI$^+$) m/z Calcd for C13H9Cl2N2O4 [M+H]$^+$ 326.9939, found 326.9942.

3.5. 4-Chloro-N-(3-fluoro-4-nitrophenyl)-2-hydroxybenzamide (A5)

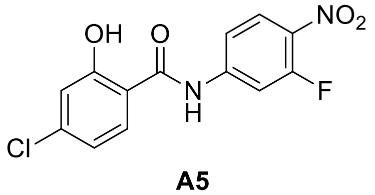

A5

The compound was purified by flash column chromatography on silica gel (Hexane/EA = 4/1, Rf = 0.29) as a yellow solid (yield: 76.5 %). **^{1}H NMR** (400 MHZ, DMSO-d_6) δ 7.03 (d, 2H), 7.65 (d, *J* = 12.0 Hz, 1H), 7.77 (d, *J* = 8.0 Hz, 1H), 8.00 (d, *J* = 12.0 Hz, 1H), 8.19 (d, *J* = 8.0 Hz, 1H), 10.87 (s, 1H). **^{13}C NMR** (100 MHz, DMSO-d_6) δ 108.40, 116.07, 117.00, 119.07, 119.88, 127.88, 131.90, 137.92, 146.00, 154.73, 157.32, 158.06, 165.85. **HRMS** (ESI$^+$) m/z Calcd for C13H9ClFN2O4 [M+H]$^+$ 311.0235, found 311.0235.

3.6. 4-Chloro-2-hydroxy-N-(4-nitro-3-(trifluoromethyl)phenyl)benzamide (A6)

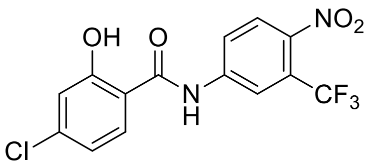

A6

The compound was purified by flash column chromatography on silica gel (Hexane/EA = 5/1, Rf = 0.31) as white solid (yield: 82.8 %). **^{1}H NMR** (400 MHz, $CDCl_3$) δ 6.96 (dd, *J* = 8.0 Hz, 4.0 Hz, 1H), 7.09 (s, 1H), 7.53 (d, *J* = 8.0 Hz, 1H), 8.04 (d, *J* = 8.0 Hz, 1H), 8.09 (d, *J* = 8.0 Hz, 2H), 8.36 (s, 1H), 11.48 (s, 1H). **^{13}C NMR** (100 MHz, DMSO-d_6) δ 110.03, 116.13, 117.63, 117.89, 118.91, 120.27, 122.96, 127.01, 130.81, 137.16, 141.26, 142.61, 157.43, 165.18. **HRMS** (ESI$^+$) m/z Calcd for C14H9ClF3N2O4 $[M+H]^+$ 361.0203, found 361.0213.

3.7. 4-chloro-2-hydroxy-N-(3-methyl-4-nitrophenyl)benzamide (A7)

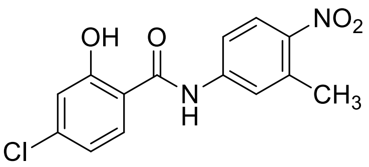

A7

The compound was purified by flash column chromatography on silica gel (Hexane/EA = 5/1, Rf = 0.27) as a white solid (yield: 70.7 %). **^{1}H NMR** (400 MHz, $CDCl_3$) δ 2.67 (s, 3H), 6.94 (dd, *J* = 8.0 Hz, 4.0 Hz, 1H), 7.07 (d, *J* = 4.0 Hz, 1H), 7.48 (d, *J* = 8.0 Hz, 1H), 7.61 (d, *J* = 8.0 Hz, 1H), 7.63 (s, 1H), 8.11 (d, *J* = 8.0 Hz, 2H), 11.76 (s, 1H). **^{13}C NMR** (100 MHz, DMSO-d_6) δ 21.12, 117.07, 118.50, 118.62, 119.86, 123.36, 126.67, 131.77, 135.32, 137.87, 143.35, 144.11, 158.40, 165.73. **HRMS** (ESI$^+$) m/z Calcd for C14H12ClN2O4 $[M+H]^+$ 307.0486, found 307.0490.

3.8. 4-chloro-2-hydroxy-N-(3-methoxy-4-nitrophenyl)benzamide (A8)

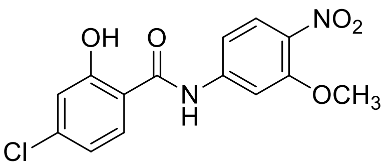

A8

The compound was purified by flash column chromatography on silica gel (Hexane/EA = 5/1, Rf = 0.22) as an off-white solid (yield: 87.9 %). **^{1}H NMR** (400 MHZ, DMSO-d_6) δ 4.02 (s, 3H), 6.94 (d, *J* = 8.0 Hz, 1H), 6.99 (d, *J* = 8.0 Hz, 1H), 7.08 (s, 1H), 7.49 (d, *J* = 8.0 Hz, 1H), 7.83 (s, 1H), 7.99 (d, *J* = 8.0 Hz, 1H), 8.14 (s, 1H), 11.70 (s, 1H). **^{13}C NMR** (100 MHz, DMSO-d_6) δ 56.85, 104.92, 111.81, 117.05, 118.73, 119.84, 127.28, 131.71, 134.26, 137.89, 144.94, 154.20, 158.34, 165.86. **HRMS** (ESI$^+$) m/z Calcd for C14H12ClN2O5

[M+H]$^+$ 323.0435, found 323.0440.

3.9. 5-chloro-2-hydroxy-N-(4-nitrophenyl)benzamide (A9)

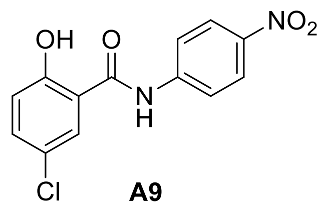

A9

The compound was purified by flash column chromatography on silica gel (Hexane/EA = 4/1, Rf = 0.58) as a yellow solid (yield: 89.0 %). **^{1}H NMR** (400 MHZ, DMSO-d_6) δ 7.02 (d, J = 8.0 Hz, 1H), 7.46 (dd, J = 8.0 Hz, 4.0 Hz, 1H), 7.80 (d, J = 4.0 Hz, 1H), 7.97 (d, J = 8.0 Hz, 2H), 8.25 (d, J = 8.0 Hz, 2H), 10.81 (s, 1H), 11.43 (s, 1H). **^{13}C NMR** (100 MHZ, DMSO-d_6) δ 119.35, 120.43, 121.15, 123.28, 125.27, 129.20, 133.49, 143.13, 144.93, 156.33, 165.42. **HRMS** (ESI$^+$) m/z Calcd for C13H10ClN2O4 [M+H]$^+$ 293.0329, found 293.0336.

3.10. 5-chloro-N-(2-fluoro-4-nitrophenyl)-2-hydroxybenzamide (A10)

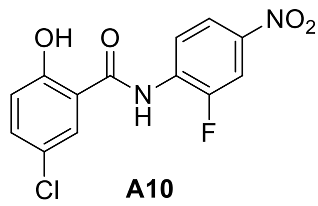

A10

The compound was purified by flash column chromatography on silica gel (Hexane/EA = 4/1, Rf = 0.27) as a light brown solid (yield: 56.1 %). **^{1}H NMR** (400 MHz, DMSO-d_6) δ 7.05 (d, J = 8.0 Hz, 1H), 7.50 (d, J = 8.0 Hz, 1H), 7.91 (s, 1H), 8.16 (d, J = 8.0 Hz, 1H), 8.24 (d, J = 8.0 Hz, 1H), 8.55 (t, J = 8.0 Hz, 1H), 11.12 (s, 1H), 12.36 (b, 1H). **^{13}C NMR** (100 MHz, DMSO-d_6) δ 111.50, 119.55, 119.84, 121.07, 121.46, 124.08, 130.25, 133.40, 134.29, 142.74, 150.14, 155.66, 163.09. **HRMS** (ESI$^+$) m/z Calcd for C13H9ClFN2O4 [M+H]$^+$ 311.0235, found 311.0241.

3.11. 5-Chloro-2-hydroxy-N-(4-nitro-2-(trifluoromethyl)phenyl)benzamide (A11)

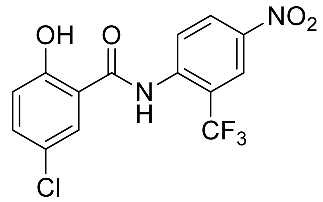

A11

The compound was purified by flash column chromatography on silica gel (Hexane/EA = 4/1, Rf = 0.50) as an off-white solid (yield: 55.4 %). **^{1}H NMR** (400 MHz, DMSO-d_6) δ 7.01 (d, J = 8.0 Hz, 1H), 7.44 (dd, J = 8.0 Hz, 4.0 Hz, 1H), 7.83 (d, J = 4.0 Hz, 1H), 8.15-8.22 (m, 2H), 8.47 (s, 1H), 11.21 (s, 1H). **^{13}C NMR** (100

MHz, DMSO-d_6) δ 108.03, 116.53, 117.17, 118.01, 119.03, 121.17, 123.06, 127.11, 131.81, 136.16, 140.26, 143.61, 156.33, 163.44. **HRMS** (ESI$^+$) m/z Calcd for C14H9ClF3N2O4 [M+H]$^+$ 361.0203, found 361.0211.

3.12. 5-Chloro-2-hydroxy-N-(2-methyl-4-nitrophenyl)benzamide (A12)

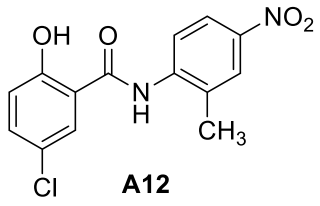

A12

The compound was purified by flash column chromatography on silica gel (Hexane/EA = 4/1, Rf = 0.19) as an off-white solid (yield: 45.7 %). **^{1}H NMR** (400 MHz, DMSO-d_6) δ 1.96 (s, 3H), 7.06 (d, *J* = 8.0 Hz, 1H), 7.49 (d, *J* = 8.0 Hz, 1H), 7.94 (s, 1H), 8.14 (d, *J* = 12.0 Hz, 1H), 8.19 (s, 1H), 8.49 (d, *J* = 8.0 Hz, 1H), 10.73 (s, 1H), 12.30 (b, 1H). **^{13}C NMR** (100 MHz, DMSO-d_6) δ 21.21, 119.56, 120.36, 121.06, 122.96, 123.93, 125.84, 129.52, 133.98, 143.09, 143.41, 155.84, 163.23, 170.80. **HRMS** (ESI$^+$) m/z Calcd for C14H12ClN2O4 [M+H]$^+$ 307.0486, found 307.0493.

3.13. 5-Chloro-2-hydroxy-N-(2-methoxy-4-nitrophenyl)benzamide (A13)

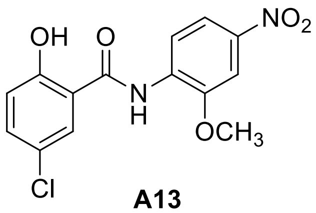

A13

The compound was purified by flash column chromatography on silica gel (Hexane/EA = 4/1, Rf = 0.17) as an off-white solid (yield: 77.9 %). **^{1}H NMR** (400 MHz, $CDCl_3$) δ 4.11 (s, 3H), 7.02 (d, *J* = 8.0 Hz, 1H), 7.44 (dd, d, *J* = 8.0 Hz, 4.0 Hz, 1H), 7.49 (d, *J* = 4.0 Hz, 1H), 7.83 (d, *J* = 4.0 Hz, 1H), 7.98 (dd, d, *J* = 8.0 Hz, 4.0 Hz, 1H), 8.61 (d, *J* = 8.0 Hz, 1H), 8.78 (b, 1H), 11.62 (s, 1H). **^{13}C NMR** (100 MHz, DMSO-d_6) δ 57.29, 106.14, 117.78, 118.77, 119.49, 120.28, 124.08, 130.46, 134.00, 134.66, 142.97, 148.36, 155.39, 162.66. **HRMS** (ESI$^+$) m/z Calcd for C14H12ClN2O5 [M+H]$^+$ 323.0435, found 323.0445.

3.14. 5-Chloro-N-(3-chloro-4-nitrophenyl)-2-hydroxybenzamide (A14)

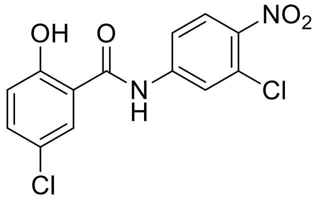

A14

The compound was purified by flash column chromatography on silica gel (Hexane/EA = 4/1, Rf = 0.44) as a yellow solid (yield: 89.6%). **^{1}H NMR** (400

MHz, DMSO-d_6) δ 7.02 (d, *J* = 12.0 Hz, 1H), 7.46 (dd, *J* = 8.0 Hz, 4.0 Hz, 1H), 7.77 (d, *J* = 4.0 Hz, 1H), 7.84 (dd, *J* = 8.0 Hz, 4.0 Hz, 1H), 8.15 (d, 2H), 10.82 (s, 1H), 11.37 (b, 1H). **^{13}C NMR** (100 MHz, DMSO-d_6) δ 119.28, 119.35, 121.06, 121.99, 123.28, 127.11, 127.85, 129.20, 133.60, 142.34, 143.73, 156.27, 165.52. **HRMS** (ESI$^+$) m/z Calcd for C13H9Cl2N2O4 [M+H]$^+$ 326.9939, found 326.9944.

3.15. 5-Chloro-N-(3-fluoro-4-nitrophenyl)-2-hydroxybenzamide (A15)

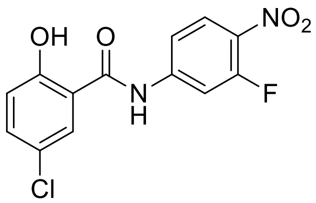

A15

The compound was purified by flash column chromatography on silica gel (Hexane/EA = 4/1, Rf = 0.39) as a yellow solid (yield: 77.1%).

^{1}H NMR (400 MHz, DMSO-d_6) δ 7.01 (d, *J* = 12.0 Hz, 1H), 7.45 (dd, *J* = 8.0 Hz, 4.0 Hz, 1H), 7.65 (d, *J* = 8.0 Hz, 1H), 7.75 (s, 1H), 7.98 (d, *J* = 12.0 Hz, 1H), 8.19 (t, *J* = 8.0 Hz, 1H), 10.90 (s, 1H), 11.36 (b, 1H). **^{13}C NMR** (100 MHz, DMSO-d_6) δ 108.36, 108.62, 116.06, 116.09, 119.33, 123.28, 127.82, 131.97, 132.04, 133.58, 145.85, 145.97, 154.71, 156.14, 157.29, 165.46, 170.76. **HRMS** (ESI$^+$) m/z Calcd for C13H9ClFN2O4 [M+H]$^+$ 311.0235, found 311.0237.

3.16. 5-chloro-2-hydroxy-N-(4-nitro-3-(trifluoromethyl)phenyl)benzamide (A16)

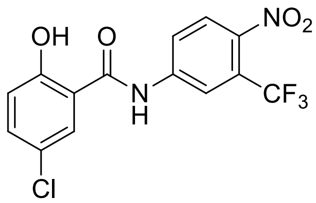

A16

The compound was purified by flash column chromatography on silica gel (Hexane/EA = 4/1, Rf = 0.42) as a yellow solid (yield: 81.3%). **^{1}H NMR** (400 MHz, DMSO-d_6) δ 7.02 (d, *J* = 8.0 Hz, 1H), 7.46 (dd, *J* = 8.0 Hz, 4.0 Hz, 1H), 7.78 (d, *J* = 4.0 Hz, 1H), 8.19-8.24 (m, 2H), 8.41 (s, 1H), 11.06 (s, 1H). **^{13}C NMR** (100 MHz, DMSO-d_6) δ 118.90, 119.42, 120.99, 123.10, 123.42, 123.90, 127.97, 129.17, 133.64, 142.25, 143.51, 156.52, 165.77. **HRMS** (ESI$^+$) m/z Calcd for C14H9ClF3N2O4 [M+H]$^+$ 361.0203, found 361.0214.

3.17. 5-chloro-2-hydroxy-N-(3-methyl-4-nitrophenyl)benzamide (A17)

The compound was purified by flash column chromatography on silica gel (Hexane/EA = 4/1, Rf = 0.29) as an off-white solid (yield: 55.0%). **^{1}H NMR** (400 MHz, DMSO-d_6) δ 2.55 (s, 3H), 7.01 (d, *J* = 12.0Hz, 1H), 7.46 (dd, *J* = 8.0Hz, 4.0 Hz, 1H), 7.79 (s, 1H), 7.81-7.83 (m, 2H), 8.07 (d, *J* = 12.0Hz, 1H), 10.71 (s, 1H), 11.50 (b, 1H). **^{13}C NMR** (100 MHz, DMSO-d_6) δ 21.21, 118.54, 119.41, 121.08, 123.25, 123.40, 126.69, 129.19, 133.53, 135.32, 143.28, 144.21, 156.41, 165.35. **HRMS** (ESI$^+$) m/z Calcd for C14H12ClN2O4 $[M+H]^+$ 307.0486, found 307.0493.

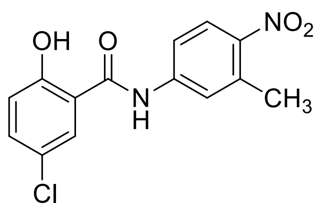

A17

3.18. 5-Chloro-2-hydroxy-N-(3-methoxy-4-nitrophenyl)benzamide (A18)

The compound was purified by flash column chromatography on silica gel (Hexane/EA = 4/1, Rf = 0.52) as a yellow solid (yield: 78.6%). **^{1}H NMR** (400 MHz, DMSO-d_6) δ 3.91 (s, 3H), 7.03 (d, *J* = 12.0 Hz, 1H), 7.42 (dd, *J* = 8.0Hz, 4.0 Hz, 1H), 7.46 (dd, *J* = 8.0Hz, 4.0 Hz, 1H), 7.80 (m, 2H), 7.97 (d, *J* = 8.0 Hz, 1H), 10.71 (s, 1H), 11.45 (b, 1H). **^{13}C NMR** (100 MHz, DMSO-d_6) δ 56.87, 104.97, 111.84, 119.36, 121.09, 123.27, 127.28, 129.14, 133.52, 134.34, 144.85, 154.17, 156.37, 165.49. **HRMS** (ESI$^+$) m/z Calcd for C14H12ClN2O5 $[M+H]^+$ 323.0435, found 323.0445.

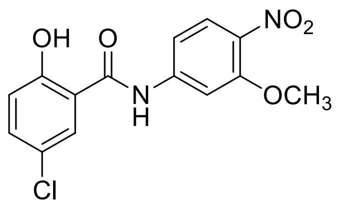

A18

3.19. 5-Bromo-N-(2-chloro-4-nitrophenyl)-2-hydroxybenzamide (A19)

The compound was purified by flash column chromatography on silica gel (Hexane/EA = 4/1, Rf = 0.35) as a white solid (yield: 92.7%). **^{1}H NMR** (400 MHz, $CDCl_3$) δ 6.94 (d, *J* = 8.0 Hz, 2H), 7.14 (d, *J* = 8.0 Hz, 2H), 7.61 (d, *J* = 8.0 Hz, 2H), 7.78 (d, *J* = 8.0 Hz, 2H), 8.17 (s, 1H), 8.27 (s, 1H), 10.14 (s, 1H). **^{13}C NMR** (100 MHz, DMSO-d_6) δ 110.32, 116.40, 119.03, 120.50, 126.65, 126.91, 133.54, 134.14, 136.98, 138.41, 149.26, 159.16, 154.40. **HRMS** (ESI$^-$) m/z Calcd for C13H7BrClN2O4 $[M-H]^-$ 368.9278, found 368.9286.

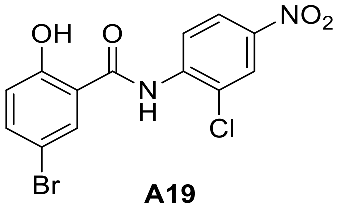

A19

3.20. 5-Bromo-2-hydroxy-N-(2-methoxy-4-nitrophenyl)benzamide (A20)

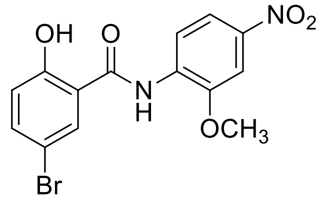

A20

The compound was purified by flash column chromatography on silica gel (Hexane/EA = 4/1, Rf = 0.35) as a white solid (yield: 81.5%). **^{1}H NMR** (400 MHz, DMSO-d_6) δ 4.02 (s, 3H), 7.00 (d, J = 8.0 Hz, 1H), 7.60 (dd, J = 8.0 Hz, 4.0 Hz, 1H), 7.84 (d, J = 4.0 Hz, 1H), 7.95 (dd, J = 8.0 Hz, 4.0 Hz, 1H), 8.05 (d, J = 4.0 Hz, 1H), 11.27 (s, 1H), 12.35 (s, 1H). **^{13}C NMR** (100 MHz, DMSO-d_6) δ 57.29, 106.15, 111.30, 117.79, 118.78, 119.98, 120.75, 133.41, 134.71, 136.80, 142.95, 148.38, 156.18, 162.69. **HRMS** (ESI$^+$) m/z Calcd for C14H12BrN2O5 [M+H]$^+$ 366.9930, found 326.9946.

3.21. N-(3,4-bis(trifluoromethyl)phenyl)-5-chloro-2-hydroxybenzamide (B1)

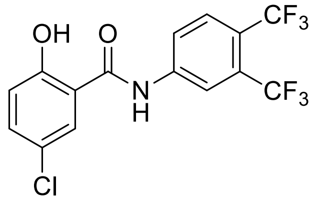

B1

The compound was purified by flash column chromatography on silica gel (Hexane/EA = 4/1, Rf = 0.44) as a colorless solid (yield: 87.9%). **^{1}H NMR** (400 MHz, CDCl3) δ 7.03 (d, J = 12.0 Hz, 1H), 7.45 (dd, J = 8.0 Hz, 4.0 Hz, 1H), 7.55 (d, J = 4.0 Hz, 1H), 7.89 (d, J = 8.0 Hz, 1H), 8.05 (d, J = 8.0 Hz, 1H), 8.08 (s, 1H), 8.19 (b, 1H), 11.36 (s, 1H). **^{13}C NMR** (100 MHz, DMSO-d_6) δ 119.38, 129.90, 121.75, 122.01, 123.23, 124.48, 124.72, 127.16, 127.47, 129.09, 129.96, 133.60, 142.91, 156.47, 165.84. **HRMS** (ESI$^+$) m/z Calcd for C15H9ClF6NO2 [M+H]$^+$ 384.0226, found 384.0227.

3.22. N-(4-amino-2-chlorophenyl)-5-chloro-2-hydroxybenzamide (B2)

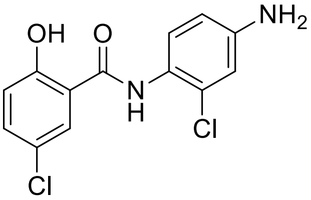

B2

The compound was recrystallized from Hexane/EA mixture (Hexane/EA = 4/1, Rf = 0.56) as a colorless solid (yield: 95.1%). **^{1}H NMR** (400 MHz, DMSO-d_6) δ 5.35 (b, 2H), 6.52 (dd, J = 8.0 Hz, 4.0 Hz, 1H), 6.68 (d, J = 4.0 Hz, 1H), 6.98 (d, J = 8.0 Hz, 1H), 7.43 (dd, J = 8.0 Hz, 4.0 Hz, 1H), 7.66 (d, J = 8.0 Hz, 1H), 7.97 (d, J = 4.0 Hz, 1H), 10.52 (b, 1H). **^{13}C NMR** (100 MHz, DMSO-d_6) δ 113.22, 113.82, 119.32, 119.69, 123.01, 123.25, 126.34, 126.81,

129.30, 133.49, 147.83, 157.50, 164.16. HRMS (ESI^{+}) m/z Calcd for C13H11Cl2N2O2 [M+H]$^{+}$ 297.0198, found 297.0201.

3.23. 5-Chloro-N-(4-cyano-3-(trifluoromethyl)phenyl)-2-hydroxybenzamide (B3)

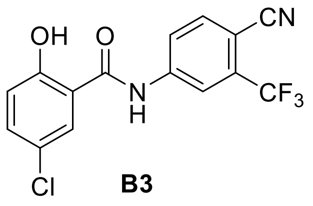

B3

The compound was purified by flash column chromatography on silica gel (Hexane/EA = 4/1, Rf = 0.43) as a colorless solid (yield: 90.1%). **^{1}H NMR** (400 MHz, DMSO-d_6) δ 7.00 (d, *J* = 12.0 Hz, 1H), 7.46 (dd, *J* = 8.0 Hz, 4.0 Hz, 1H), 7.77 (d, *J* = 4.0 Hz, 1H), 8.14 (s, 2H), 8.40 (s, 1H), 10.94 (s, 1H), 11.32 (b, 1H). **^{13}C NMR** (100 MHz, DMSO-d_6) δ 106.19, 107.38, 116.18, 117.99, 119.36, 121.28, 123.23, 123.55, 129.20, 132.00, 133.59, 136.89, 143.49, 156.24, 165.74. HRMS (ESI^{+}) m/z Calcd for C15H8ClF3N2O2 [M+H]$^{+}$ 341.0304, found 341.0303.

3.24. N-(3,4-bis(trifluoromethyl)phenyl)-5-bromo-2-hydroxybenzamide (B4)

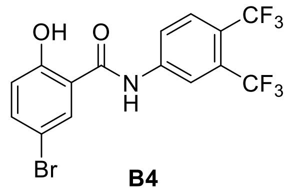

B4

The compound was purified by flash column chromatography on silica gel (Hexane/EA = 4/1, Rf = 0.47) as a colorless solid (yield: 92.7%). **^{1}H NMR** (400 MHz, DMSO-d_6) δ 6.96 (d, *J* = 8.0 Hz, 1H), 7.57 (d, *J* = 8.0 Hz, 1H), 7.92 (s, 1H), 8.03 (d, *J* = 8.0 Hz, 1H), 8.20 (d, *J* = 8.0 Hz, 1H), 8.42 (s, 1H), 10.96 (s, 1H), 11.38 (b, 1H). **^{13}C NMR** (100 MHz, DMSO-d_6) δ 110.61, 119.43, 119.49, 119.81, 121.49, 123.55, 129.93, 129.99, 131.94, 136.41, 142.93, 156.92, 165.81. **HRMS** (ESI^{+}) m/z Calcd for C15H9BrF6NO2 [M + H]$^{+}$ 427.9721, found 427.9717.

3.25. N-(2-chloro-4-(trifluoromethyl)phenyl)-5-fluoro-2-hydroxybenzamide (B5)

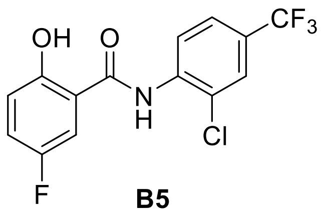

B5

The compound was purified by flash column chromatography on silica gel (Hexane/EA = 4/1, Rf = 0.48) as a colorless solid (yield: 80.4%). **^{1}H NMR** (400 MHz, DMSO-d_6) δ 7.04 (dd, *J* = 8 Hz, 4Hz, 1H), 7.34 (t, *J* = 8 Hz, 1H), 7.70 (dd, *J* = 8 Hz, 4Hz, 1H), 7.76 (d, *J* = 8 Hz, 1H), 7.96 (t, *J* = 8 Hz, 1H), 8.72 (d, *J* = 8 Hz, 1H), 11.23 (s, 1H), 12.11 (bs, 1H). ^{13}C NMR (100 MHz, DMSO-*d6*) δ 116.02, 118.70, 119.37, 121.28, 122.70, 124.61, 124.78, 125.14,

126.33, 138.81, 152.59, 154.32, 156.67, 162.60. HRMS (ESI$^+$) m/z Calcd for C14H8ClF4NO2 [M+H]$^+$ 334.0258, found 334.0252.

3.26. 5-Fluoro-N-(2-fluoro-4-(trifluoromethyl)phenyl)-2-hydroxybenzamide (B6)

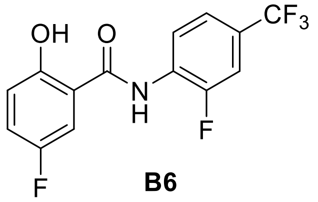

B6

The compound was purified by flash column chromatography on silica gel (Hexane/EA = 4/1, Rf = 0.40) as a colorless solid (yield: 70.0%). **^{1}H NMR** (400 MHz, DMSO-*d6*) δ 7.14 (dd, *J* = 8 Hz, 4Hz, 1H), 7.54 (d, *J* = 4 Hz, 1H), 7.67 (dd, *J* = 8 Hz, 4Hz, 1H), 7.80 (d, *J* = 8 Hz, 1H), 8.05 (d, *J* = 4 Hz, 1H), 8.72 (d, *J* = 8 Hz, 1H), 10.99 (s, 1H), 11.98 (s, 1H). ^{13}C NMR (100MHz, DMSO-*d6*) δ 112.70, 115.90, 118.70, 118.82, 121.10, 122.16, 124.70, 130.20, 150.73, 152.76, 153.17, 154.25, 156.60, 162.92. HRMS (ESI$^+$) m/z Calcd for C14H8F5NO2 [M+H]$^+$ 318.0553, found 318.0551.

3.27. N-(2-cyano-4-(trifluoromethyl)phenyl)-5-fluoro-2-hydroxybenzamide (B7)

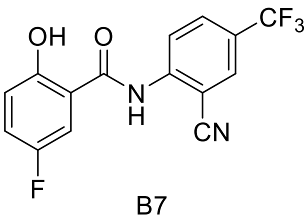

B7

The compound was purified by flash column chromatography on silica gel (Hexane/EA = 4/1, Rf = 0.30) as a colorless solid (yield: 80.0%). **^{1}H NMR** (400 MHz, DMSO-*d6*) δ 7.14 (dd, *J* = 8 Hz, 4Hz, 1H), 7.56 (d, *J* = 4 Hz, 1H), 7.70 (d, *J* = 8 Hz, 4Hz, 1H), 7.79 (d, *J* = 8 Hz, 1H), 8.09 (s, 1H), 8.56 (d, *J* = 8 Hz, 1H), 11.01 (s, 1H), 12.05 (s, 1H). ^{13}C NMR (100 MHz, DMSO-*d6*) δ 104.57, 113.50, 117.80, 118.97, 121.40, 123.50, 123.47, 124.08, 125.00, 126.2, 130.00, 152.00, 155.72, 157.67, 164.60. **HRMS** (ESI$^+$) m/z Calcd for C15H9F4N2O2 [M+H]$^+$, 325.0600, found 325.0598.

3.28. N-(3-cyano-4-(trifluoromethyl)phenyl)-5-fluoro-2-hydroxybenzamide (B8)

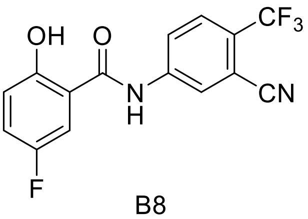

B8

The compound was purified by flash column chromatography on silica gel (Hexane/EA = 4/1, Rf = 0.50) as a colorless solid (yield: 67.0%). **^{1}H NMR** (400 MHz, DMSO-*d6*) δ 7.04 (m, 1H), 7.33 (m, 1H), 7.63 (m, 1H), 8.20 (d, *J* = 8.8 Hz,

1H), 8.22 (d, *J* = 8.8 Hz, 1H), 10.87 (s, 1H), 11.24 (b, 1H). **^{13}C NMR** (100 MHz, DMSO-*d_6*) δ 109.20, 115.08, 115.33, 118.50, 119.40, 120.60, 124.10, 124.98, 125.30, 128.20, 142.43, 153.41, 153.79, 156.13, 165.18. **HRMS** (ESI$^+$) m/z Calcd for C15H8F4N2O2 [M+H]$^+$ 325.0600, found 325.0599.

3.29. N-(3,4-bis(trifluoromethyl)phenyl)-5-fluoro-2-hydroxybenzamide (B9)

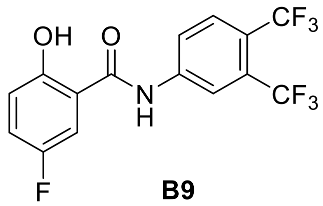

B9

The compound was purified by flash column chromatography on silica gel (Hexane/EA = 4/1, Rf = 0.35) as a white solid (yield: 78.0%). **^{1}H NMR** (400 MHz, DMSO-*d_6*) δ 7.01 (q, *J* = 8.0 Hz, 1H), 7.31 (t, *J* = 8.0 Hz, 1H), 7.62 (dd, *J* = 8.0 Hz, 4.0 Hz, 1H), 8.03 (d, *J* = 8.0 Hz, 1H), 8.20 (d, *J* = 8.0 Hz, 1H), 8.44 (s, 1H), 10.86 (s, 1H), 11.17 (s, 1H). **^{13}C NMR** (100 MHz DMSO-*d_6*) δ 115.41, 115.66, 118.88, 118.96, 119.40, 119.47, 119.78, 119.85, 120.93, 121.16, 123.55, 129.93, 129.99, 142,91, 154.00, 154.17, 156.51, 165.88. **HRMS** (ESI$^+$) m/z Calcd for C15H8F7NO2 [M+H]$^+$ 368.0521, found 368.0520.

3.30. 5-Fluoro-2-hydroxy-N-(6-(trifluoromethyl)pyridin-3-yl)benzamide (B10)

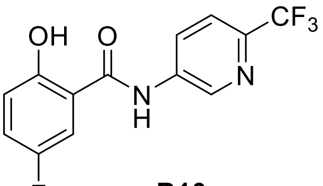

B10

The compound was purified by flash column chromatography on silica gel (Hexane/EA = 4/1, Rf = 0.35) as a yellow solid (yield: 54.1%). **^{1}H NMR** (400 MHz, DMSO-*d_6*) δ 7.02 (dd, *J* = 8 Hz, 4Hz, 1H), 7.30 (t, *J* = 8 Hz, 1H), 7.64 (dd, *J* = 8 Hz, 4Hz, 1H), 7.91 (d, *J* = 8 Hz, 1H), 8.44 (t, *J* = 8 Hz, 1H), 9.00 (s, 1H), 10.79 (s, 1H), 11.30 (bs, 1H). **^{13} C NMR** (100 MHz, DMSO-*d_6*) δ 115.20, 118.50, 119.10, 120.60, 121.16, 123.17, 128.60, 137.92, 140.70, 141.50, 153.70, 156.12, 165.30. **HRMS** (ESI$^+$) m/z Calcd for C13H8F4N2O2 [M+H]$^+$ 301.0600, found 301.0599.

3.31. 5-Fluoro-2-hydroxy-N-(2-(trifluoromethyl)pyridin-4-yl)benzamide (B11)

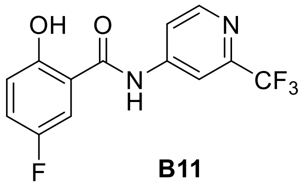

B11

The compound was purified by flash column chromatography on silica gel (Hexane/EA = 4/1, Rf = 0.36) as a yellow solid (yield: 50.2%). **^{1}H NMR** (400 MHz, DMSO-*d_6*) δ 7.02 (dd, *J* = 8 Hz, 4Hz, 1H), 7.32 (t, *J* = 8 Hz, 1H), 7.67 (dd, *J* = 8 Hz,

4Hz, 1H), 7.71 (d, J = 8 Hz, 1H), 7.95 (d, J = 8 Hz, 1H), 8.56 (t, J = 8 Hz, 1H), 11.07 (s, 1H), 12.05 (bs, 1H). **^{13} C NMR** (100 MHz, DMSO-d_6) δ107.60, 109.50, 113.70, 119.00, 119.67, 121.50, 123.10, 145.40, 150.00, 154.98, 155.10, 155.90, 164.60. **HRMS** (ESI$^+$) m/z Calcd for C13H8F4N2O2 $[M+H]^+$ 301.0600, found 301.0598.

3.32. N-(2-chloro-4-cyanophenyl)-5-fluoro-2-hydroxybenzamide (B12)

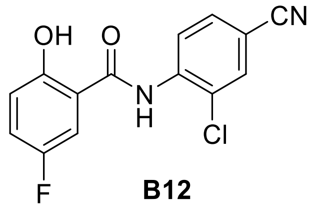

B12

The compound was purified by flash column chromatography on silica gel (Hexane/EA = 4/1, Rf = 0.42) as an off-white solid (yield: 40.9%). **^{1}H NMR** (400 MHz, DMSO-d_6) δ 7.02 (d, J = 8 Hz, 1H), 7.53 (d, J = 8 Hz, 1H), 7.72 (d, J = 8 Hz, 1H), 7.86 (d, J = 8 Hz, 1H), 7.99 (s, 1H), 8.17 (s, 1H), 11.03 (s, 1H), 12.09 (b, 1H). **^{13}C NMR** (100 MHz, DMSO-d_6) δ 13 C NMR (100 MHz, DMSO-d6) δ 109.40, 113.67, 118.50, 119.00, 119.60, 121.40, 123.70, 124.10, 131.00, 131.57, 132.98, 139.10, 155.00, 164.67. **HRMS** (ESI$^+$) m/z Calcd for C14H9ClFN2O2 $[M+H]^+$ 291.0336, found 291.0330.

3.33. N-(4-cyano-2-fluorophenyl)-5-fluoro-2-hydroxybenzamide (B13)

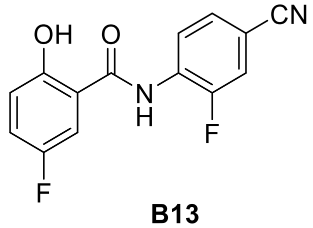

B13

The compound was purified by flash column chromatography on silica gel (Hexane/EA = 4/1, Rf = 0.45) as an off-white solid (yield: 60.1%). **^{1}H NMR** (400 MHz, DMSO-d_6) δ 7.06 (m, 1H), 7.36 (m, 1H), 7.72 (m, 2H), 7.96 (t, J = 5.6 Hz, 1H), 8.85 (d, J = 8.82 Hz, 1H), 11.09 (s, 1H), 12.04 (b, 1H). **^{13} C NMR** (100 MHz, DMSO-d_6) δ 106.00, 116.00, 118.66, 118.89, 119.18, 121.12, 121.80, 129.89, 131.30, 150.20, 152.69, 154.26, 156.61, 162.84. **HRMS** (ESI$^+$) m/z Calcd for C14H8F2N2O2 $[M + H]^+$ 275.0632, found 275.0631.

3.34. N-(3,4-bis(trifluoromethyl)phenyl)-4,5-difluoro-2-hydroxybenzamide (B14)

The compound was purified by flash column chromatography on silica gel (Hexane/EA = 4/1, Rf = 0.38) as an off-white solid (yield: 61.2%). ^{1}**H NMR** (400 MHz, DMSO-d_6) δ 7.01 (q, *J* = 8.0 Hz, 1H), 7.88 (t, *J* = 8.0 Hz, 1H), 8.02 (d, *J* = 8.0 Hz, 1H), 8.18 (d, *J* = 8.0 Hz, 1H), 8.41 (s, 1H), 10.79 (s, 1H), 11.67 (b, 1H). ^{13}**C NMR** (100 MHz, DMSO-d_6) δ 106.13, 115.35, 117.87, 119.43, 121.74, 123.58, 124.46, 129.89, 141.89, 142.77, 144.26, 151.18, 153.88, 155.13, 165.27. **HRMS** (ESI$^+$) m/z Calcd for C15H7F8NO2 [M+H]$^+$ 386.0427, found 386.0414.

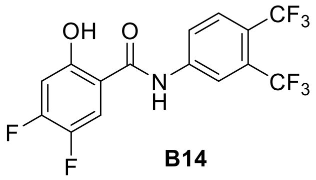

B14

3.35. N-(3,4-bis(trifluoromethyl)phenyl)-5-cyano-2-hydroxybenzamide (B15)

The compound was purified by flash column chromatography on silica gel (Hexane/EA = 4/1, Rf = 0.38) as an off-white solid (yield: 61.2%). ^{1}**H NMR** (300 MHz, DMSO-d_6) δ 7.16 (d, *J* = 9.0 Hz, 1H), 7.89 (dd, *J* = 9.0 Hz, 3.0 Hz, 1H), 8.12 (d, *J* = 9.0 Hz, 1H), 8.23 (m, 2H), 8.49 (s, 1H), 11.17 (s, 1H). ^{13}**C NMR** (75 MHz, DMSO-d_6) δ105.34, 115.40, 116.10, 117.89, 119.10, 120.50, 123.00 (×2), 125.20, 125.50, 126.10, 133.20, 139.50, 140.00, 159.98, 164.67. **HRMS** (ESI$^+$) m/z Calcd for C16H7F6N2O2 [M + H]$^+$ 374.0490, found 374.0475.

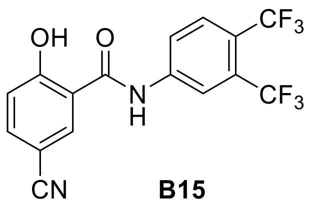

B15

3.36. N-(3,4-bis(trifluoromethyl)phenyl)-2-hydroxy-5-(trifluoromethyl)benzamide (B16)

The compound was purified by flash column chromatography on silica gel (Hexane/EA = 4/1, Rf = 0.41) as an off-white solid (yield: 51.1%). ^{1}**H NMR** (300 MHz, DMSO-d_6) δ 7.23 (d, *J* = 9.0 Hz, 1H), 7.82 (dd, *J* = 9.0 Hz, 3.0 Hz, 1H), 8.10 (d, *J* = 9.0 Hz, 1H), 8.13 (d, *J* = 3.0 Hz, 1H), 8.27 (d, *J* = 9.0 Hz, 1H), 8.48 (s, 1H), 10.99 (s, 1H). ^{13}**C NMR** (75 MHz, DMSO-d_6) δ116.10, 117.89, 119.10, 120.50, 123.30 (×2), 124.00, 125.00 (×2), 125.20, 125.50, 135.50, 139.98, 159.98, 162.20, 164.67. **HRMS** (ESI$^+$) m/z Calcd for C16H8F9NO2 [M+H]$^+$ 417.0411, found 417.0418.

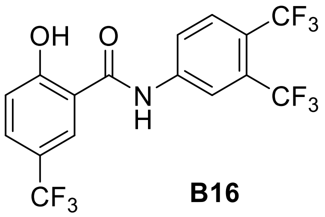

B16

3.37. N-(3,4-bis(trifluoromethyl)phenyl)-3-chlorobenzamide (C1)

The compound was purified by flash column chromatography on silica gel (Hexane/EA = 4/1, Rf = 0.31) as a white solid (yield: 90.5%). **^{1}H NMR** (400 MHz, $CDCl_3$) δ 7.08 (m, 1H), 7.31 (d, *J* = 4.0 Hz, 1H), 7.41 (b, 1H), 7.59 (b, 1H), 7.70 (s, 1H), 7.69-7.94 (m, 3H). **^{13}C NMR** (100 MHz, DMSO-d_6) δ 119.32, 121.81, 122.05, 123.26, 124.53, 124.76, 127.15, 128.00, 129.95, 130.99, 132.51, 133.75, 136.28, 143.51, 165.28. **HRMS** (ESI^+) m/z Calcd for C15H9ClF6NO $[M+H]^+$ 368.0276, found 368.0289.

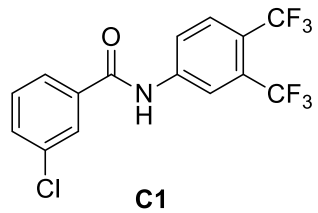

C1

3.38. 3-Chloro-N-(2-chloro-4-nitrophenyl)benzamide (C2)

The compound was purified by flash column chromatography on silica gel (Hexane/EA = 4/1, Rf = 0.31) as a white solid (yield: 90.5%). **^{1}H NMR** (400 MHz, DMSO-d_6) δ 7.59 (t, *J* = 8.0 Hz, 1H), 7.71 (d, *J* = 8.0 Hz, 1H), 7.93 (d, *J* = 8.0 Hz, 1H), 7.98 (d, *J* = 8.0 Hz, 1H), 8.02 (s, 1H), 8.25 (dd, *J* = 8.0 Hz, 4.0 Hz, 1H), 8.40 (d, *J* = 4.0 Hz, 1H), 10.48 (s, 1H). **^{13}C NMR** (100 MHz, DMSO-d_6) δ 123.34, 125.39, 127.18, 127.74, 128.15, 129.00, 131.08, 132.60, 133.81, 135.83, 141.49, 145.28, 164.71. **HRMS** (ESI^+) m/z Calcd for C13H9Cl2N2O3 $[M+H]^+$ 310.9990, found 310.9999.

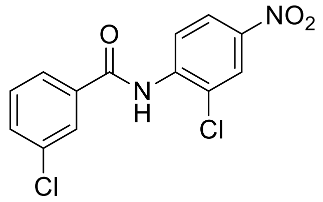

C2

3.39. 3-Chloro-N-(4-nitro-3-(trifluoromethyl)phenyl)benzamide (C3)

The compound was purified by flash column chromatography on silica gel (Hexane/EA = 4/1, Rf = 0.44) as a white solid (yield: 87.9%). **^{1}H NMR** (400 MHz, CDC3) δ 7.49 (t, *J* = 8.0 Hz, 1H), 7.59 (d, *J* = 4.0 Hz, 1H), 7.77 (d, *J* = 8.0 Hz, 1H), 7.88 (s, 1H), 8.04 (d, *J* = 8.0 Hz, 1H), 8.09 (s, 1H), 8.15 (d, 2H). **^{13}C NMR** (100 MHz, DMSO-d_6) δ 118.90, 121.15, 123.00, 123.33, 123.76, 123.87, 127.22, 131.06, 132.66, 133.79, 136.13, 142.18, 144.08, 165.37. **HRMS** (ESI^+) m/z Calcd for C14H9ClF3N2O3 $[M+H]^+$ 345.0254, found 345.0265.

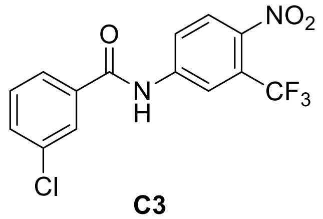

C3

3.40. 3-Bromo-N-(2-chloro-4-nitrophenyl)benzamide (C4)

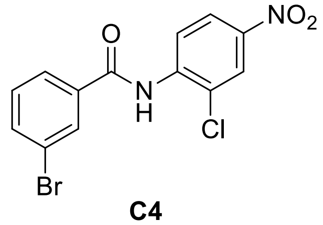

C4

The compound was purified by flash column chromatography on silica gel (Hexane/EA = 4/1, Rf = 0.34) as a colorless solid (yield: 67.2%). **^{1}H NMR** (400 MHz, DMSO-d_6) δ 7.52 (t, J = 8.0 Hz, 1H), 7.84 (d, J = 8.0 Hz, 1H), 7.97 (d, J = 12.0 Hz, 2H), 8.16 (s, 1H), 8.25 (dd, J = 8.0 Hz, 4.0 Hz, 1H), 8.40 (d, J = 4.0 Hz, 1H), 10.48 (s, 1H). **^{13}C NMR** (100 MHz, DMSO-d_6) δ 108.36, 116.07, 119.33, 123.28, 127.82, 131.97, 132.04, 133.58, 145.85, 154.71, 156.14, 157.29, 165.46. **HRMS** (ESI$^+$) m/z Calcd for C13H9BrClN2O3 [M+H]$^+$ 354.9485, found 354.9495.

3.41. 3-Bromo-N-(4-nitro-3-(trifluoromethyl)phenyl)benzamide (C5)

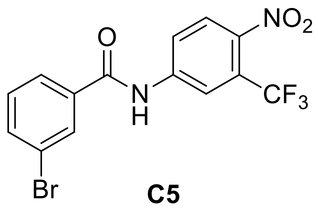

C5

The compound was purified by flash column chromatography on silica gel (Hexane/EA = 4/1, Rf = 0.24) as a colorless solid (yield: 74.9%). **^{1}H NMR** (400 MHz, $CDCl_3$) δ 7.42 (t, J = 8.0 Hz, 1H), 7.75 (d, J = 8.0 Hz, 1H), 7.82 (d, J = 8.0 Hz, 1H), 8.04 (d, 2H), 8.09 (d, J = 8.0 Hz, 1H), 8.14 (b, 2H). **^{13}C NMR** (100 MHz, DMSO-d_6) δ 118.88, 118.94, 121.15, 122.23, 123.00, 123.33, 123.77, 127.60, 130.87, 135.55, 136.32, 142.18, 144.08, 165.28. **HRMS** (ESI$^+$) m/z Calcd for C14H9BrF3N2O3 [M+H]$^+$ 388.9749, found 388.9758.

3.42. 5-Chloro-2-((2-chloro-4-nitrophenyl)carbamoyl)phenyl trifluoromethanesulfonate (C6)

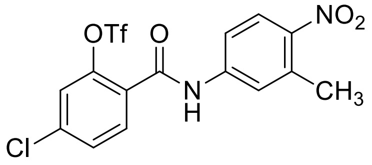

C6

The compound was purified by flash column chromatography on silica gel (Hexane/EA = 4/1, Rf = 0.24) as a white solid (yield: 90.3%). **^{1}H NMR** (400 MHz, $CDCl_3$) δ 2.66 (s, 3H), 7.42 (s, 1H), 7.55 (d, J = 8.0 Hz, 1H), 7.59 (d, J = 8.0 Hz, 1H), 7.66 (s, 1H), 7.90 (d, J = 12.0 Hz, 1H), 8.01 (b, 1H), 8.09 (d, J = 12.0 Hz, 1H). **^{13}C NMR** (100 MHz, DMSO-d_6) δ 21.11, 118.17, 120.06, 122.90, 123.43, 126.80, 128.79, 129.76, 132.31, 135.33, 137.12, 143.35, 144.43, 146.50, 162.80. HRMS (ESI$^+$) m/z Calcd for C15H11ClF3N2O6S [M+H]$^+$ 438.9978, found

438.9990.

3.43. 4-Chloro-2-((2-chloro-4-nitrophenyl)carbamoyl)phenyl acetate (C7)

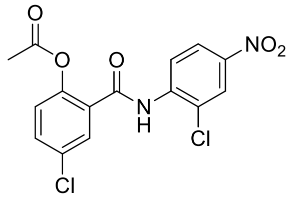

C7

The compound was purified by flash column chromatography on silica gel (Hexane/EA = 5/1, Rf = 0.31) as a white solid (yield: 89.3%). **^{1}H NMR** (400 MHz, $CDCl_3$) δ 2.39 (s, 3H), 7.18 (d, *J* = 8.0 Hz, 1H), 7.55 (d, *J* = 8.0 Hz, 1H), 7.97 (s, 1H), 8.24 (d, *J* = 12.0 Hz, 1H), 8.35 (s, 1H), 8.45 (d, *J* = 12.0 Hz, 1H), 9.05 (b, 1H). **^{13}C NMR** (100 MHz, $CDCl_3$) δ 21.31, 120.69, 122.33, 123.81, 124.81, 125.16, 128.04, 130.66, 132.44, 133.23, 140.11, 143.32, 146.35, 162.20, 168.31. **HRMS** (ESI^+) m/z Calcd for C15H11Cl2N2O5 $[M+H]^+$ 369.0045, found 369.0041.

3.44. 4-Chloro-2-((2-chloro-4-nitrophenyl)carbamoyl)phenyl trifluoromethanesulfonate (C8)

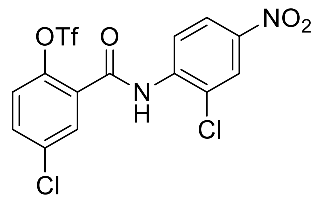

C8

The compound was purified by flash column chromatography on silica gel (Hexane/EA = 10/1, Rf = 0.30) as a white solid (yield: 97.7%). **^{1}H NMR** (400 MHz, $CDCl_3$) δ 7.40 (d, *J* = 12.0 Hz, 1H), 7.64 (d, *J* = 8.0 Hz, 1H), 7.91 (s, 1H), 8.25 (d, *J* = 8.0 Hz, 1H), 8.36 (s, 1H), 8.58 (b, 1H), 8.79 (d, *J* = 12.0 Hz, 1H). **^{13}C NMR** (100 MHz, $CDCl_3$) δ 109.89, 116.82, 120.02, 123.53, 124.85, 125.55, 126.68, 127.72, 130.90, 133.36, 133.59, 140.71, 145.20, 162.51. **HRMS** (ESI^+) m/z Calcd for C14H8Cl2F3N2O6S $[M+H]^+$ 458.9432, found 458.9446.

4. NMR Spectrum of Target Compounds

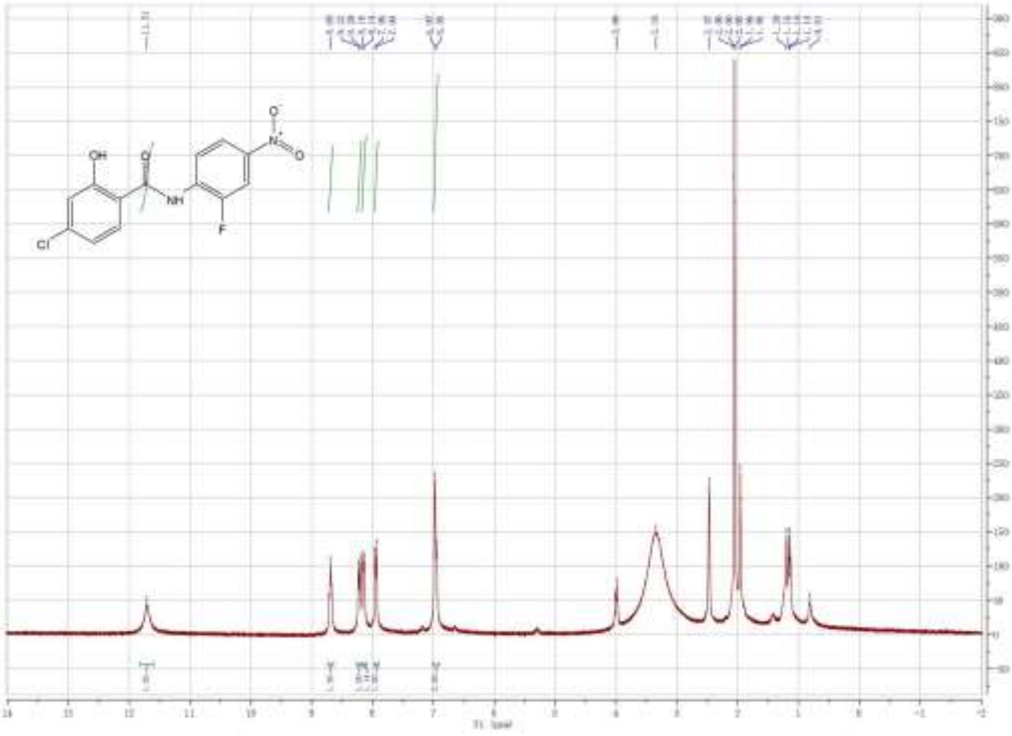

Figure 1． ¹H NMR spectrogram of Compound A1

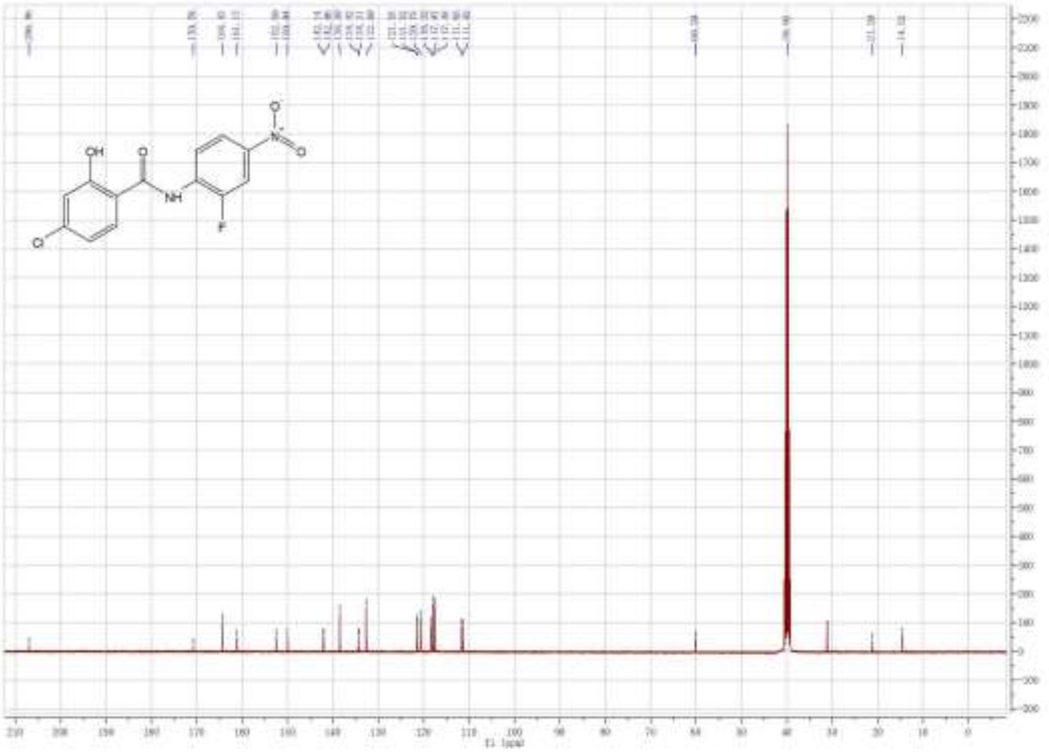

Figure 2. ¹³C NMR spectrogram of Compound A1

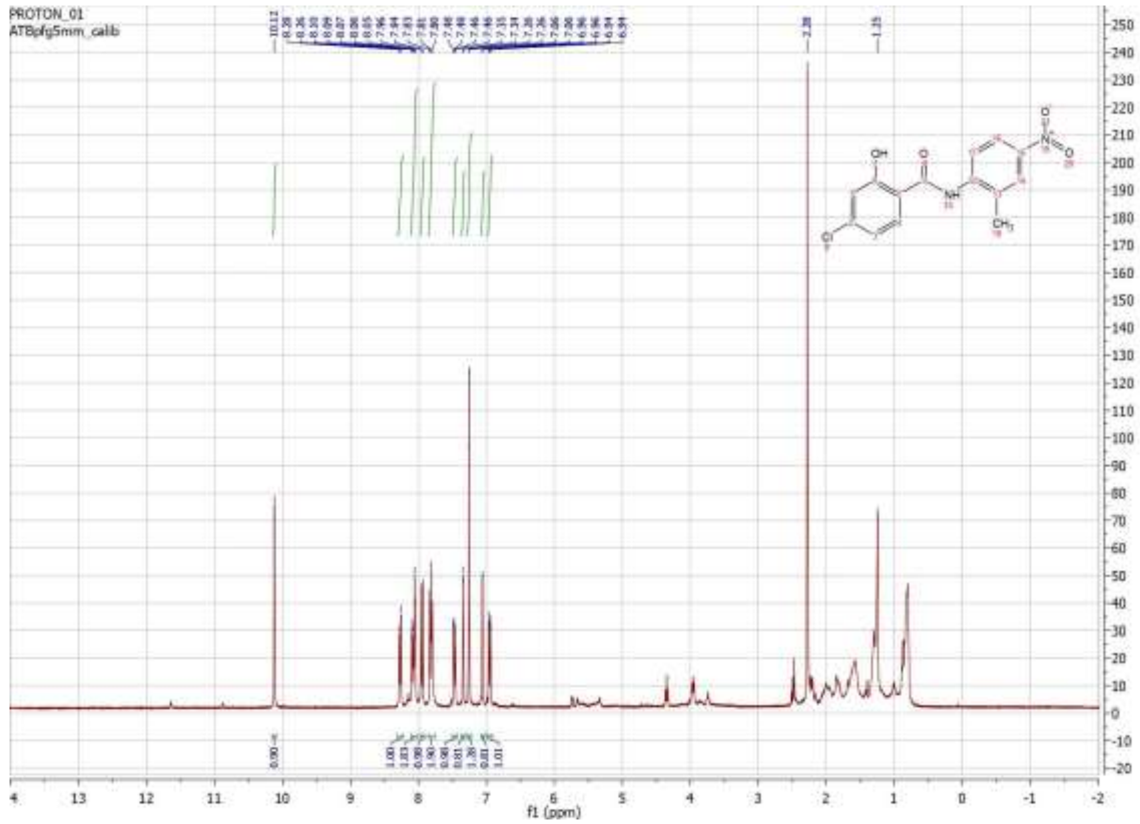

Figure 3. ^{1}H NMR spectrogram of Compound A2

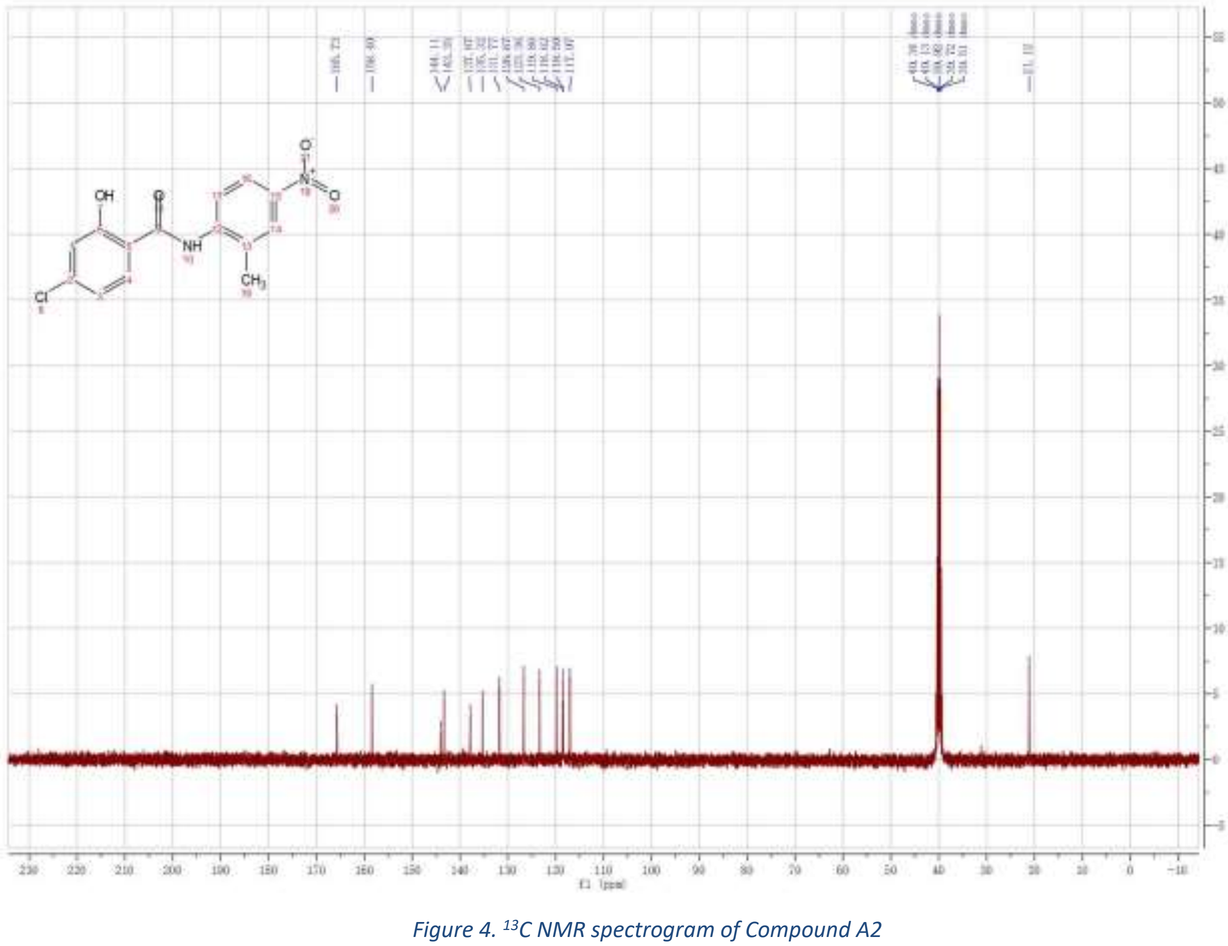

Figure 4. ^{13}C NMR spectrogram of Compound A2

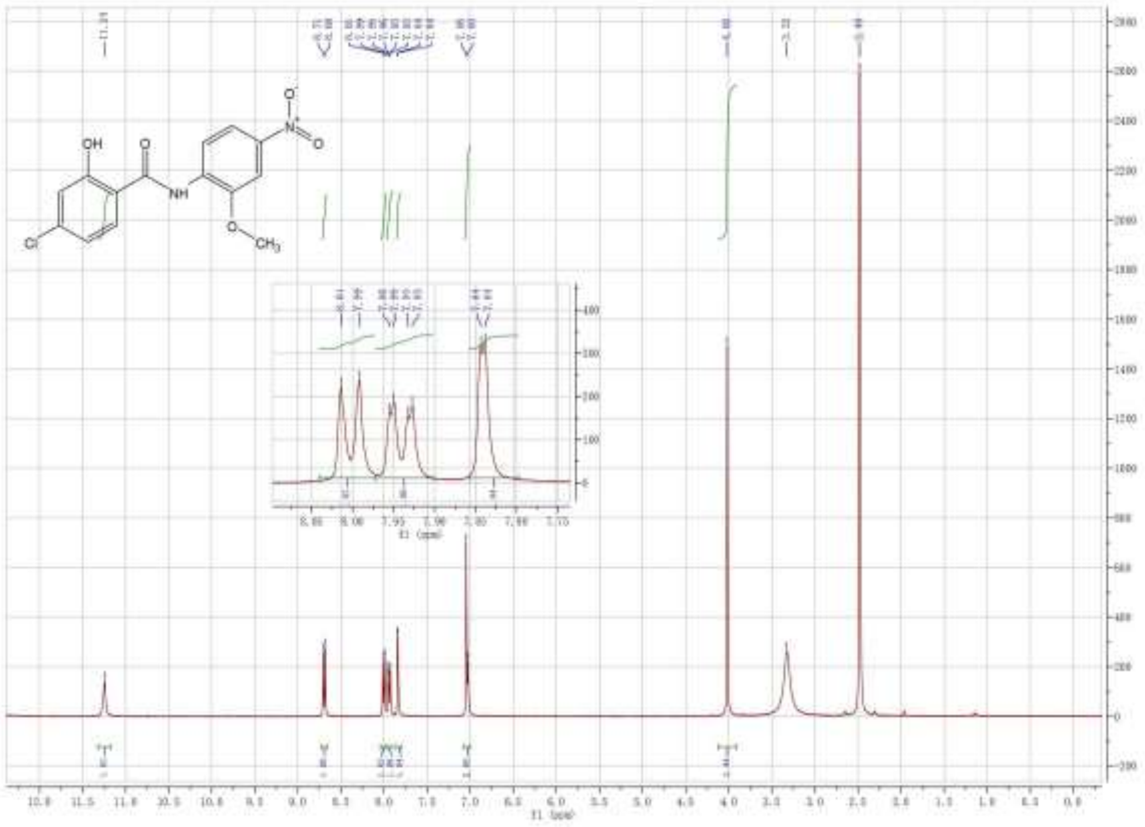

Figure 5. ^{1}H NMR spectrogram of Compound A3

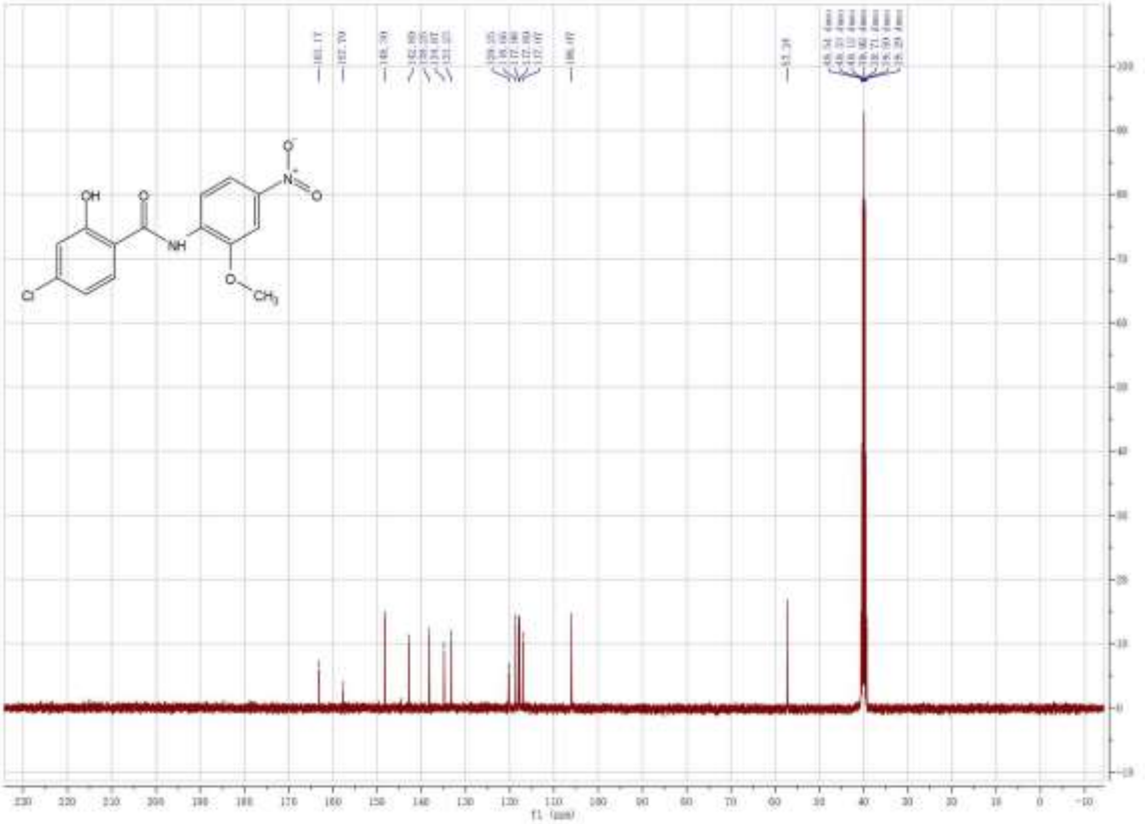

Figure 6. ^{13}C NMR spectrogram of Compound A3

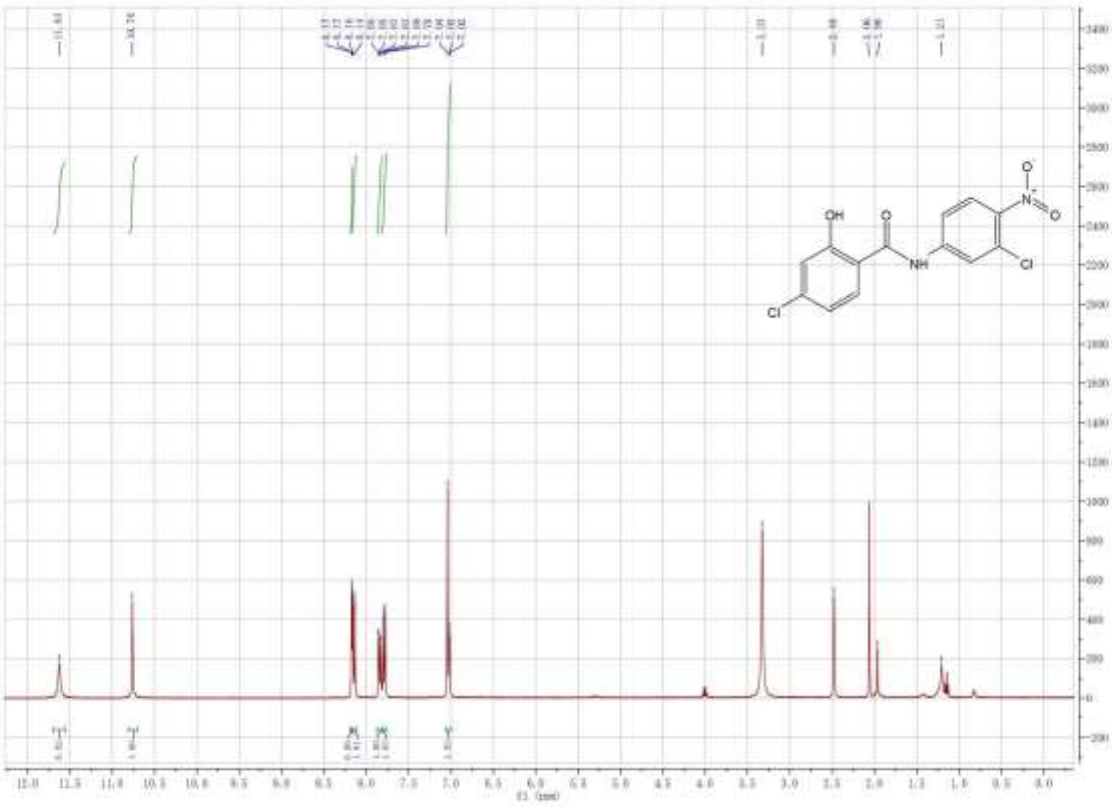

Figure 7. ¹H NMR spectrogram of Compound A4

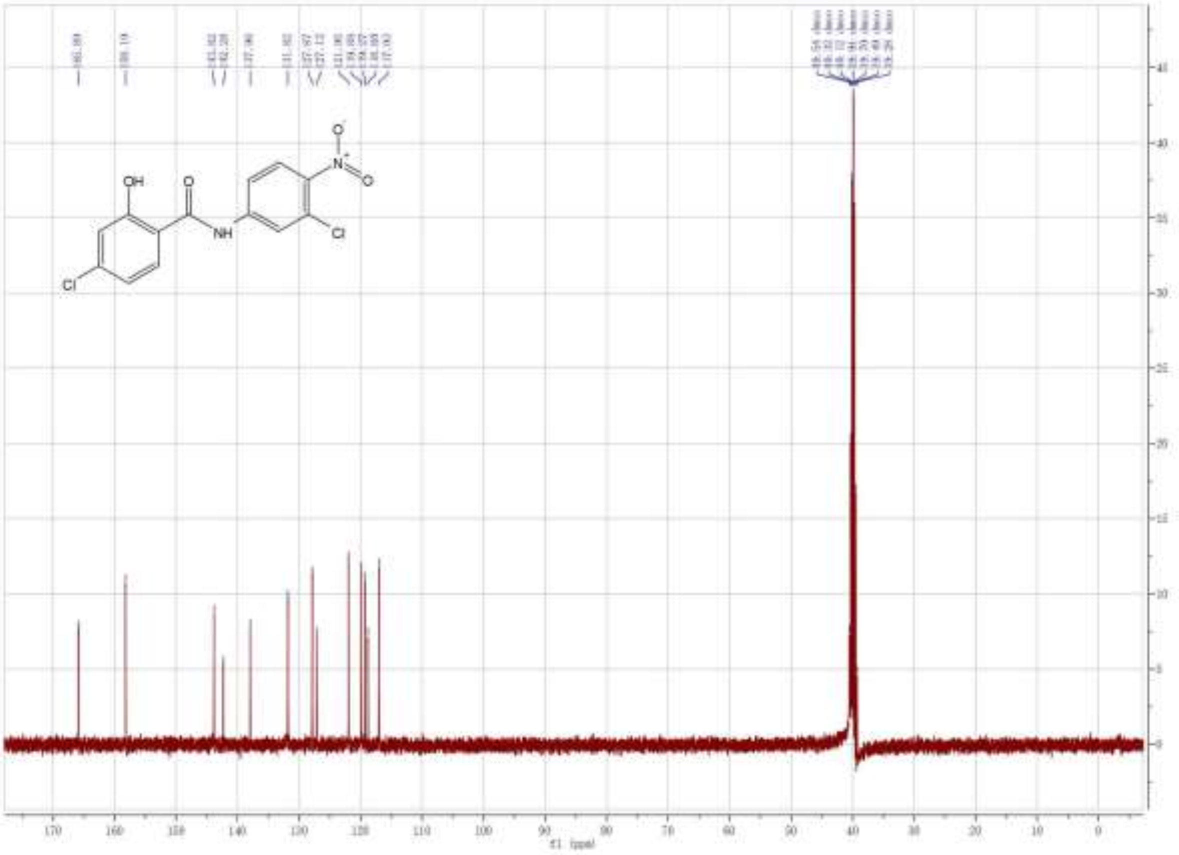

Figure 8. ¹³C NMR spectrogram of Compound A4

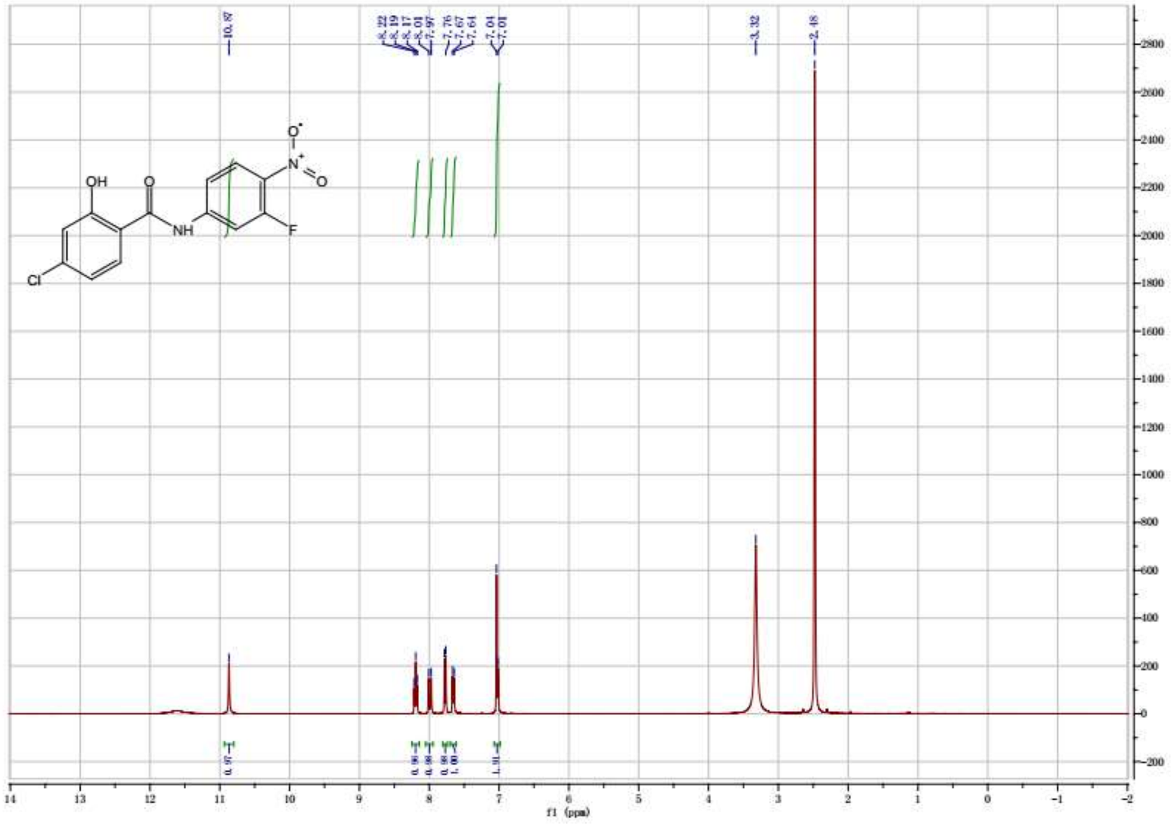

Figure 9. ^{1}H NMR spectrogram of Compound A5

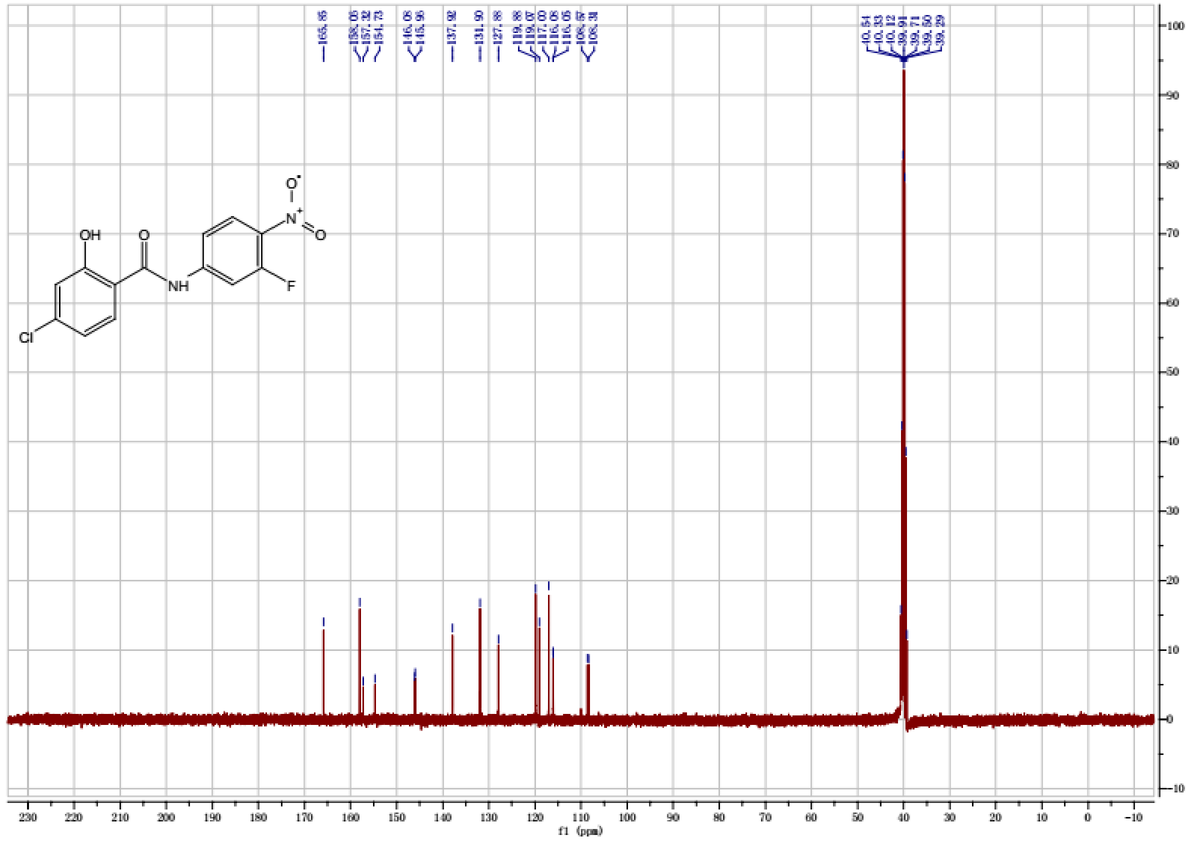

Figure 10. ^{13}C NMR spectrogram of Compound A5

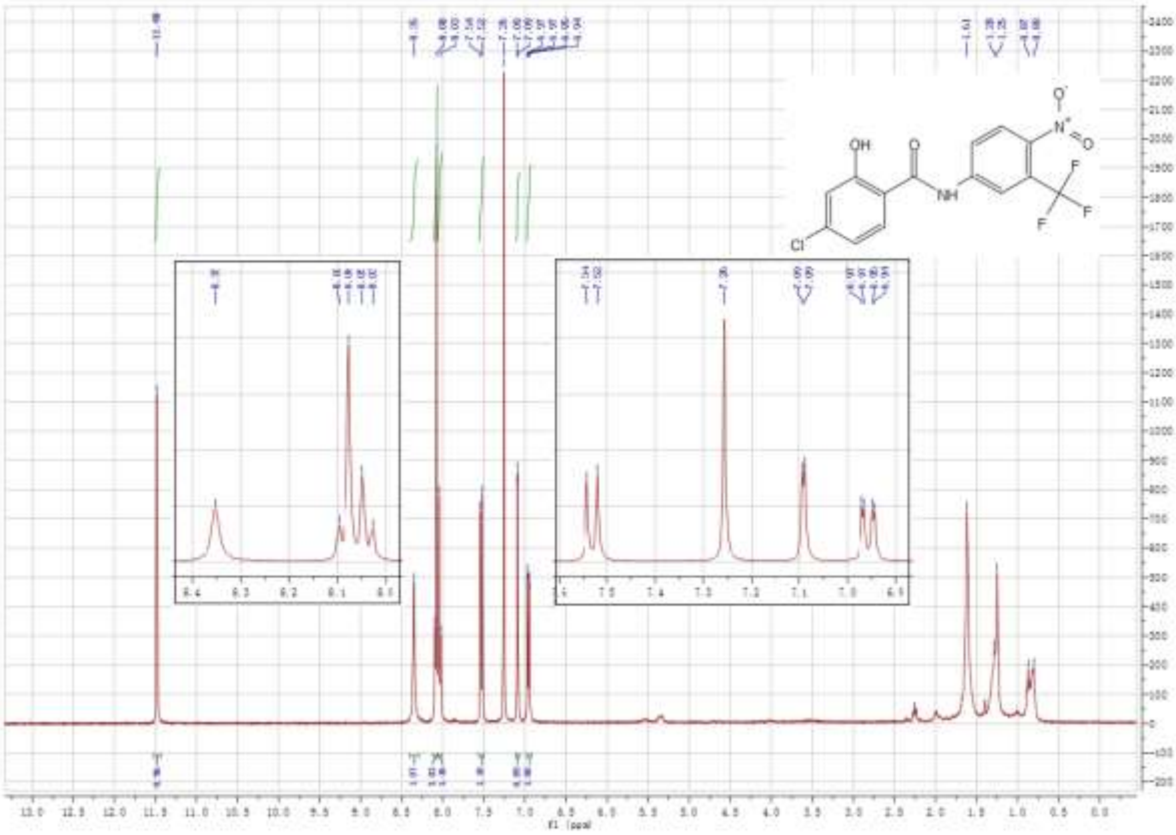

Figure 11. ¹H NMR spectrogram of Compound A6

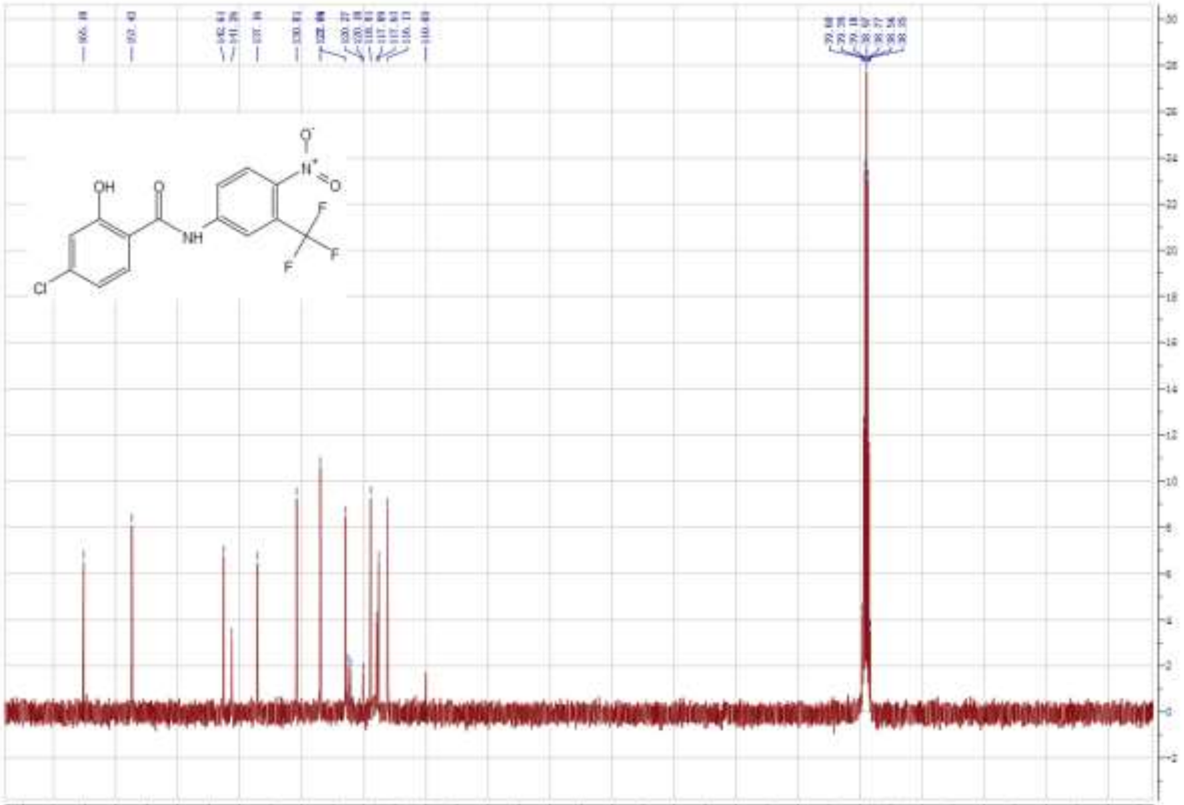

Figure 12. ¹³C NMR spectrogram of Compound A6

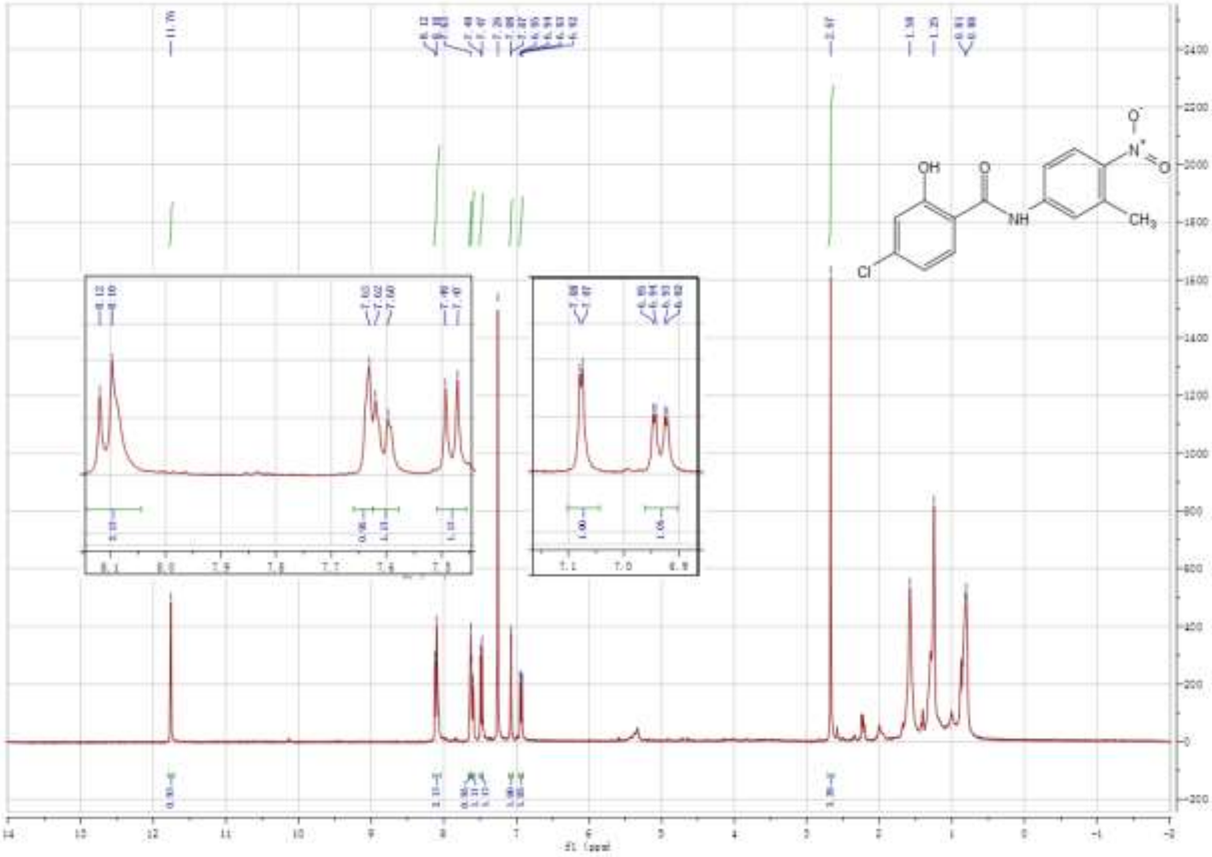

Figure 13. ^{1}H NMR spectrogram of Compound A7

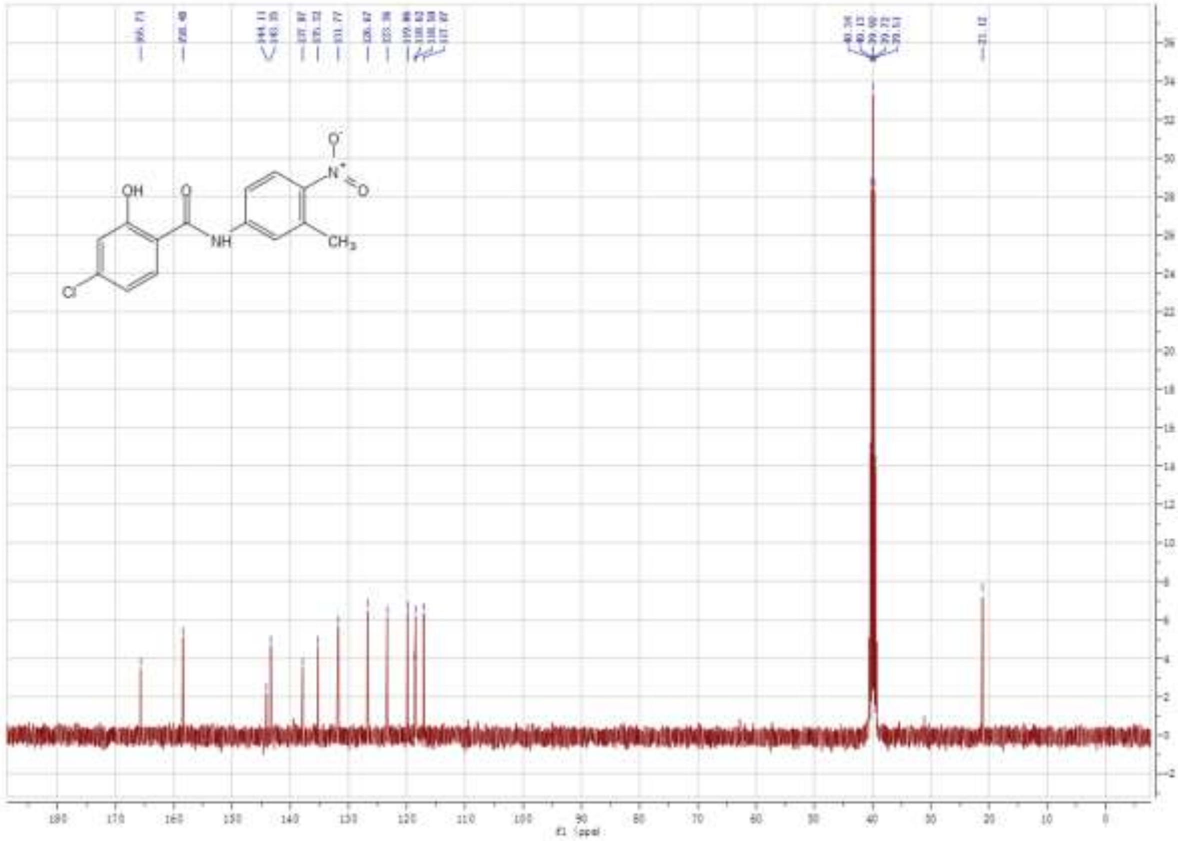

Figure 14. ^{13}C NMR spectrogram of Compound A7

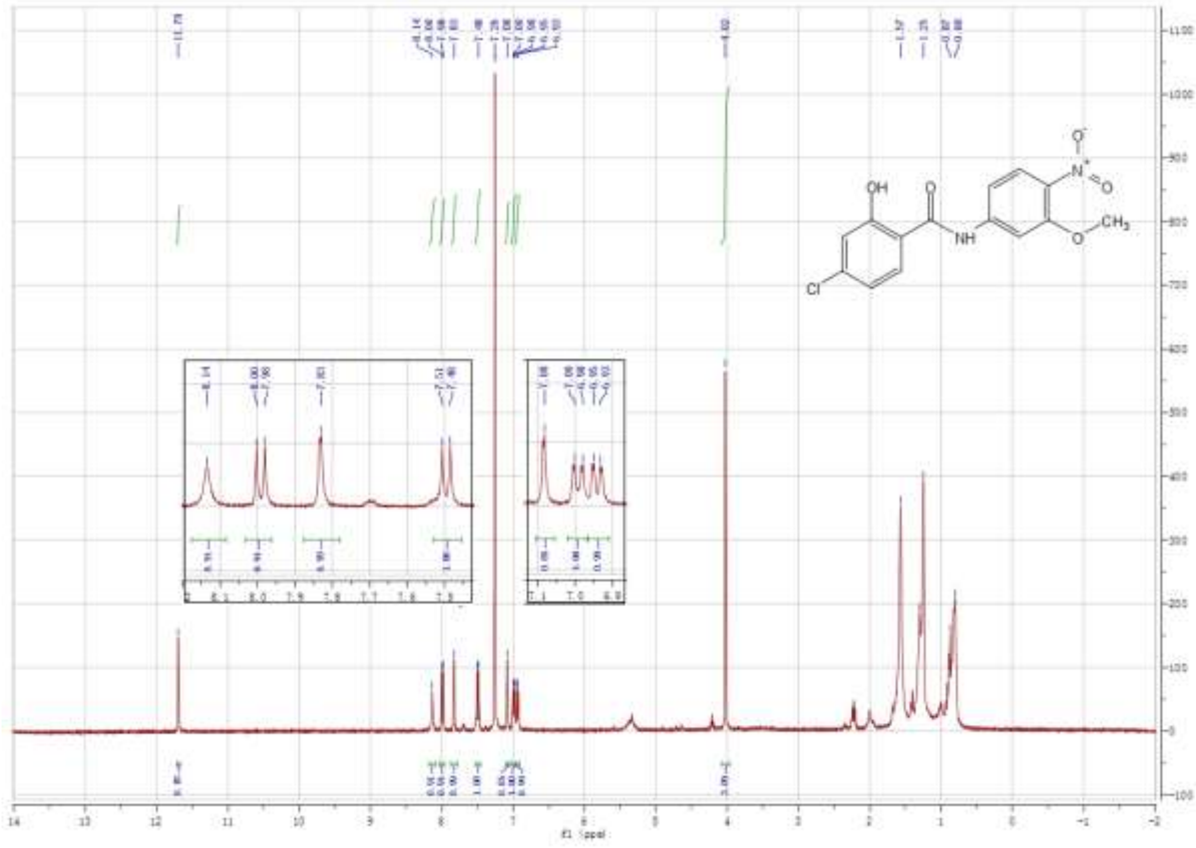

Figure 15. ¹H spectrogram of Compound A8

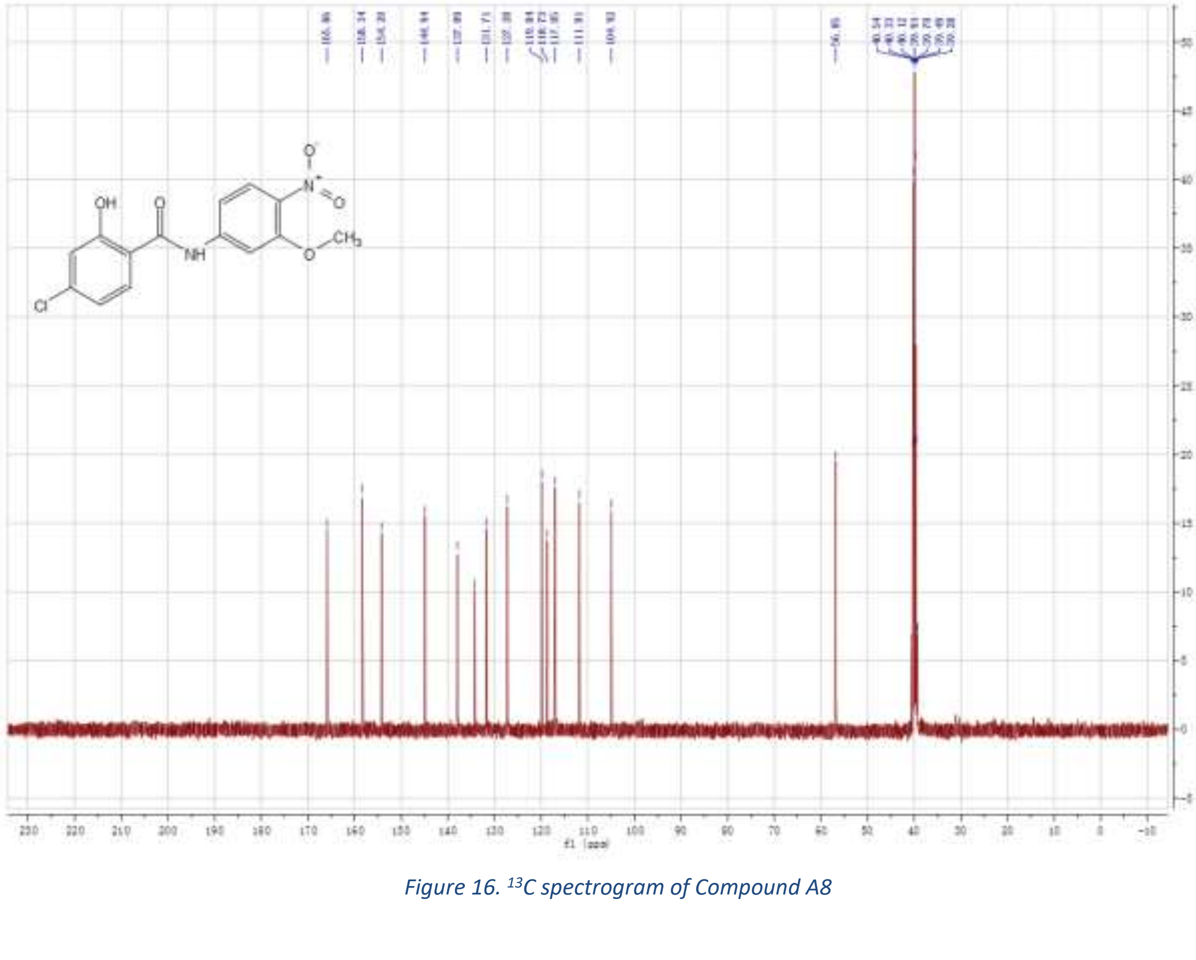

Figure 16. ¹³C spectrogram of Compound A8

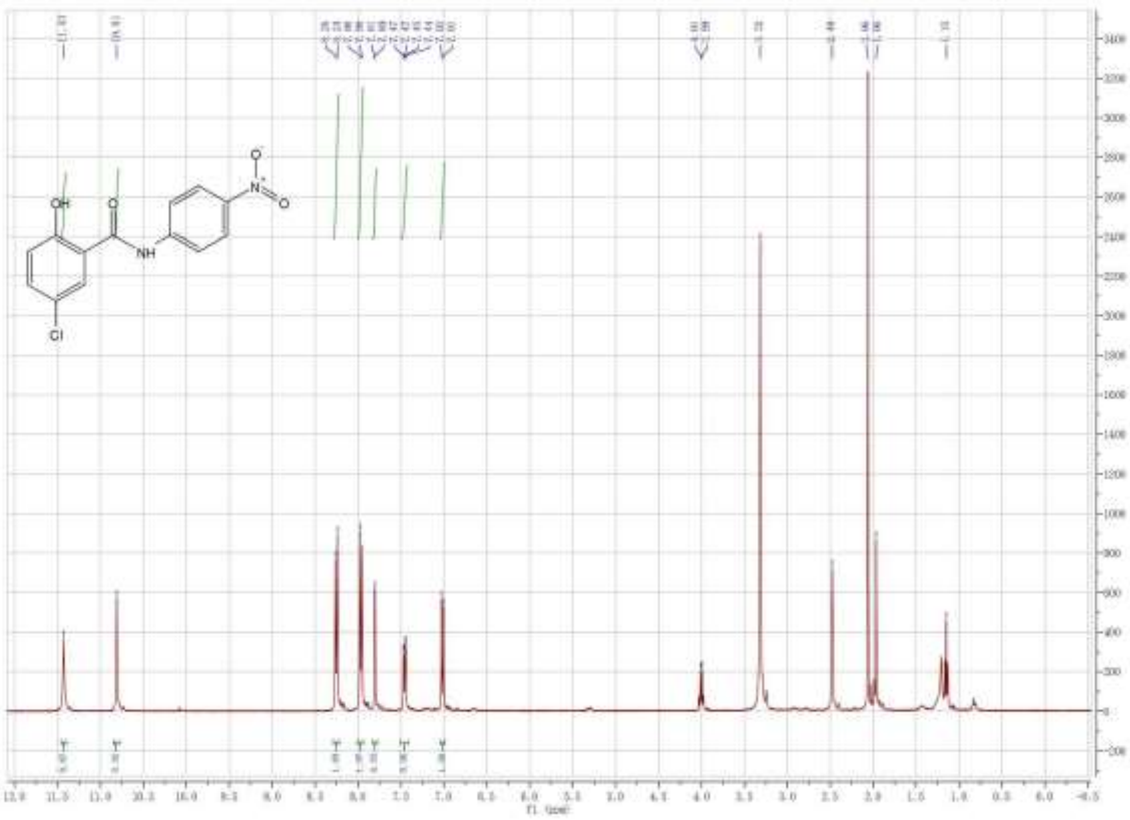

Figure 17. ^{1}H NMR spectrogram of Compound A9

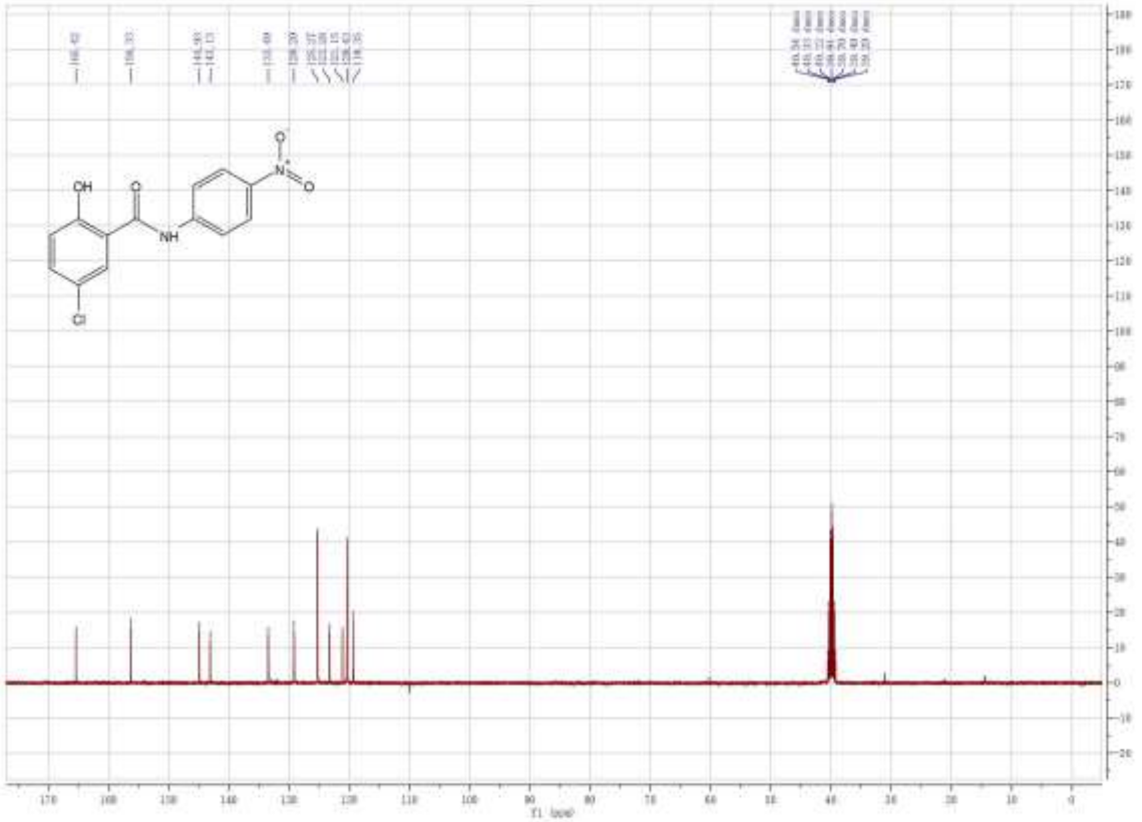

Figure 18. ^{13}C NMR spectrogram of Compound A9

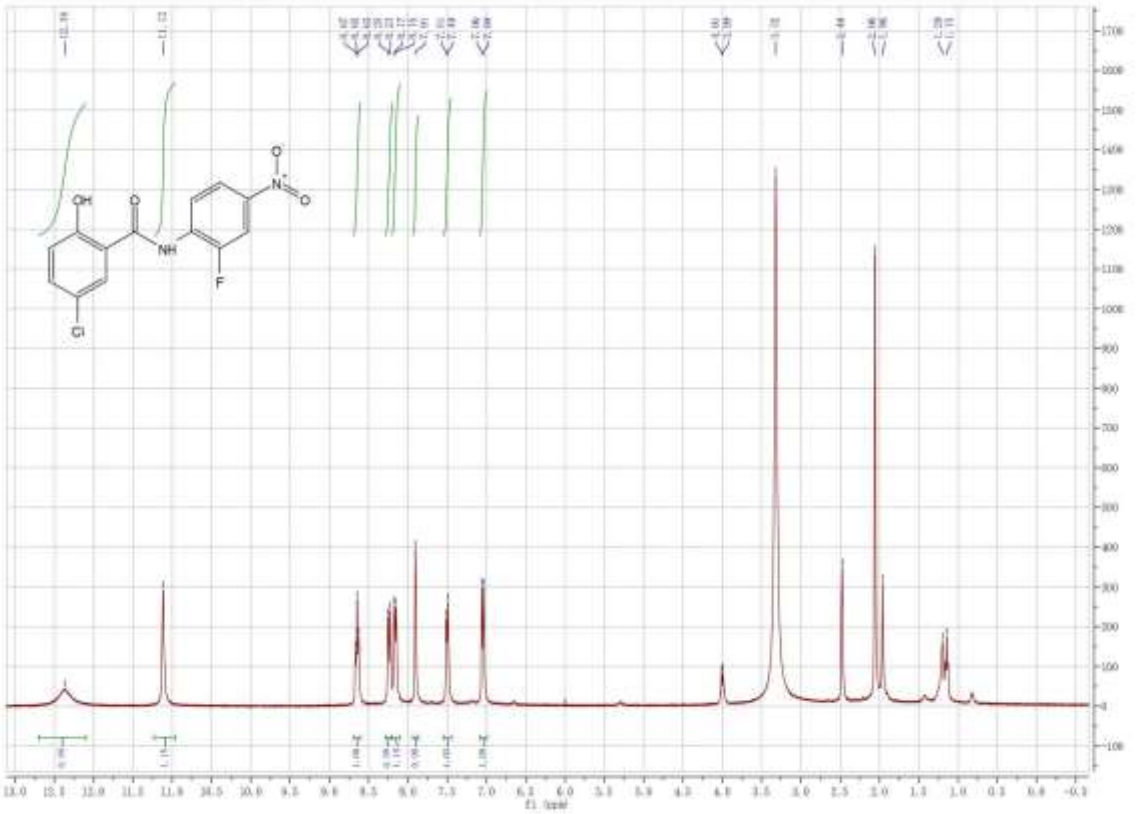

Figure 19. ¹H NMR spectrogram of Compound A10

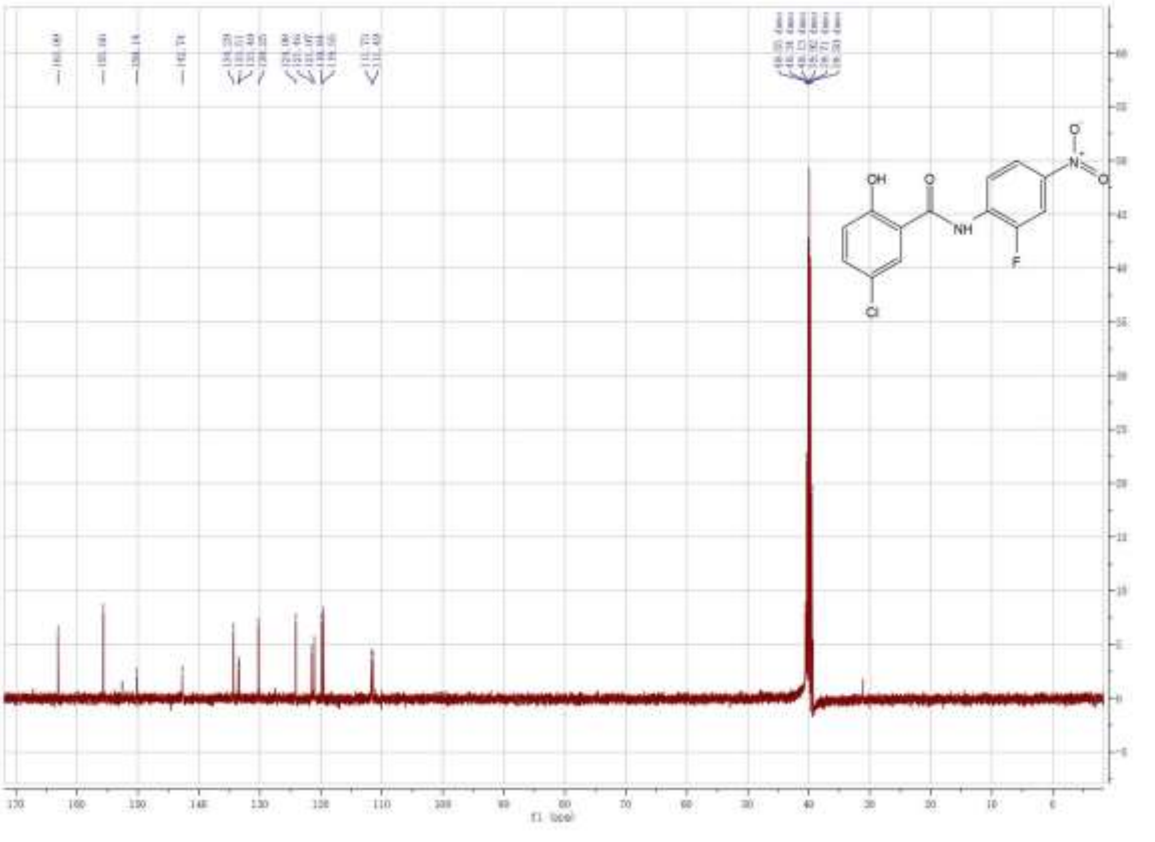

Figure 20. ¹³C NMR spectrogram of Compound A10

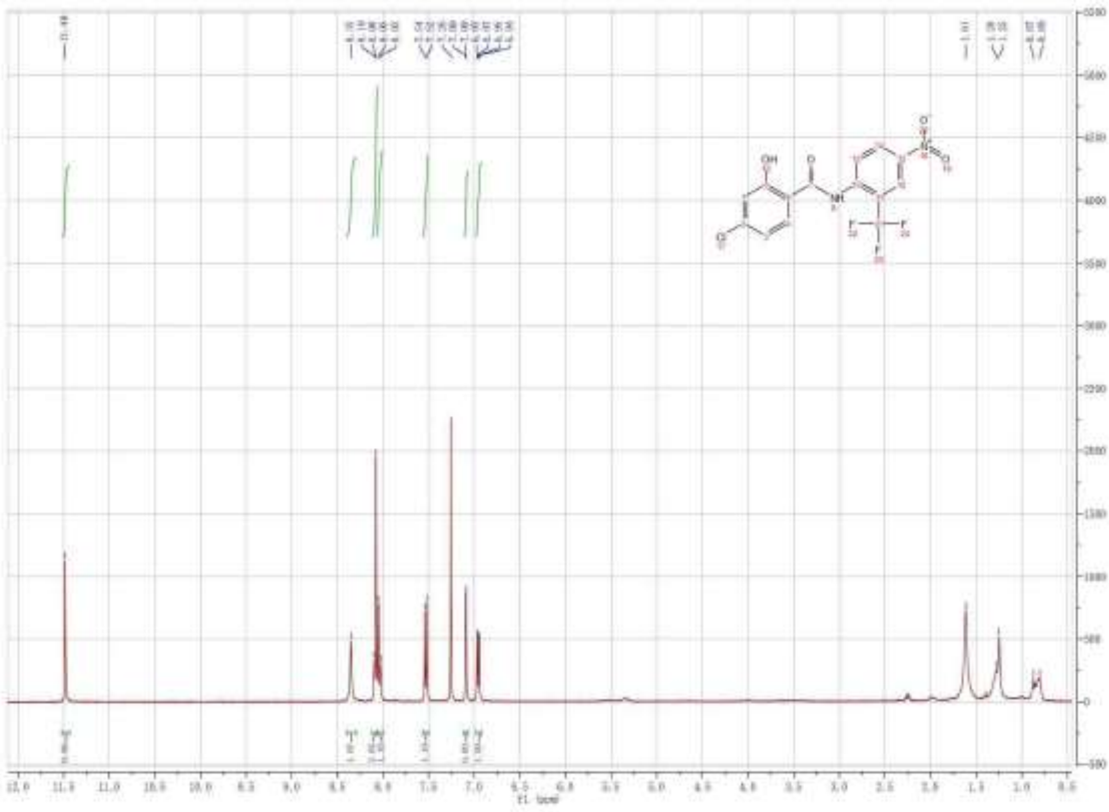

Figure 21. ¹H NMR spectrogram of Compound A11

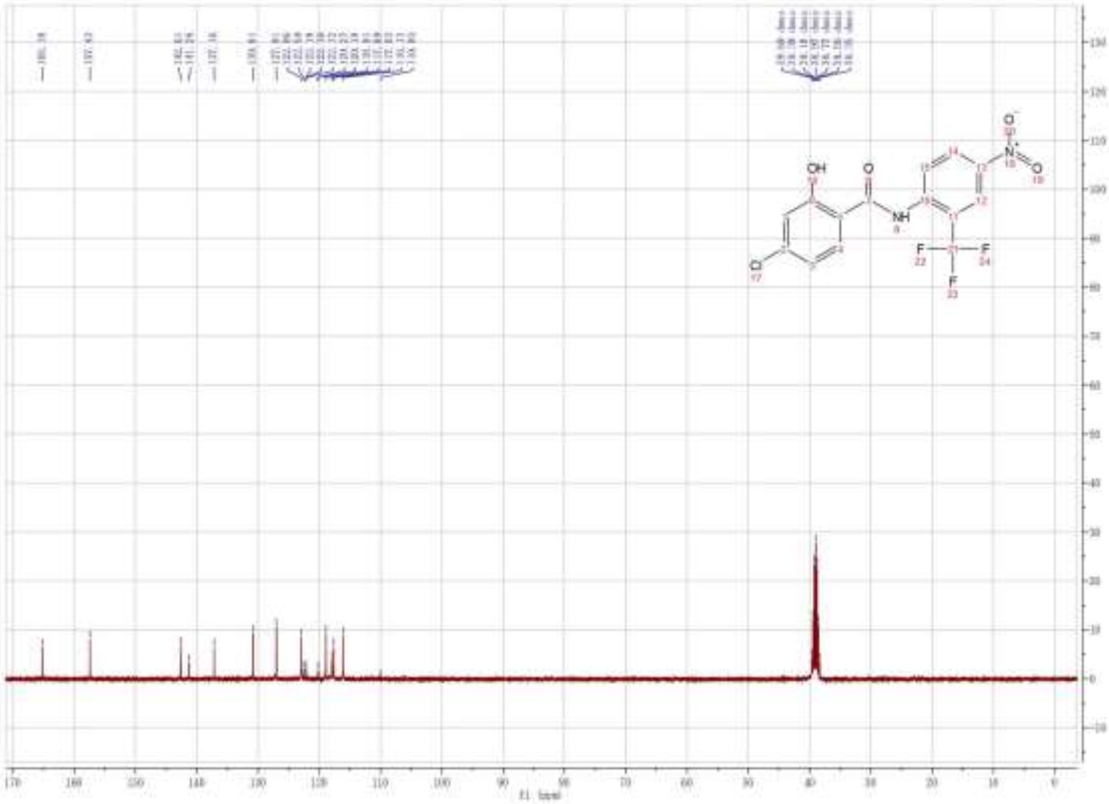

Figure 22. ¹³C NMR spectrogram of Compound A11

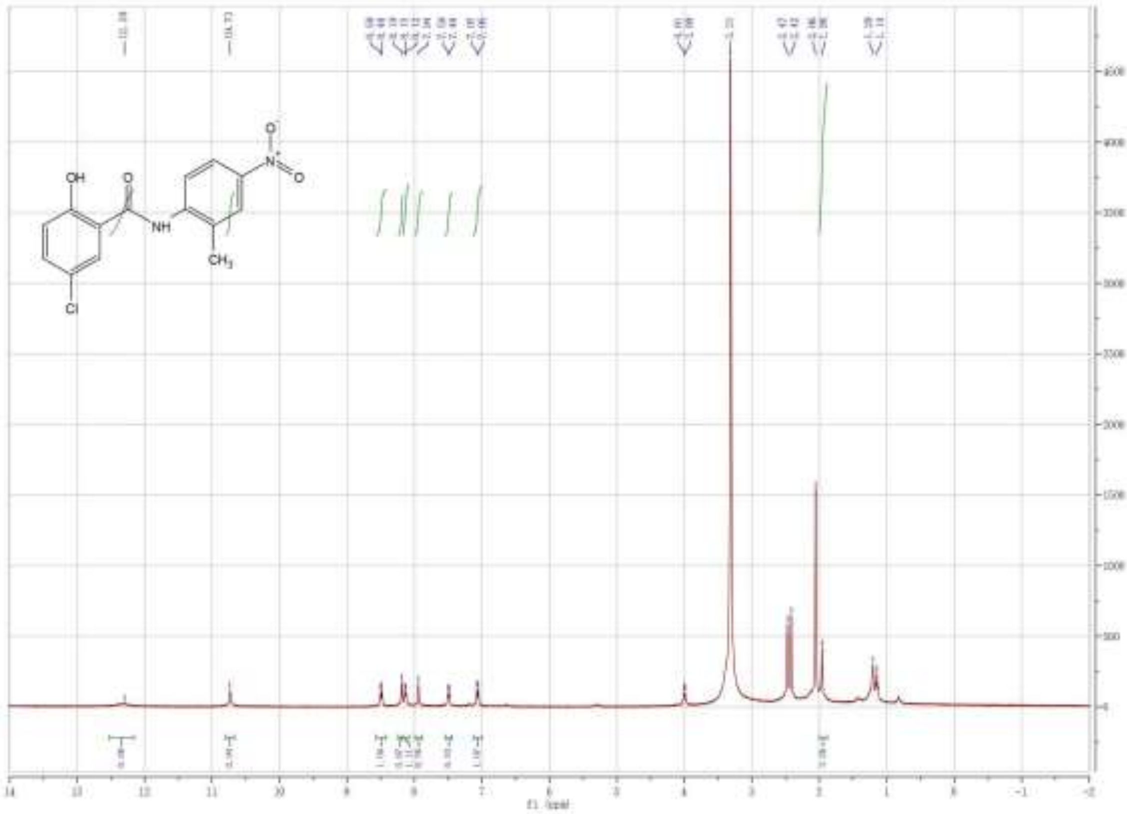

Figure 23. ^1H NMR spectrogram of Compound A12

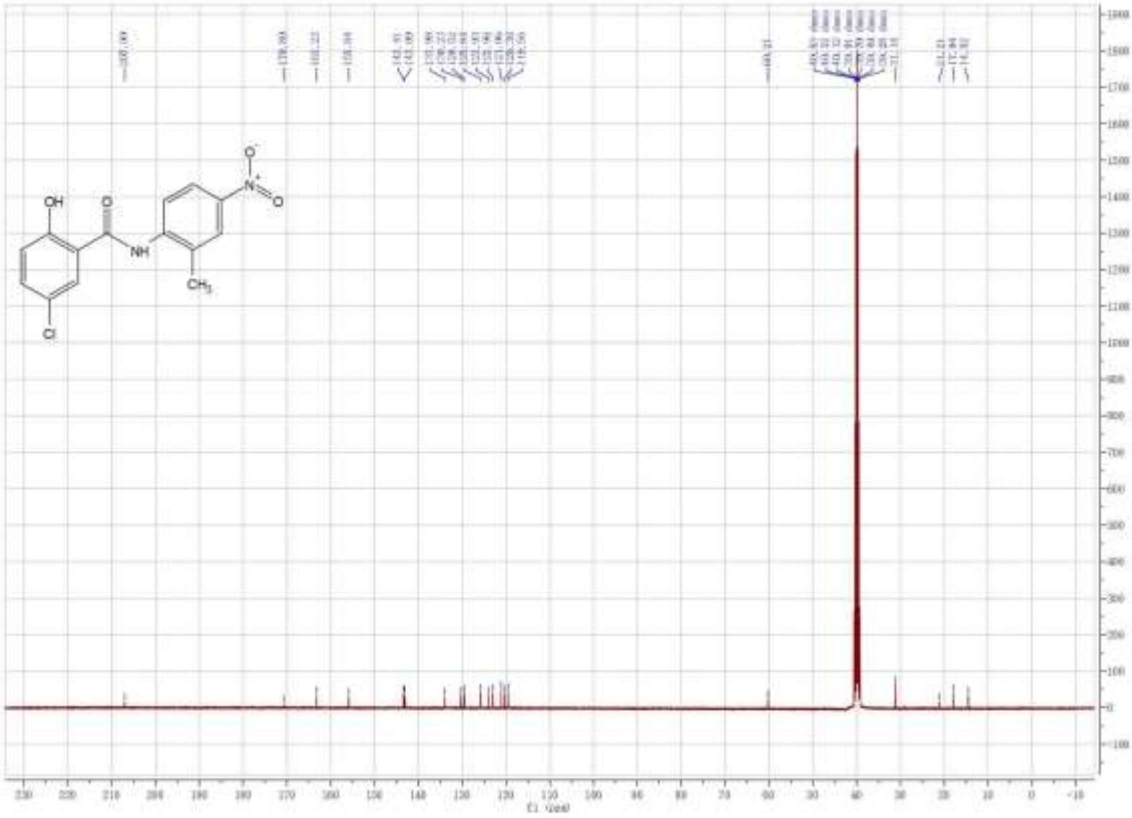

Figure 24. ^{13}C NMR spectrogram of Compound A12

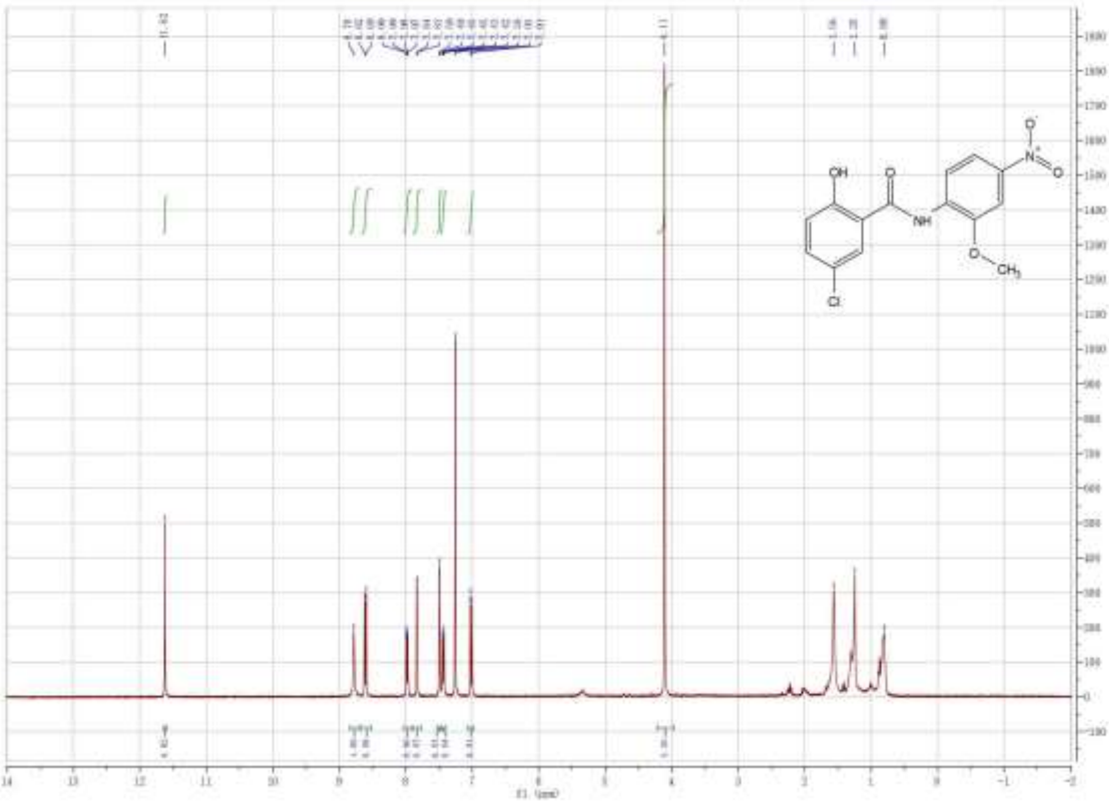

Figure 25. ¹H NMR spectrogram of Compound A13

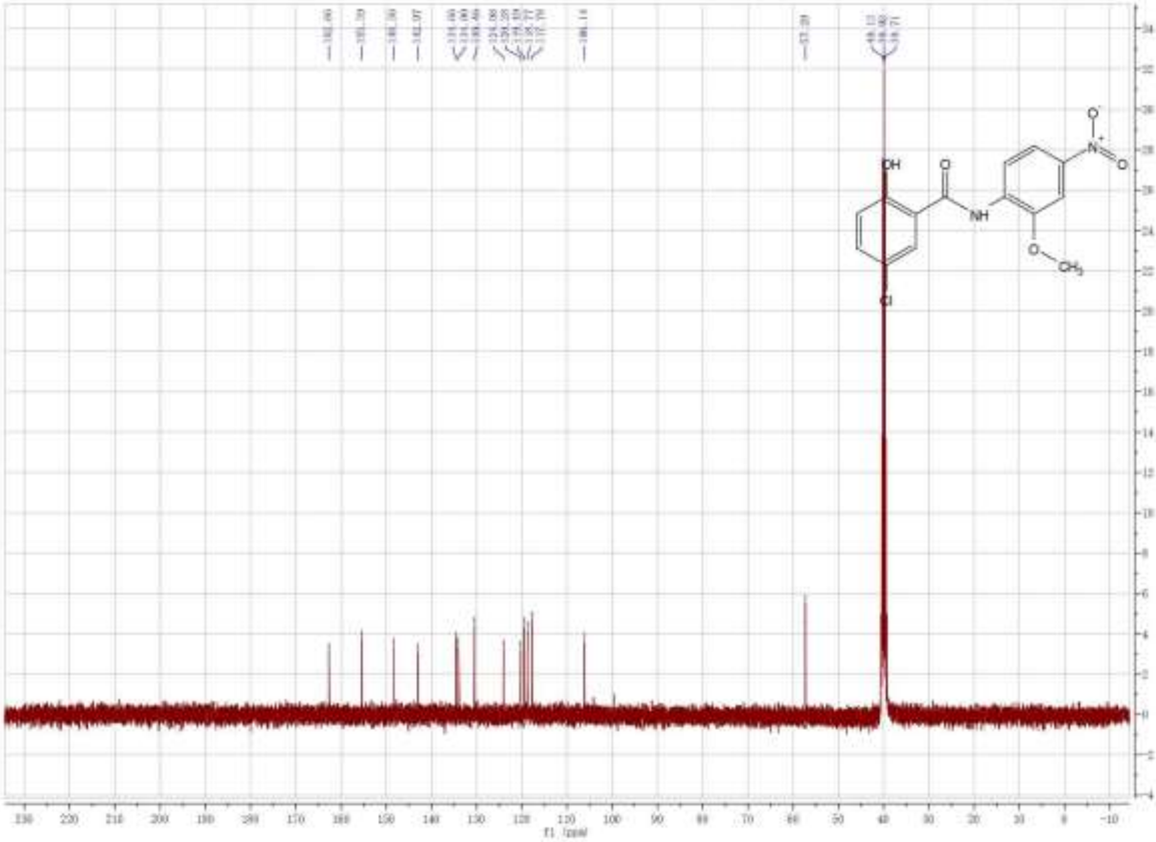

Figure 26. ¹³C NMR spectrogram of Compound A13

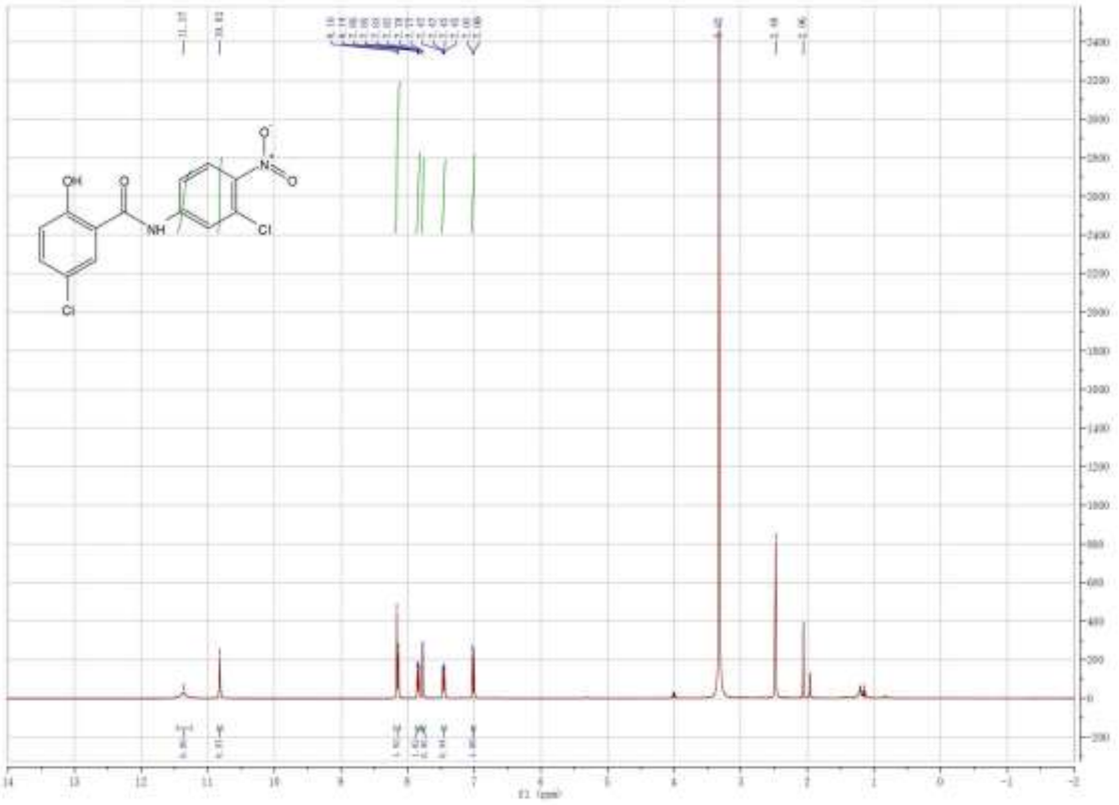

Figure 27. ^{1}H NMR spectrogram of compound A14

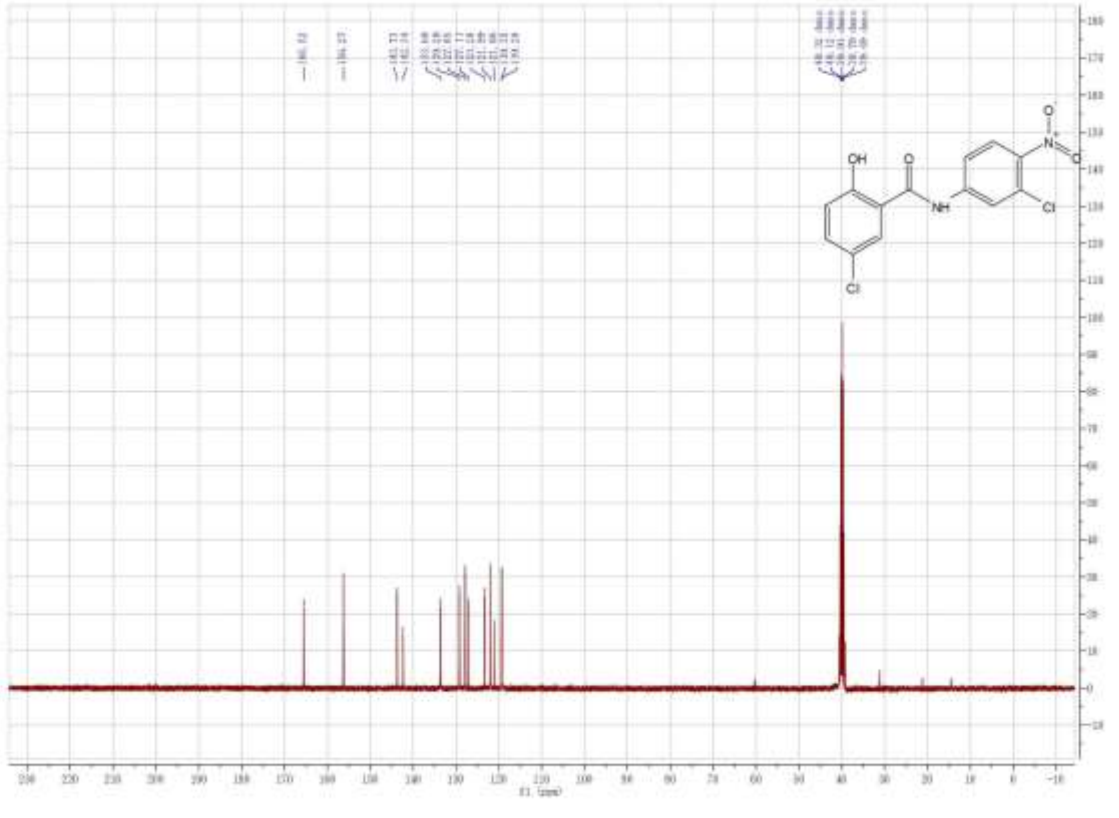

Figure 28. ^{13}C NMR spectrogram of compound A14

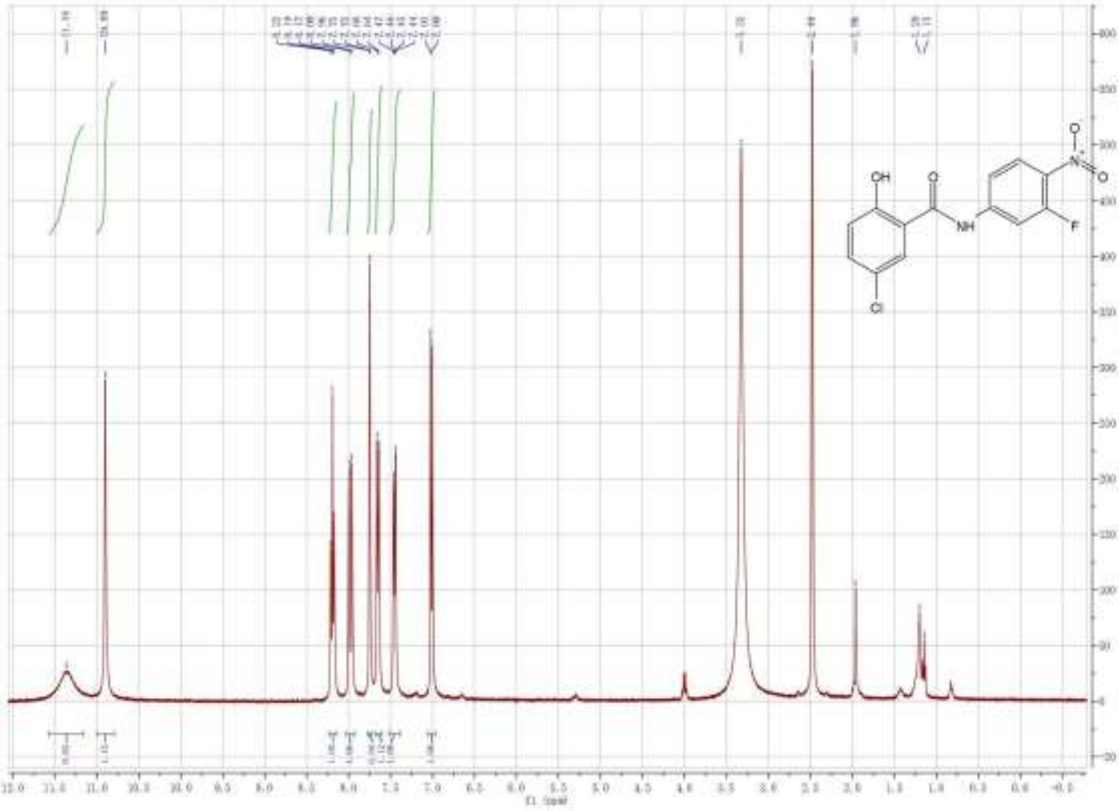

Figure 29. ^{1}H NMR spectrogram of Compound A15

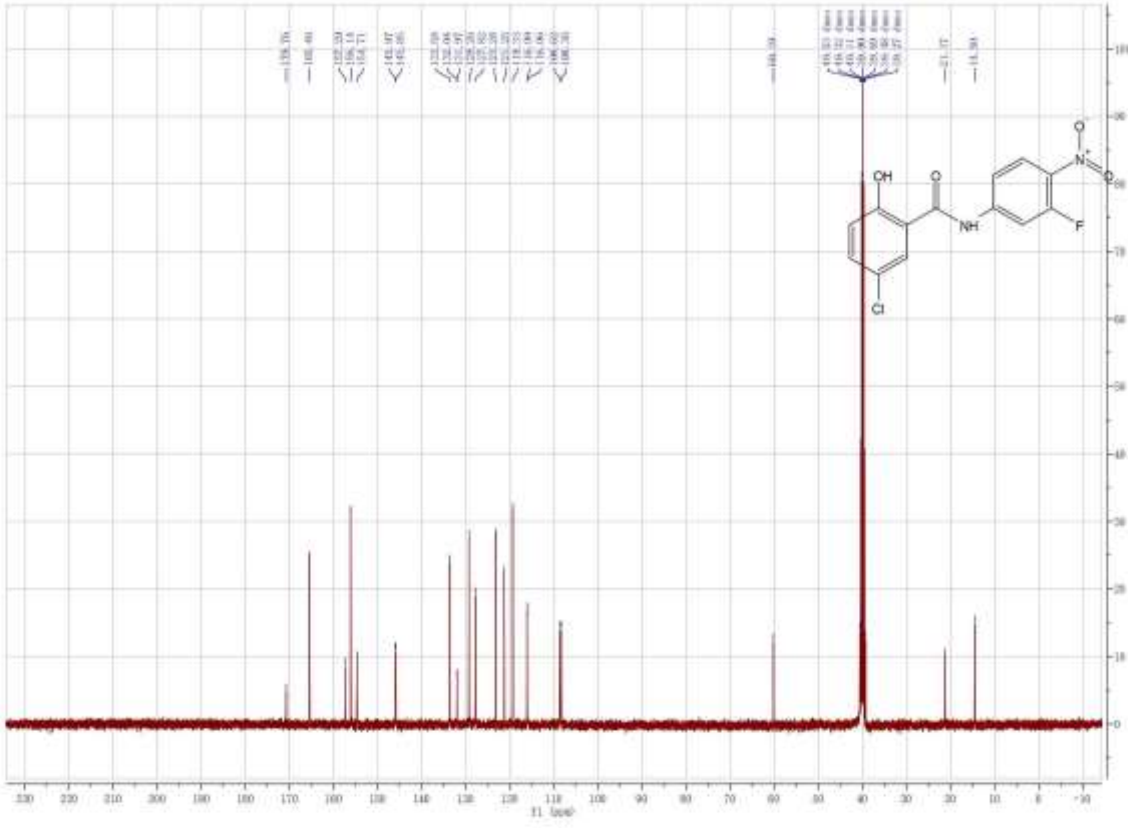

Figure 30. ^{13}C NMR specrtogram of Compound A15

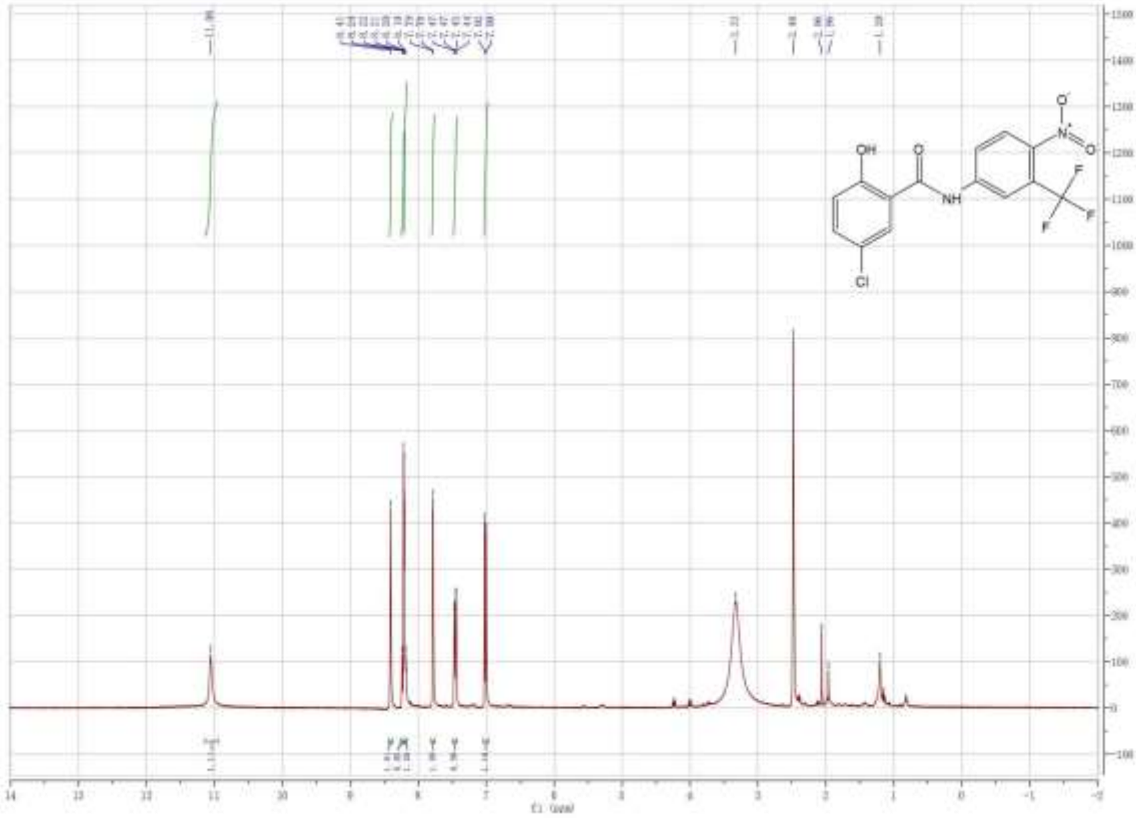

Figure 31. ^{1}H NMR spectrogram of Compound A16

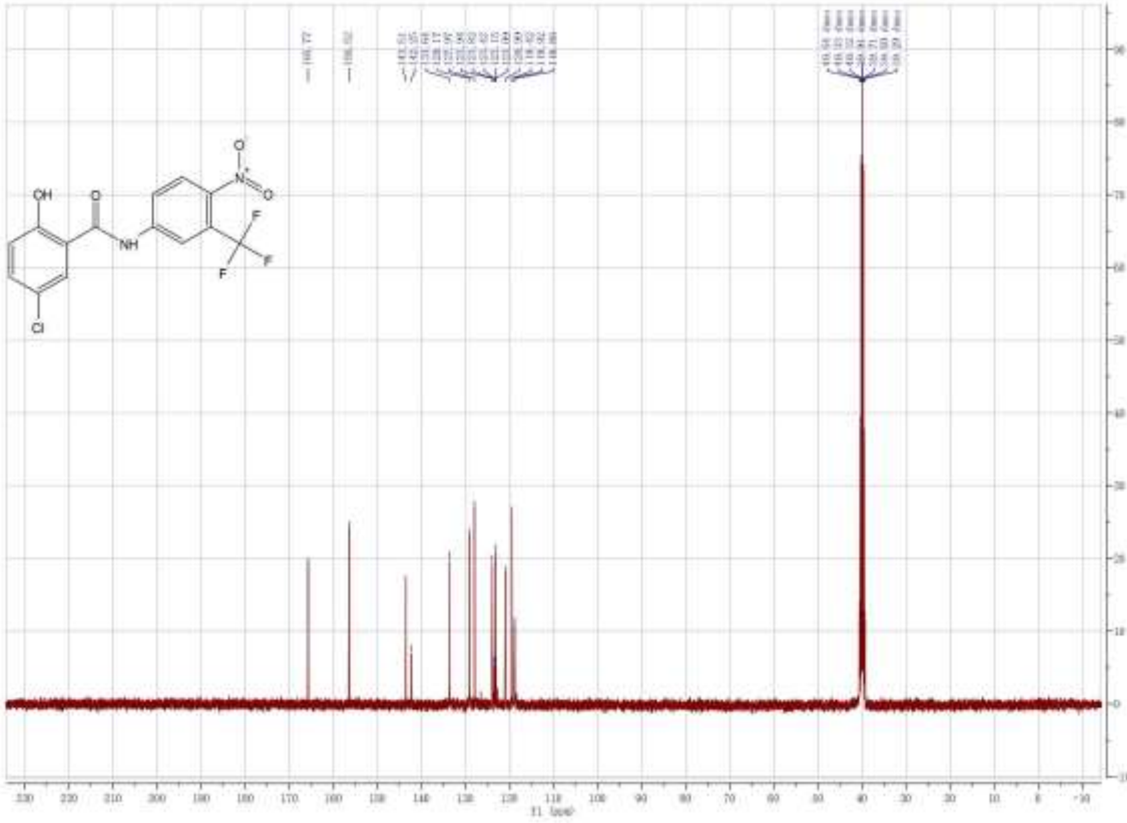

Figure 32. ^{13}C NMR spectrogram of Compound A16

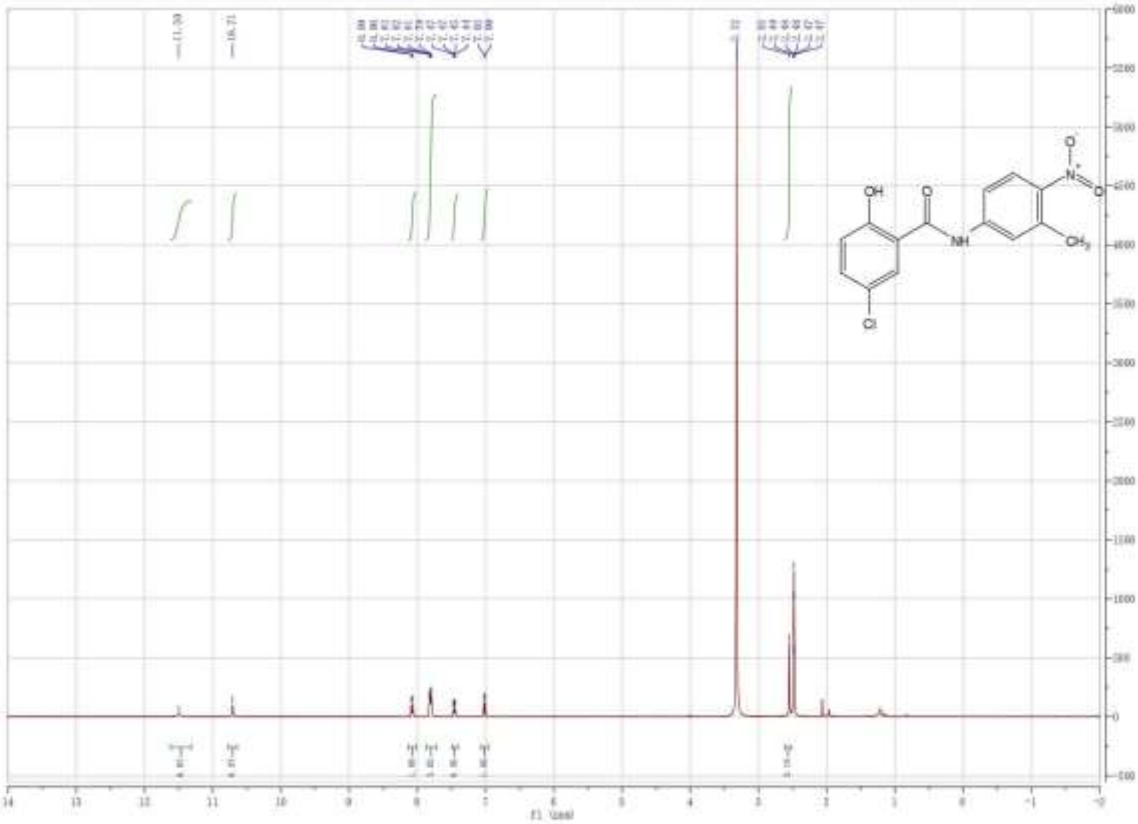

Figure 33. 1H NMR spectrogram of Compound A17

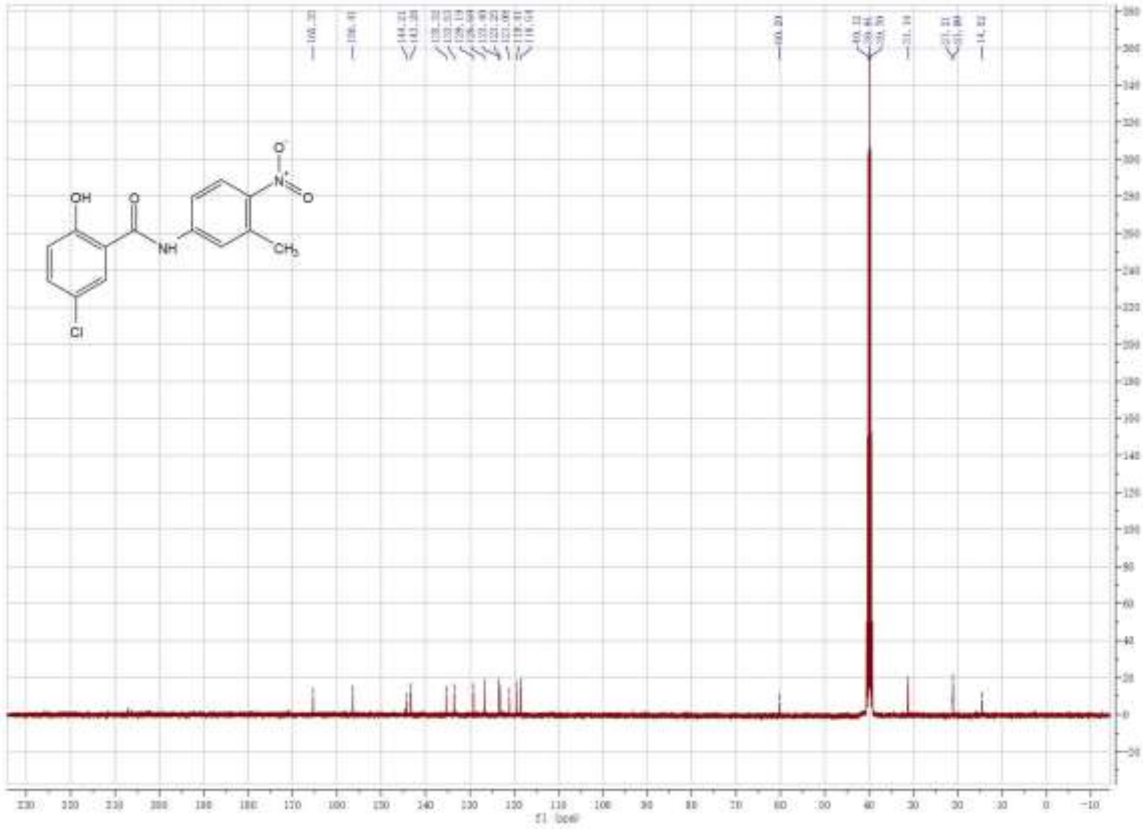

Figure 34. 13C NMR spectrogram of Compound A17

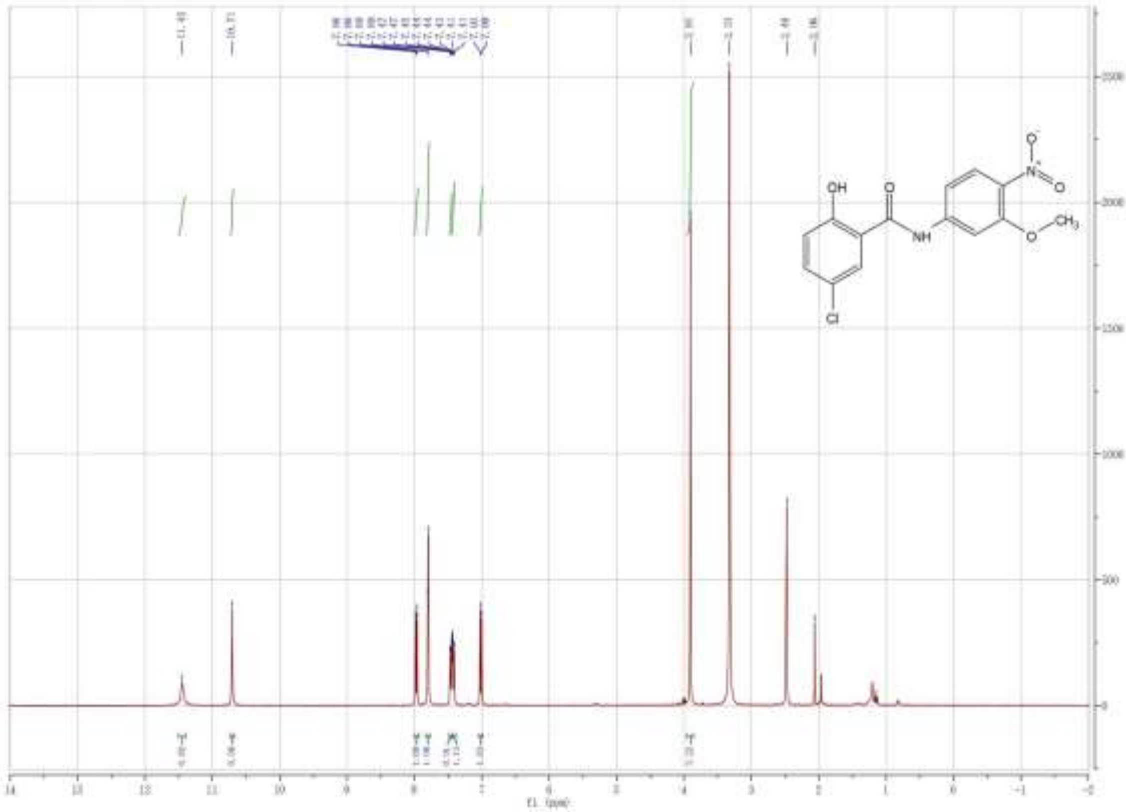

Figure 35. ^{1}H NMR spectrogram of Compound A18

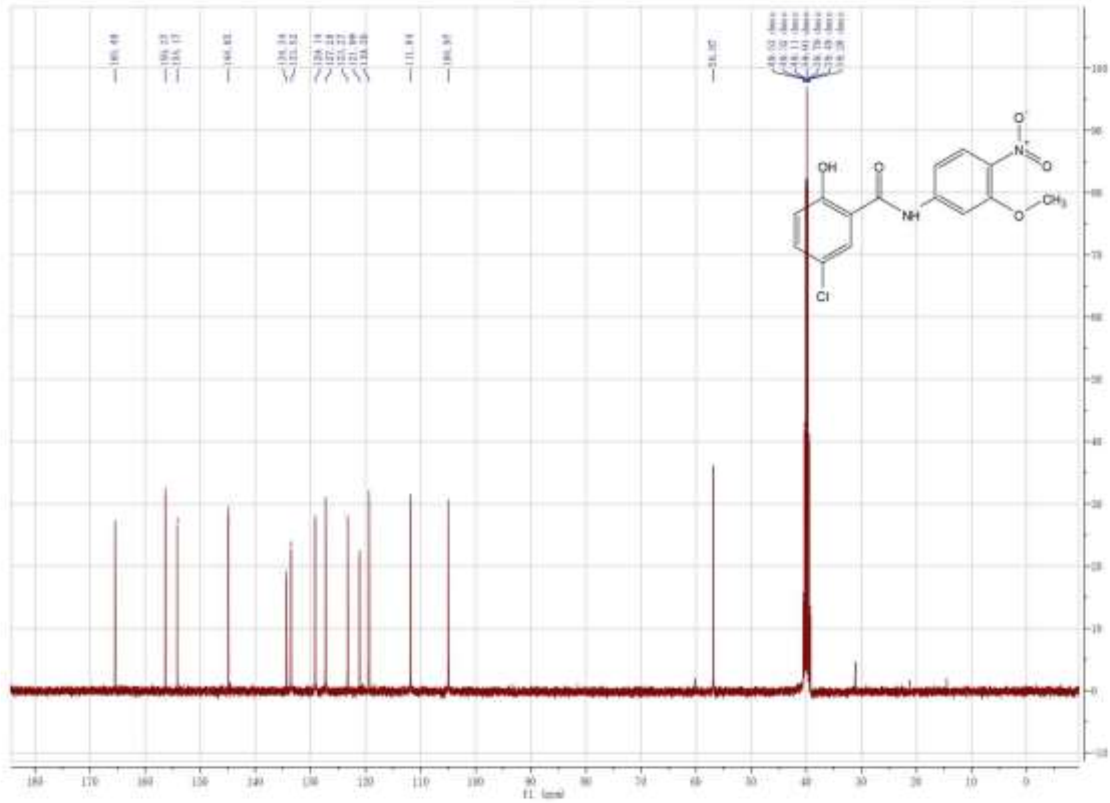

Figure 36. ^{13}C NMR spectrogram of Compound A18

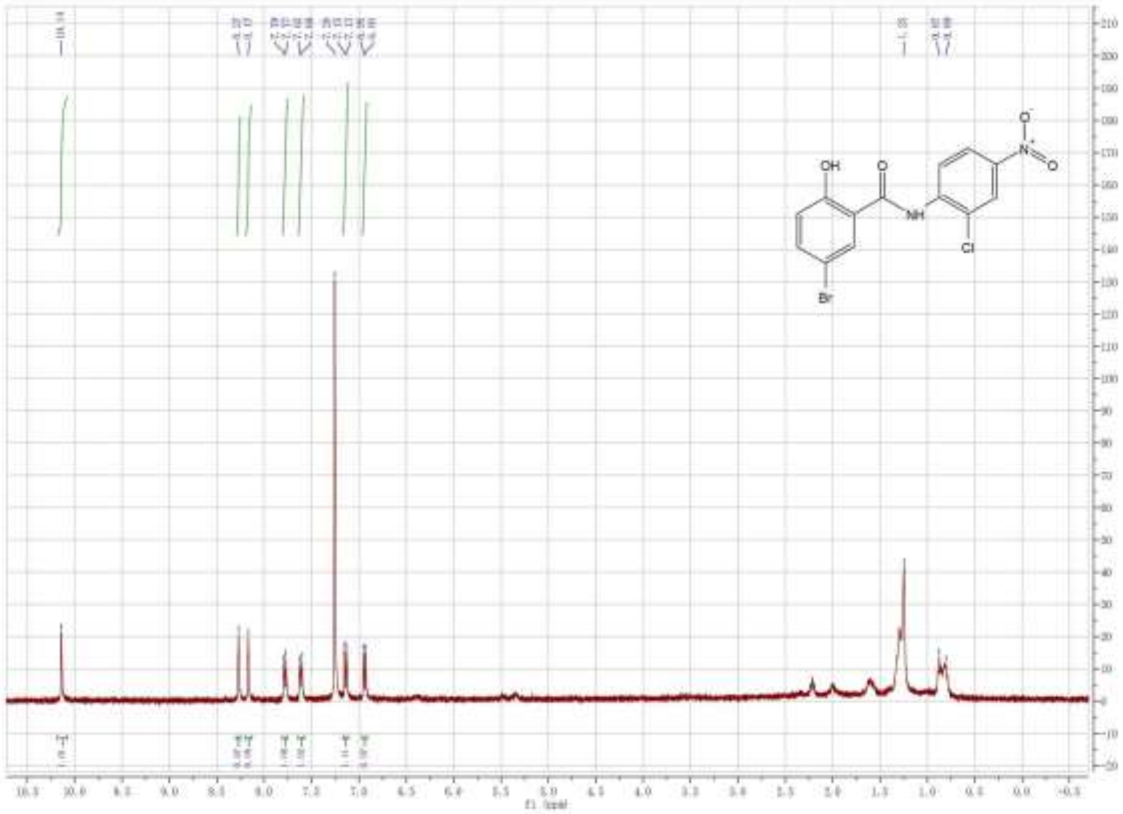

Figure 37. ¹H NMR spectrogram of Compound A19

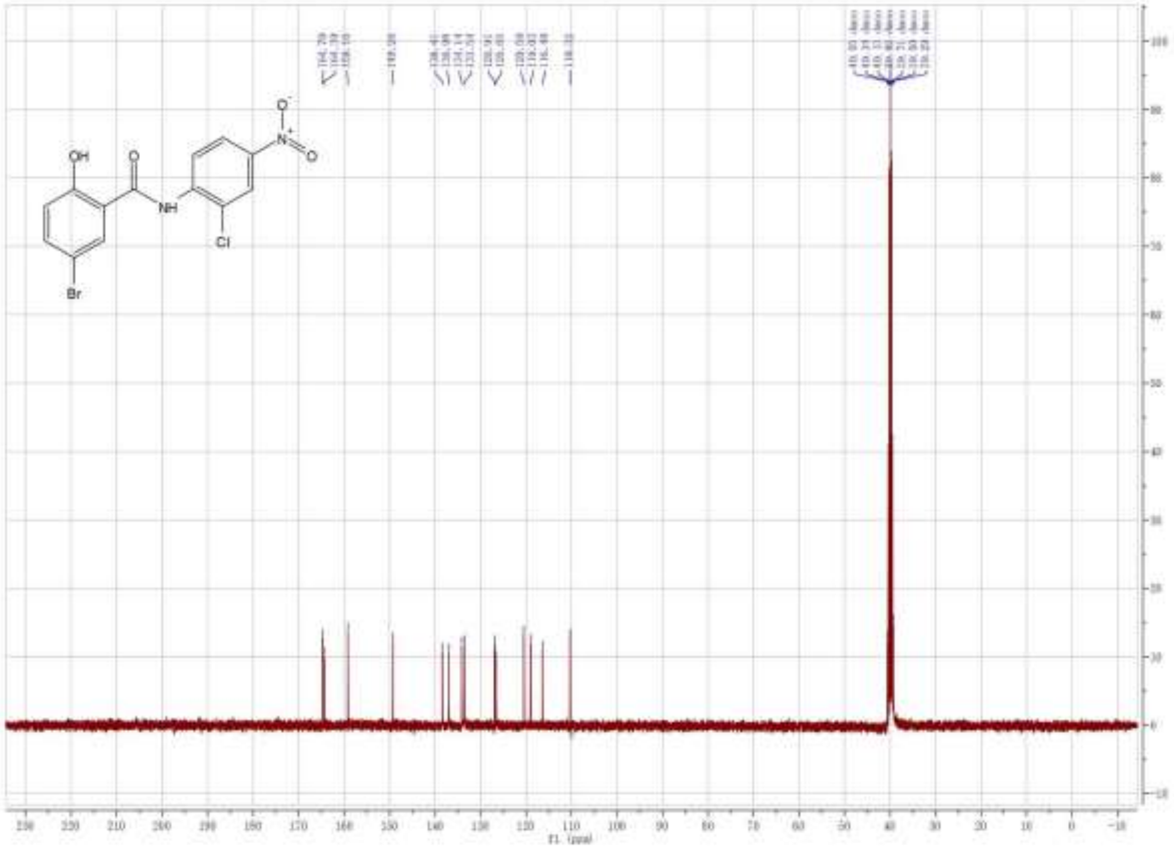

Figure 38. ¹³C NMR spectrogram of Compound A19

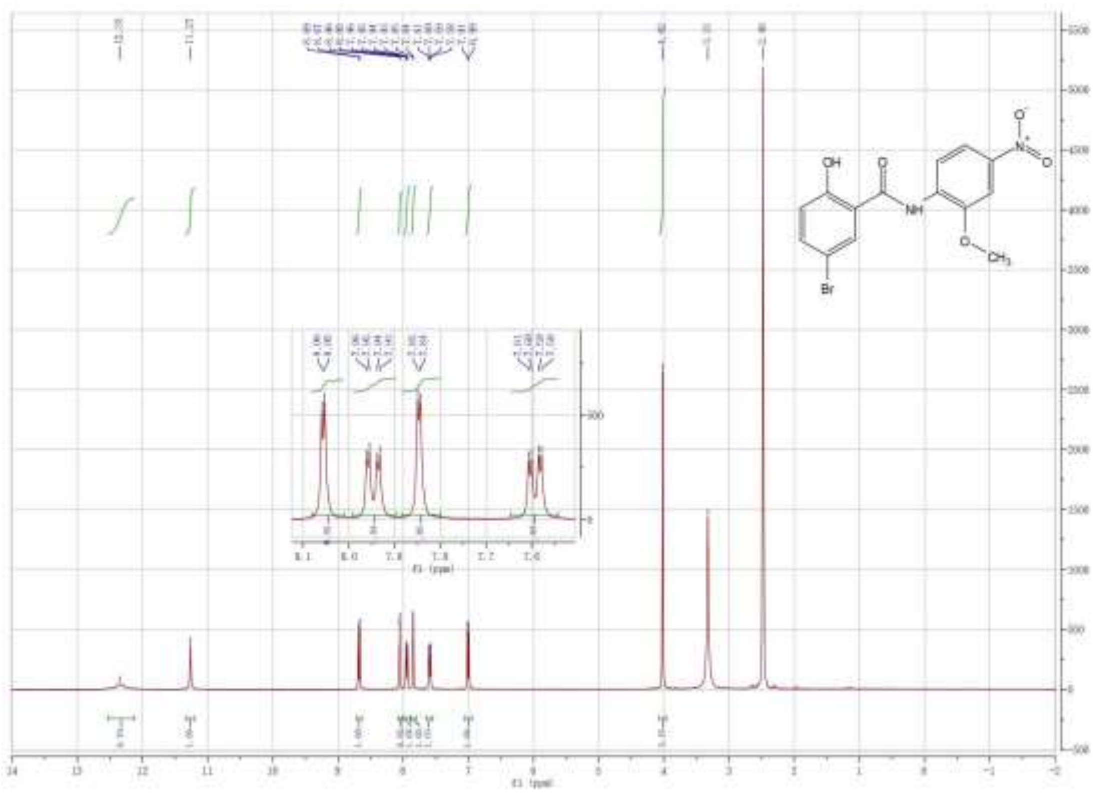

Figure 39. ^{1}H NMR spectrogram of Compound A20

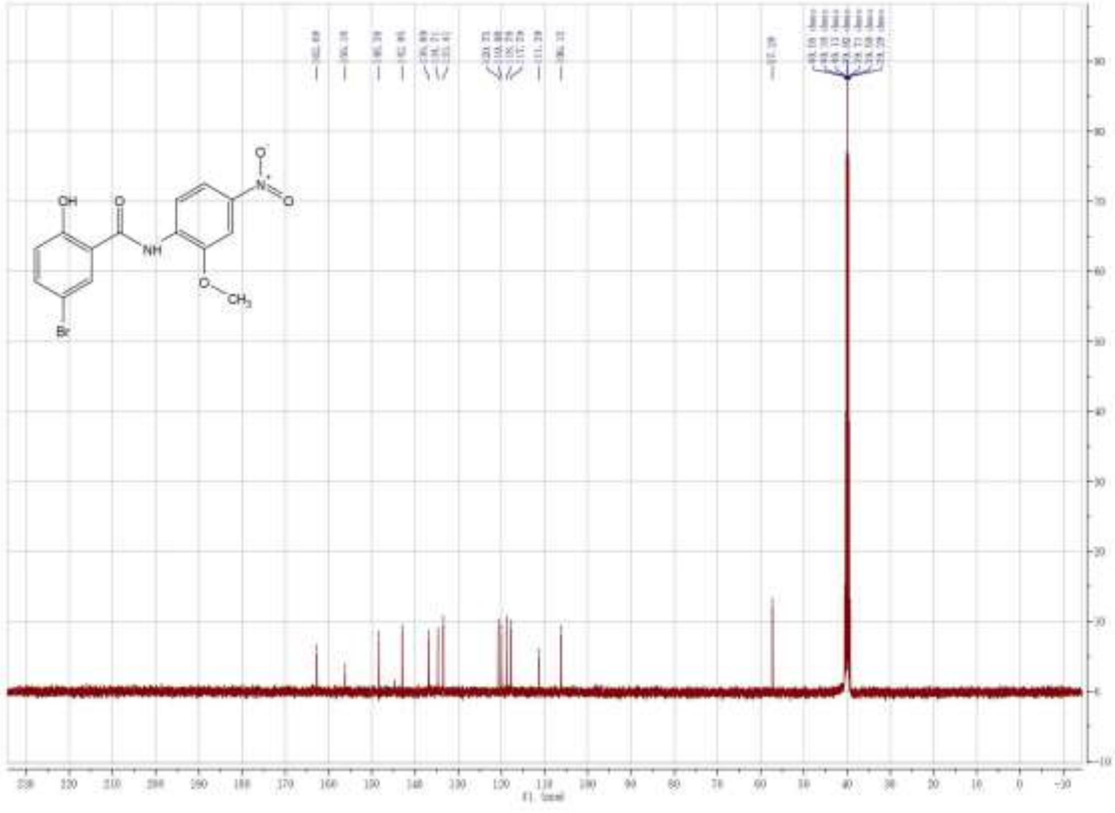

Figure 40. ^{13}C NMR spectrogram of Compound A20

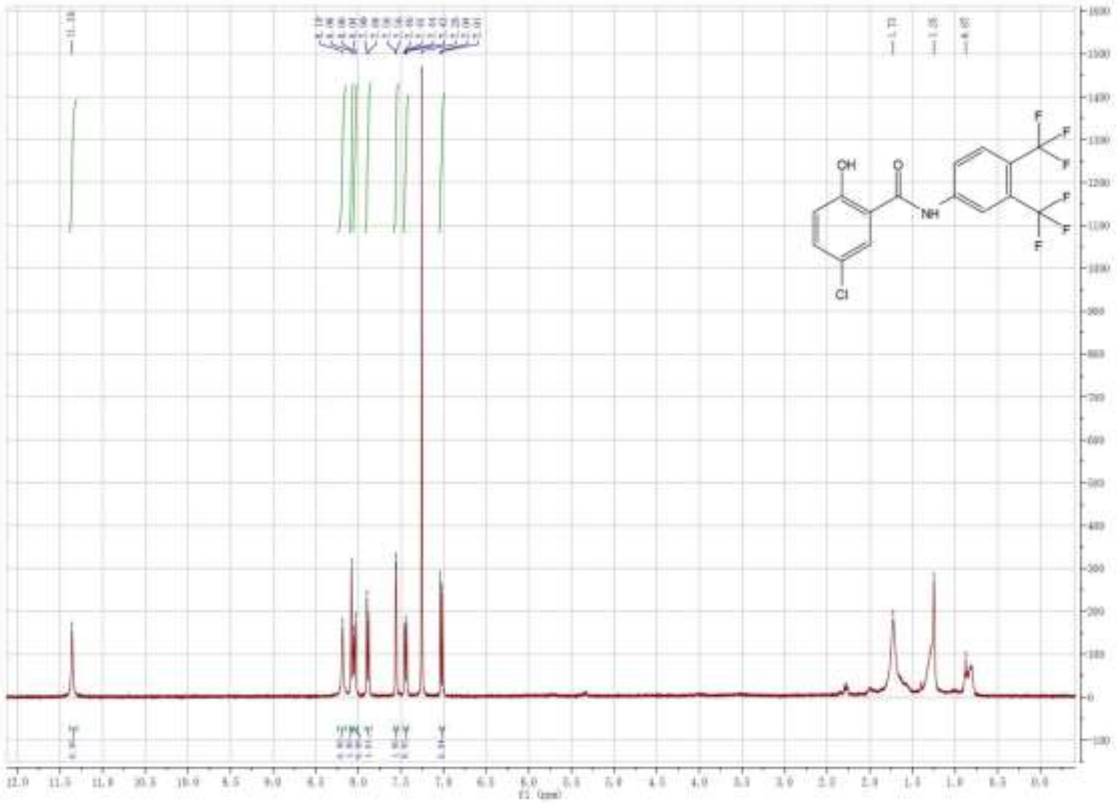

Figure 41. ^{1}H NMR spectrogram of Compound B1

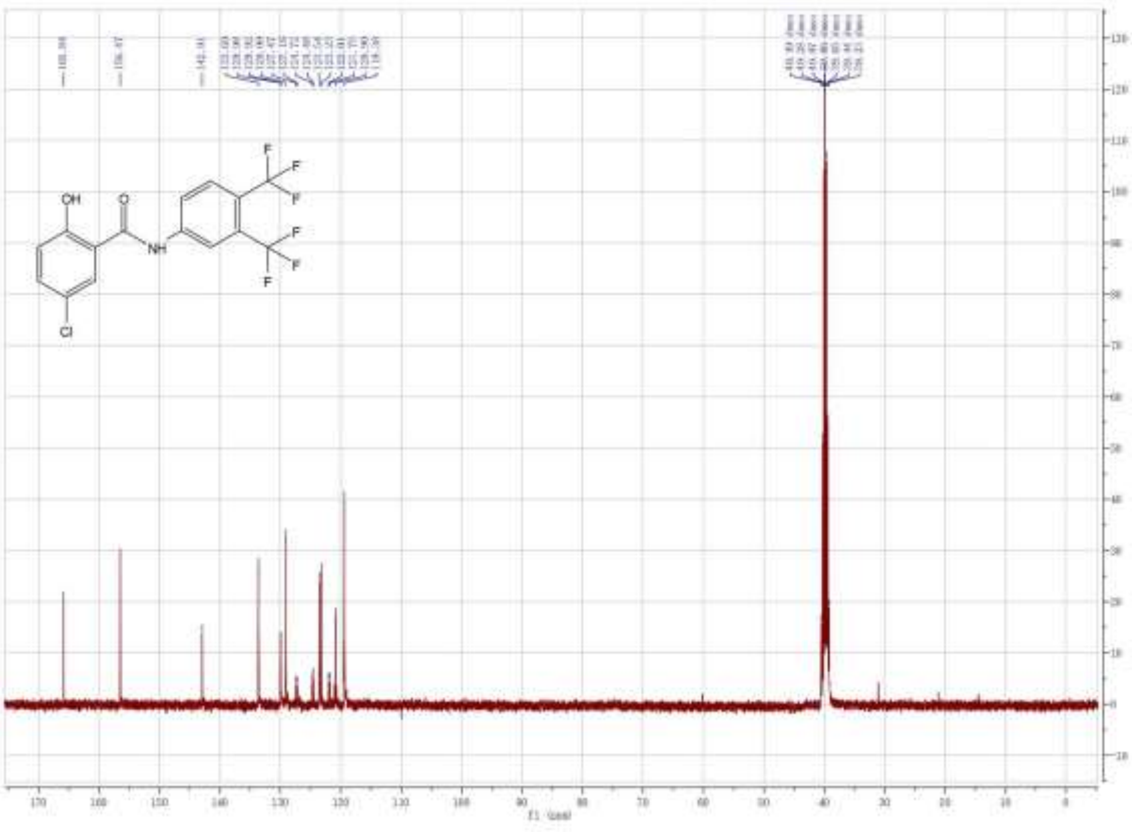

Figure 42. ^{13}C NMR spectrogram of Compound B1

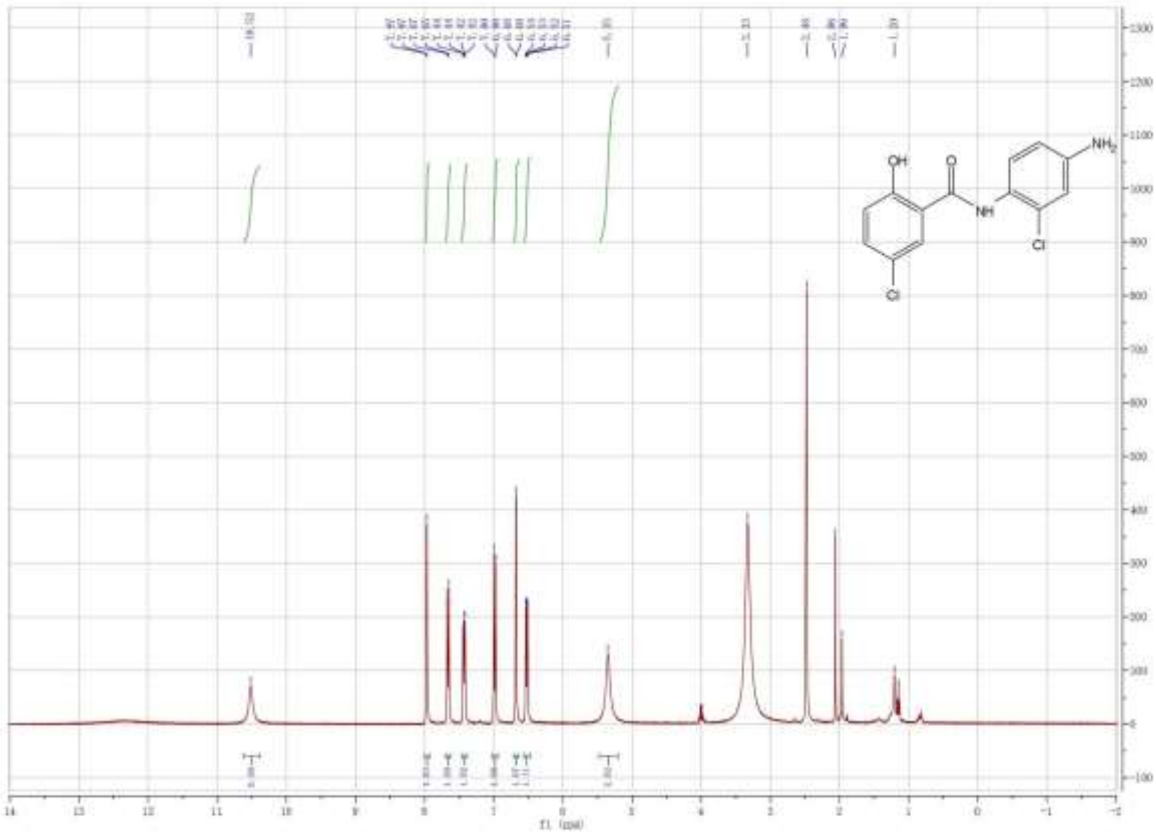

Figure 43. ¹H NMR spectrogram of Compound B2

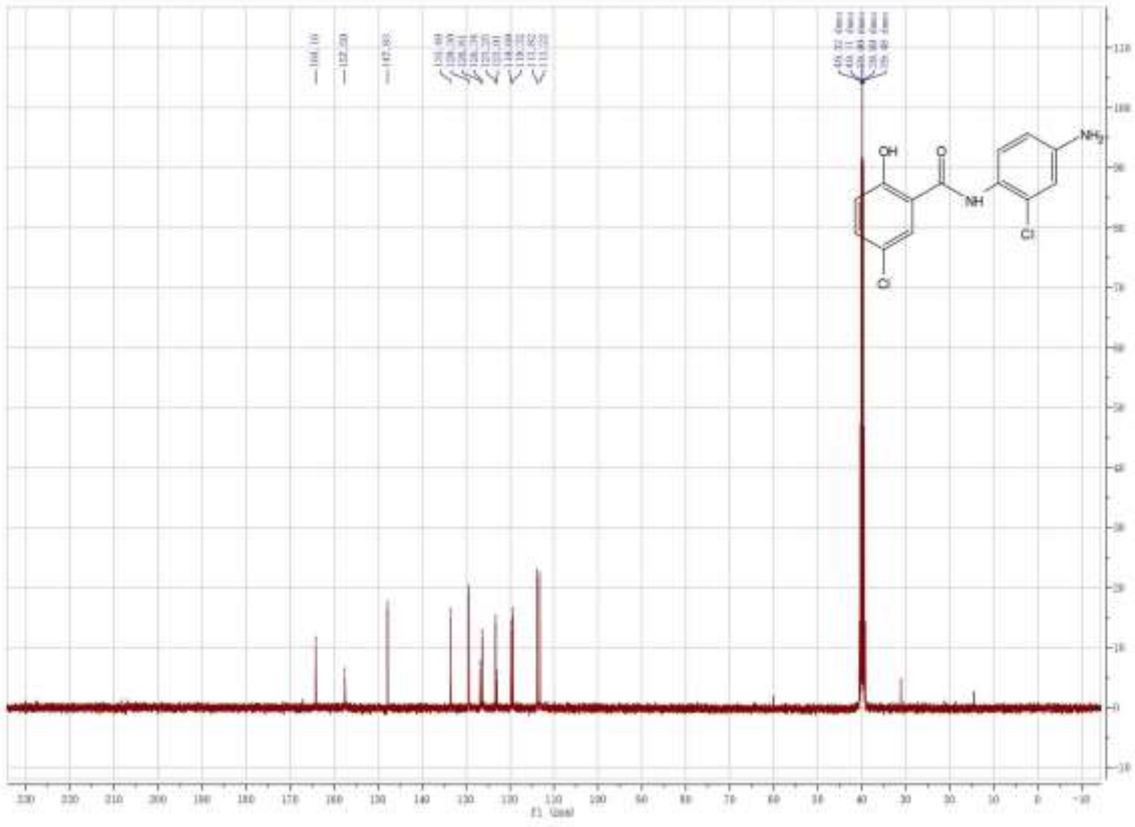

Figure 44. ¹³C NMR spectrogram of Compound B2

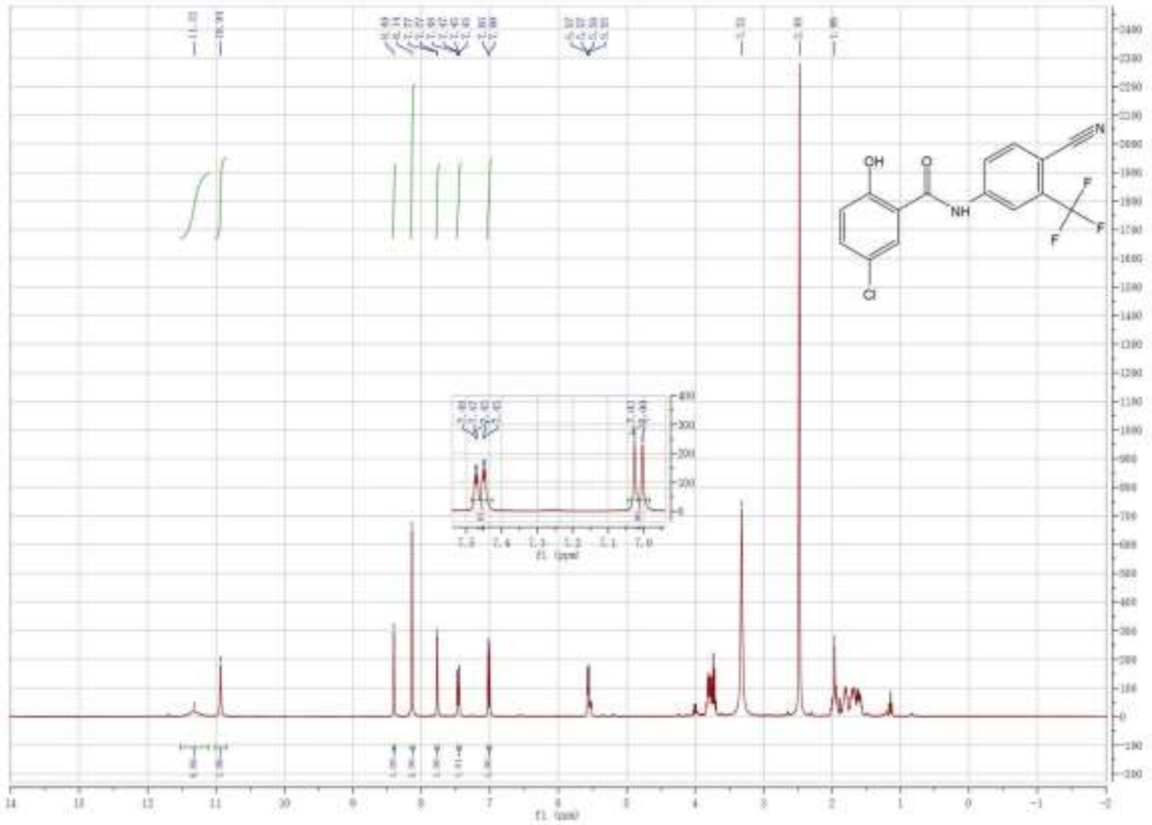

Figure 45. ^{1}H NMR spectrogram of Compound B3

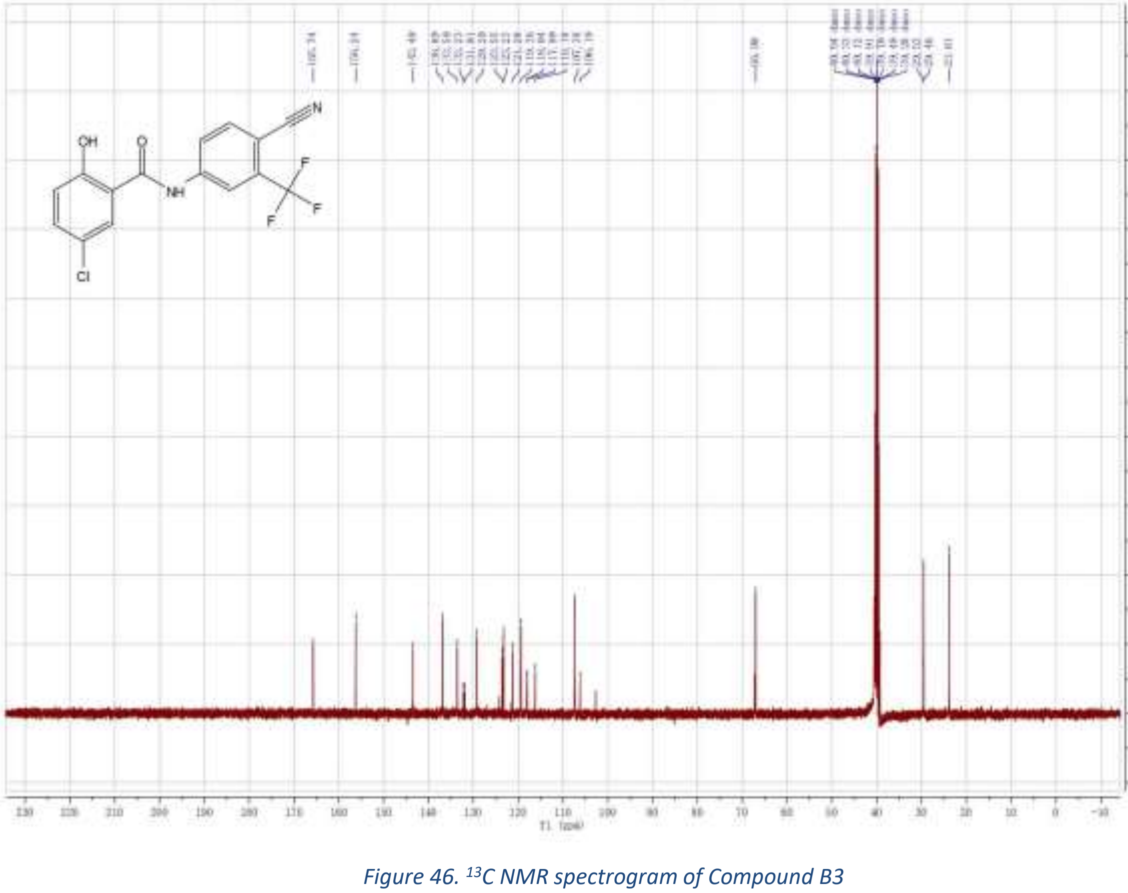

Figure 46. ^{13}C NMR spectrogram of Compound B3

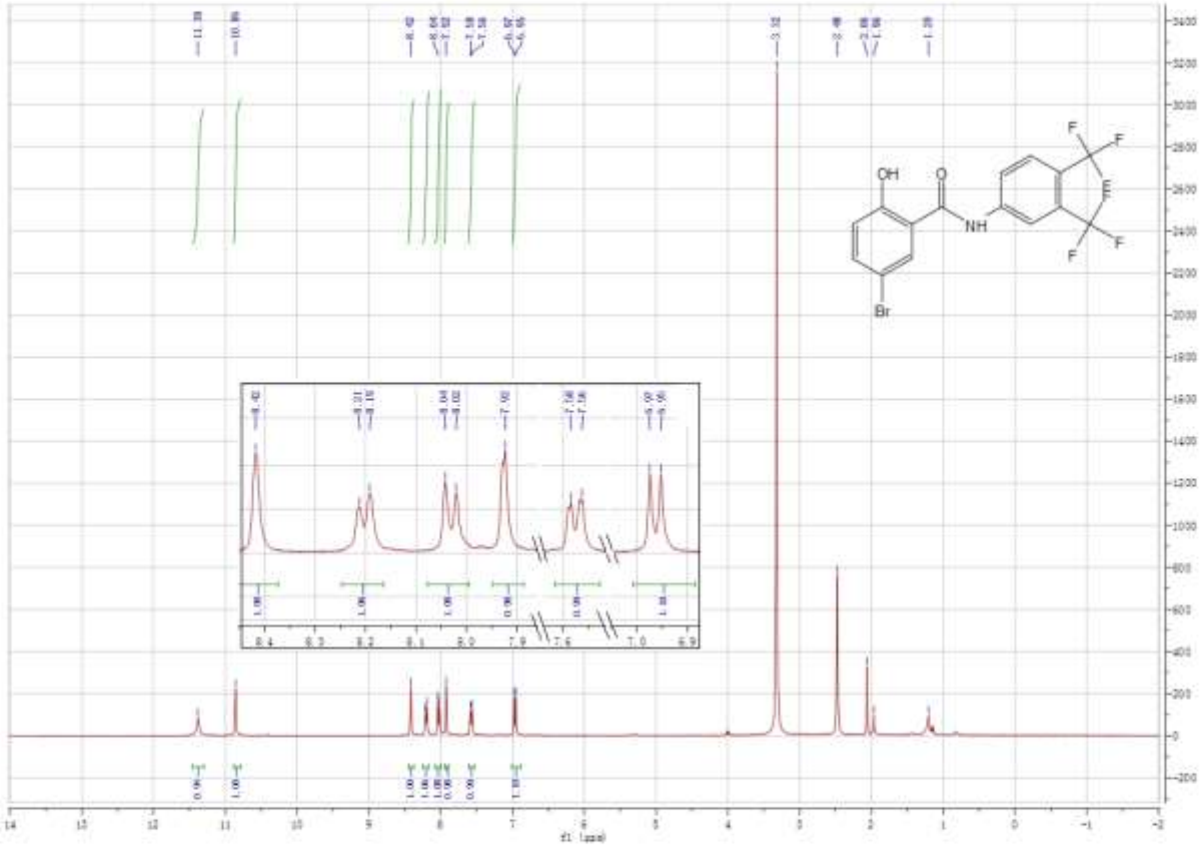

Figure 47. ^{1}H NMR spectrogram of Compound B4

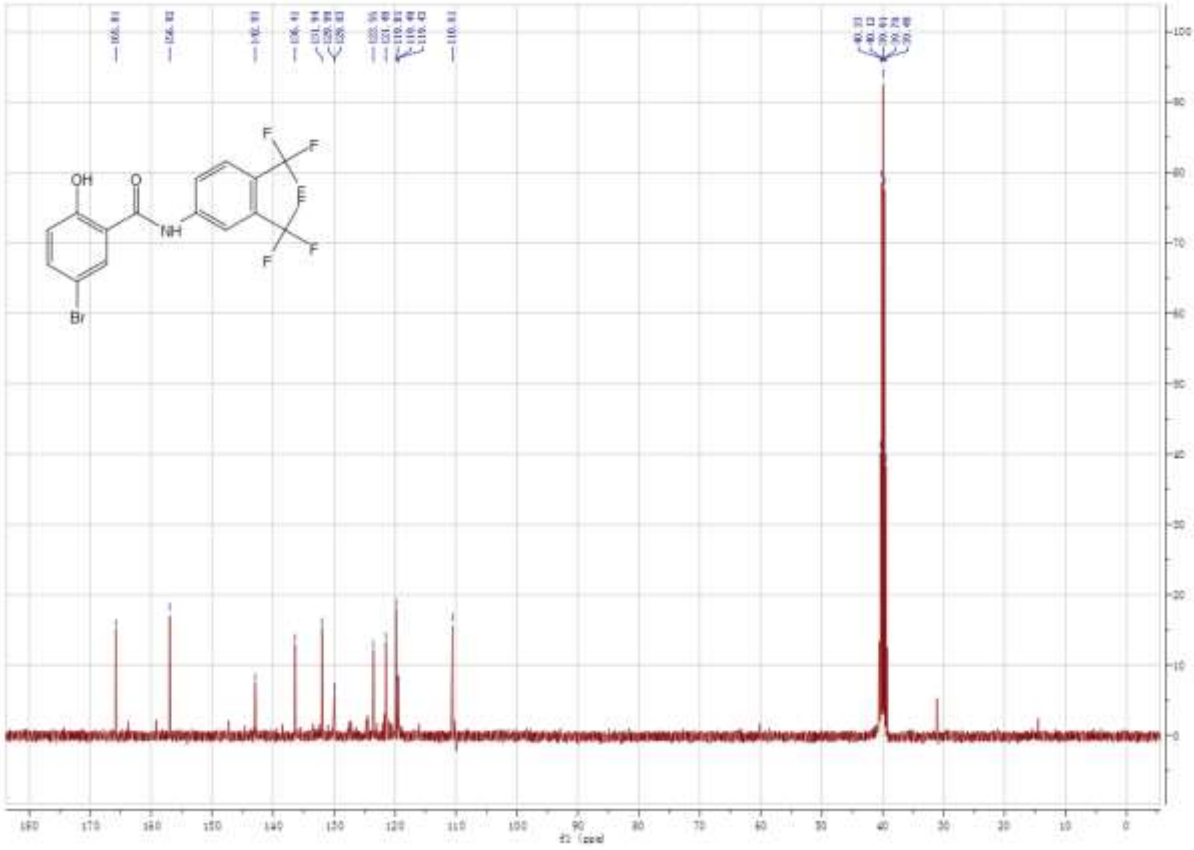

Figure 48. ^{13}C NMR spectrogram of Compound B4

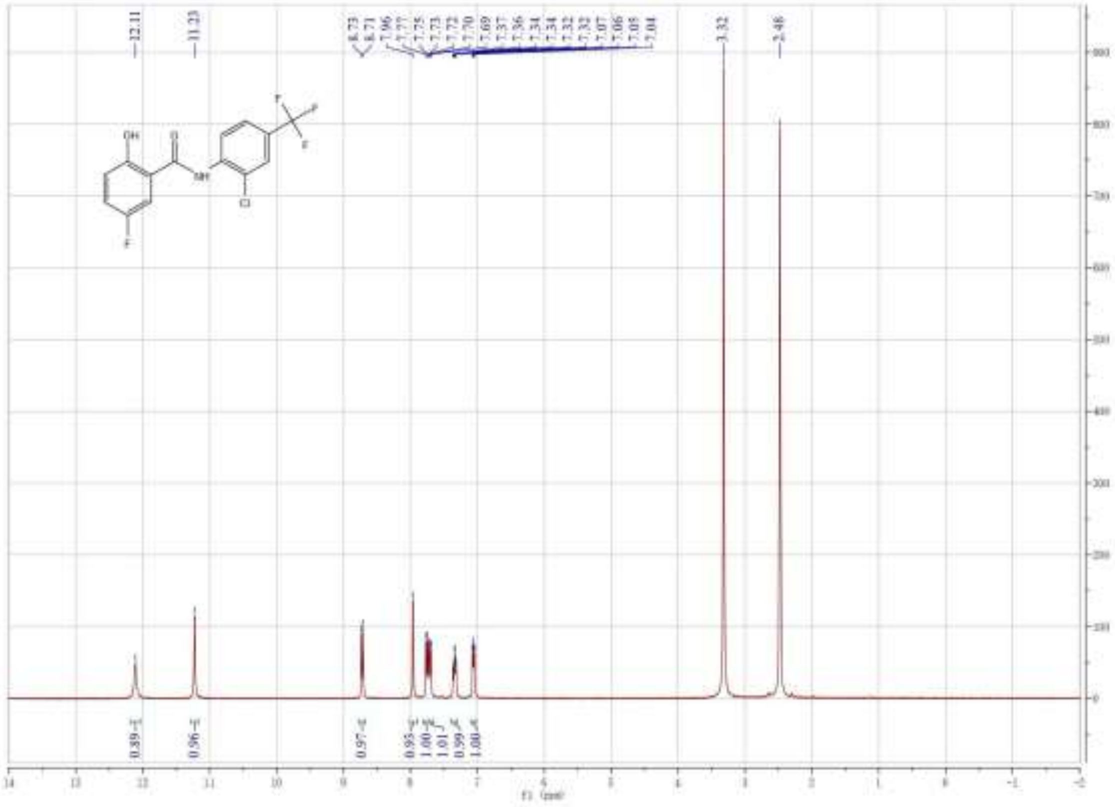

Figure 49. ^{1}H NMR spectrogram of Compound B5

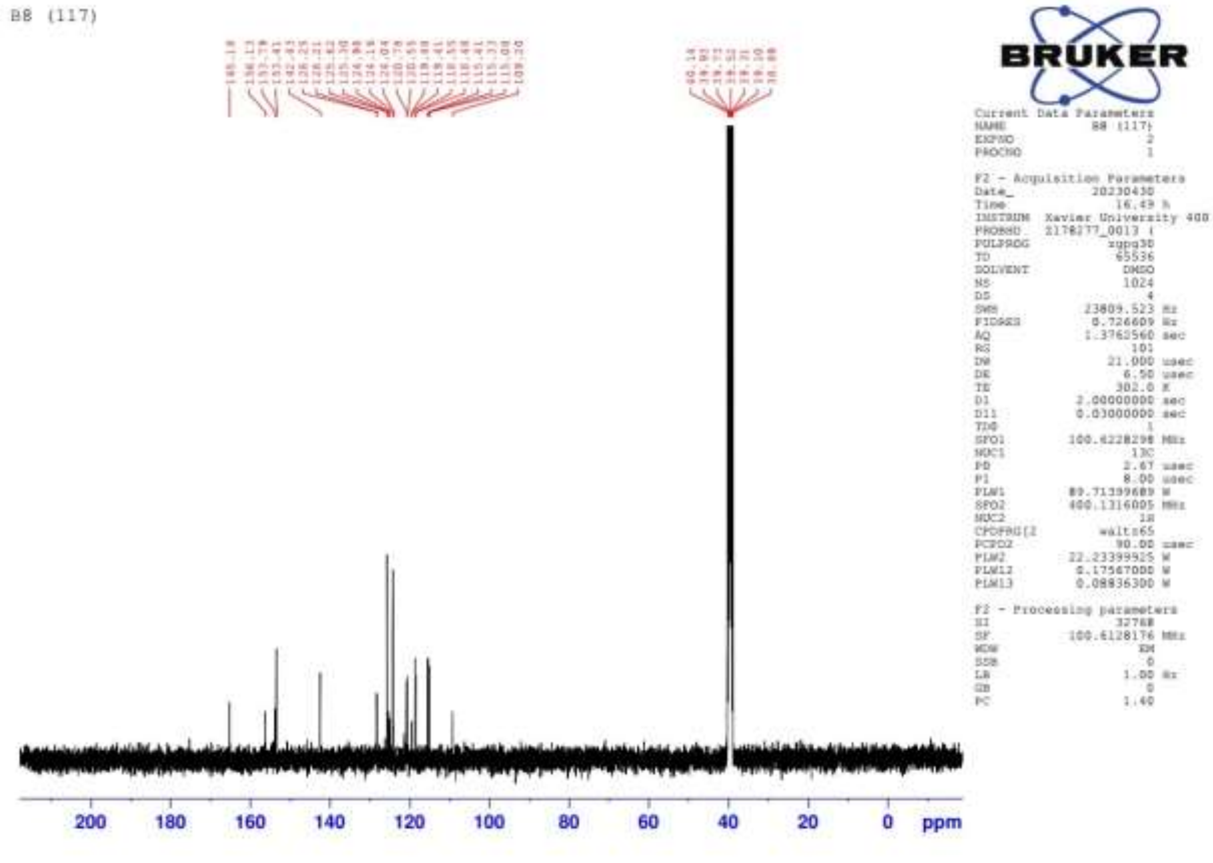

Figure 50. ^{13}C NMR spectrogram of Compound B5

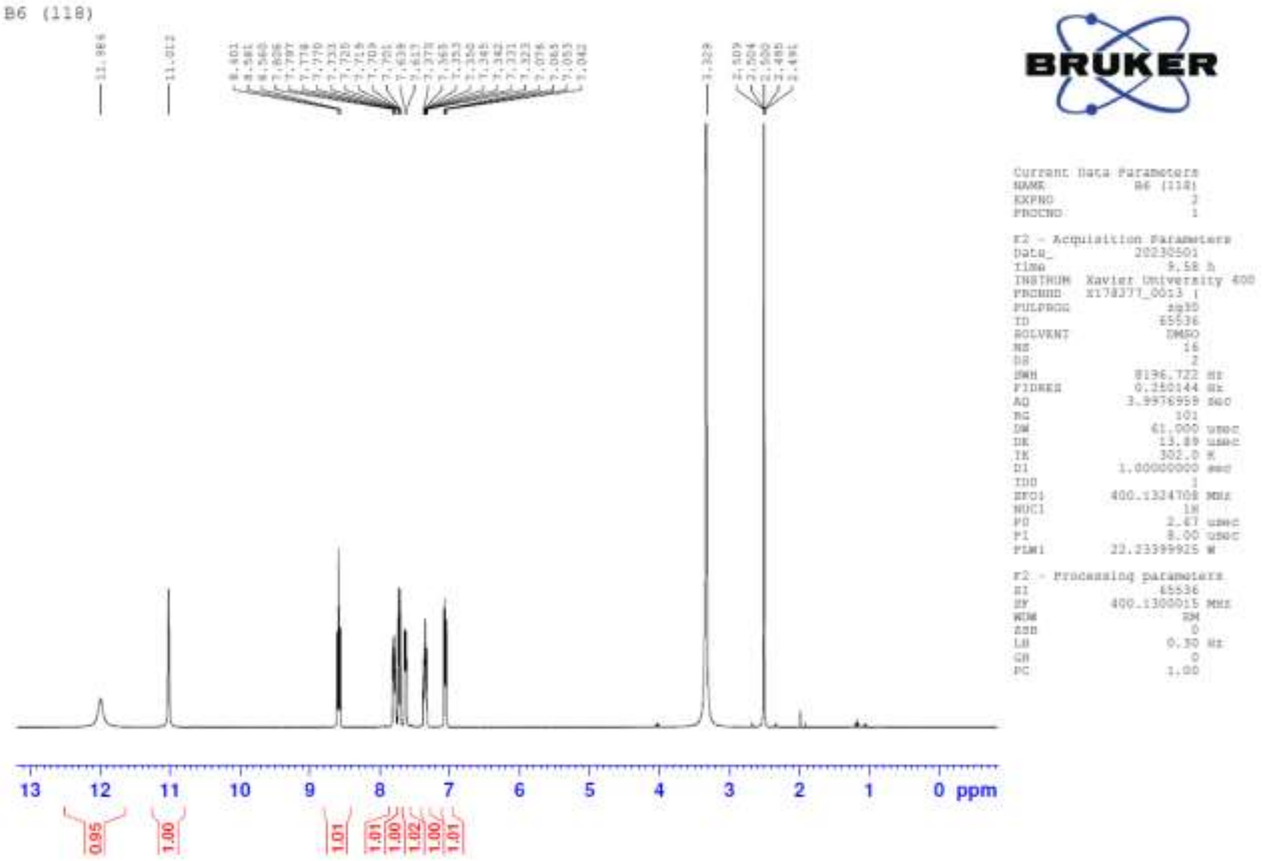

Figure 51. ^{1}H NMR spectrogram of Compound B6

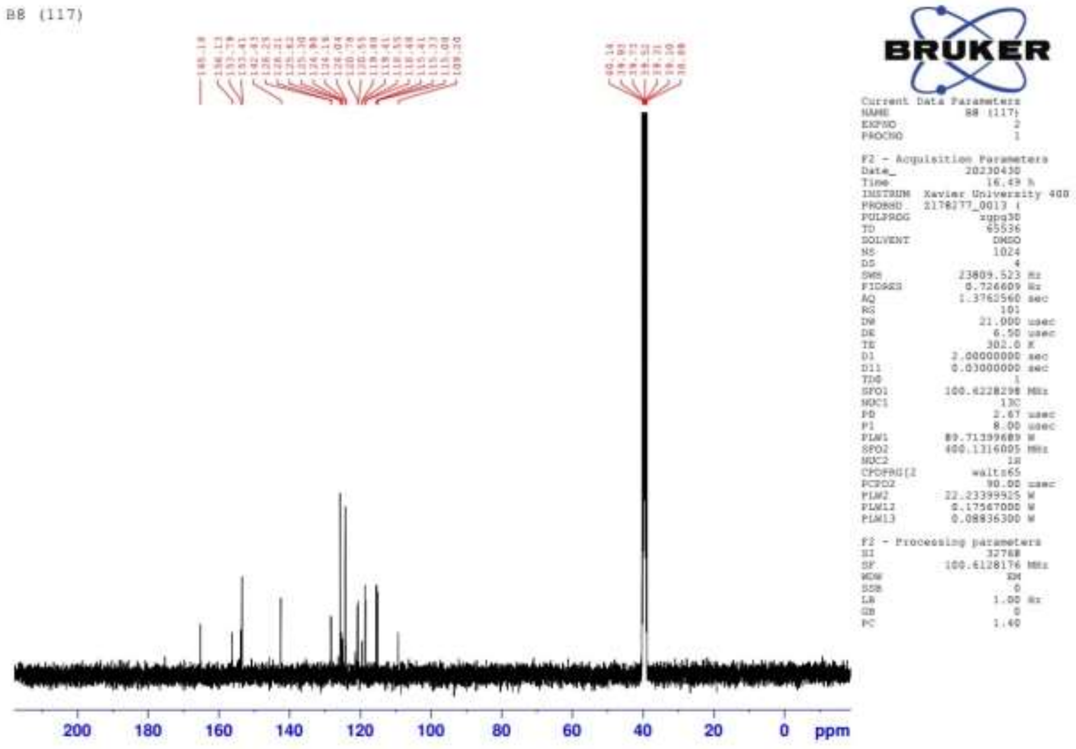

Figure 52. ^{13}C NMR spectrogram of Compound B6

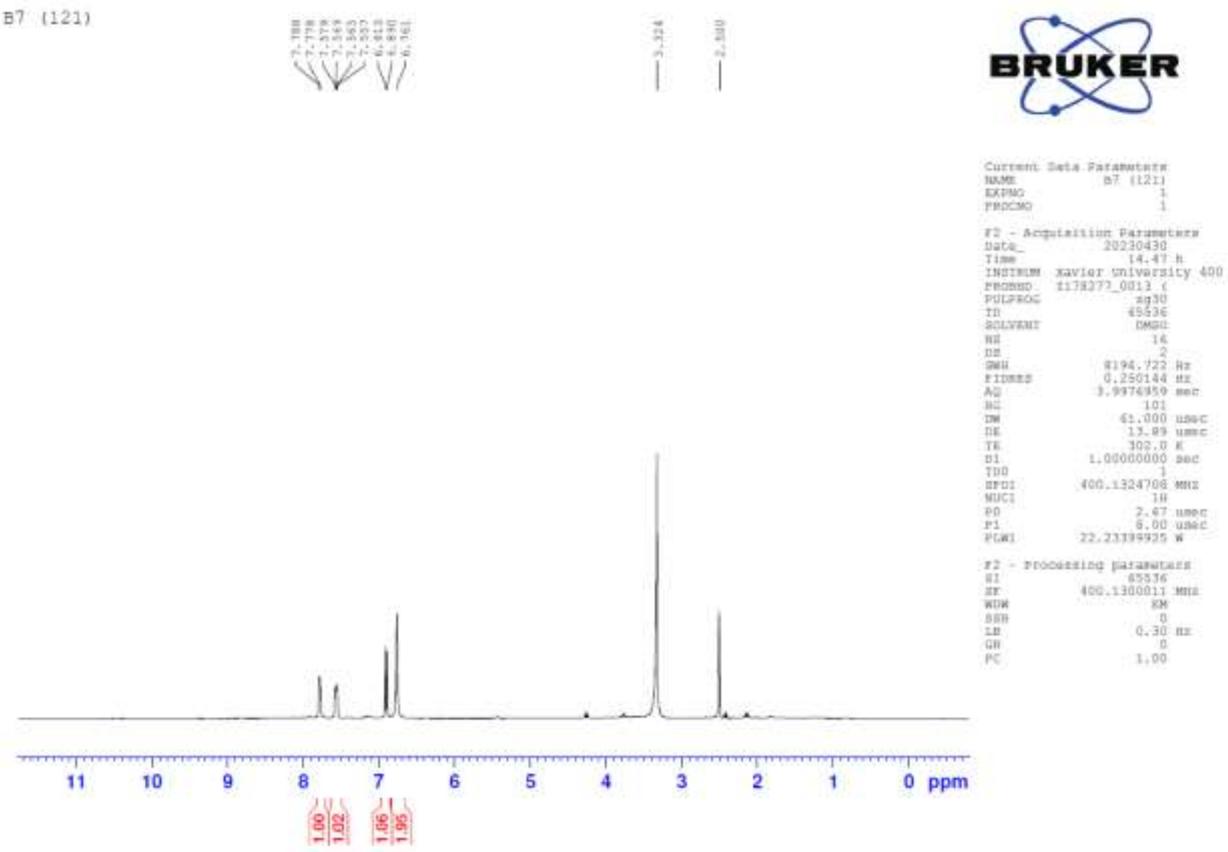

Figure 53. ^{1}H NMR spectrogram of Compound B7

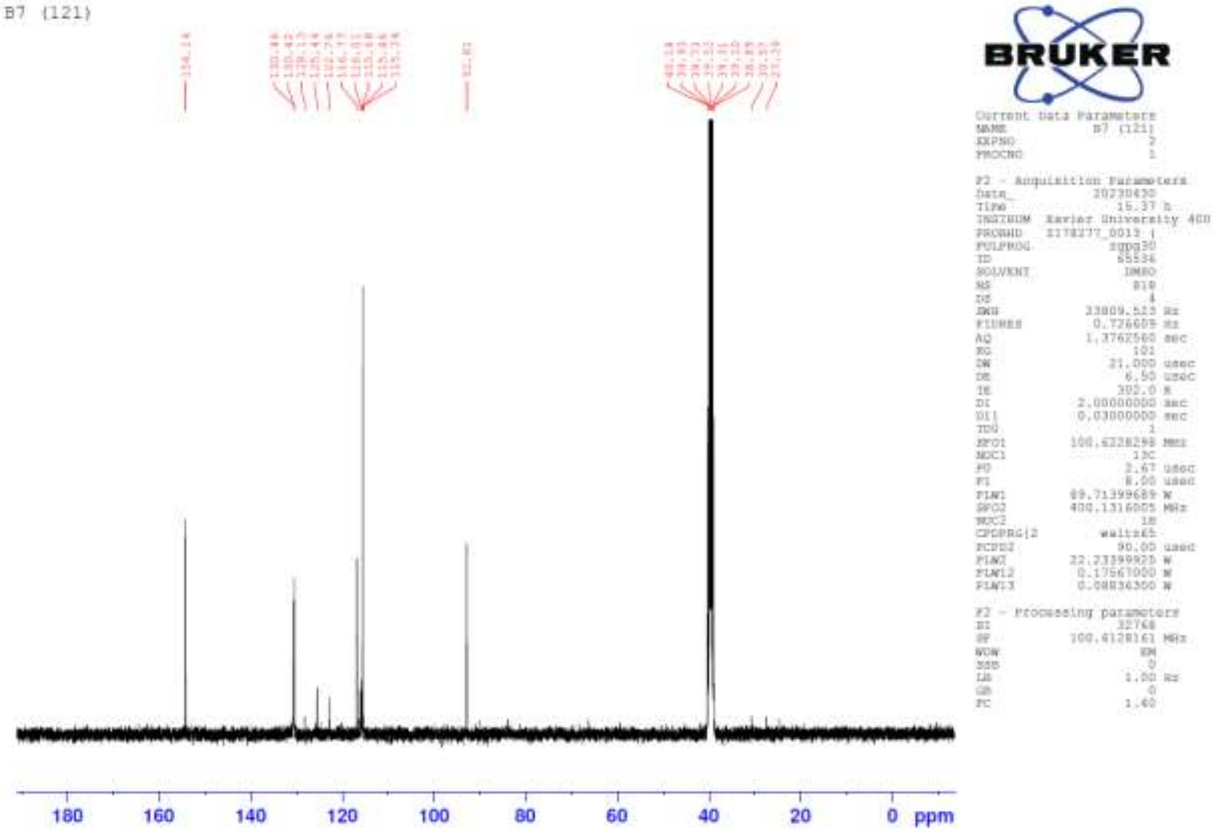

Figure 54. ^{13}C NMR spectrogram of Compound B7

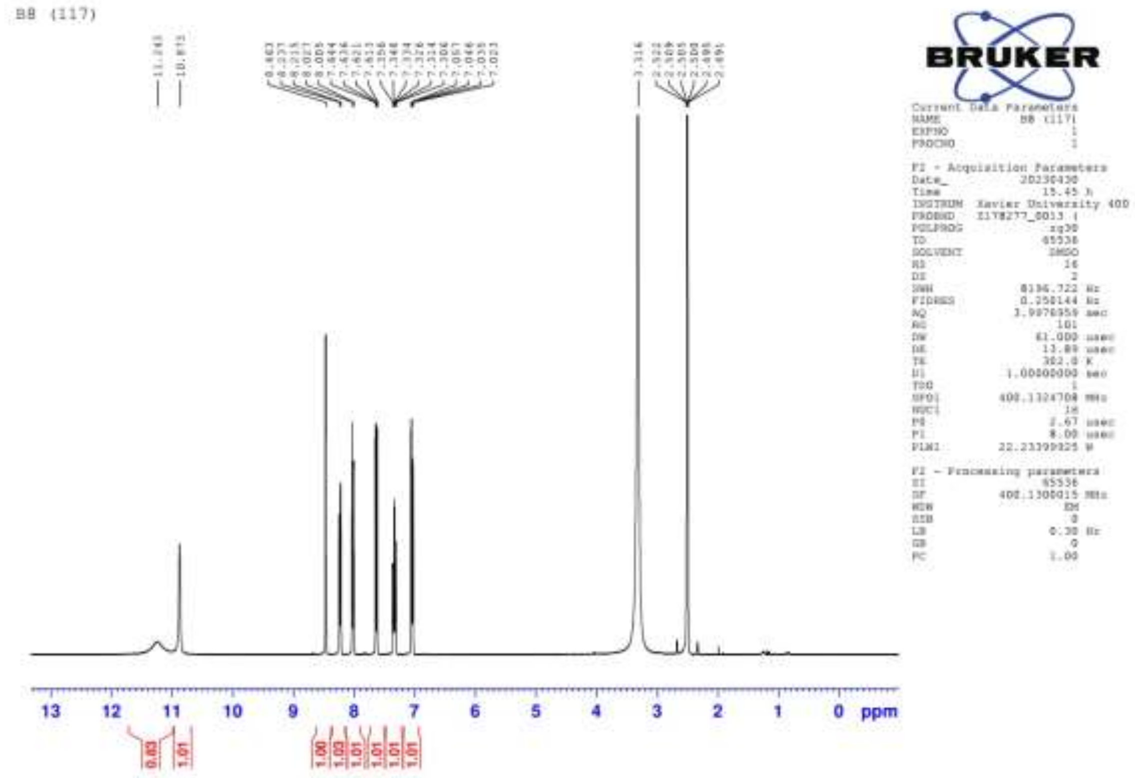

Figure 55. ^{1}H NMR spectrogram of Compound B8

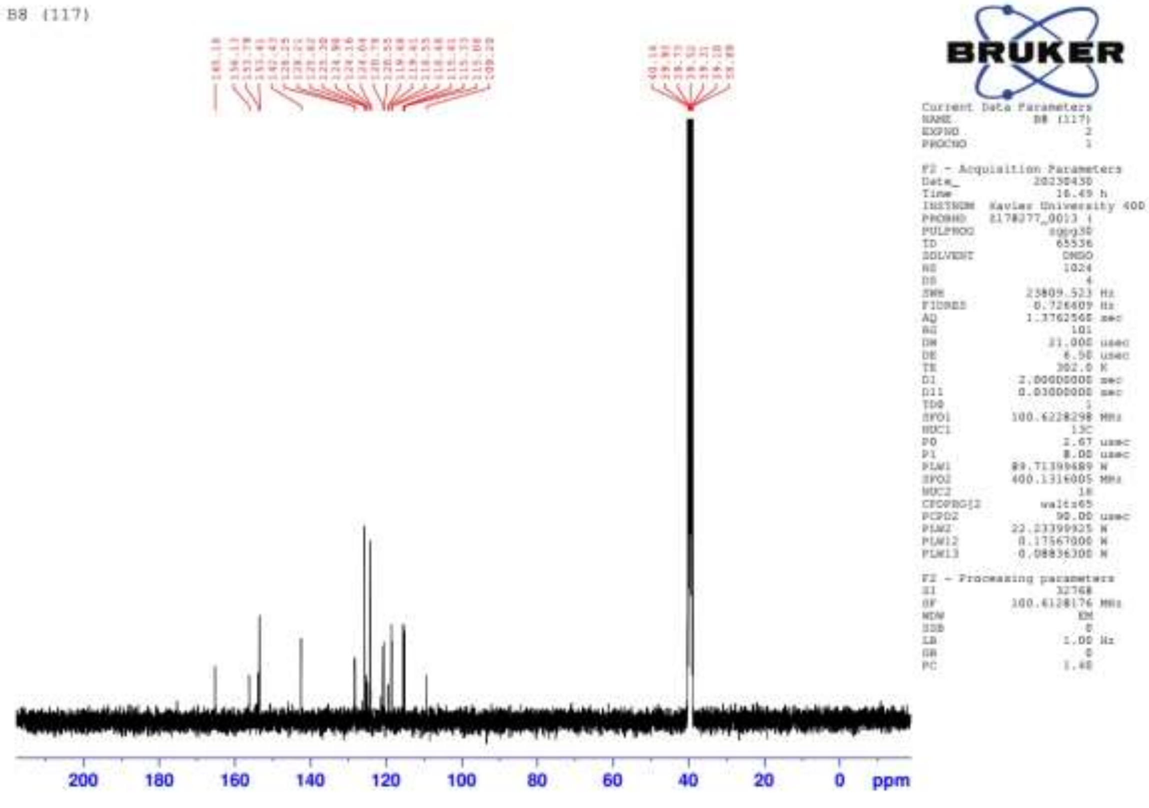

Figure 56. ^{13}C NMR spectrogram of Compound B8

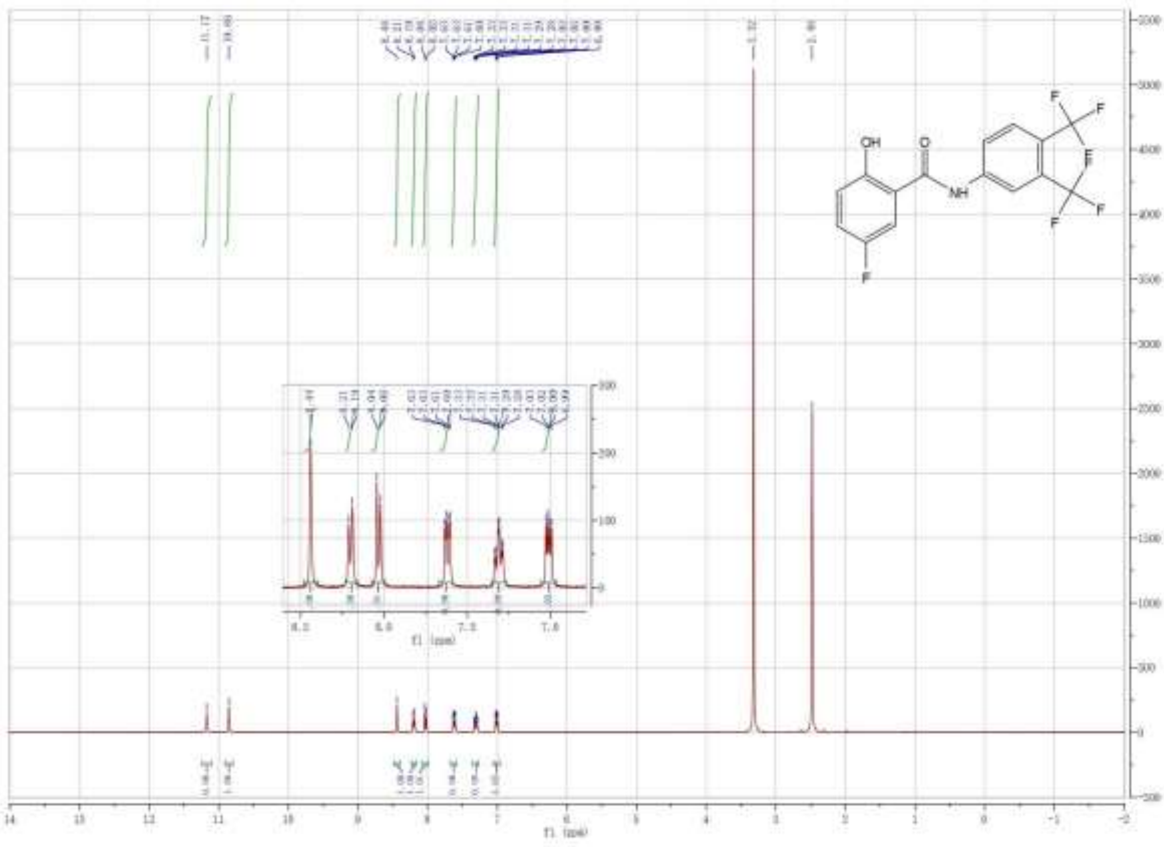

Figure 57. ^{1}H NMR spectrogram of Compound B9

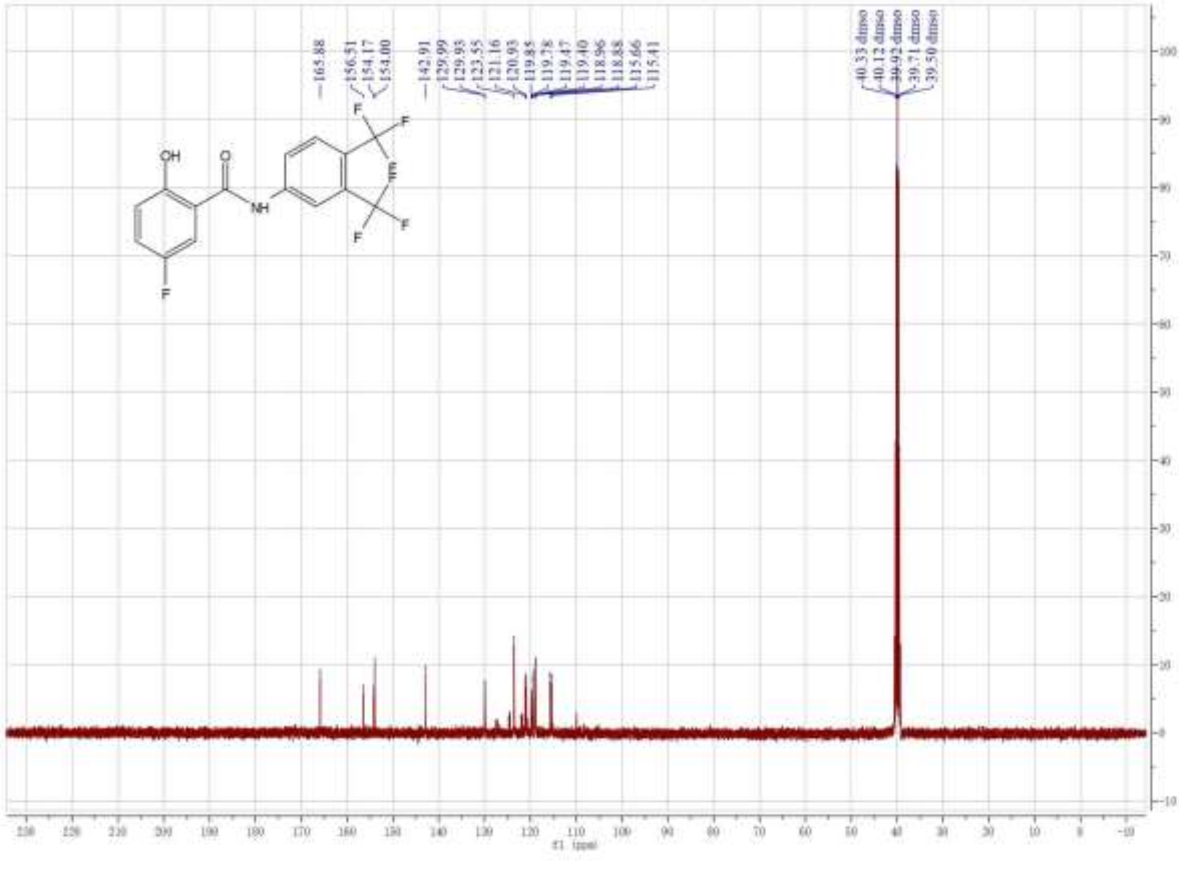

Figure 58. ^{13}C NMR spectrogram of Compound B9

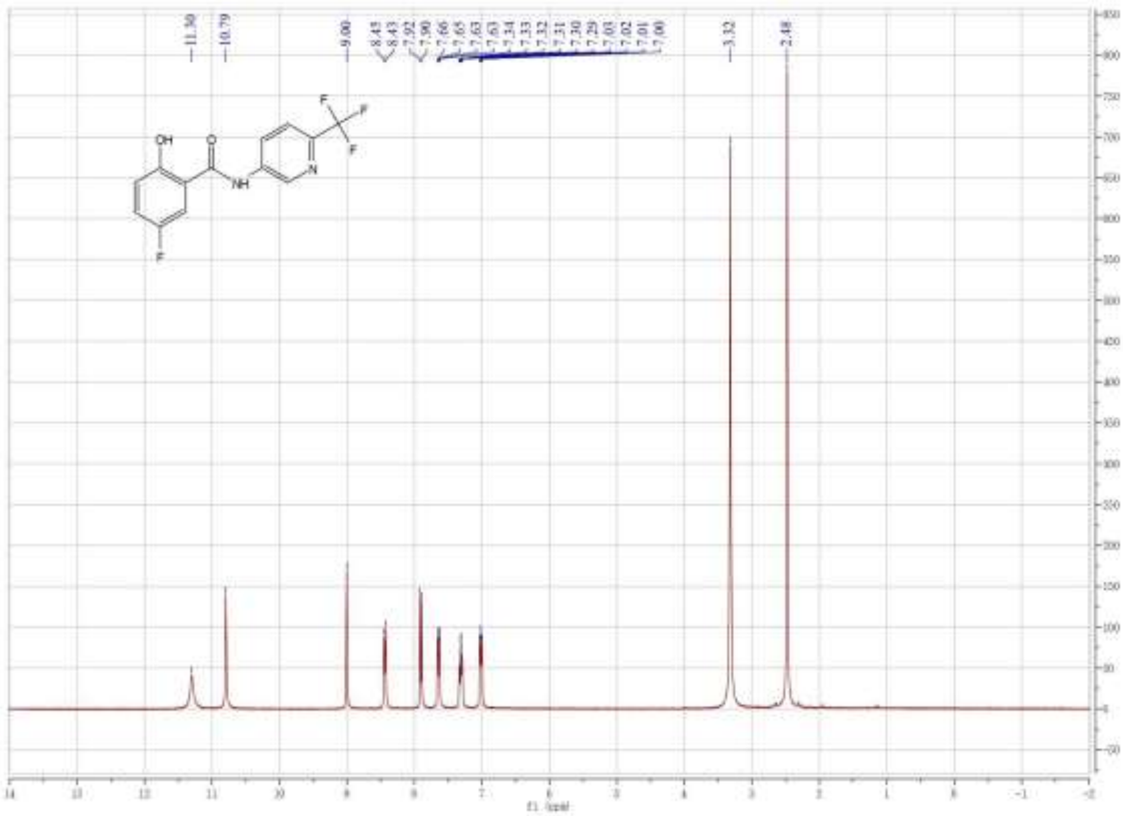

Figure 59. ¹H NMR spectrogram of Compound B10

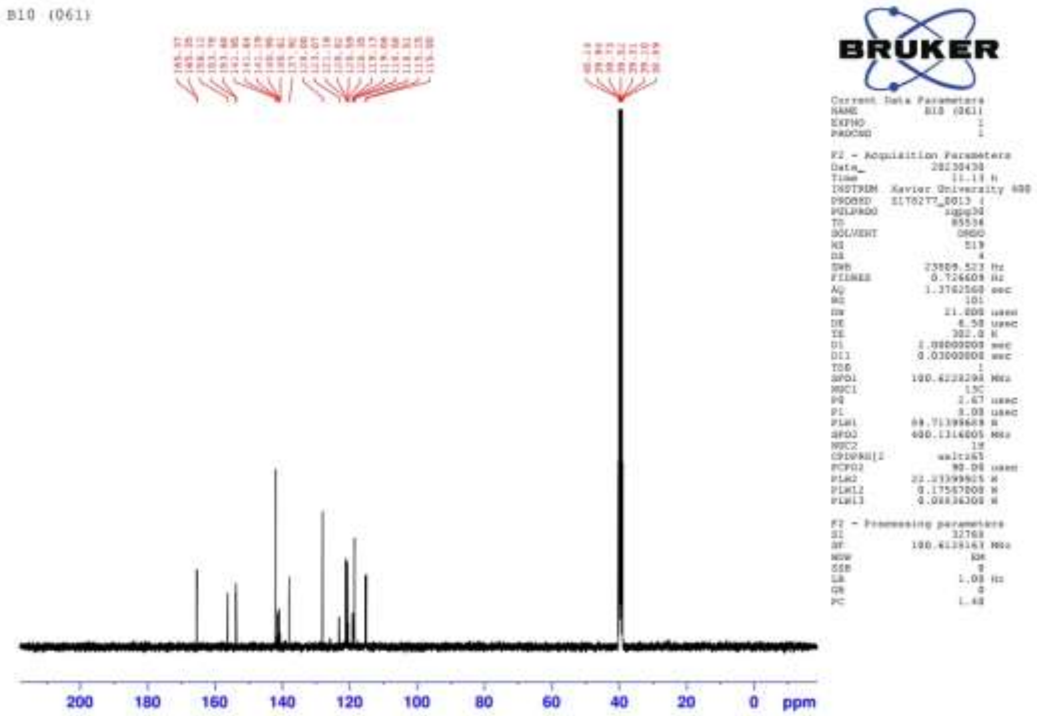

Figure 60. ¹³C NMR spectrogram of Compound B10

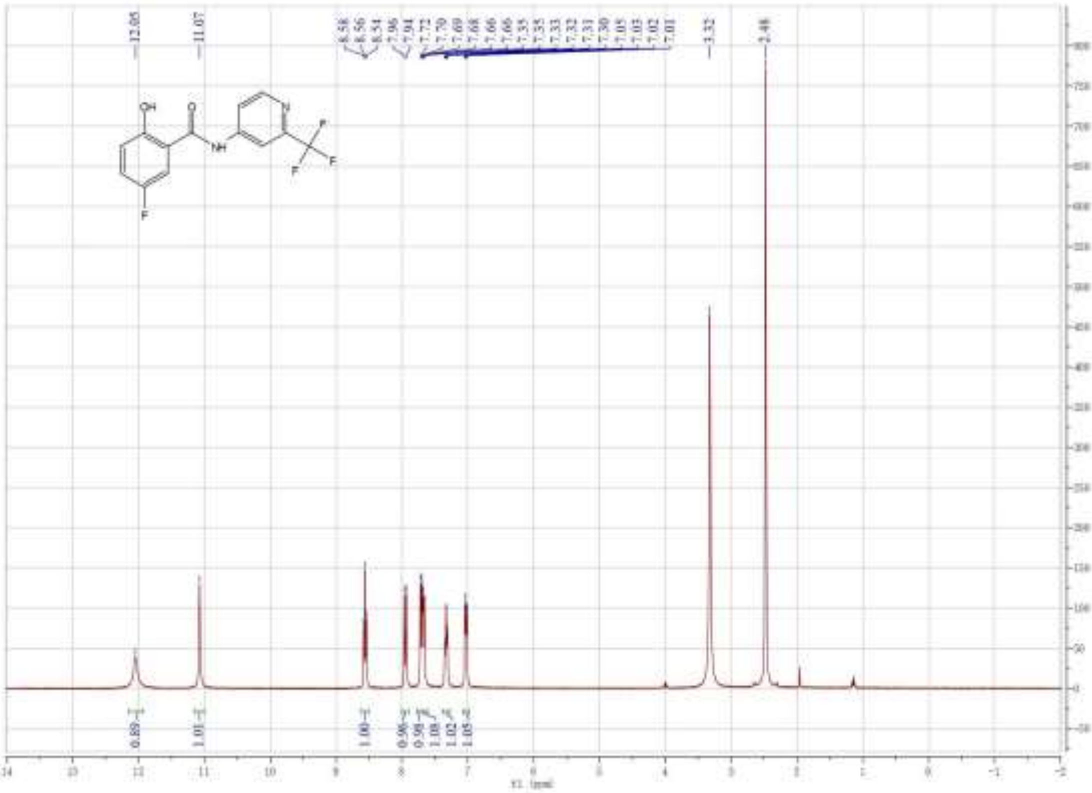

Figure 61. ¹H NMR sprctrogram of Compound B11

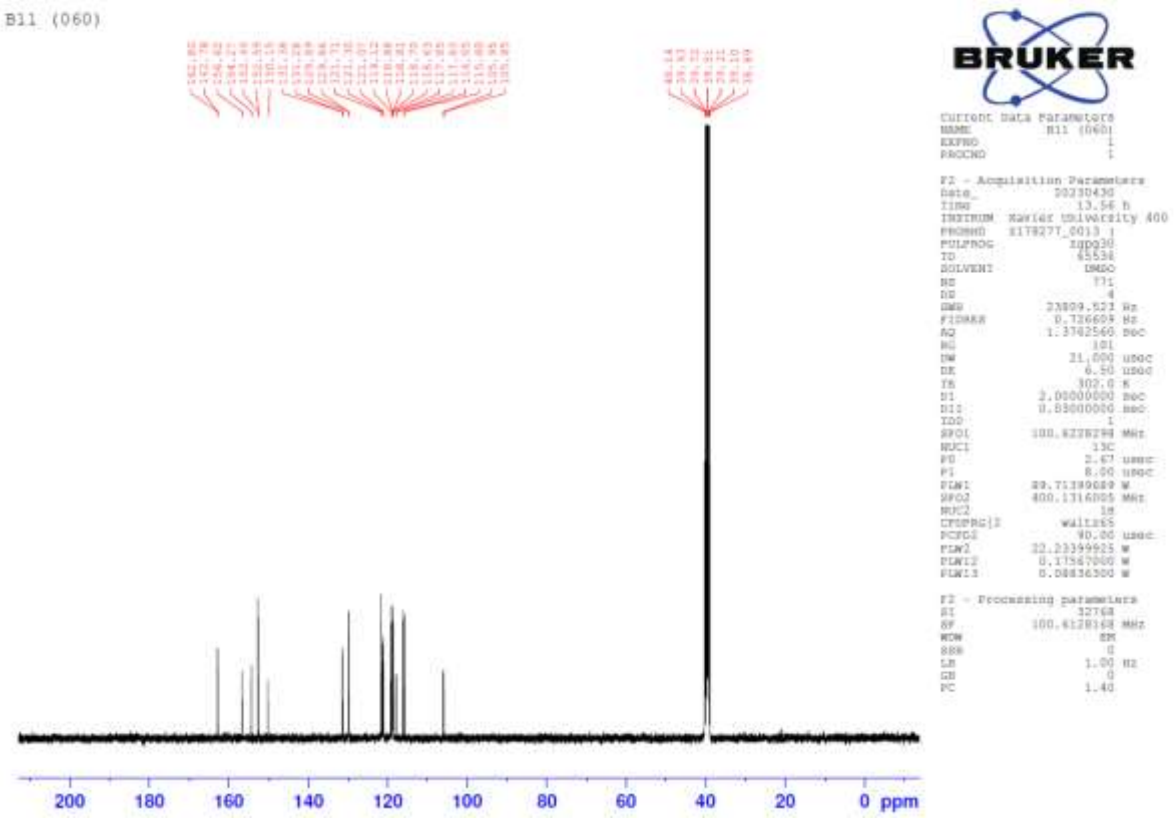

Figure 62. ¹³C NMR spectrogram of Compound B11

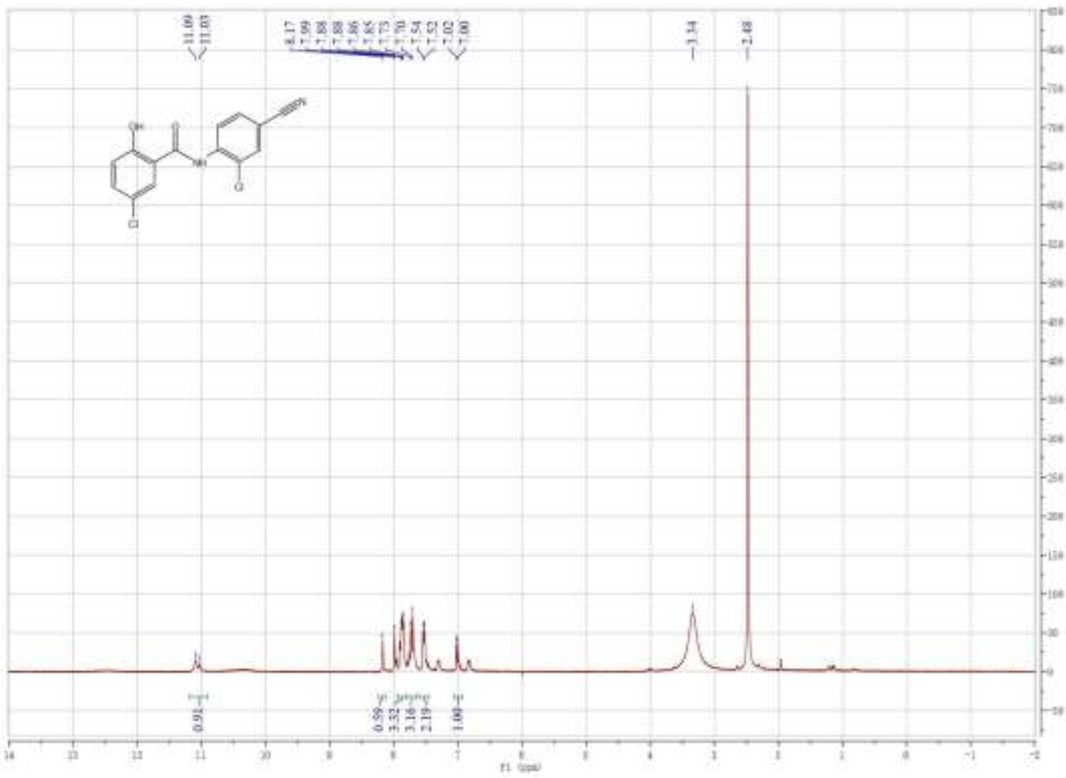

Figure 63. H NMR spectrogram of Compound B12

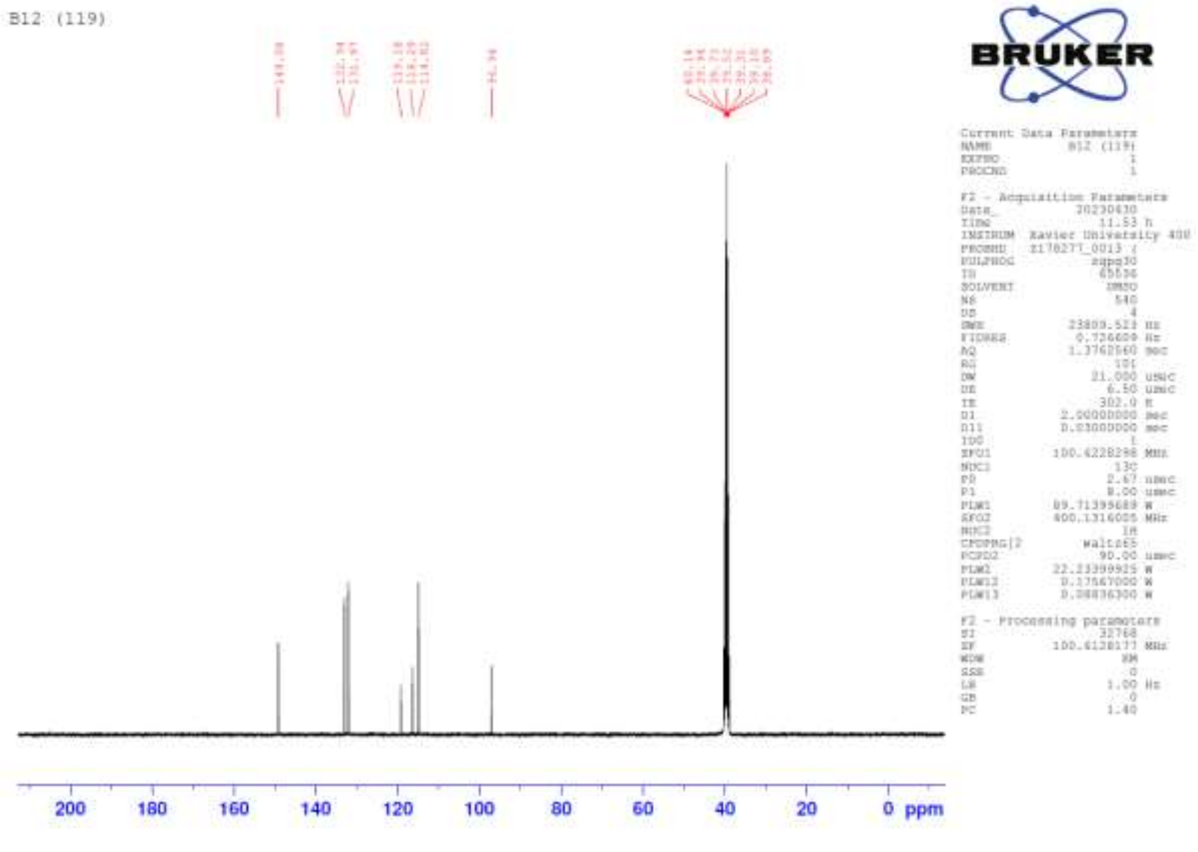

Figure 64. ^{13}C NMR spectrogram of B12

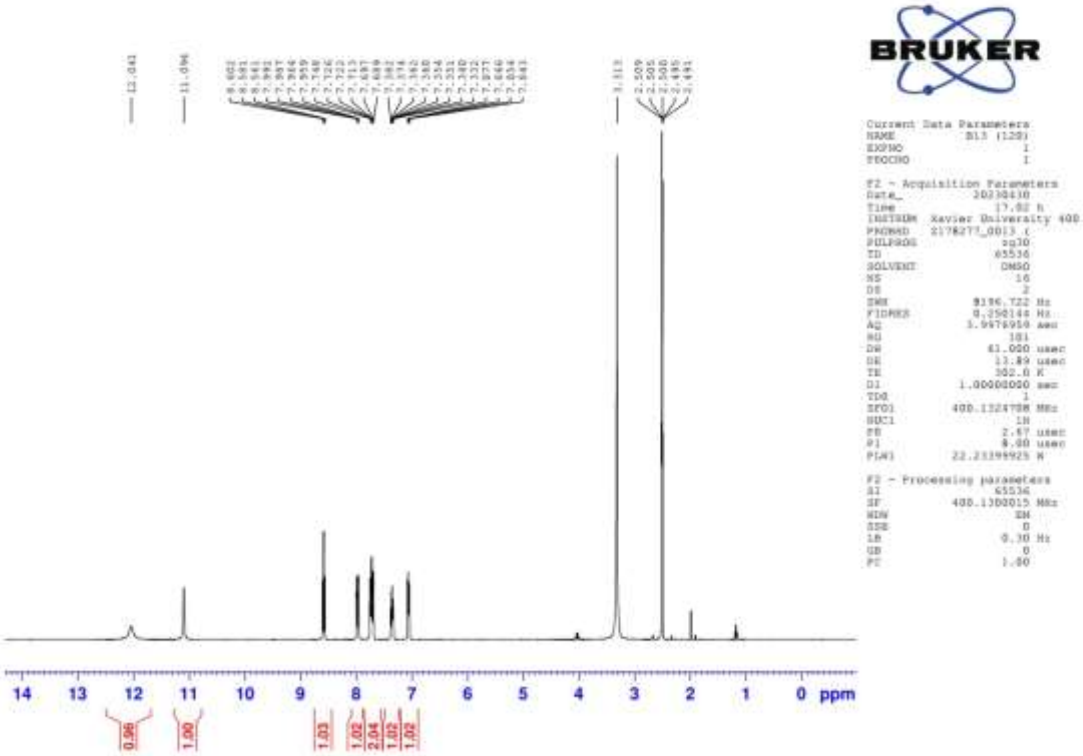

Figure 65. ^{1}H NMR spectrogram of Compound B13

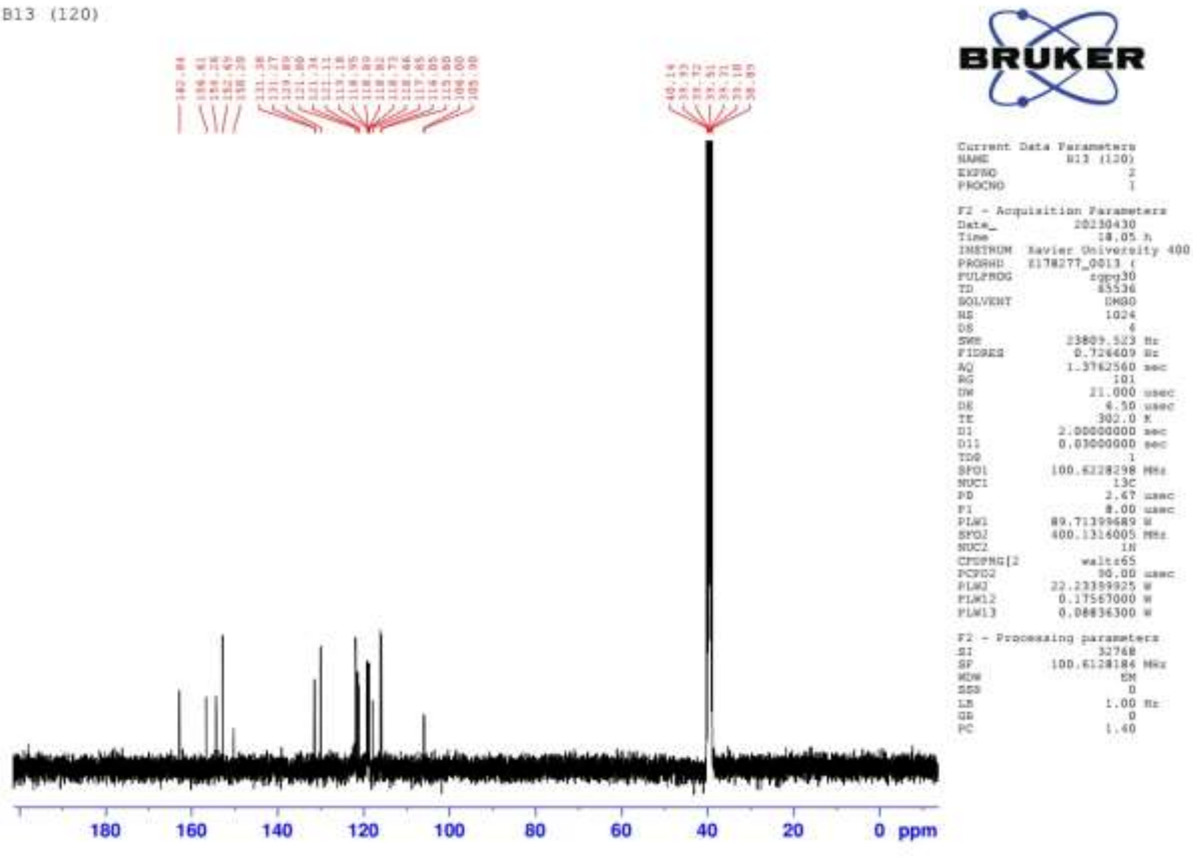

Figure 66. ^{13}C NMR spectrogram of Compound B13

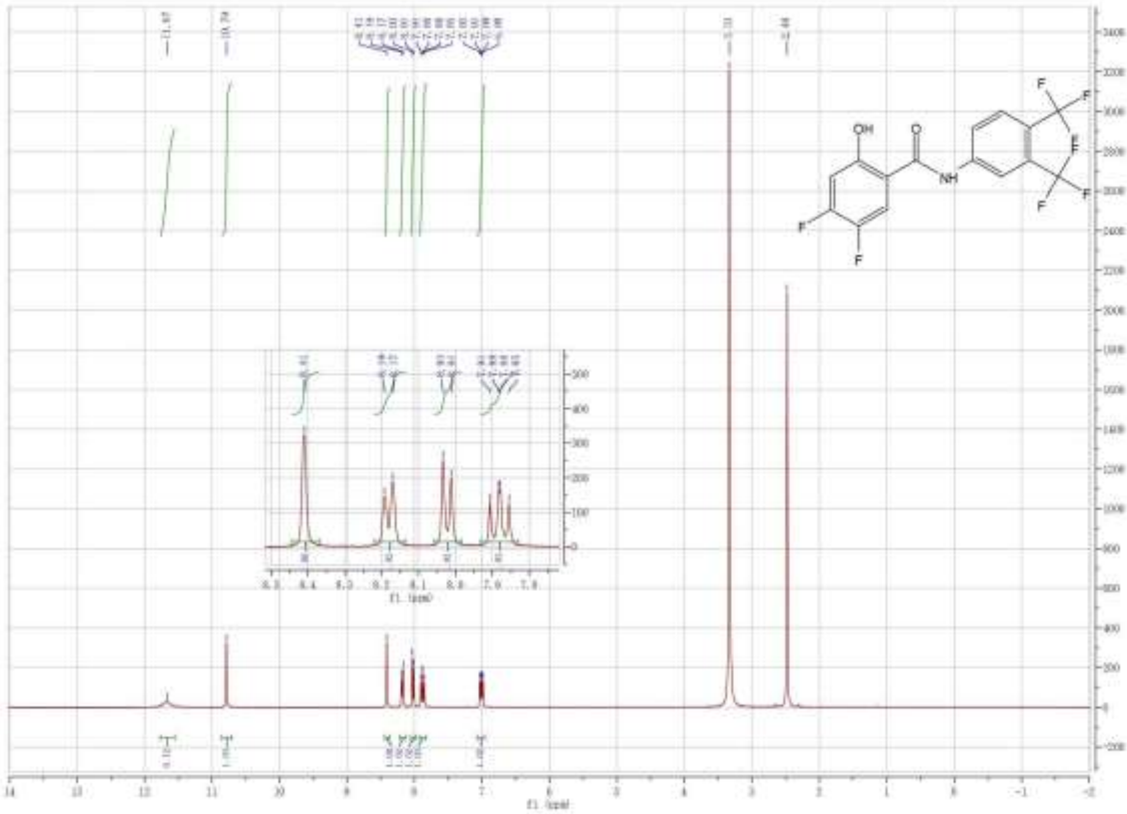

Figure 67. ^{1}H NMR spectrogram of Compound B14

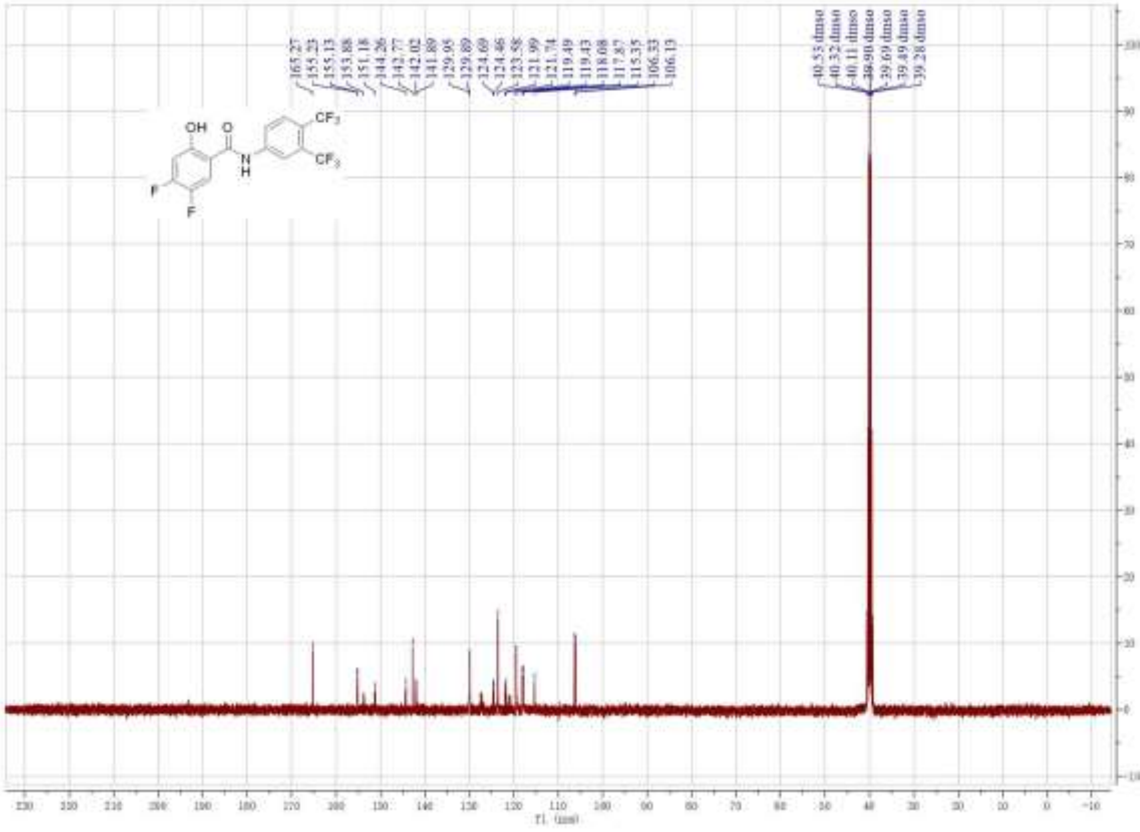

Figure 68. ^{13}C NMR spectrogram of Compound B14

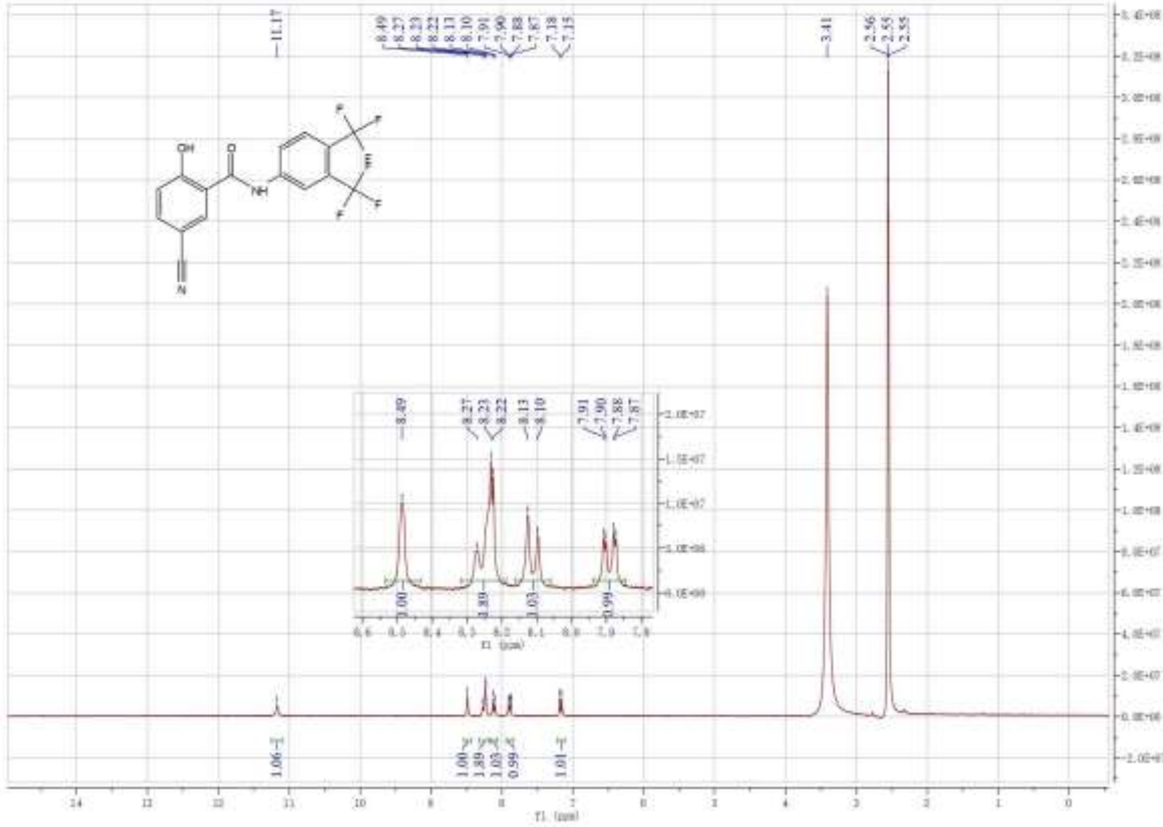

Figure 69. ¹H NMR spectrogram of Compound B15

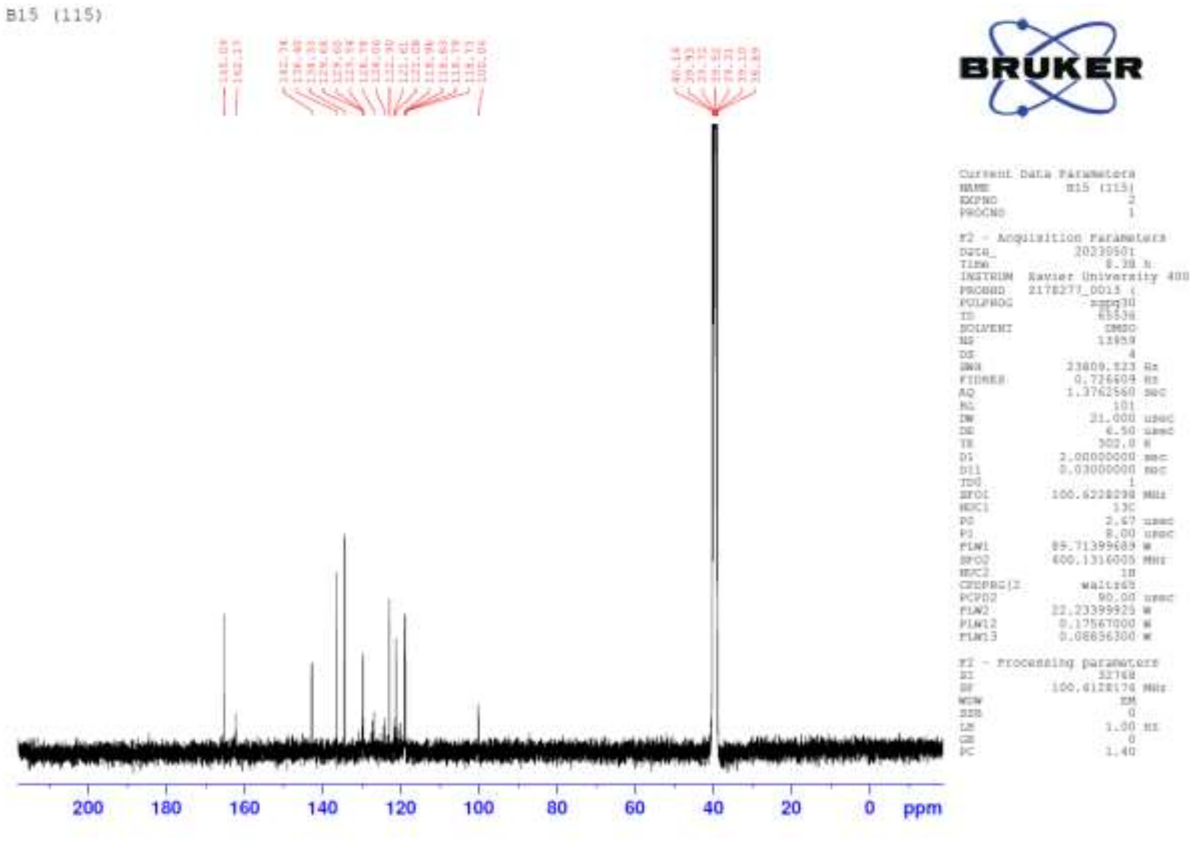

Figure 70. ¹³C NMR spectrogram of Compound B15

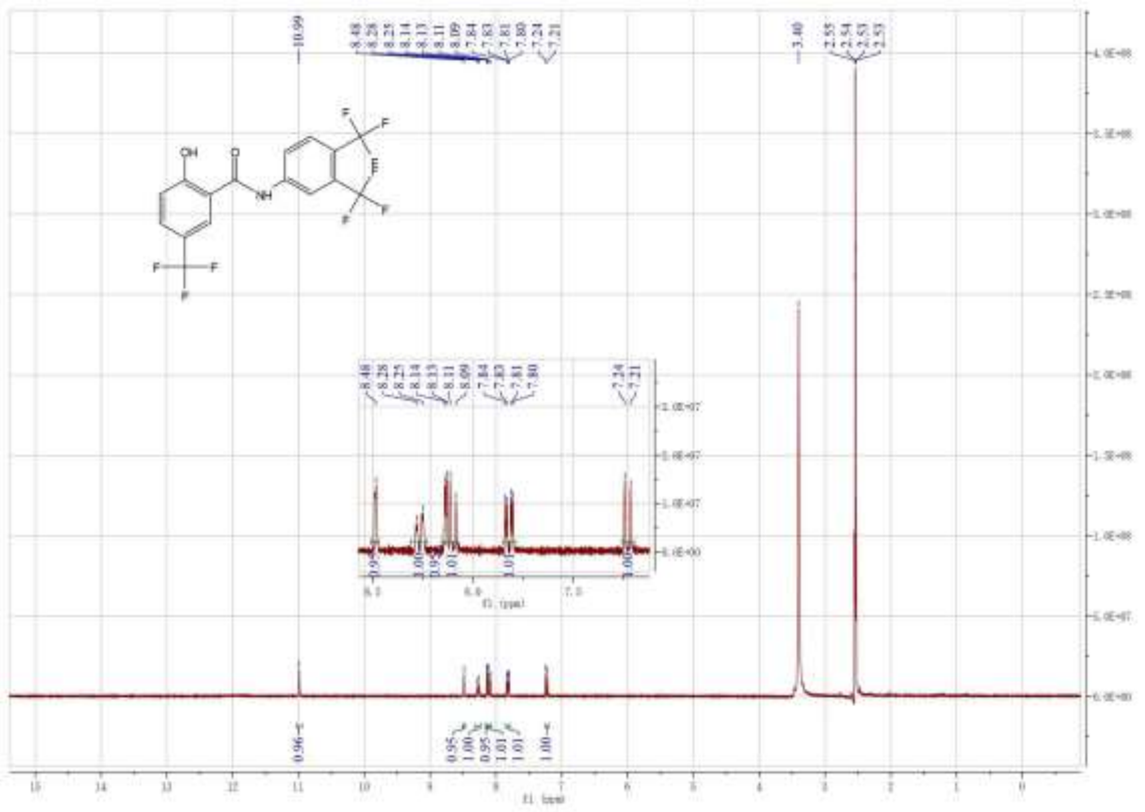

Figure 71. ^{1}H NMR spectrogram of Compound B16

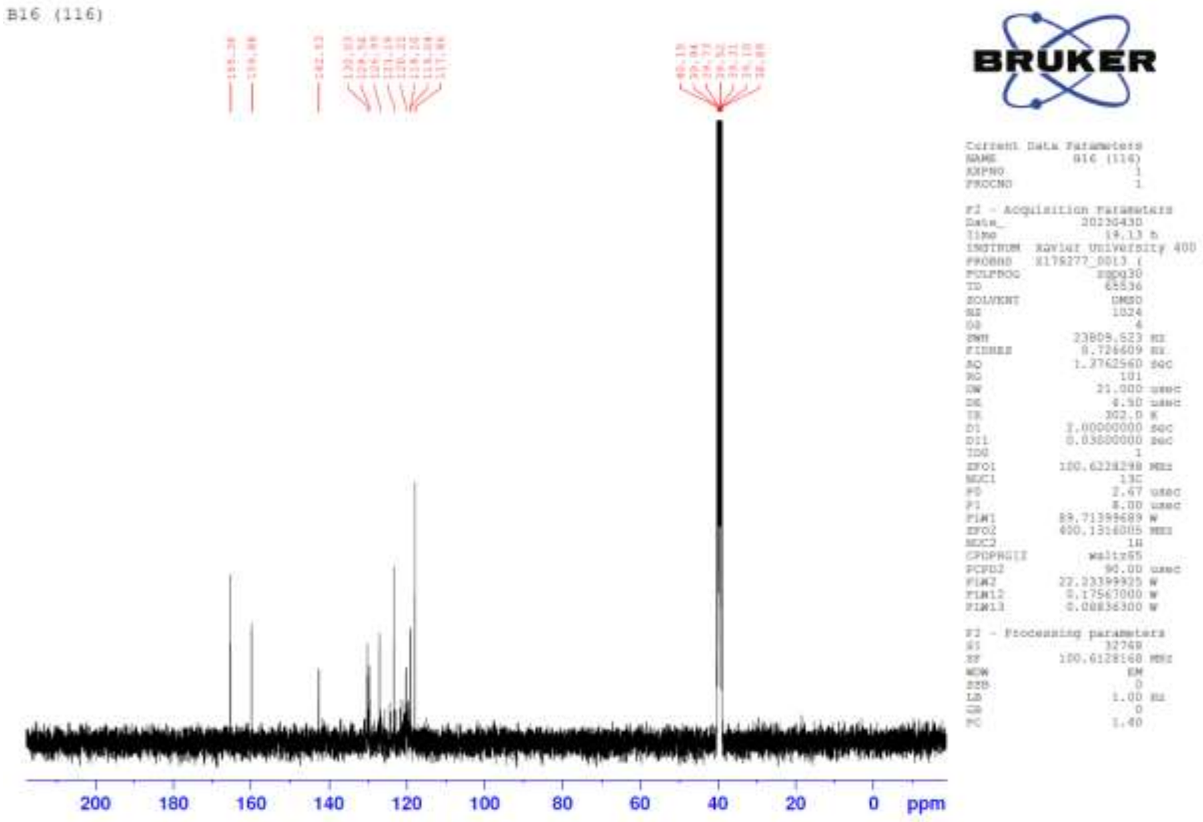

Figure 72. ^{13}C NMR spectrogram of Compound B16

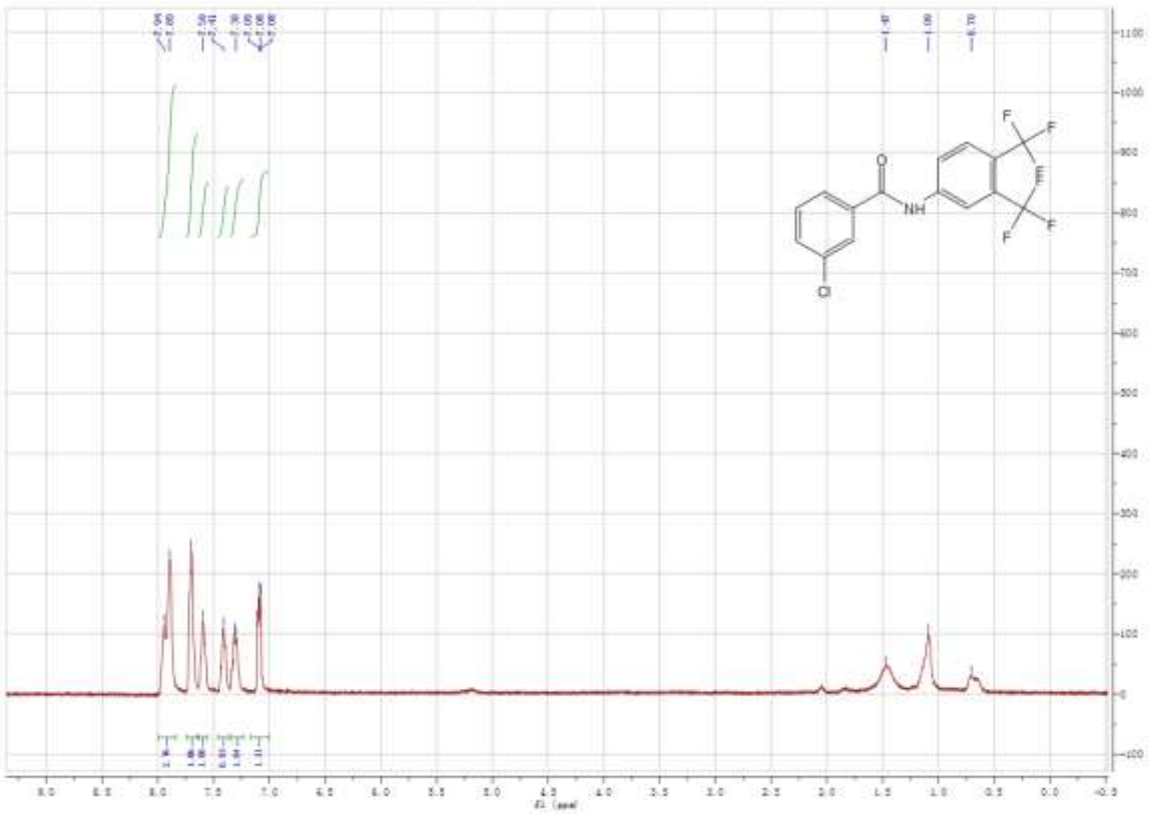

Figure 73. ^{1}H NMR spectrogram of Compound C1

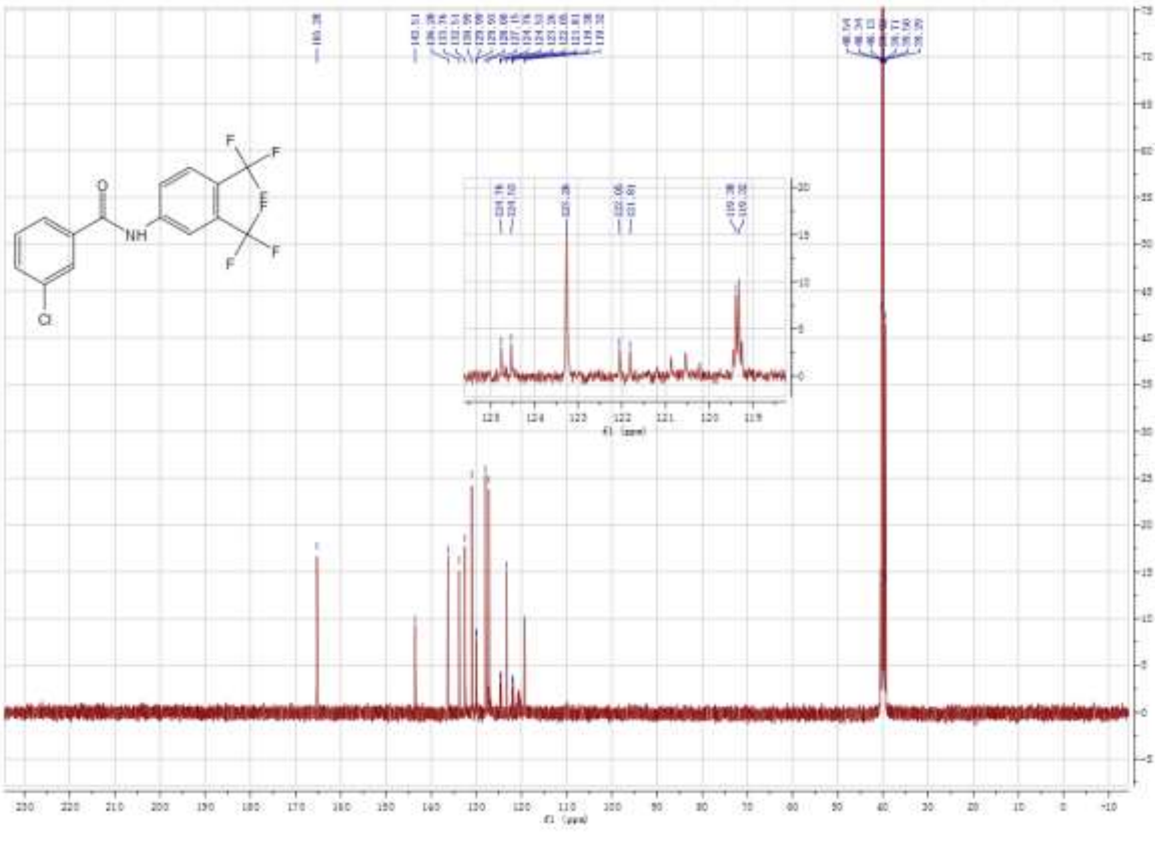

Figure 74. ^{13}C NMR spectrogram of Compound C1

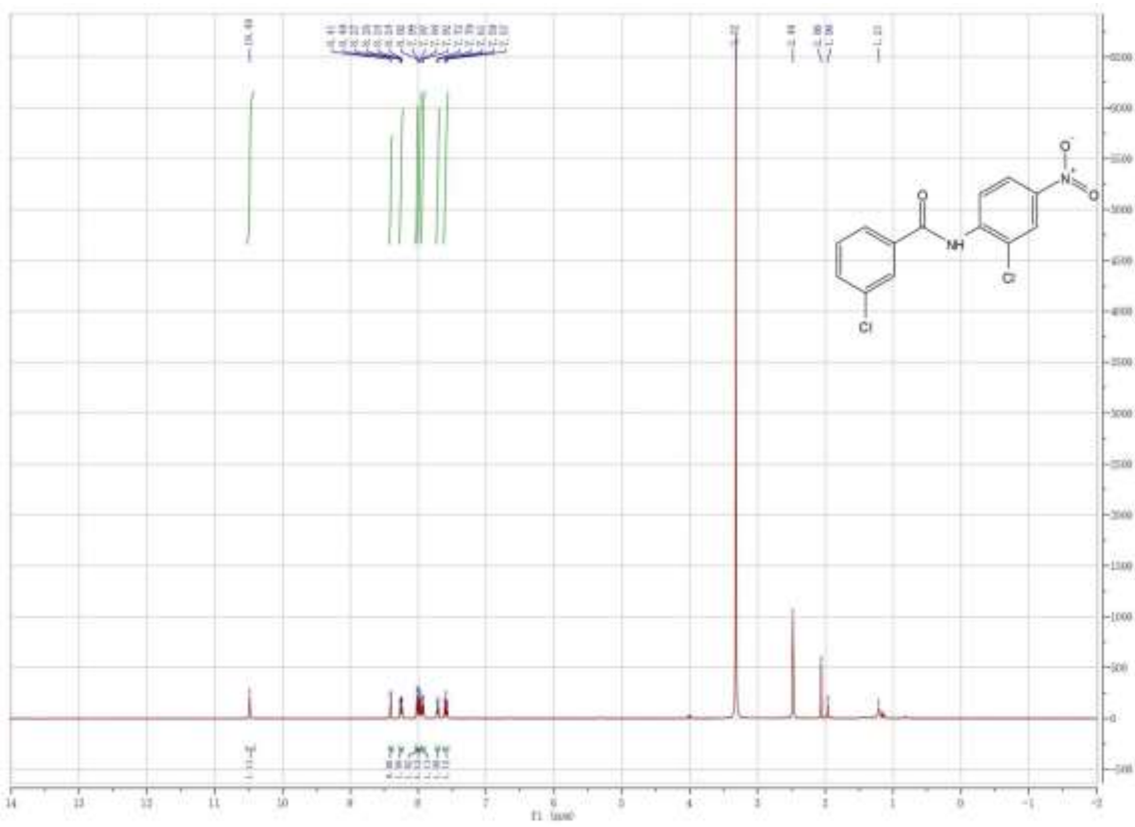

Figure 75. ^{1}H NMR spectrogram of Compound C2

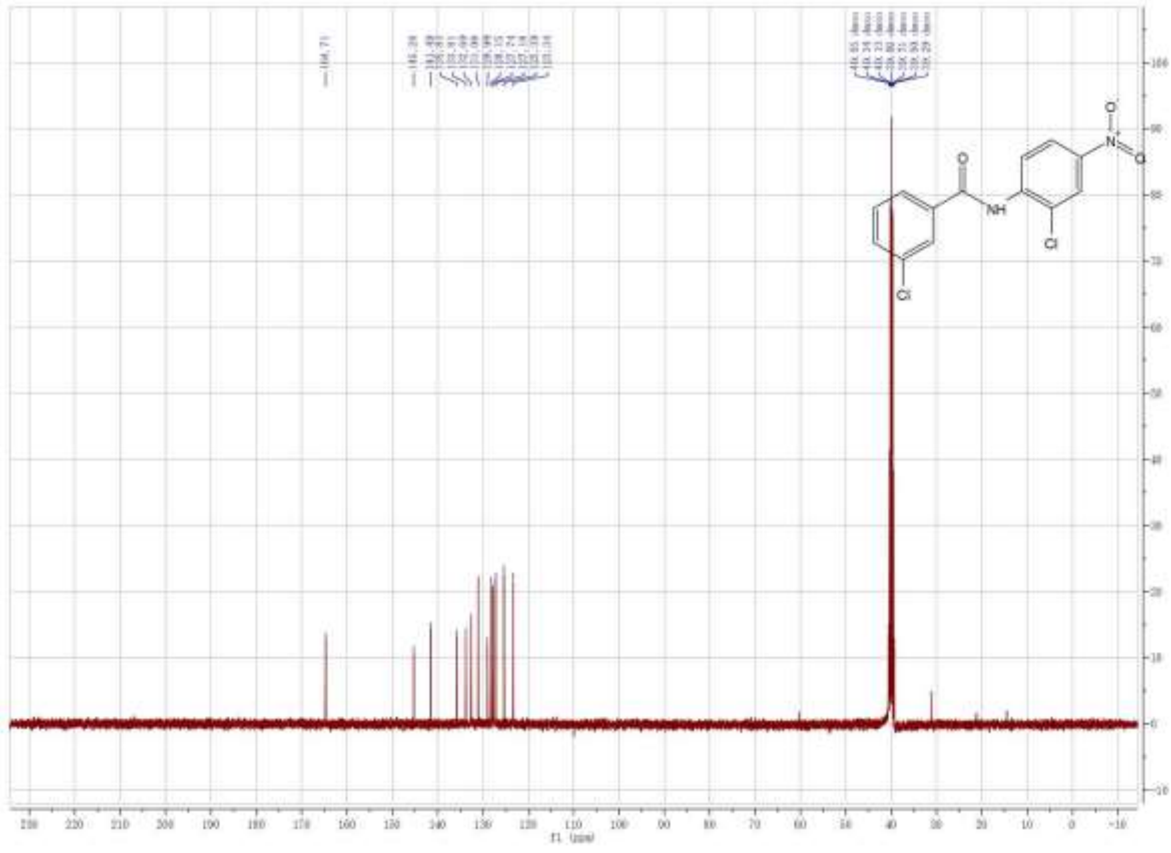

Figure 76. ^{13}C NMR spectrogram of Compound C2

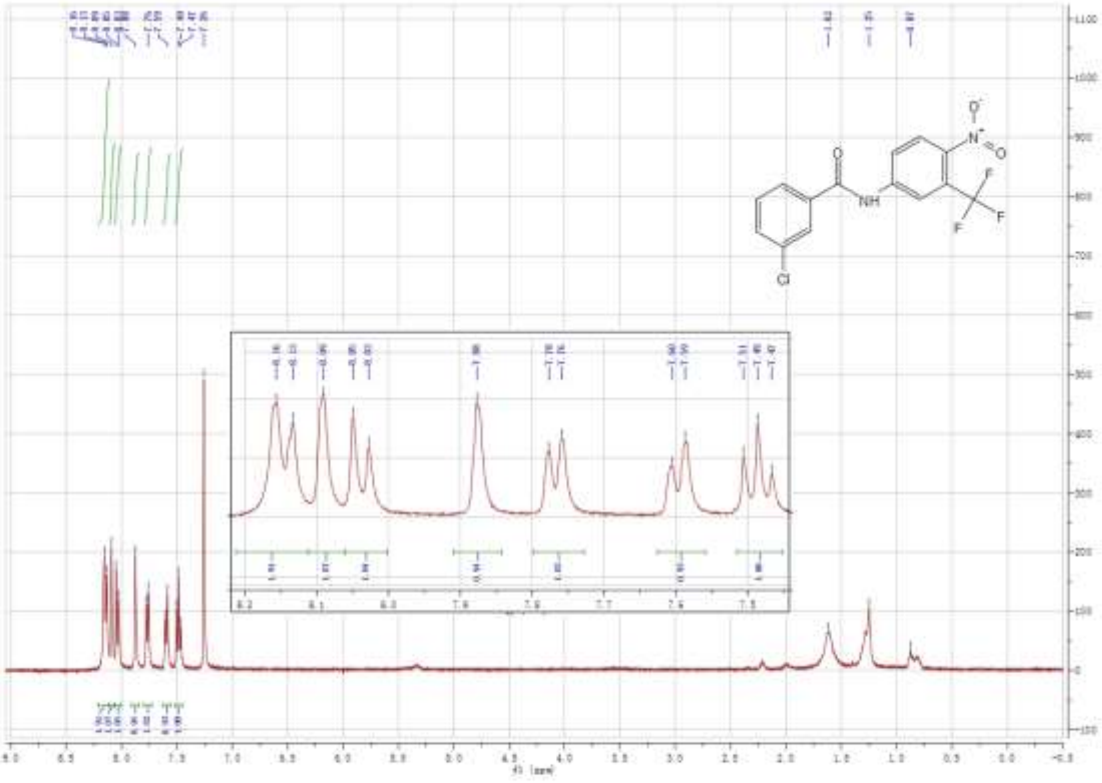

Figure 77. ^{1}H NMR spectrogram of Compound C3

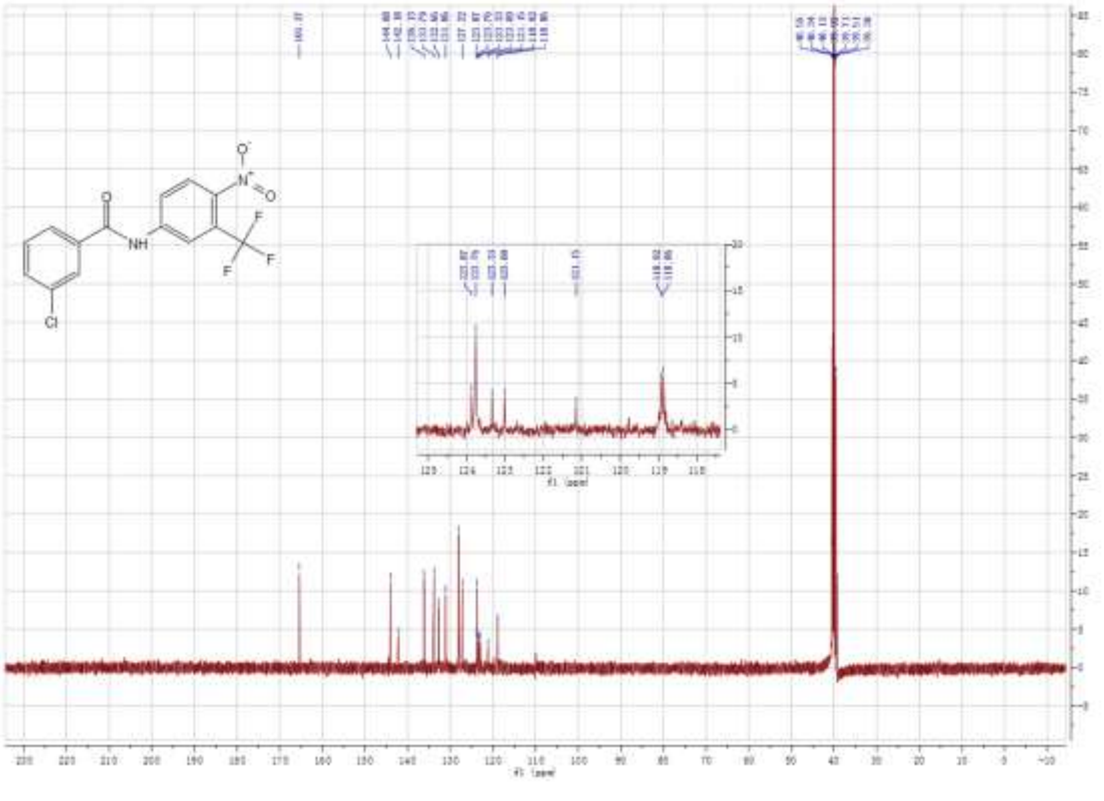

Figure 78. ^{13}C NMR sprctrogram of Compound C3

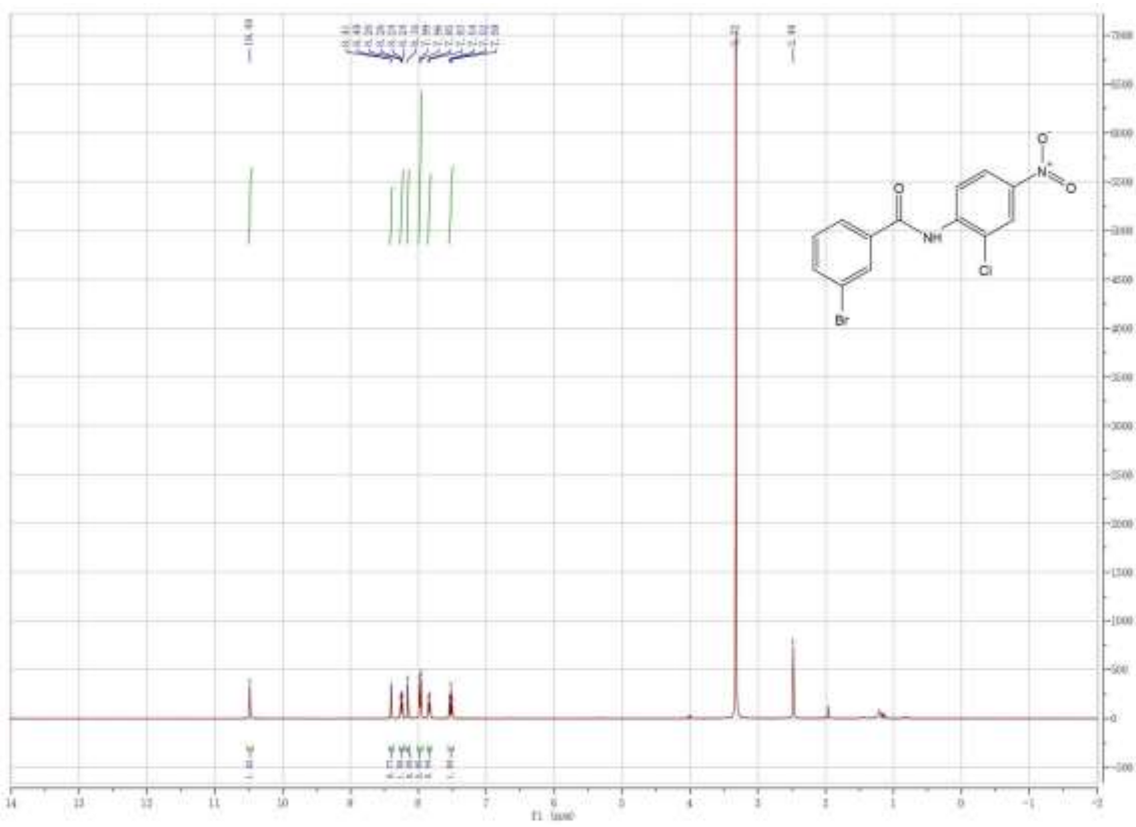

Figure 79. ¹H NMR spectrogram of Compound C4

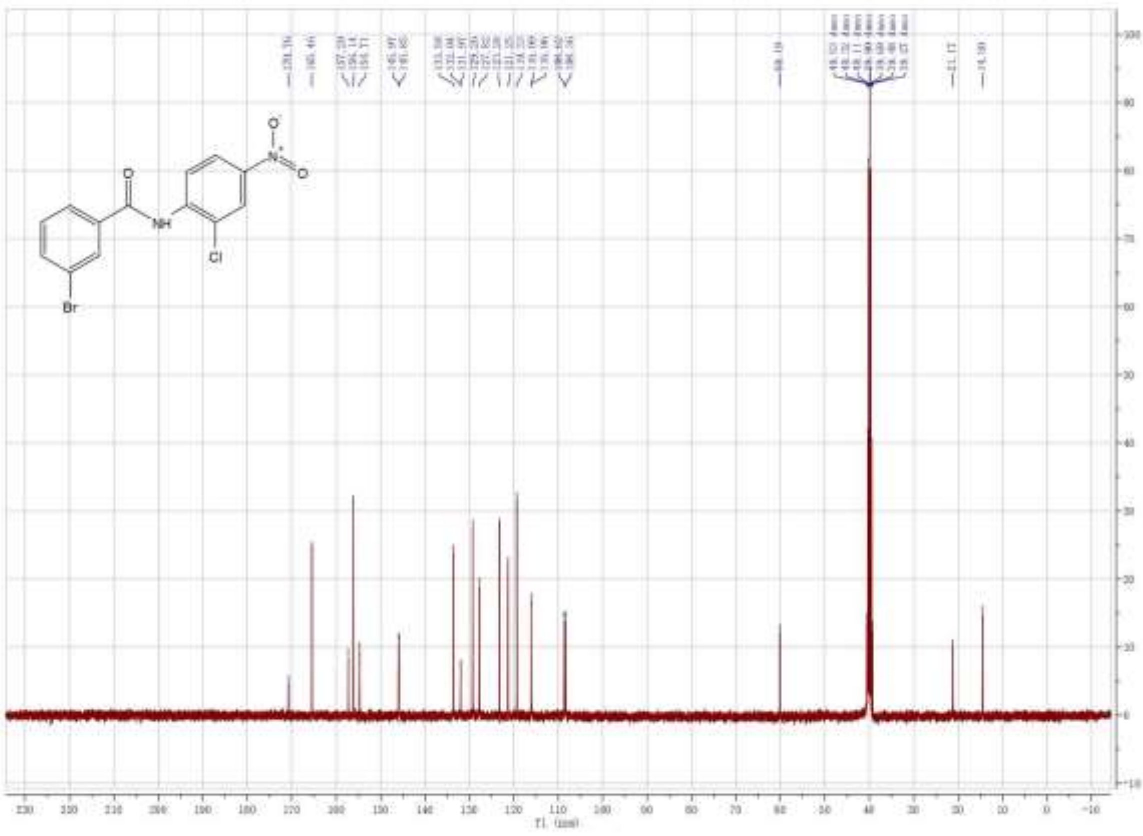

Figure 80. ¹³C NMR spectrogram of Compound C4

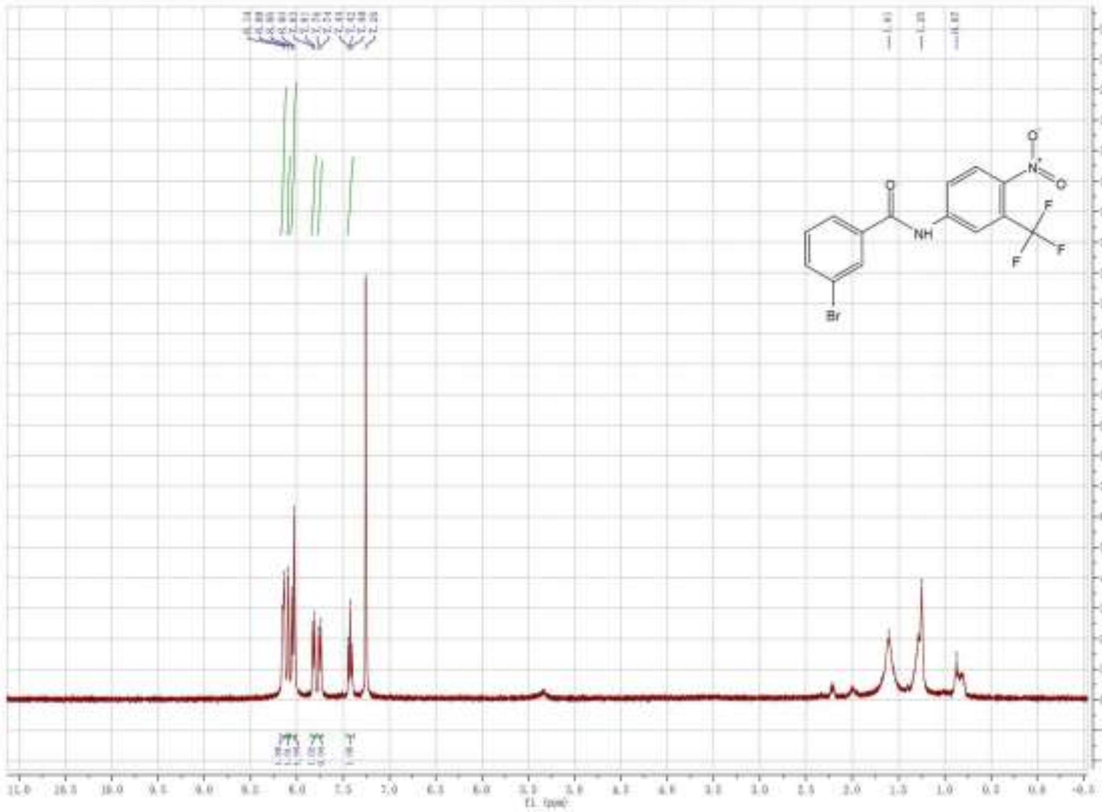

Figure 81. ^{1}H NMR spectrogram of Compound C5

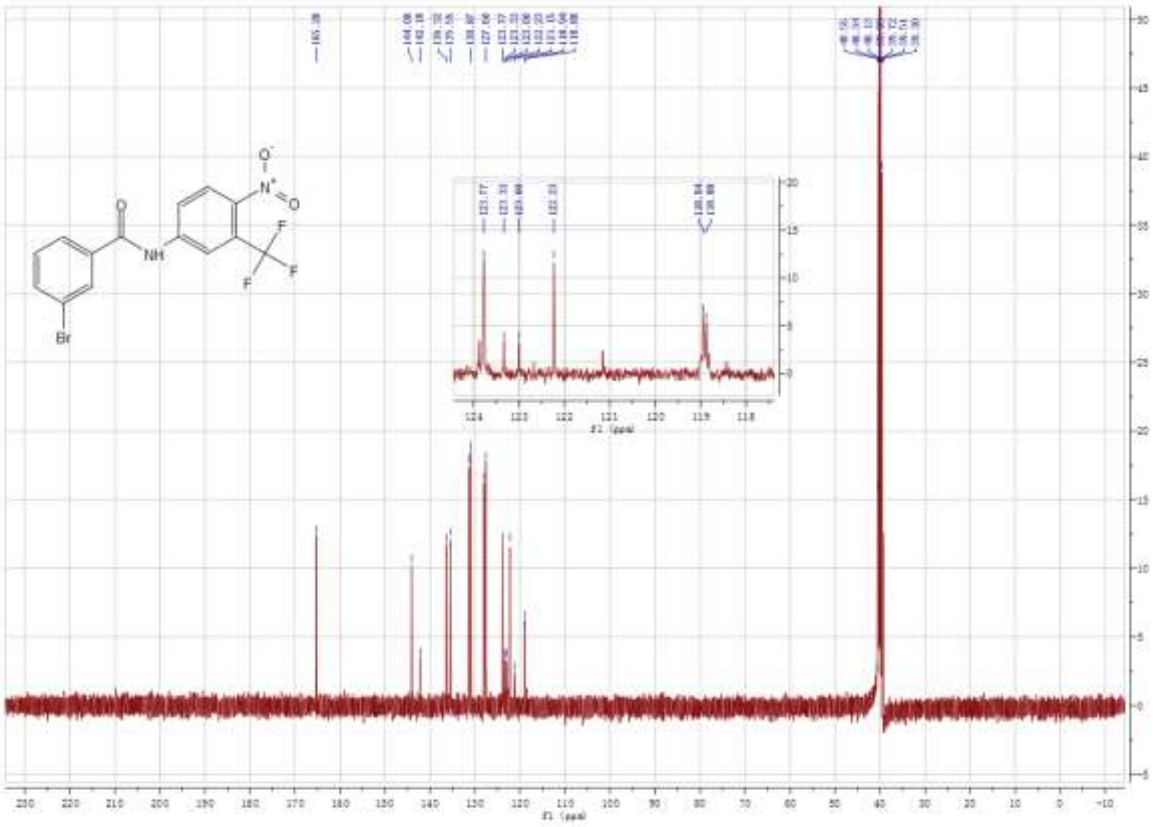

Figure 82. ^{13}C NMR spectrogram of Compound C5

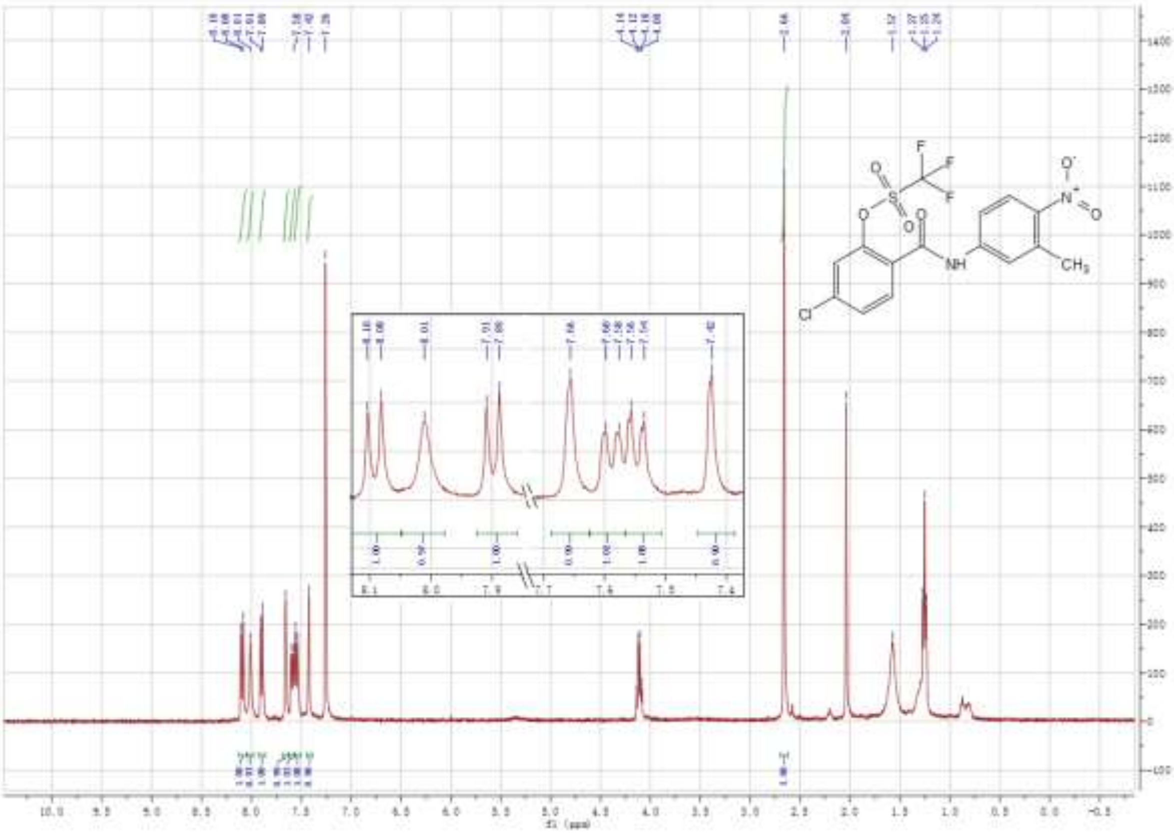

Figure 83. ^{1}H NMR spectrogram of Compound C6

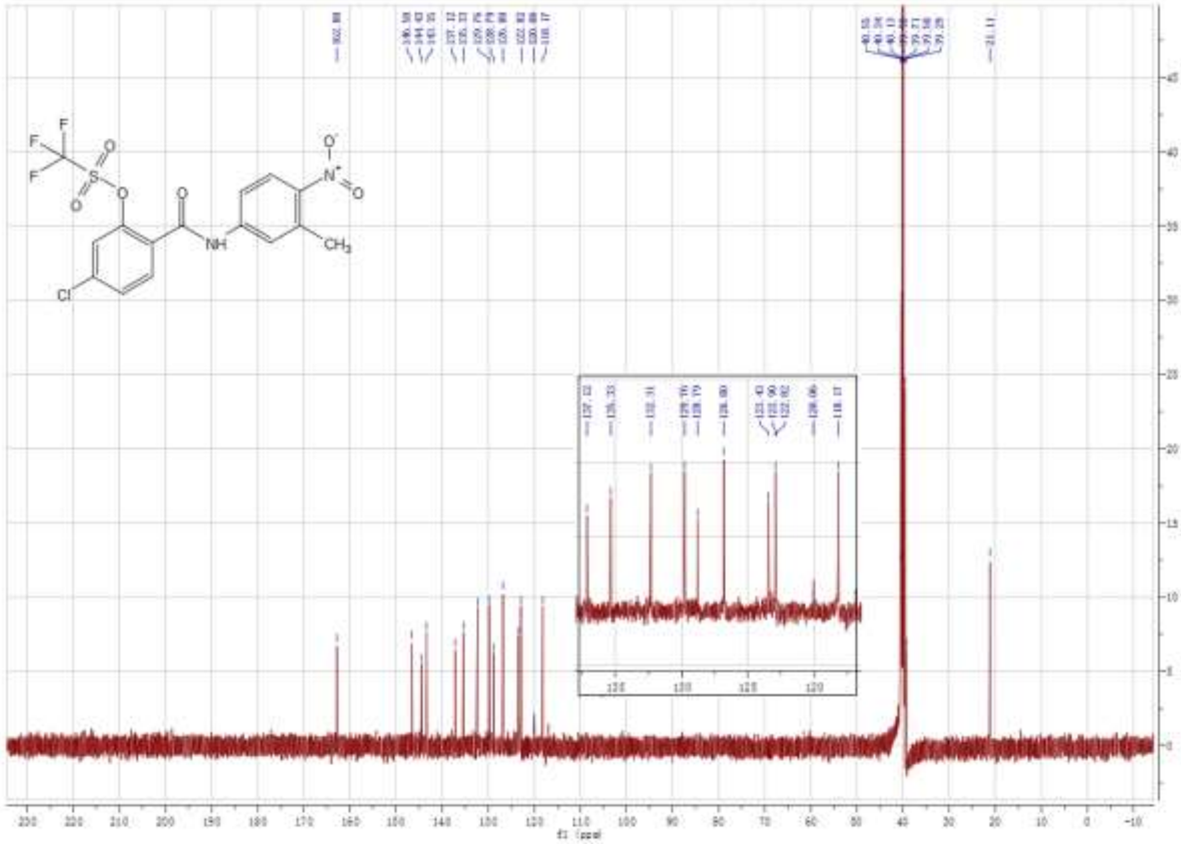

Figure 84. ^{13}C NMR spectrogram of Compound C6

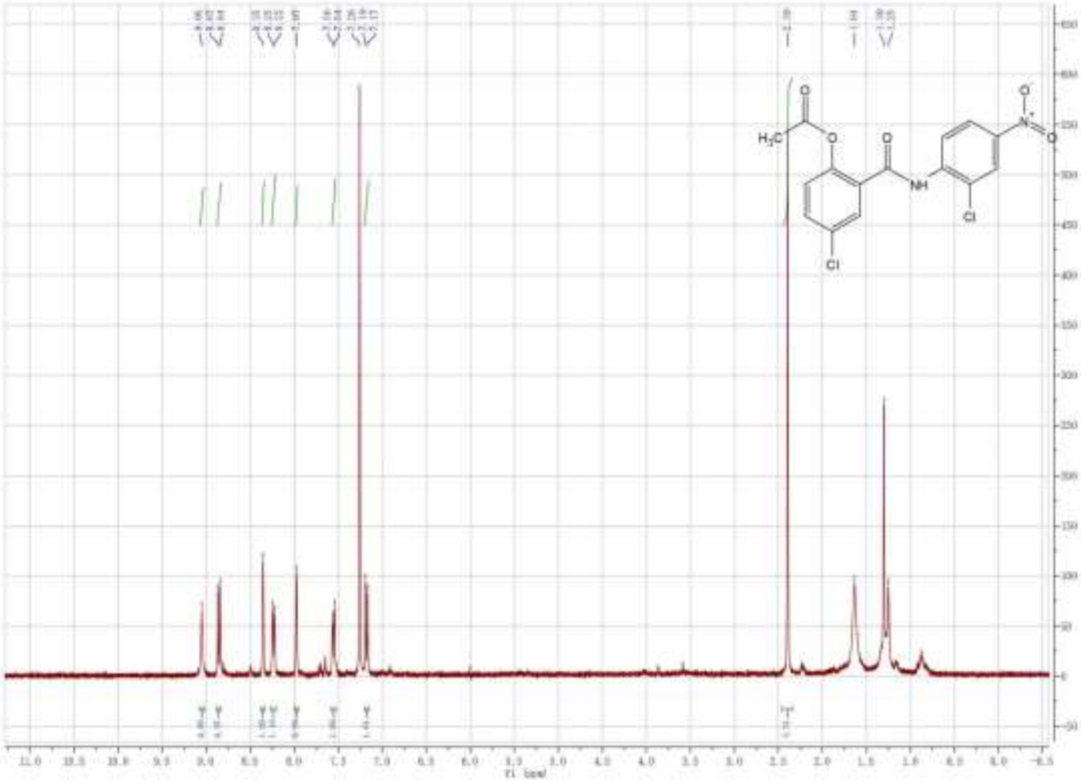

Figure 85. ^{1}H NMR spectrogram of Compound C7

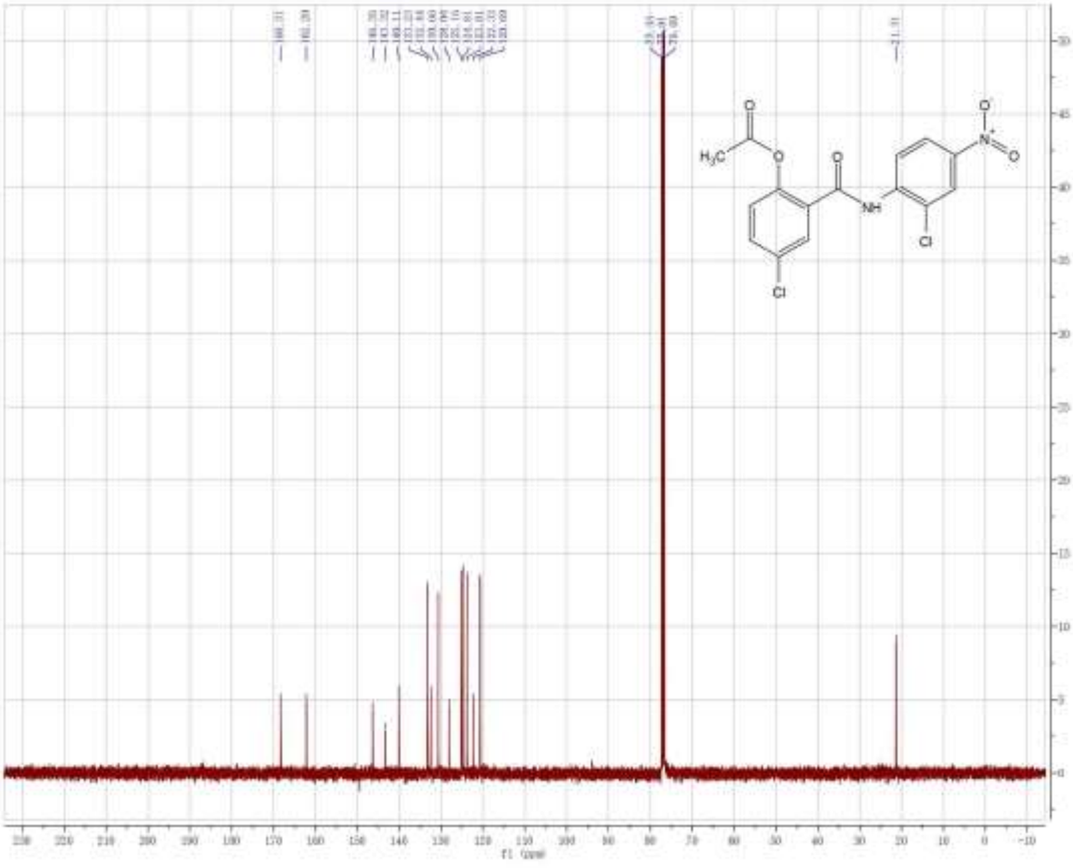

Figure 86. ^{13}C NMR spectrogram of Compound C7

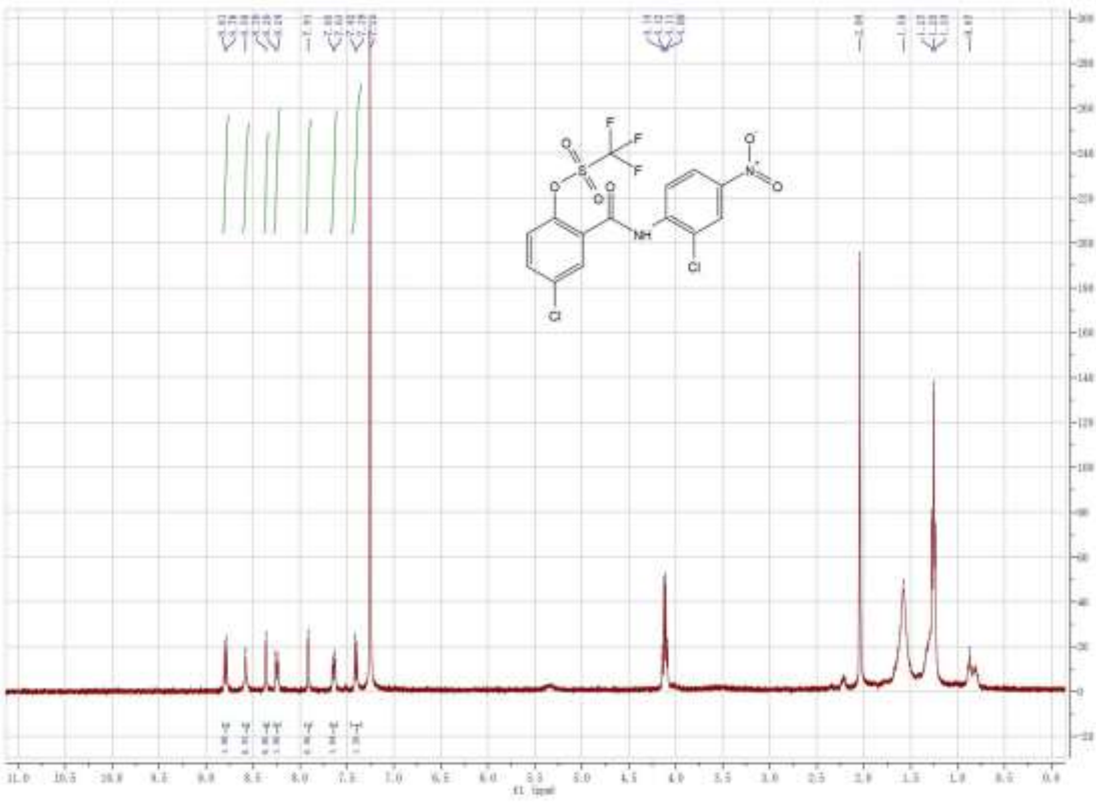

Figure 87. ^{1}H NMR spectrogram of Compound C8

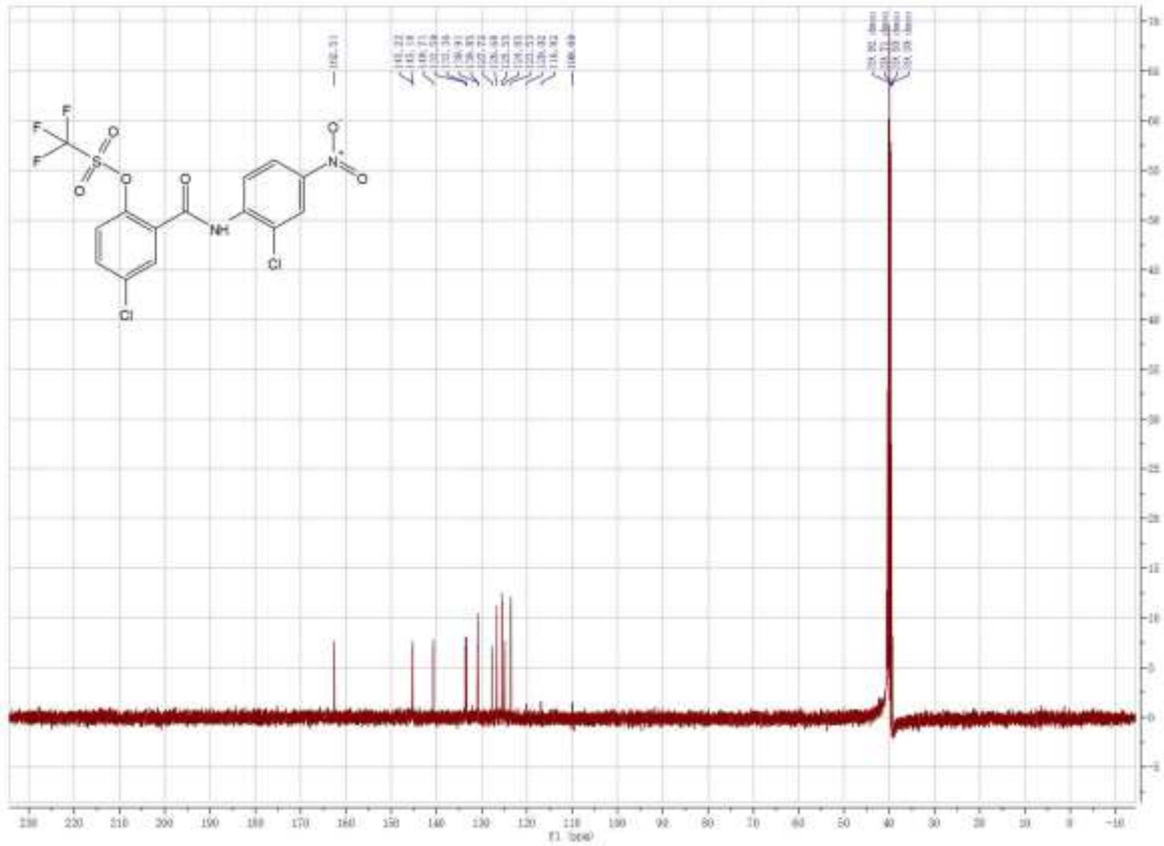

Figure 88. ^{13}C NMR spectrogram of Compound C8

5. HRMS Spectrum of Target Compounds

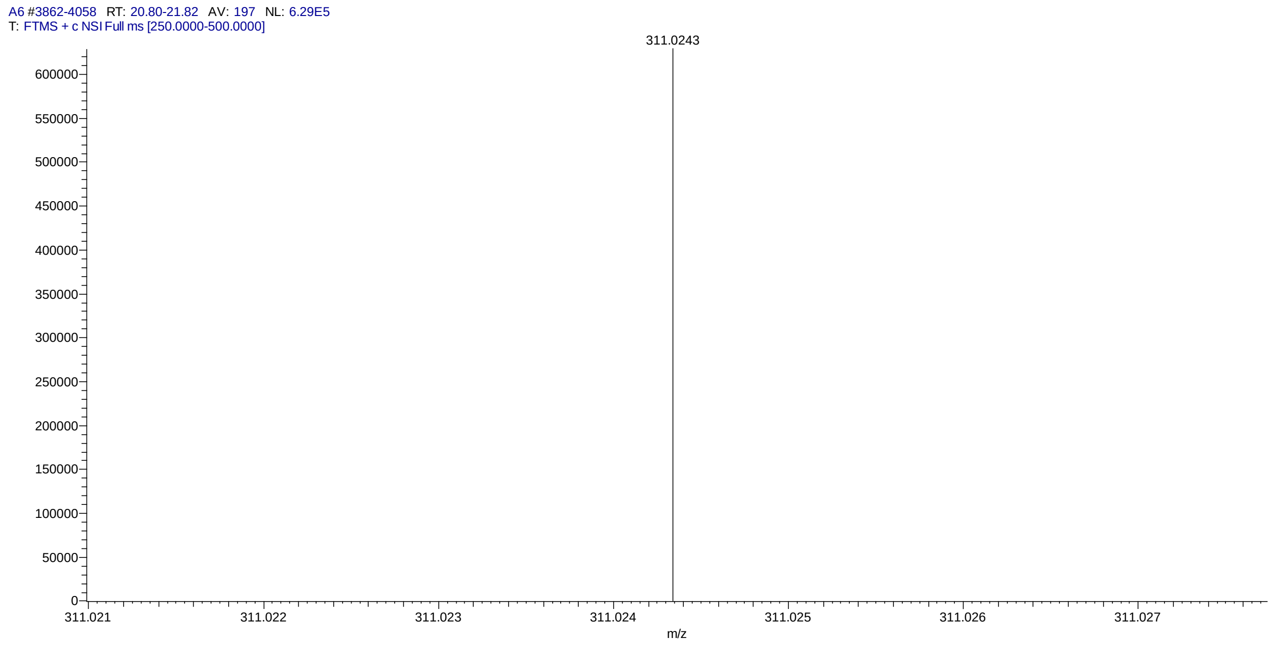

Figure 89. HRMS of Compound A1

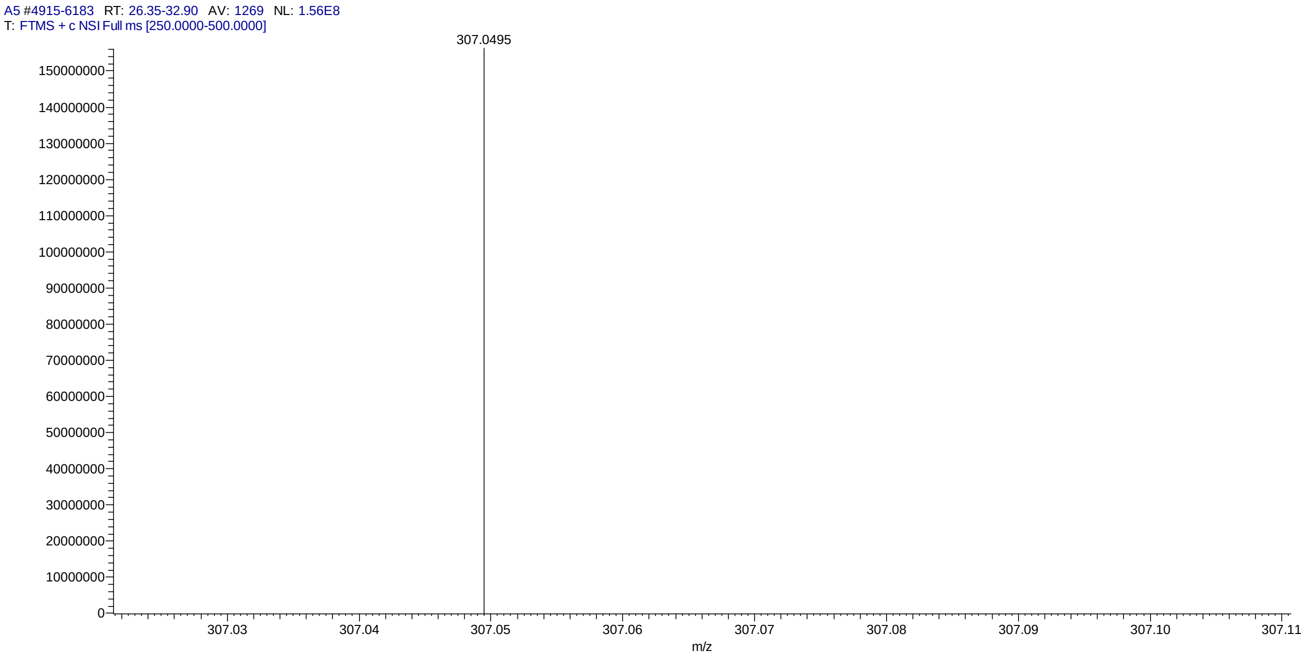

Figure 90.HRMS of Compound A2

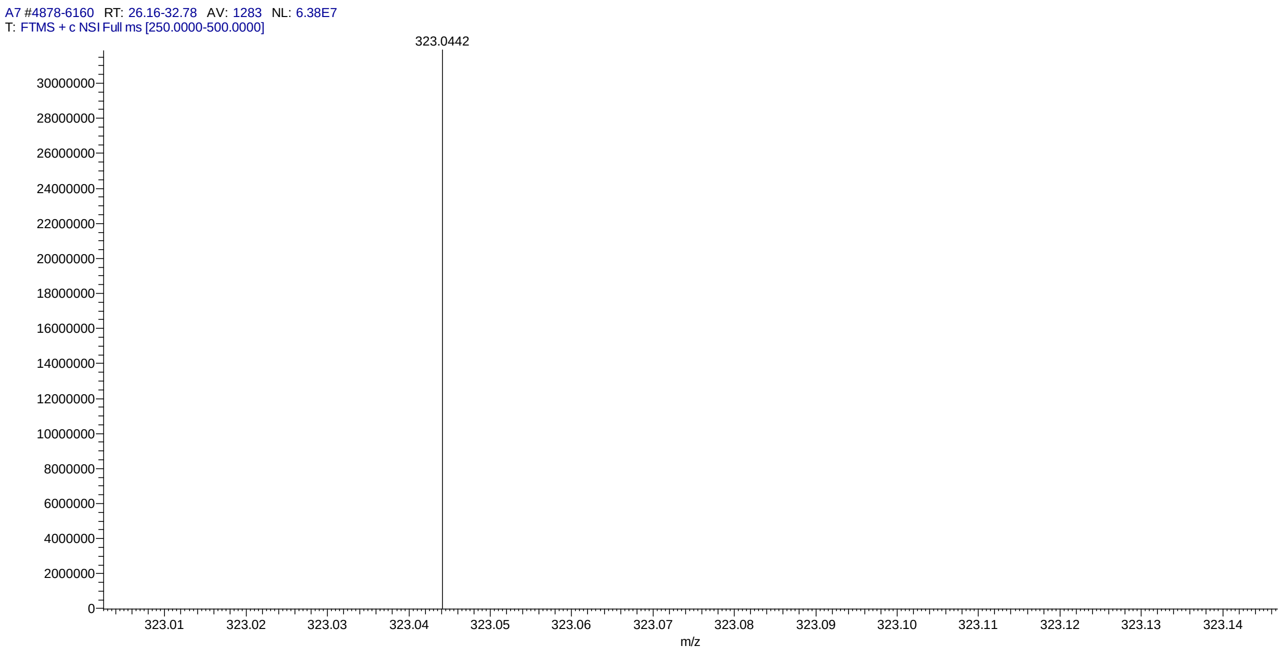

Figure 91. HRMS of Compound A3

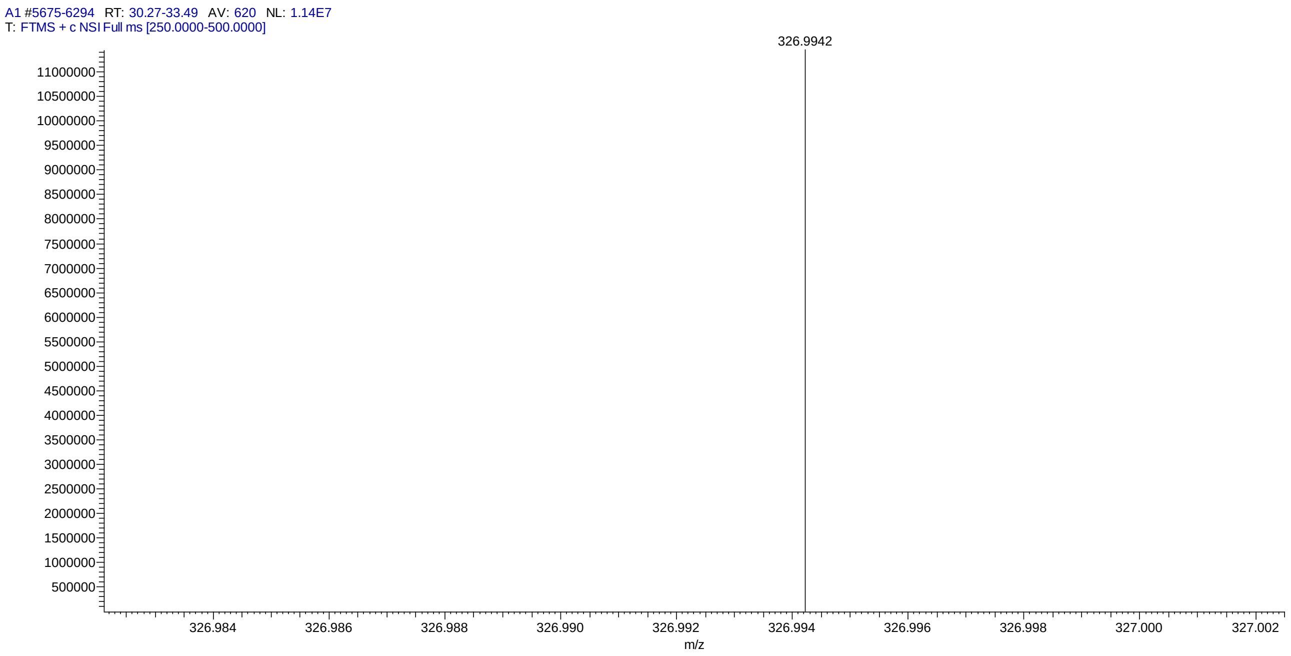

Figure 92. HRMS of Compound A4

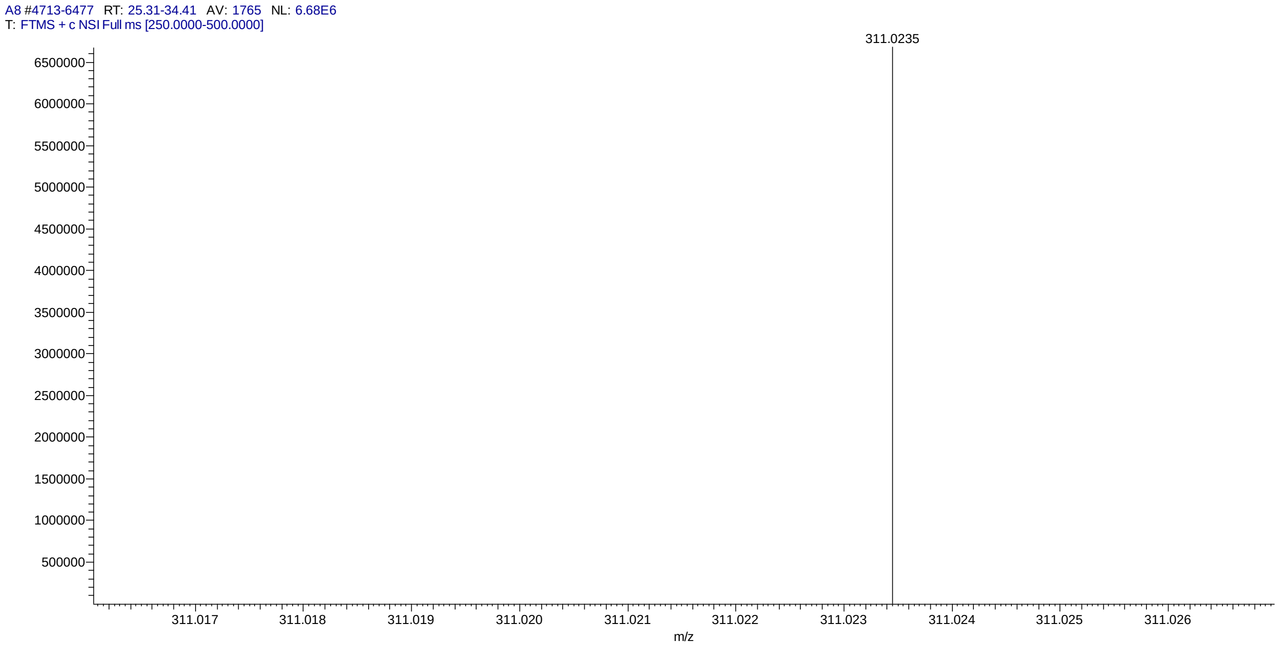

Figure 93. HRMS of Compound A5

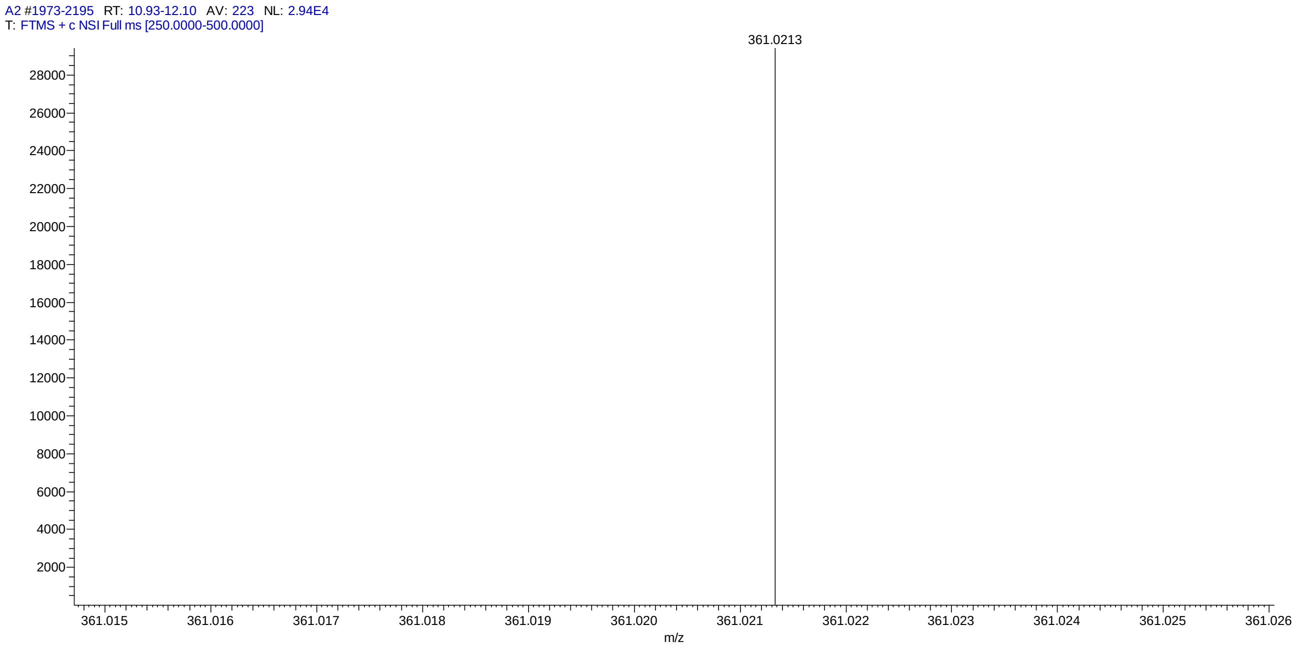

Figure 94. HRMS of Compound A6

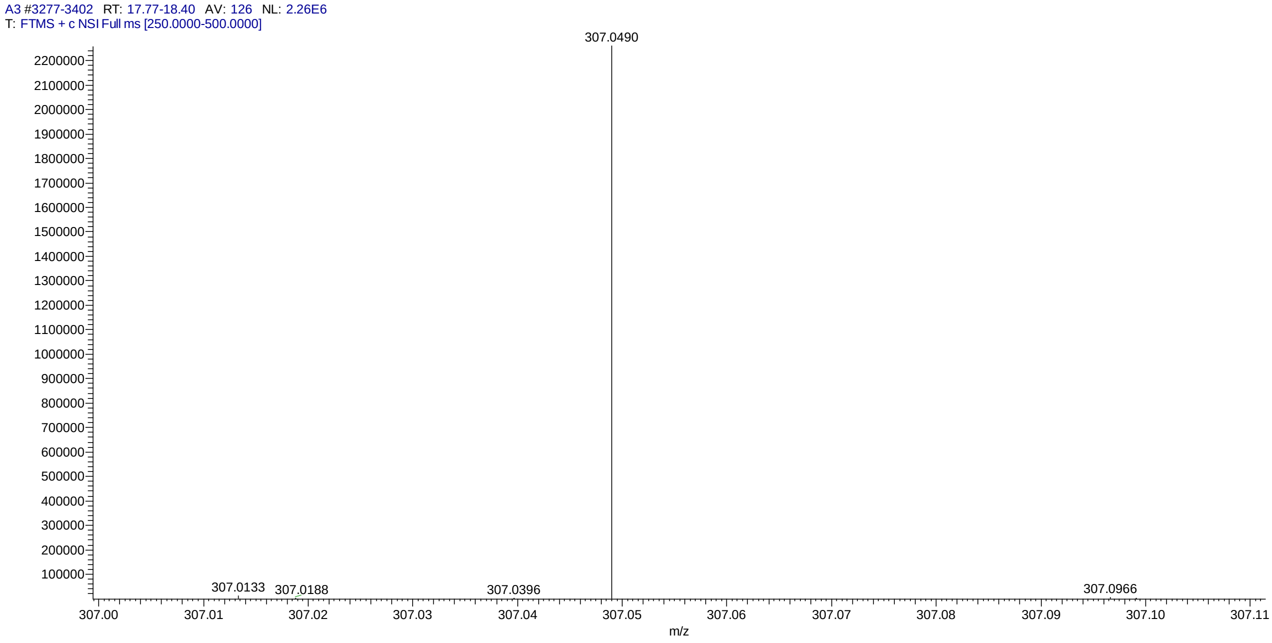

Figure 95. HRMS of Compound A7

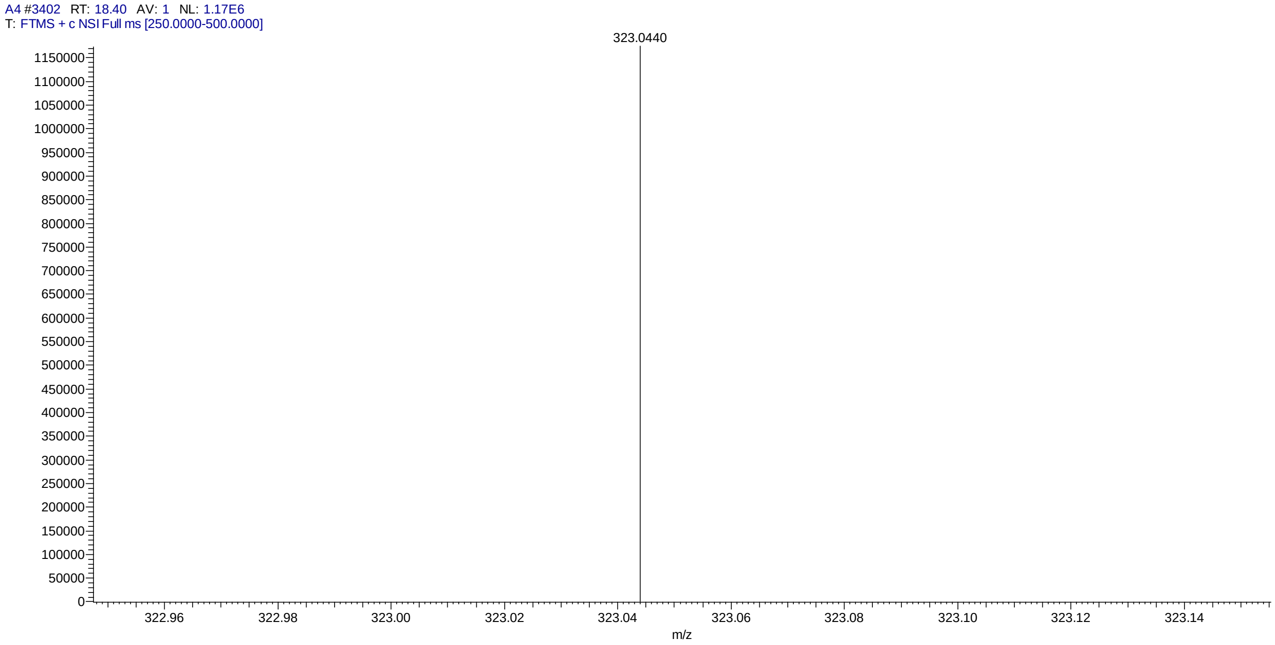

Figure 96. HRMS of Compound A8

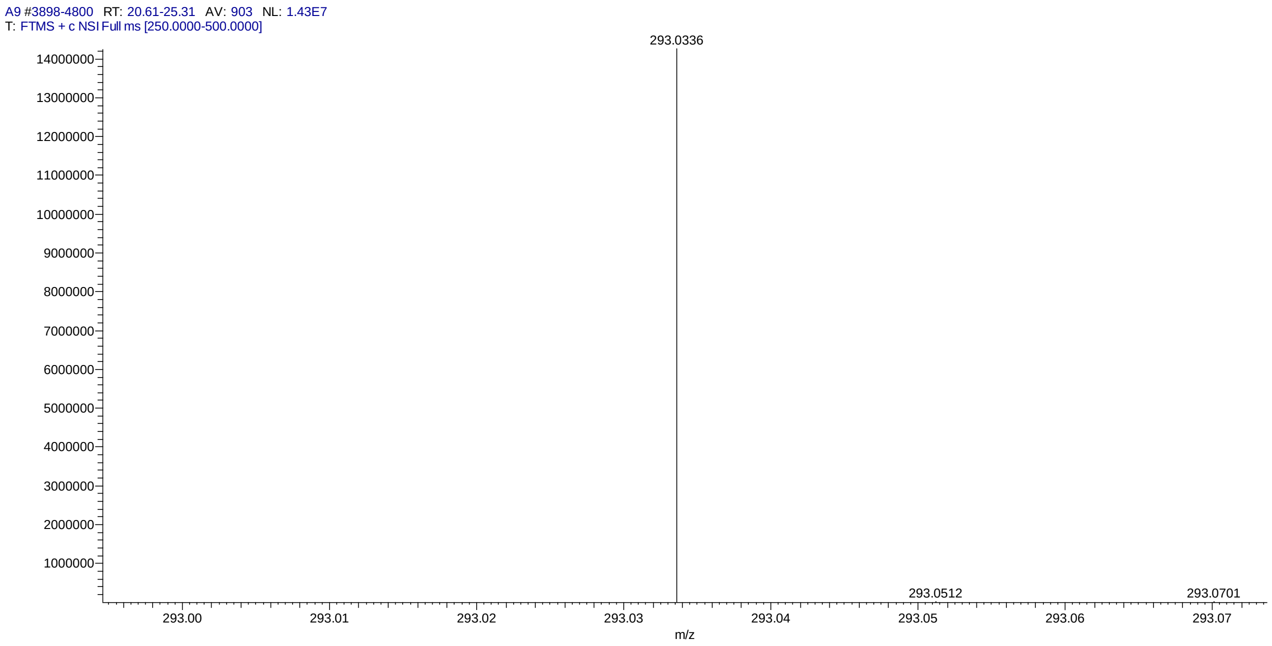

Figure 97. HRMS of Compound A9

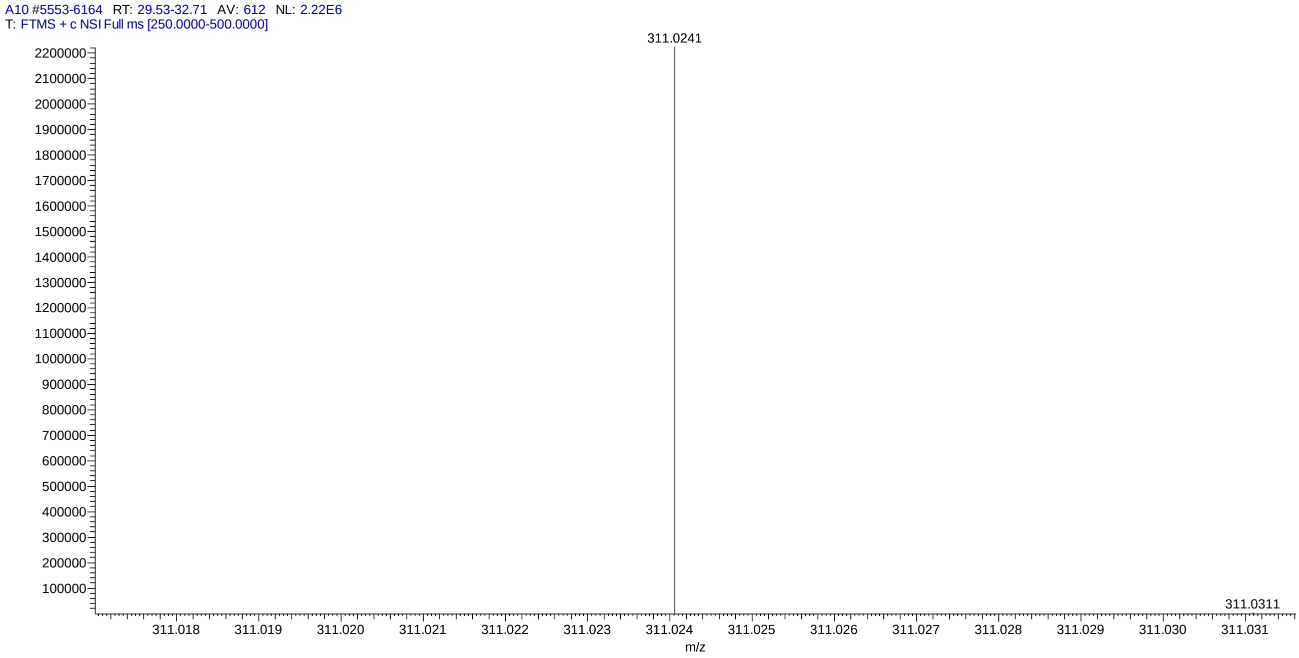

Figure 98. HRMS of Compound A10

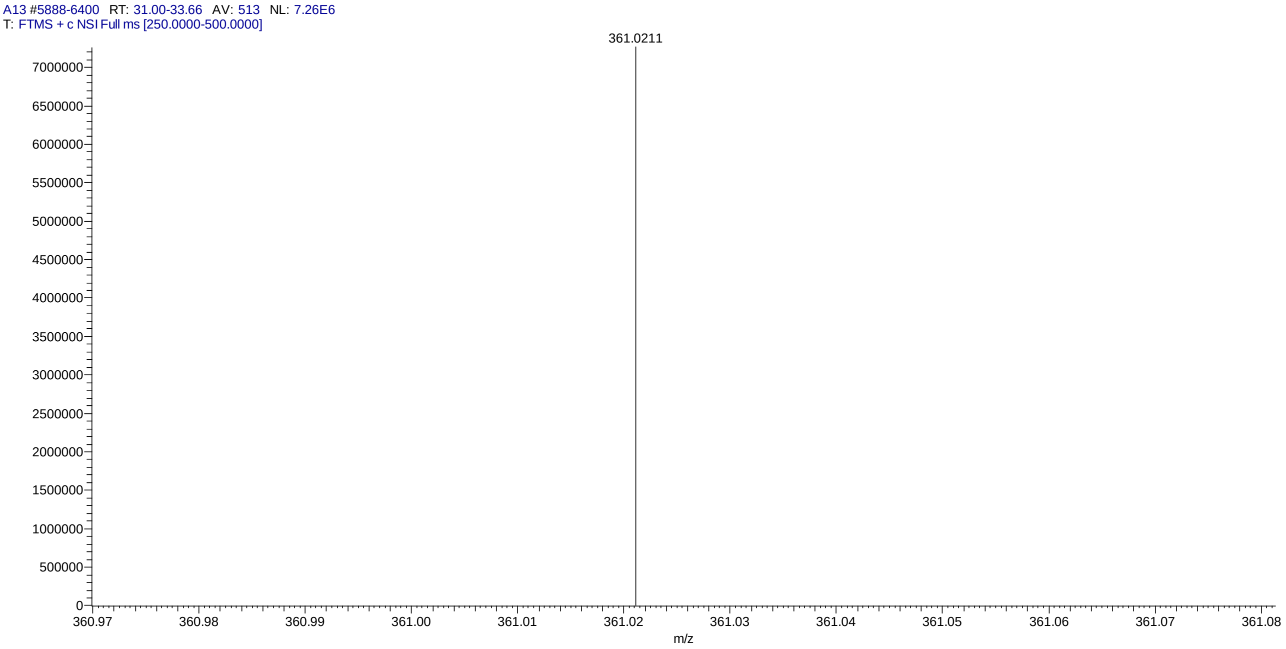

Figure 99. HRMS of Compound A11

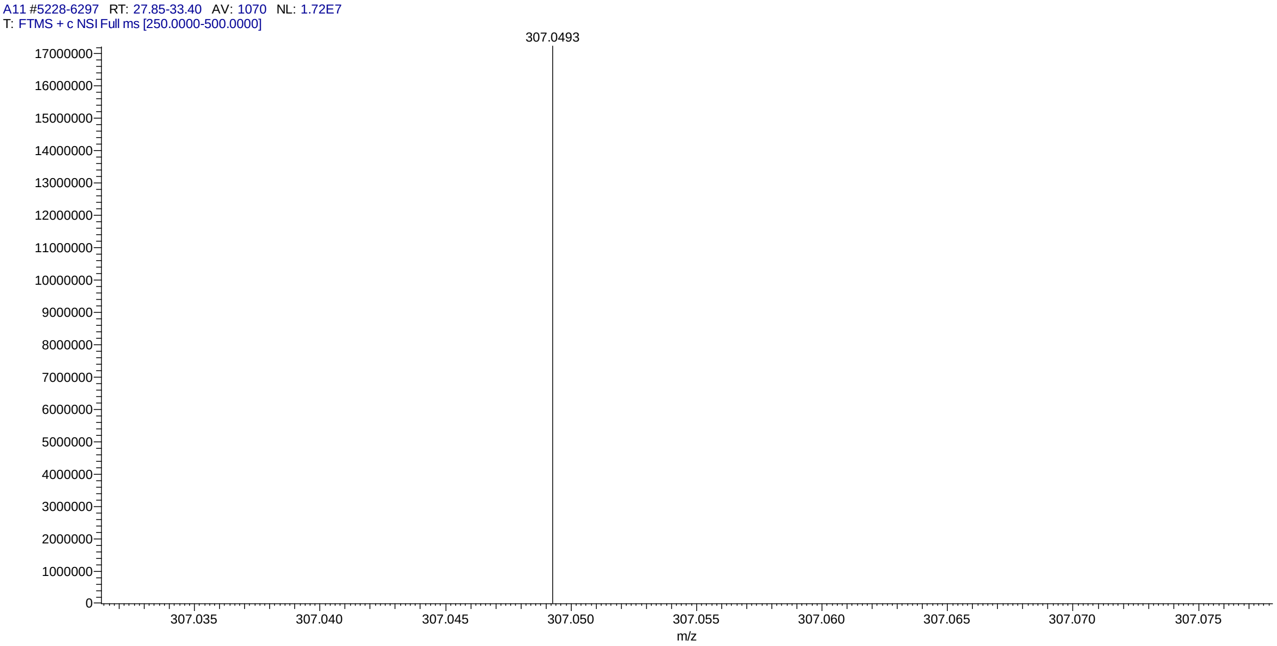

Figure 100. HRMS of Compound A12

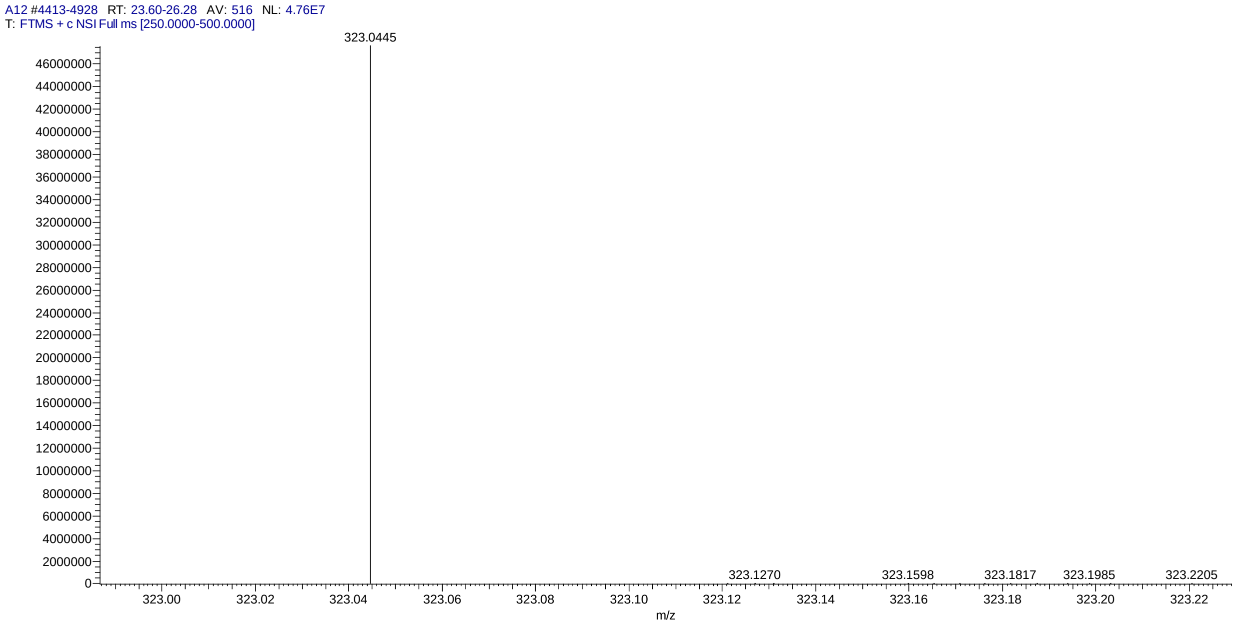

Figure 101. HRMS of Compound A13

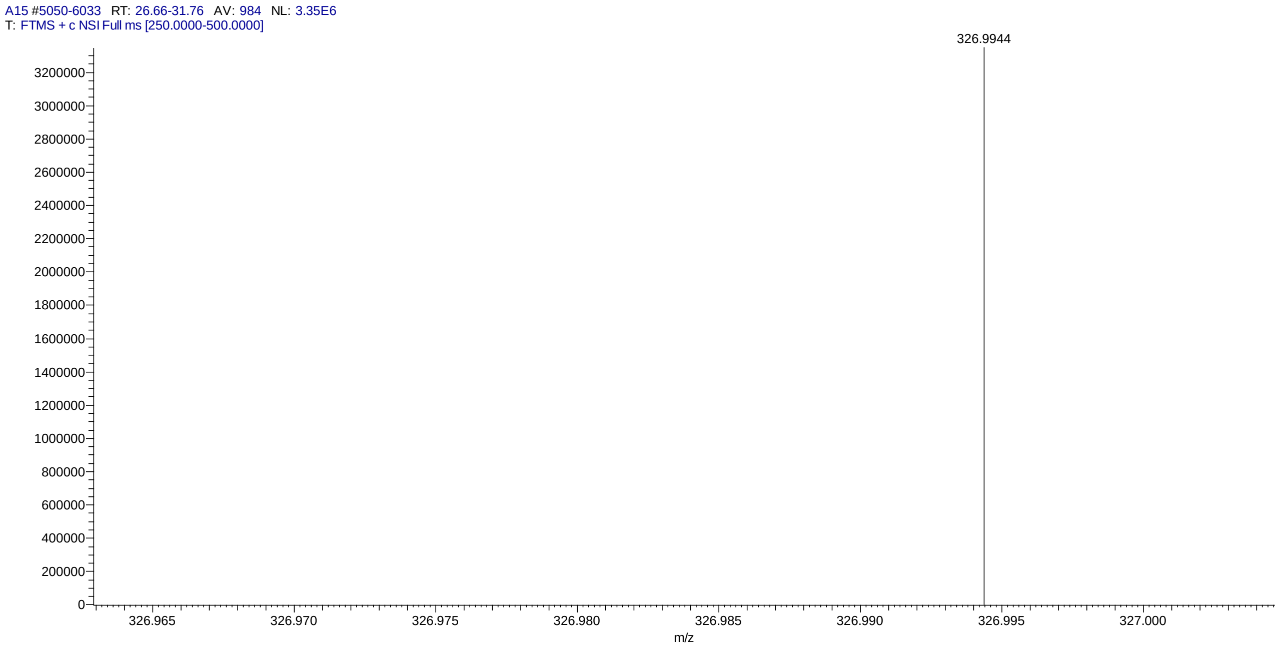

Figure 102. HRMS of Compound A14

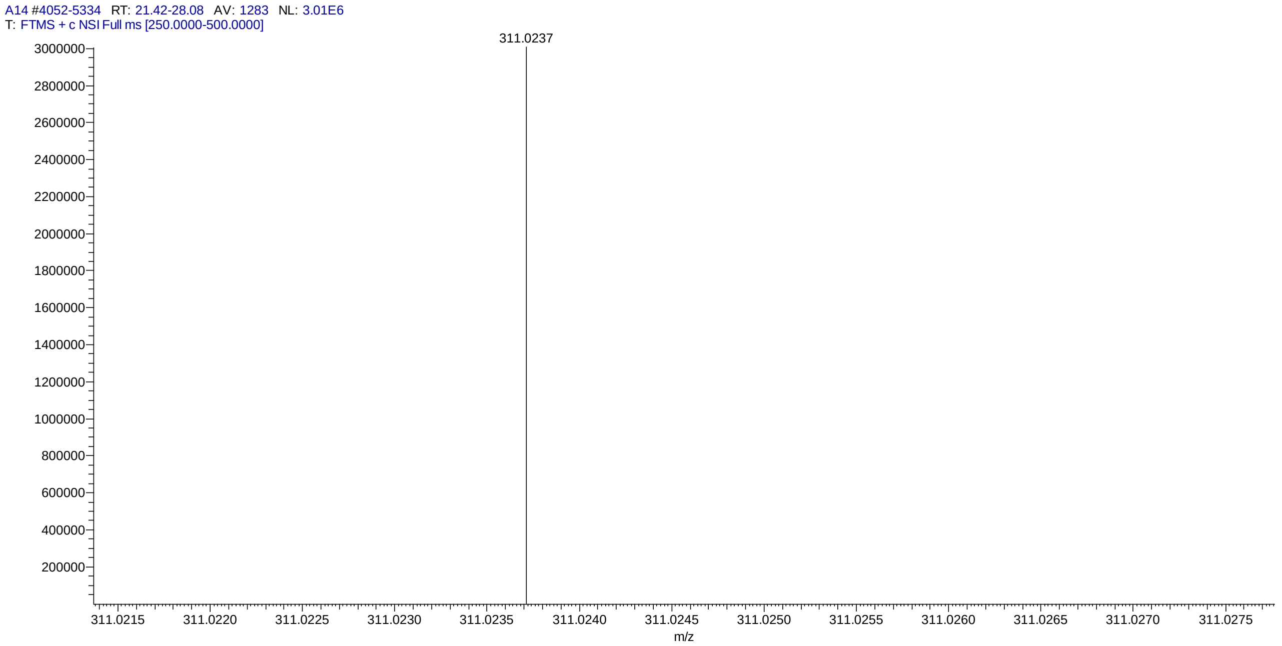

Figure 103. HRMS of Compound A15

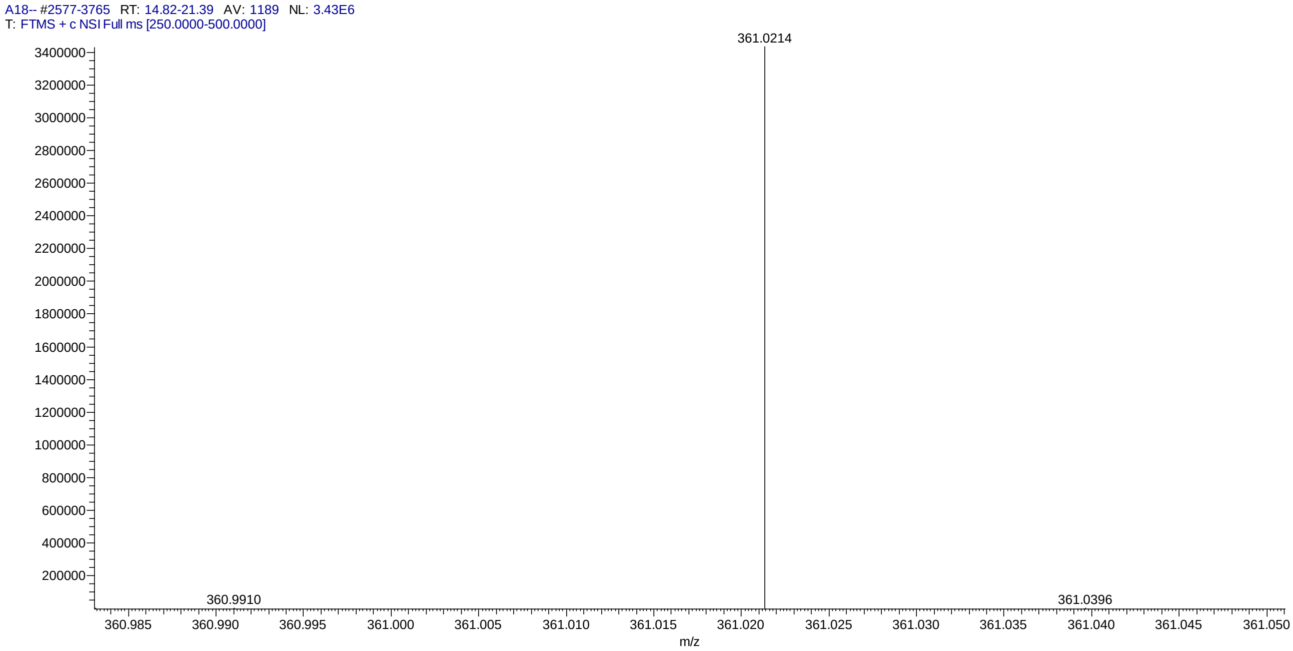

Figure 104. HRMS of Compound A16

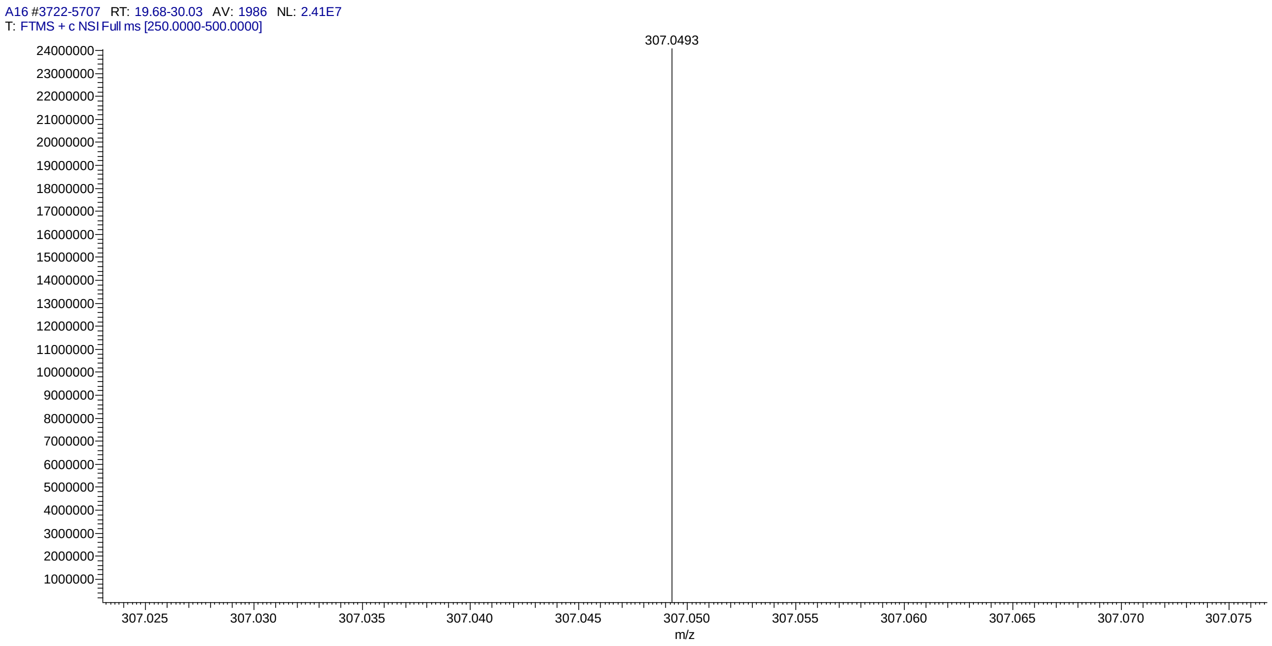

Figure 105. HRMS of Comppound A17

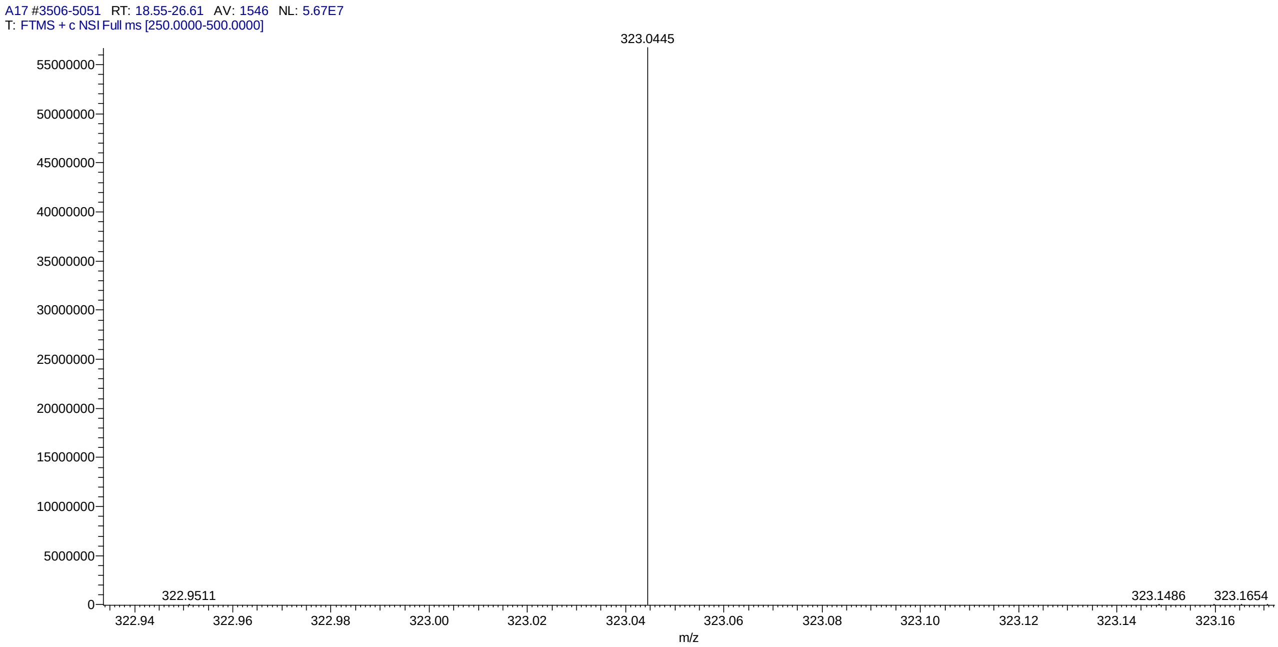

Figure 106. HRMS of Compound A18

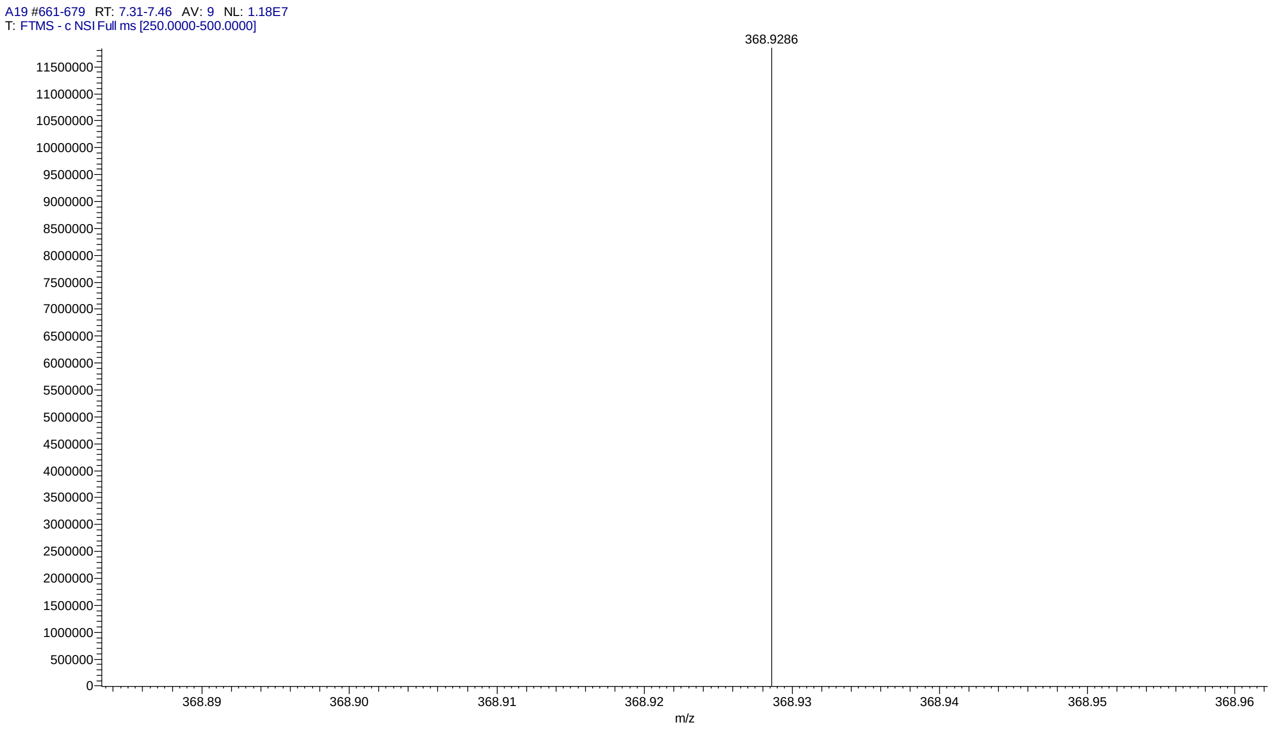

Figure 107. HRMS of Compound A19

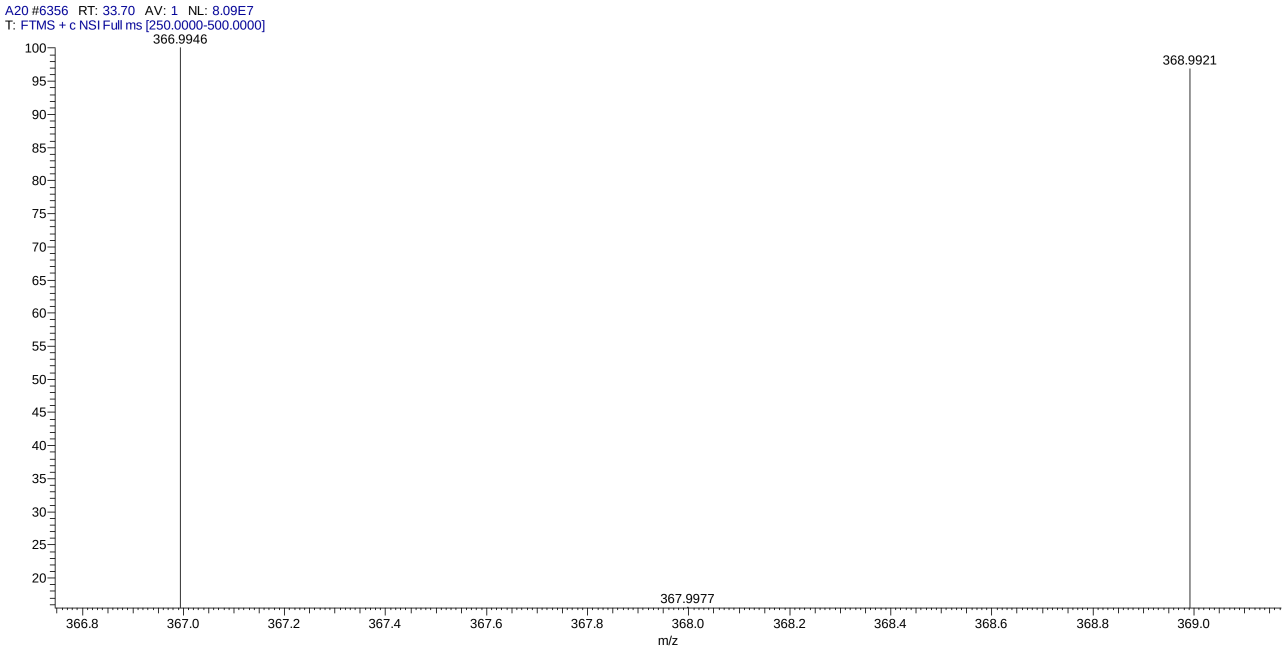

Figure 108. HRMS of Compound A20

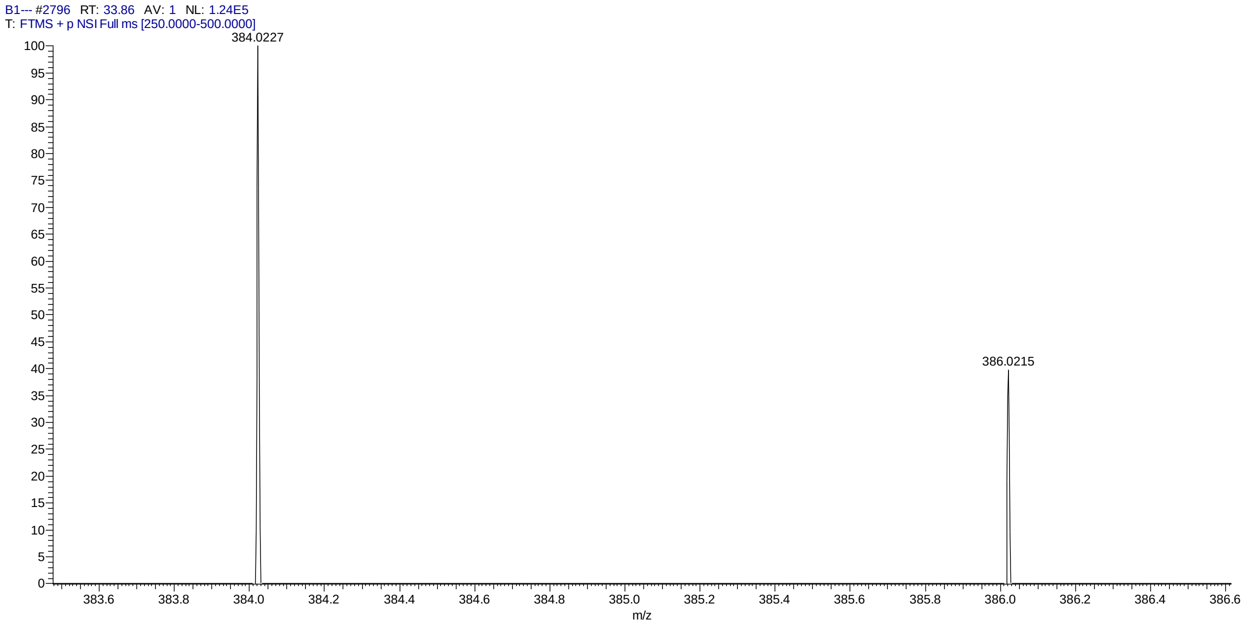

Figure 109. HRMS of Compound B1

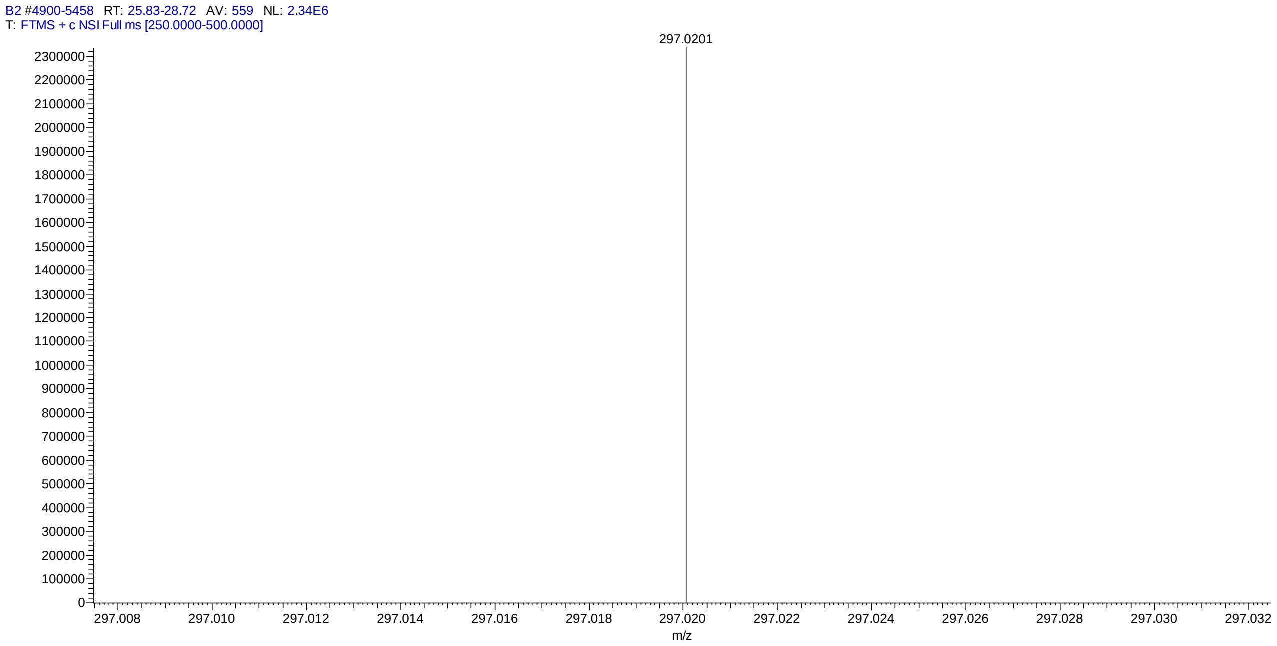

Figure 110. HRMS of Compound B2

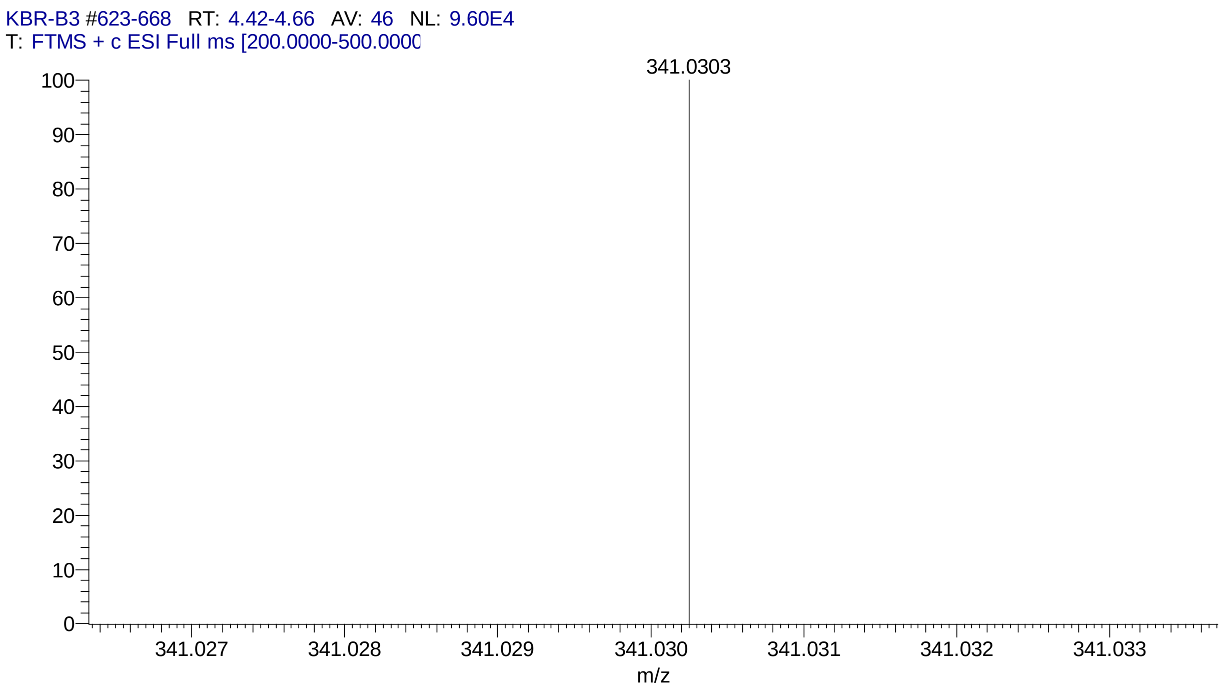

Figure 111. HRMS of Compound B3

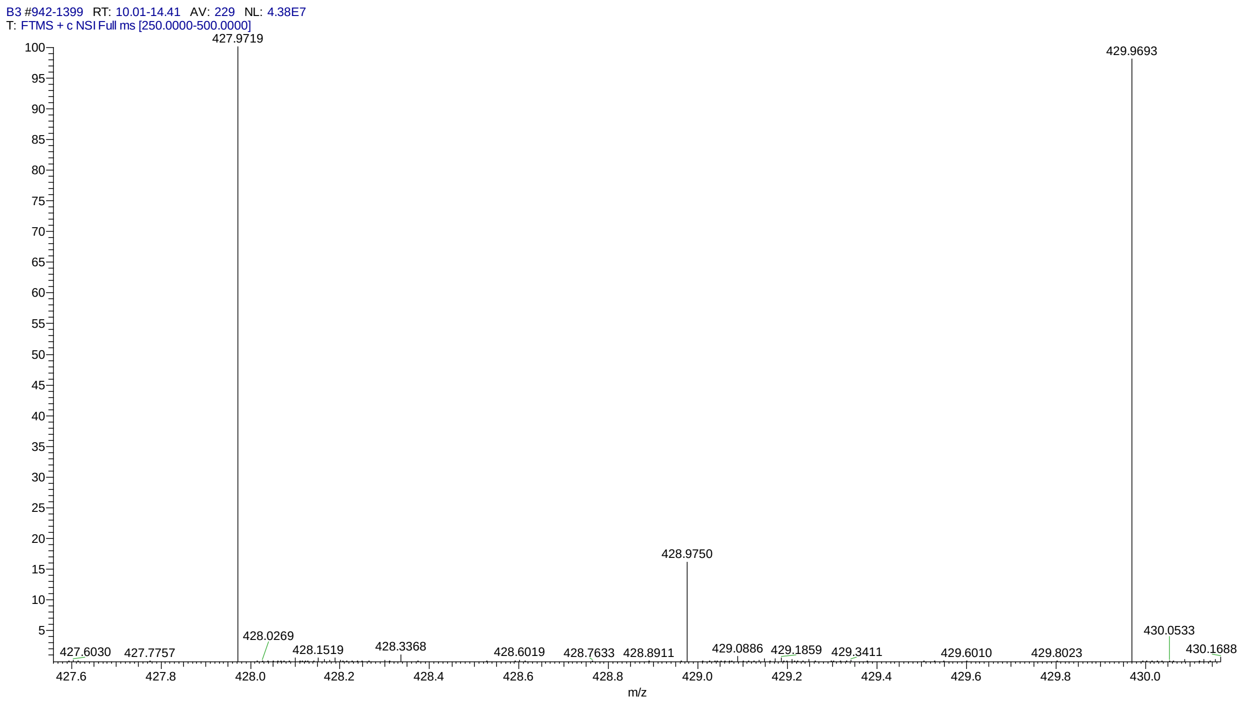

Figure 112. HRMS of Compound B4

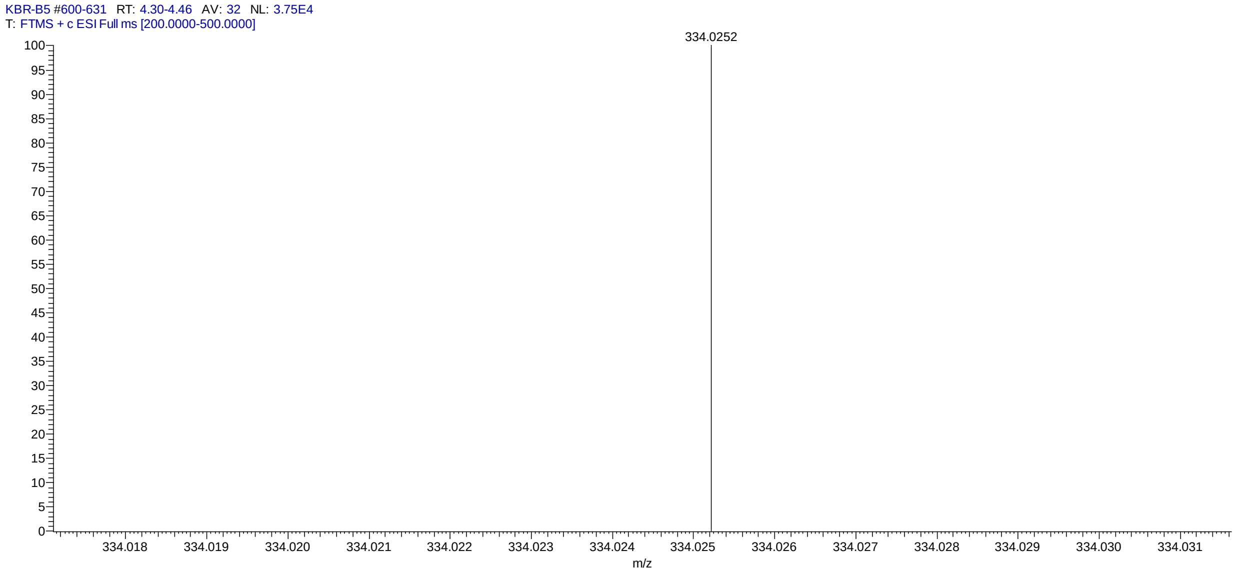

Figure 113. HRMS of Compound B5

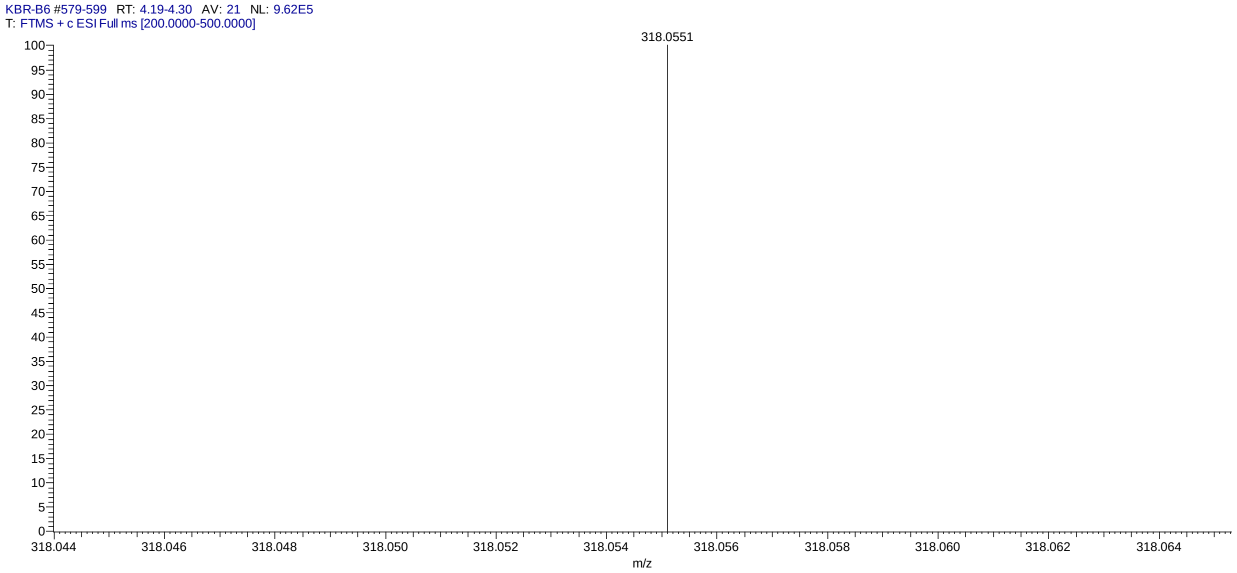

Figure 114. HRMS of Compound B6

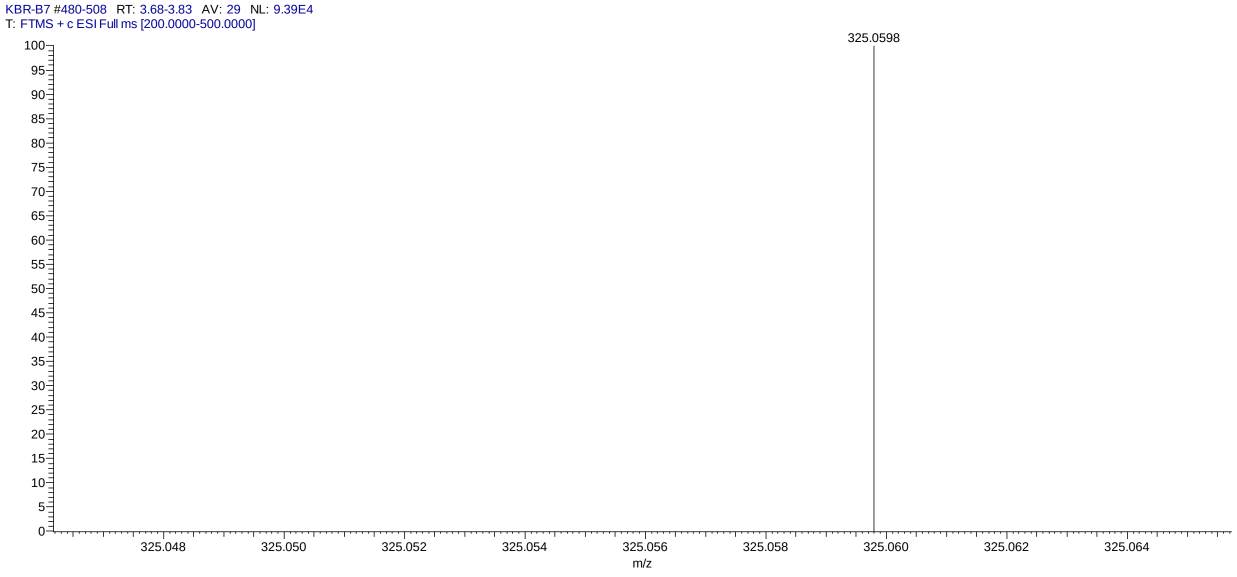

Figure 115. HRMS of Compound B7

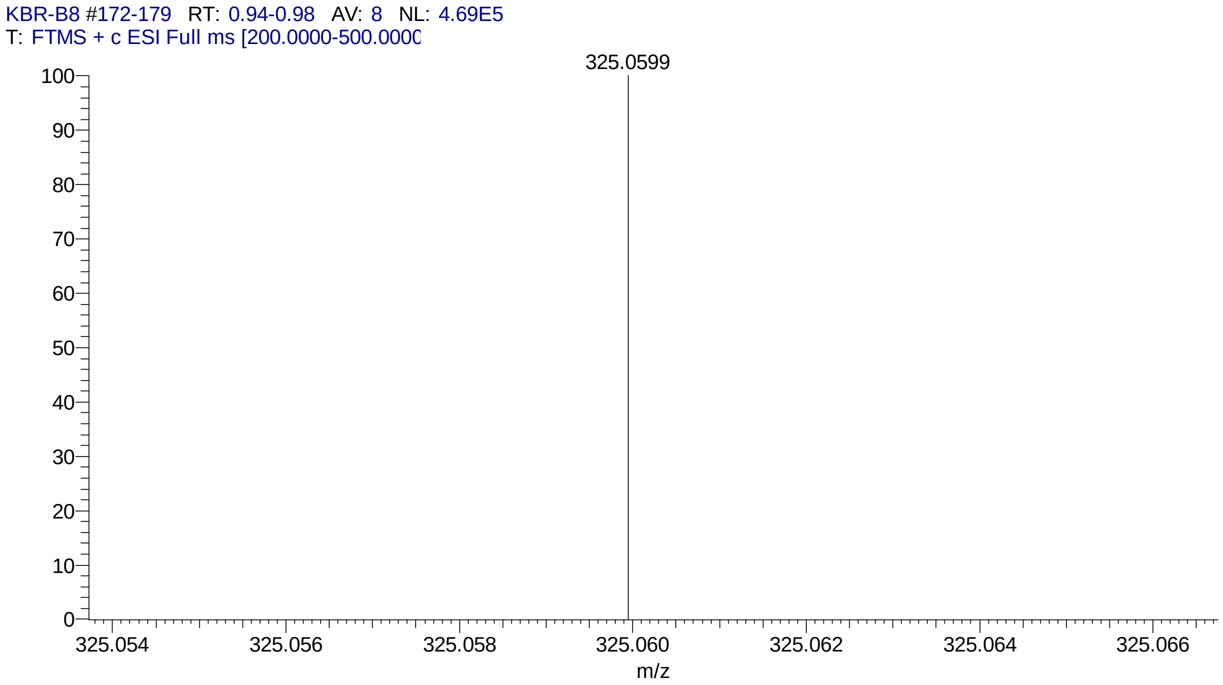

Figure 116. HRMS of Compound B8

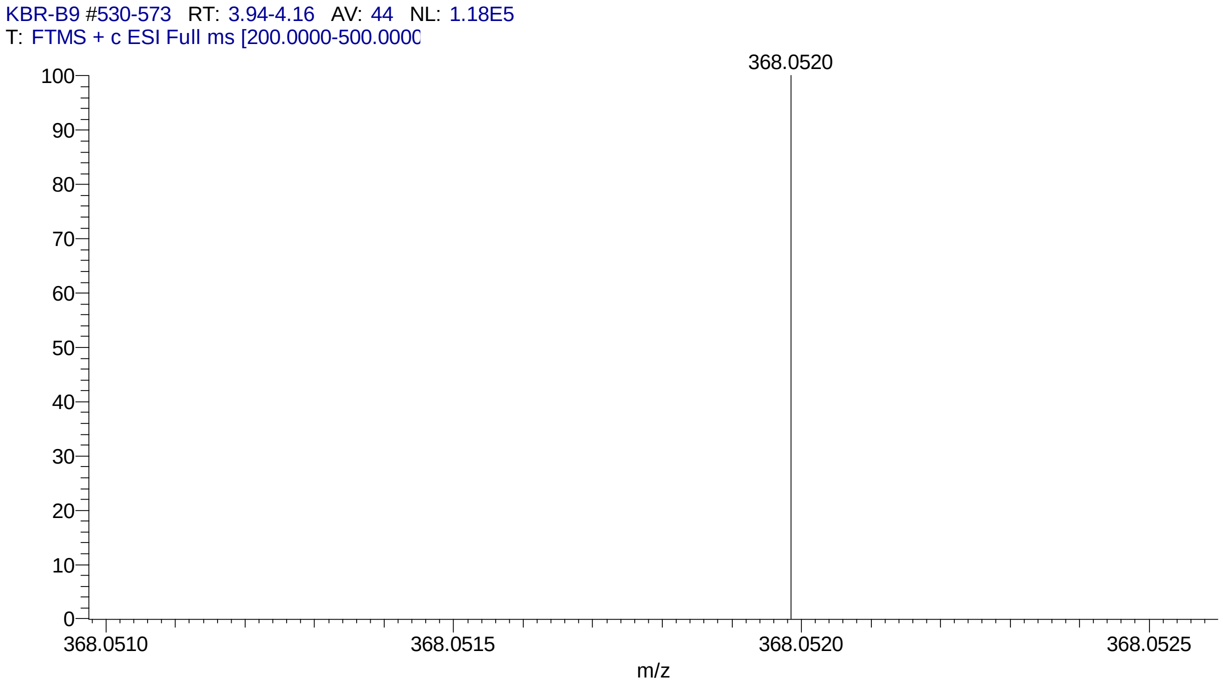

Figure 117. HRMS of Compound B9

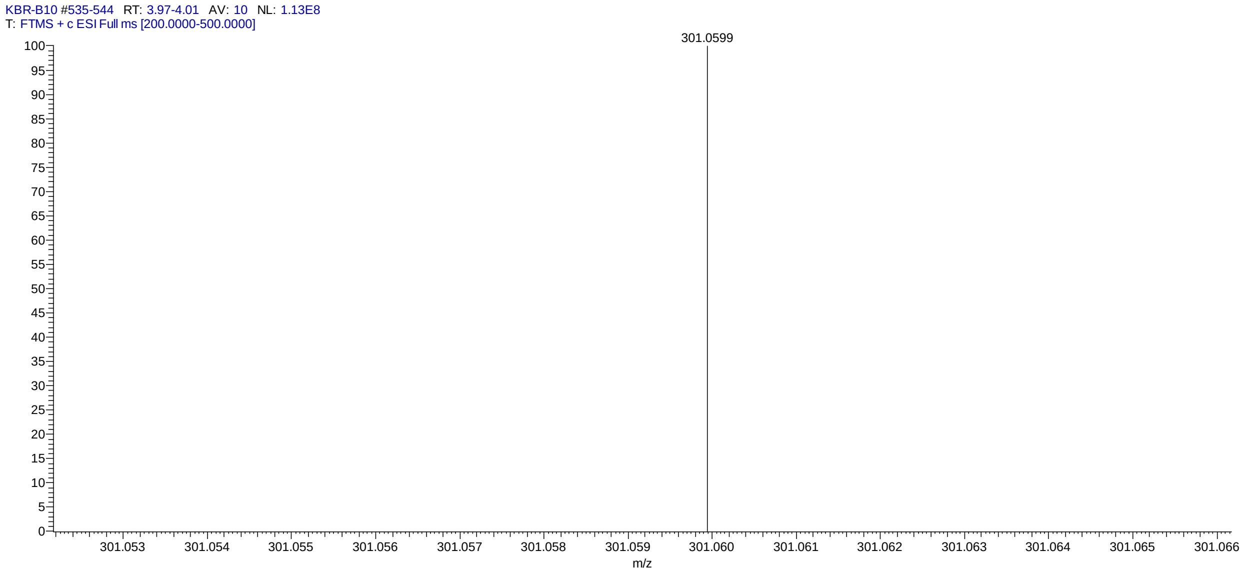

Figure 118. HRMS of Compound B10

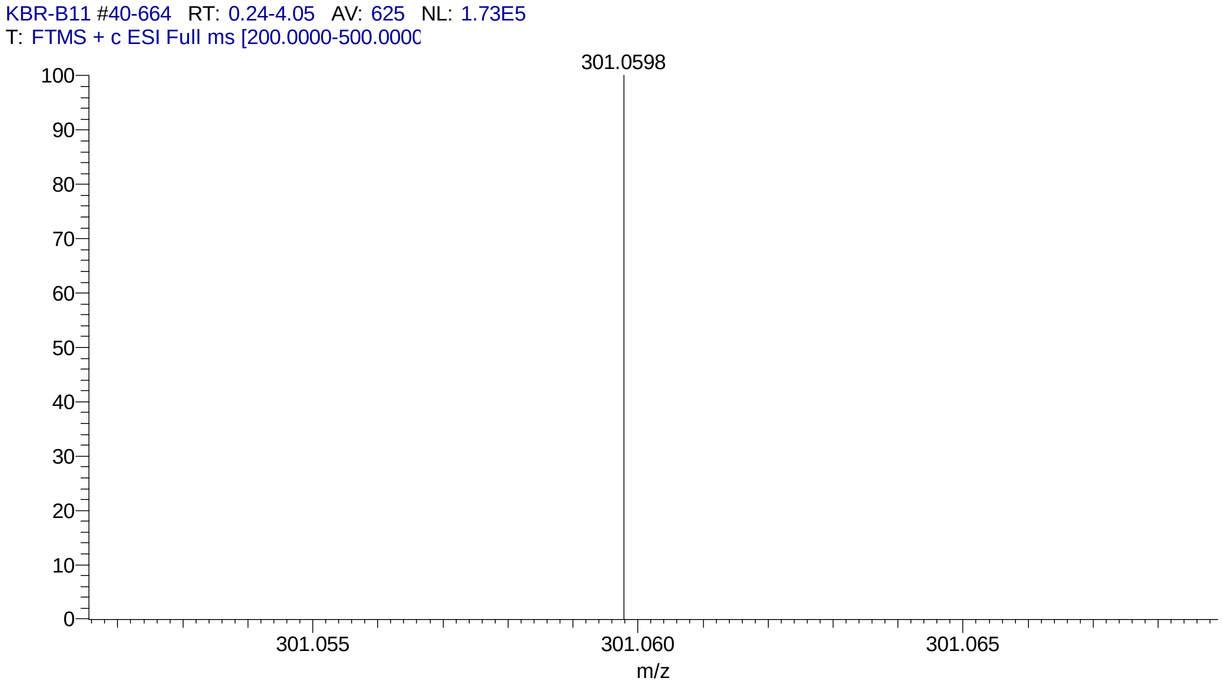

Figure 119. HRMS of Compound B11

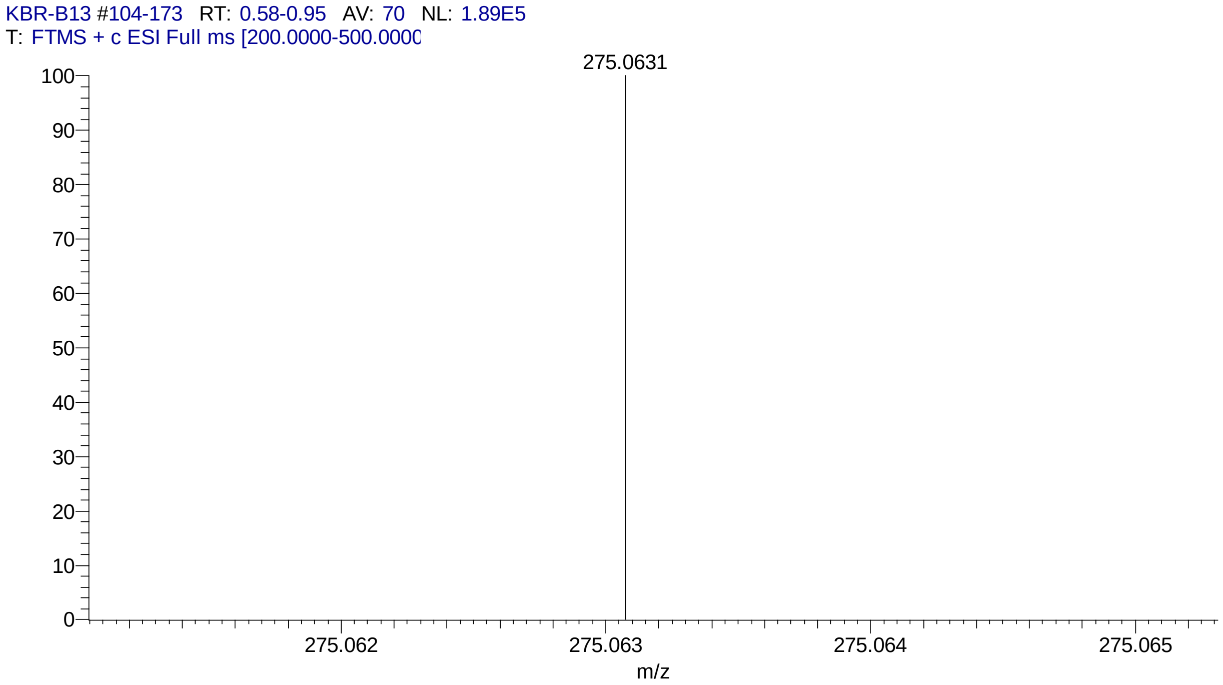

Figure 120. HRMS of Compound B13

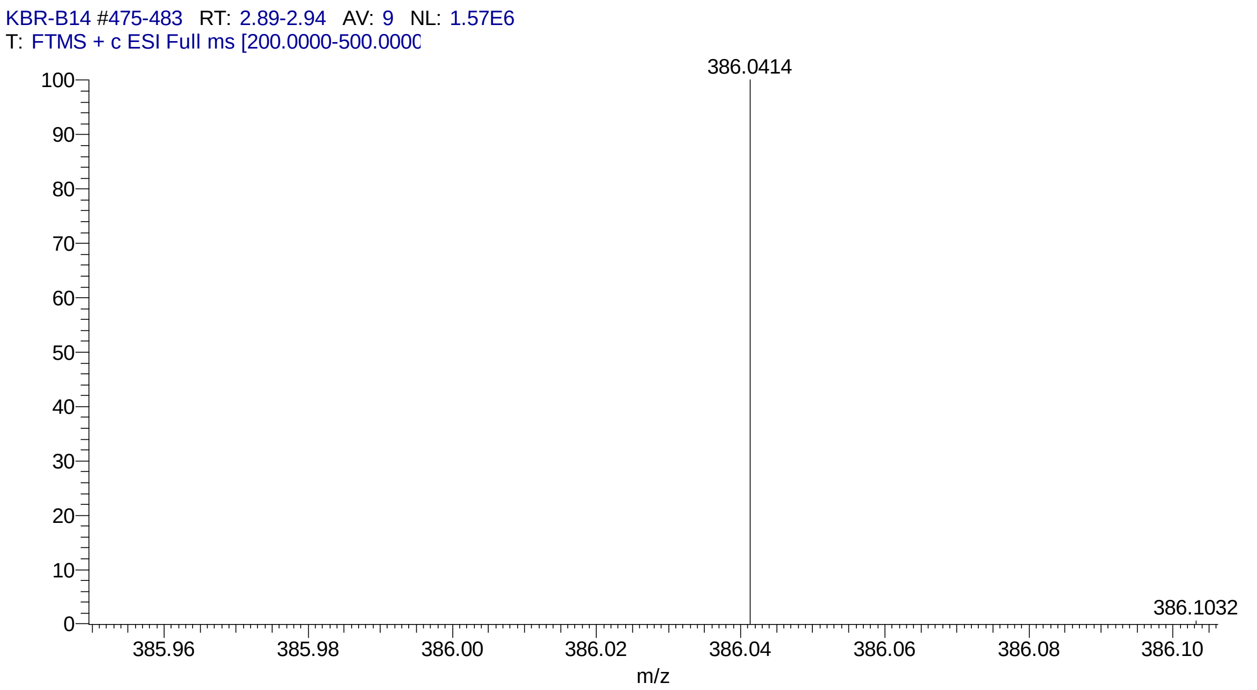

Figure 121. HRMS of Compound B14

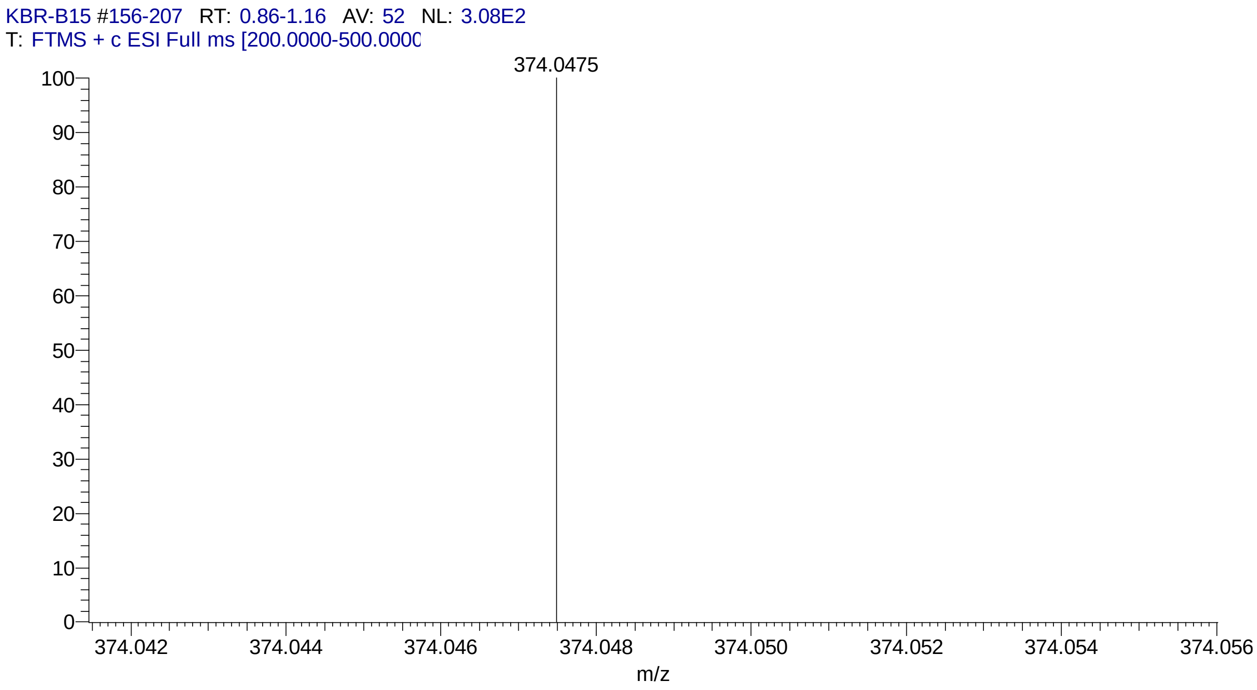

Figure 122. HRMS of Compound B15

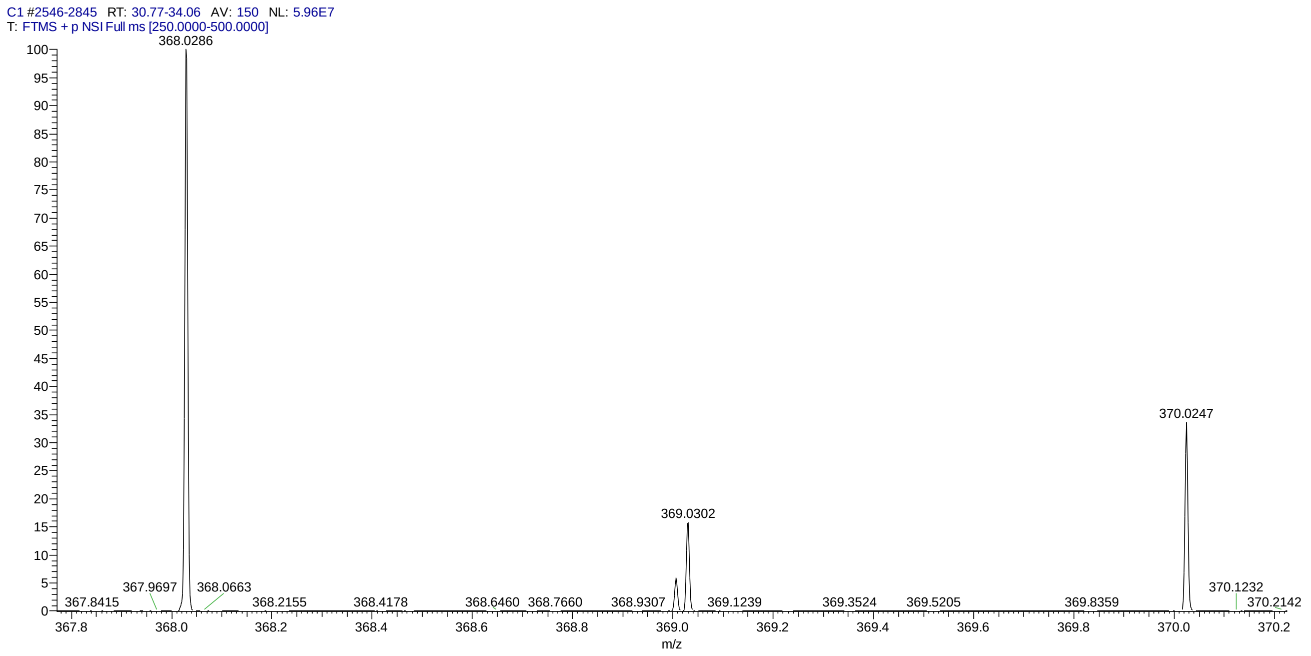

Figure 123. HRMS of Compound C1

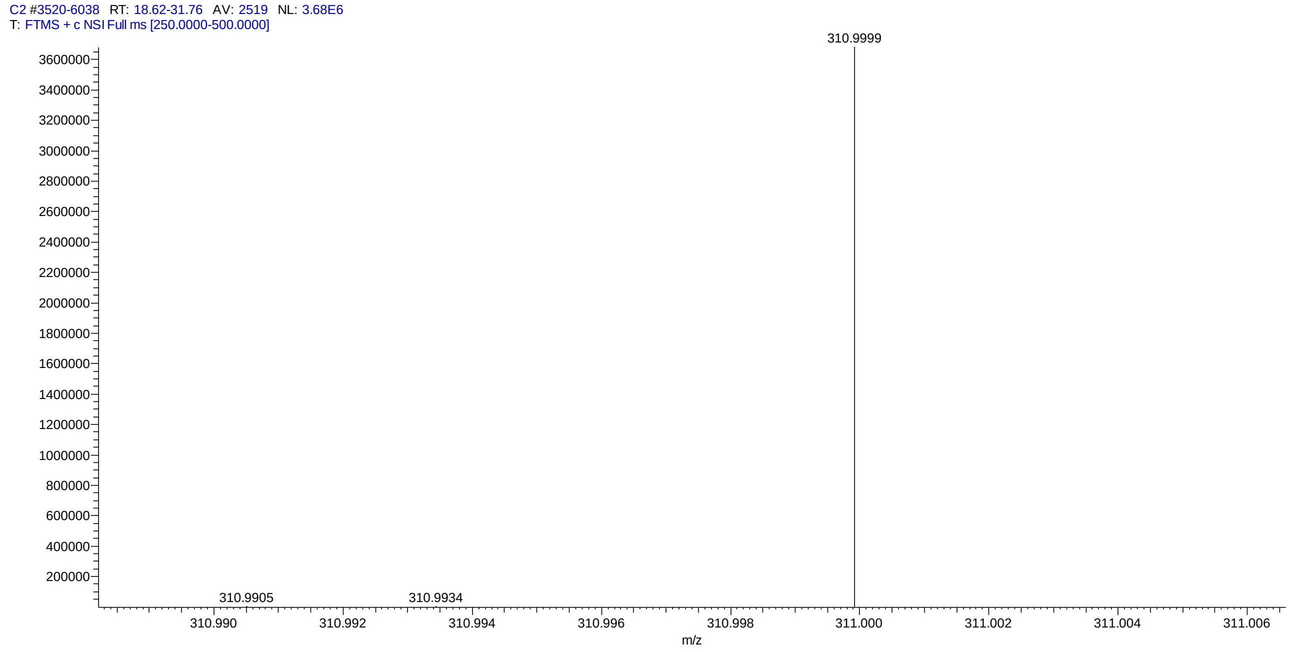

Figure 124. HRMS of Compound C2

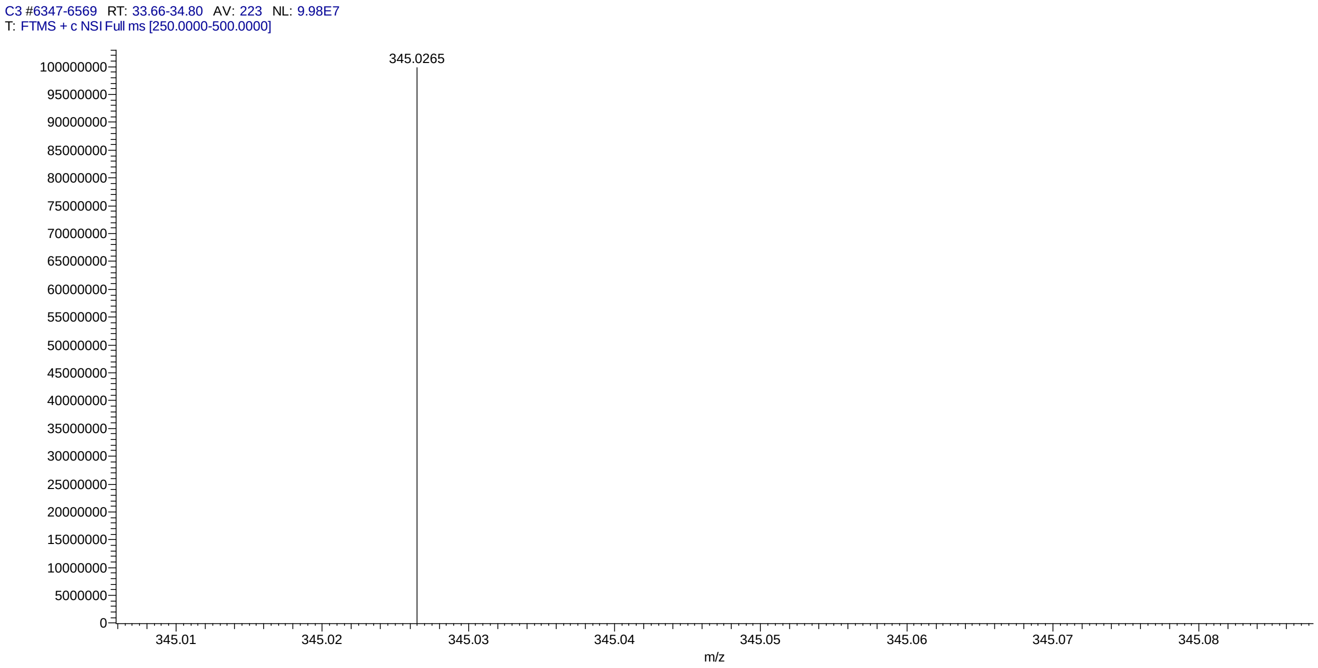

Figure 125. HRMS of Compound C3

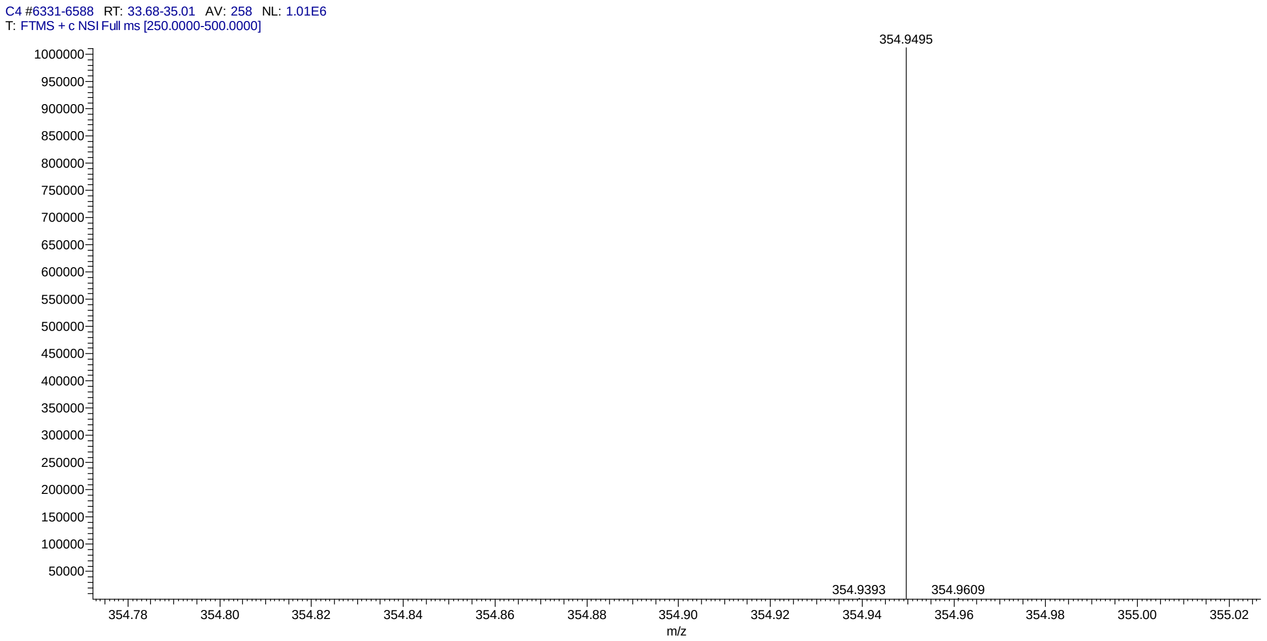

Figure 126. HRMS of Compound C4

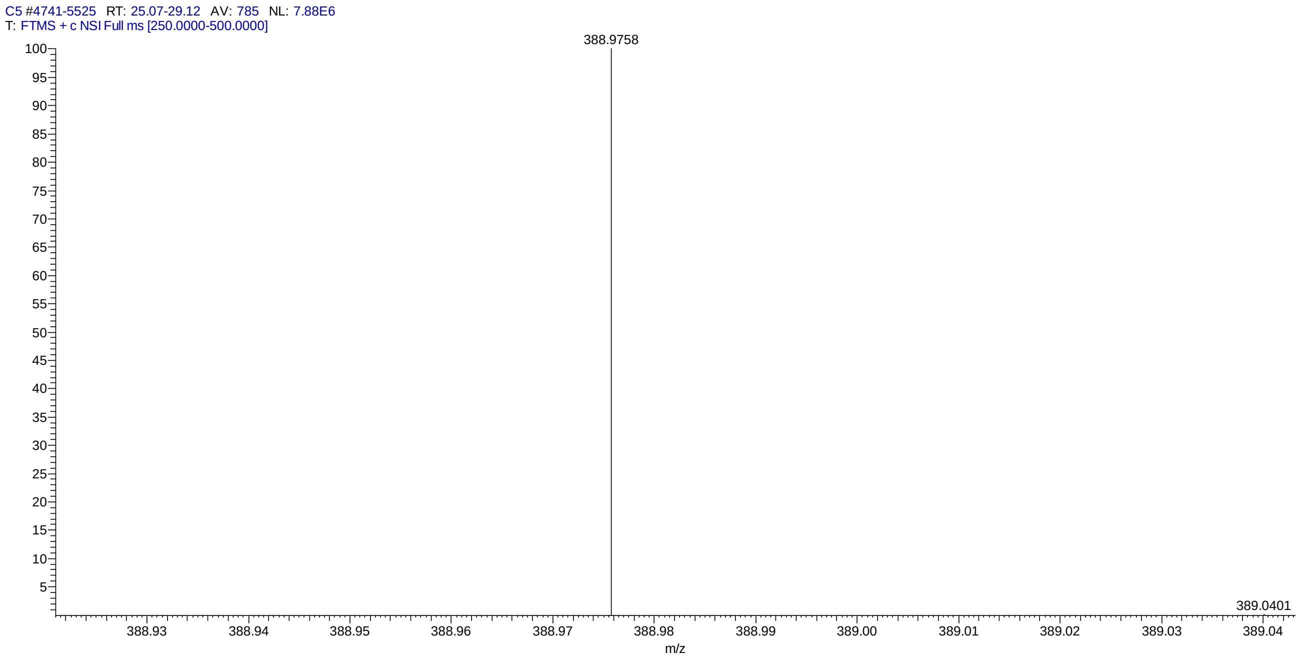

Figure 127. HRMS of Compound C5

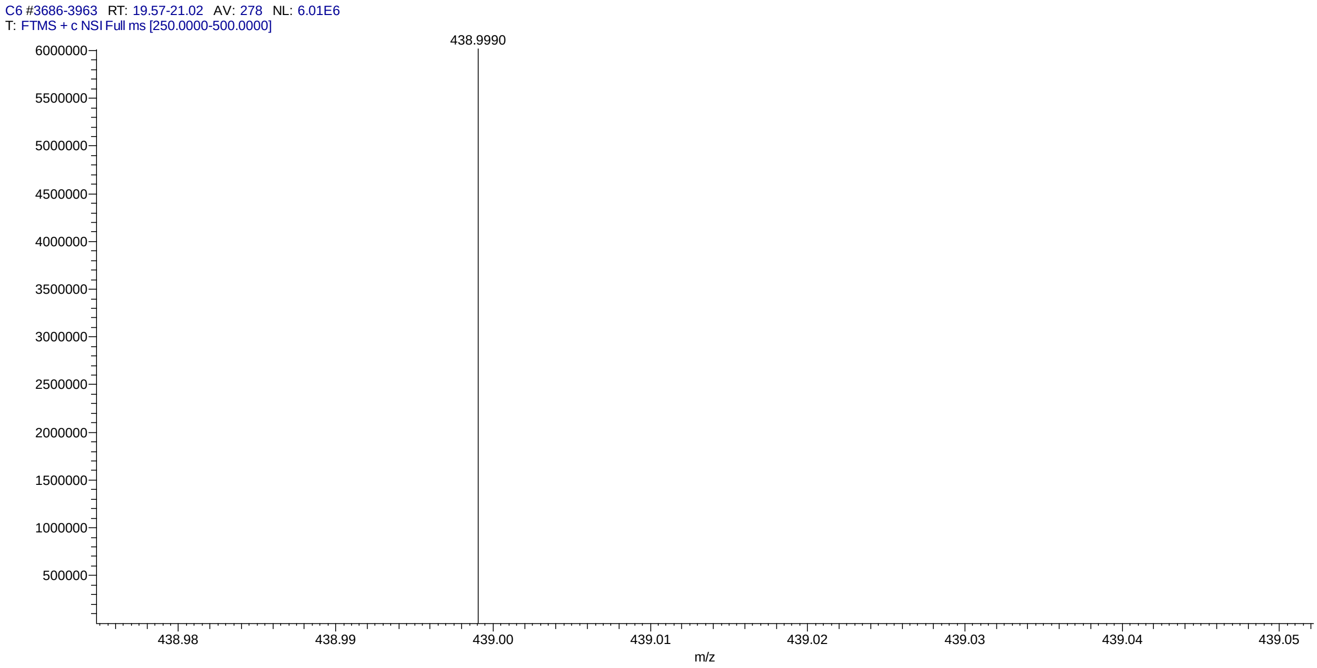

Figure 128. HRMS of Compound C6

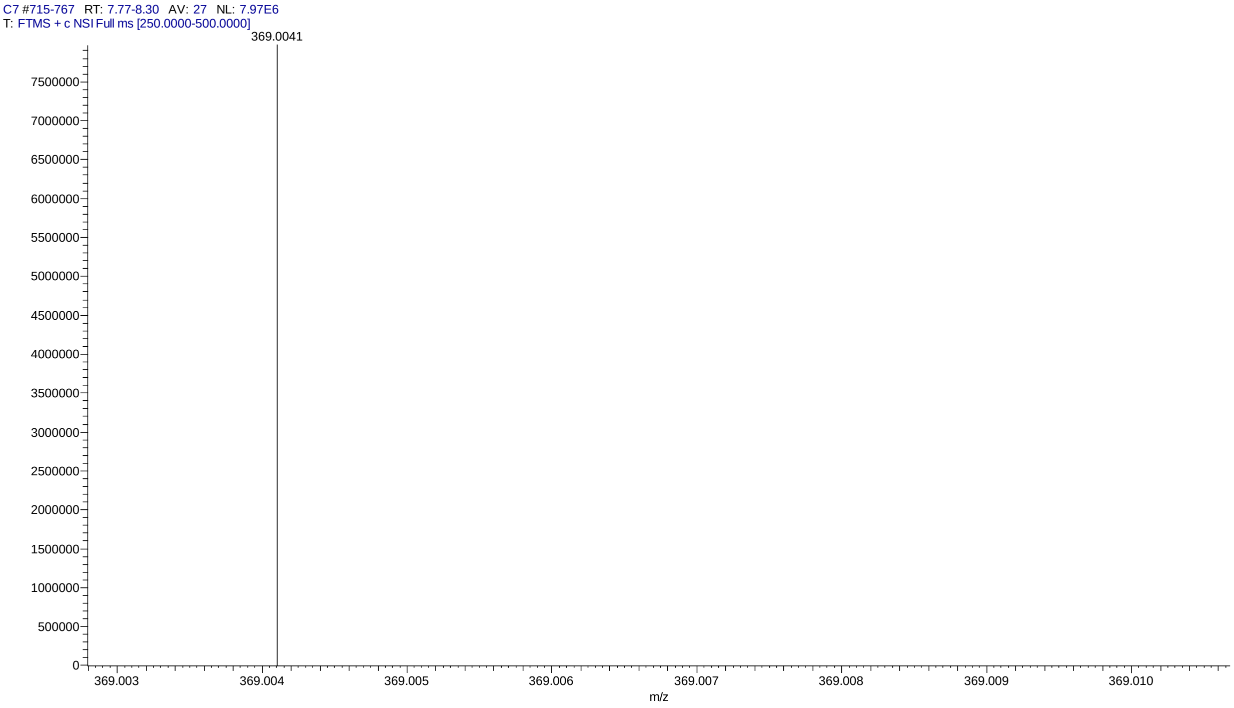

Figure 129. HRMS of Compound C7

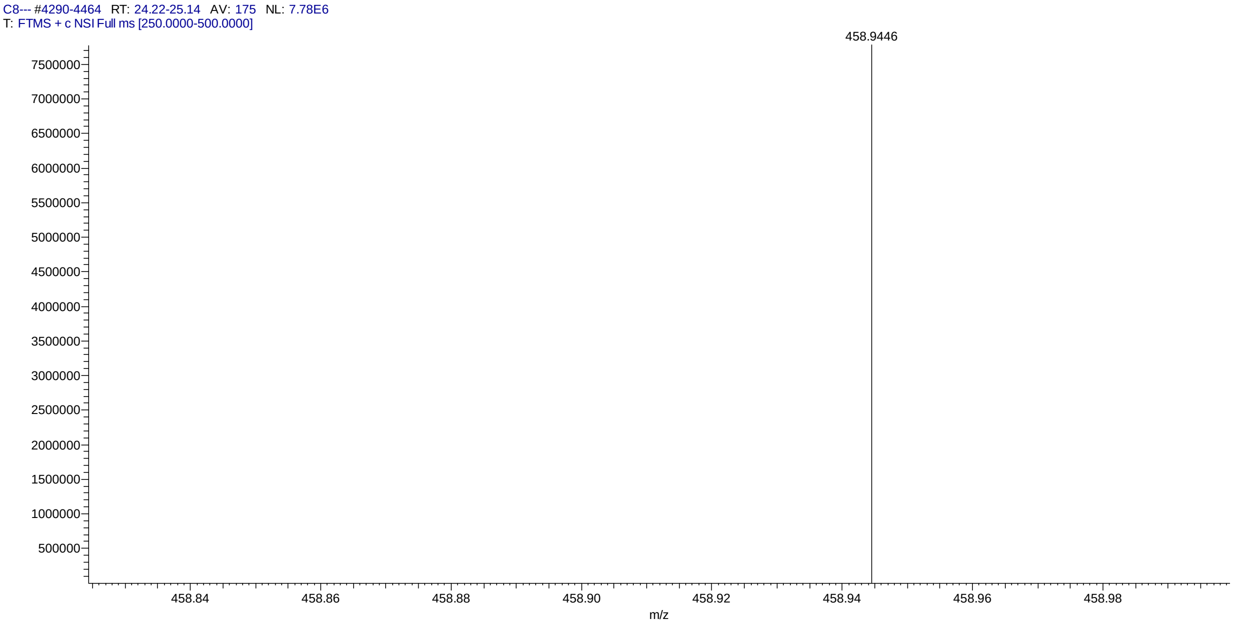

Figure 130. HRMS of Compound C8

6. In Silico ADME Prediction

Table S1. In silico ADME properties of niclosamide analogs (**A** series compounds) predicted by QikProp.

Compound	MW	SASA	HBD	HBA	QPlogPo/w	QPlogS	QPPCaco	QPlogBB	QPPMDCK	QPlogKp	QPlogKhsa	HOA	PSA
**A1**	310.669	532.93	1	3.25	2.97	-4.66	179.51	-1.17	321.13	-3.69	0.21	3	99.27
**A2**	306.705	538.83	1	3.25	2.94	-4.59	216.26	-1.15	233.09	-3.60	0.30	3	98.16
**A3**	322.704	565.74	1	4.00	2.78	-4.65	176.51	-1.38	187.15	-3.68	0.20	3	106.27
**A4**	327.123	545.26	1	3.25	3.16	-4.93	211.25	-1.06	493.57	-3.55	0.28	3	99.56
**A5**	310.669	532.63	1	3.25	2.98	-4.65	194.18	-1.14	338.01	-3.60	0.21	3	100.31
**A6**	360.676	565.11	1	3.25	3.64	-5.51	268.63	-0.86	1075.72	-3.39	0.40	3	97.02
**A7**	306.705	549.02	1	3.25	3.00	-4.77	241.12	-1.15	262.18	-3.46	0.30	3	98.07
**A8**	322.704	566.70	1	4.00	2.84	-4.66	203.84	-1.32	218.66	-3.53	0.20	3	106.25
**A9**	292.678	521.94	1	3.25	2.70	-4.26	193.68	-1.22	206.63	-3.50	0.16	3	99.60
**A10**	310.669	533.22	1	3.25	2.97	-4.67	179.03	-1.17	320.04	-3.69	0.21	3	99.31
**A12**	306.705	539.07	1	3.25	2.94	-4.59	215.77	-1.15	232.21	-3.60	0.30	3	98.19
**A13**	322.704	566.61	1	4.00	2.79	-4.66	175.46	-1.38	185.70	-3.68	0.20	3	106.32
**A14**	327.123	545.64	1	3.25	3.16	-4.94	210.82	-1.06	491.81	-3.55	0.28	3	99.61
**A15**	310.669	533.04	1	3.25	2.99	-4.65	194.06	-1.14	337.24	-3.60	0.21	3	100.34
**A16**	360.676	565.58	1	3.25	3.64	-5.51	268.08	-0.87	1072.01	-3.39	0.40	3	97.08
**A17**	306.705	549.59	1	3.25	3.01	-4.78	240.57	-1.15	261.20	-3.46	0.31	3	98.14
**A18**	322.704	566.94	1	4.00	2.84	-4.67	203.37	-1.32	217.83	-3.53	0.20	3	106.29
**A20**	367.155	571.29	1	4.00	2.85	-4.77	175.90	-1.38	200.23	-3.68	0.22	3	106.31

The descriptors and desired values are molecular weight (MW): < 500 Da, Solvent accessible surface area (SASA): 300-1000 $Å^2$, hydrogen bond donor (HBD): 0-6, hydrogen bond acceptor (HBA): 2-20.0, predicted octanol/water partition coefficient (QPlogPo/w): -2.0-6.5, predicted aqueous solubility (QPlogS): -6.5-0.5 mol dm^{-3}, predicted apparent Caco-2 cell permeability in nm/sec (QPPCaco): <25 poor; >500 great, predicted brain/blood partition coefficient (QPlogBB): -3.0-1.2, predicted apparent MDCK cell permeability in nm/sec (QPPMDCK): <25 poor; >500 great, predicted skin permeability (QPlogKp): -8.0--1.0, prediction of binding to human serum albumin (QPlogKhsa): -1.5-1.5, human oral absorption (HOA): 1, 2, or 3 for low, medium or high, and Van der Waals surface area of polar nitrogen and oxygen atoms (PSA): 2-15.

Table S2. In silico ADME properties of niclosamide analogs (**B** and **C** series compounds) predicted by QikProp.

Compound	MW	SASA	HBD	HBA	QPlogPo/w	QPlogS	QPPCaco-2	QPlogBB	QPPMDCK	QPlogKp	QPlogKhsa	HOA	PSA
**B1**	383.677	570.37	1	2.25	5.07	-6.51	1629.76	0.23	10000.00	-1.98	0.68	1	54.57
**B3**	340.689	562.05	1	3.75	3.57	-6.01	436.45	-0.64	1919.19	-2.93	0.28	3	80.34
**B4**	428.128	575.47	1	2.25	5.15	-6.63	1629.43	0.24	10000.00	-1.98	0.70	1	54.56
**B5**	333.669	533.80	1	2.25	4.54	-5.53	1796.98	0.19	10000.00	-1.86	0.48	3	53.08
**B6**	317.215	532.37	1	2.25	4.36	-5.45	1503.17	0.05	10000.00	-1.98	0.43	3	53.98
**B7**	324.234	546.88	1	3.75	3.38	-5.86	450.77	-0.63	1659.07	-2.90	0.22	3	79.07
**B8**	324.234	548.38	1	3.75	3.30	-5.88	421.25	-0.70	1343.33	-2.92	0.20	3	80.28
**B9**	367.223	555.99	1	2.25	4.80	-6.14	1628.52	0.18	10000.00	-1.94	0.60	3	54.56
**B10**	300.212	517.68	1	3.25	3.43	-4.78	1011.53	-0.20	4158.75	-2.30	0.17	3	67.85
**B11**	300.212	516.58	1	3.25	3.55	-4.79	1127.14	-0.14	4822.51	-2.20	0.17	3	65.92
**B12**	290.681	519.58	1	3.75	2.92	-4.65	372.85	-0.83	657.88	-2.98	0.07	3	79.03
**B13**	274.226	516.86	1	3.75	2.78	-4.59	313.60	-0.98	423.28	-3.09	0.02	3	79.79
**B14**	385.213	563.85	1	2.25	5.01	-6.46	1628.52	0.27	10000.00	-2.06	0.64	1	54.56
**B15**	374.242	584.23	1	3.75	3.82	-6.76	340.35	-0.74	1799.54	-3.16	0.40	1	80.40
**B16**	417.230	600.37	1	2.25	5.59	-7.27	1628.52	0.33	10000.00	-2.05	0.83	1	54.56
**C1**	367.678	561.88	1	2.50	5.15	-6.44	3746.60	0.67	10000.00	-1.27	0.62	1	35.42
**C3**	344.677	557.12	1	3.50	3.73	-5.44	616.55	-0.43	2638.69	-2.68	0.34	3	77.90
**C5**	389.128	562.20	1	3.50	3.80	-5.56	616.46	-0.42	2837.57	-2.68	0.37	3	77.88
**C6**	438.762	640.35	1	8.00	2.48	-4.94	153.28	-1.26	566.62	-3.85	-0.15	3	122.74
**C7**	369.160	601.52	1	6.00	2.85	-5.01	288.46	-0.96	703.76	-3.37	0.07	3	113.00
**C8**	459.180	629.46	1	8.00	2.58	-5.05	134.64	-1.12	1131.81	-3.98	-0.19	3	123.27
**NIC**[a]	327.123	536.31	1	3.25	3.12	-4.80	212.32	-1.02	497.61	-3.58	0.26	3	98.64

The descriptors and desired values are molecular weight (MW): < 500 Da, Solvent accessible surface area (SASA): 300-1000 Å^2, hydrogen bond donor (HBD): 0-6, hydrogen bond acceptor (HBA): 2-20.0, predicted octanol/water partition coefficient (QPlogPo/w): -2.0-6.5, predicted aqueous solubility (QPlogS): -6.5-0.5 mol dm^{-3}, predicted apparent Caco-2 cell permeability in nm/sec (QPPCaco): <25 poor; >500 great, predicted brain/blood partition coefficient (QPlogBB): -3.0-1.2, predicted apparent MDCK cell permeability in nm/sec (QPPMDCK): <25 poor; >500 great, predicted skin permeability (QPlogKp): -8.0--1.0, prediction of binding to human serum albumin (QPlogKhsa): -1.5-1.5, human oral absorption (HOA): 1, 2, or 3 for low, medium or high, and Van der Waals surface area of polar nitrogen and oxygen atoms (PSA): 2-15. [a]NIC = Niclosamide.
